# Supplementary material for: De novo design of protein peptides to block association of the SARS-CoV-2 spike protein with human ACE2
Source: Aging (Albany NY). 2020 Jun 16;12(12):11263–76. doi: 10.18632/aging.103416 (PMC7343451; doi:10.18632/aging.103416)
Supplement: Supplementary Figures [file aging-12-103416-s001..pdf]

SUPPLEMENTARY FIGURES

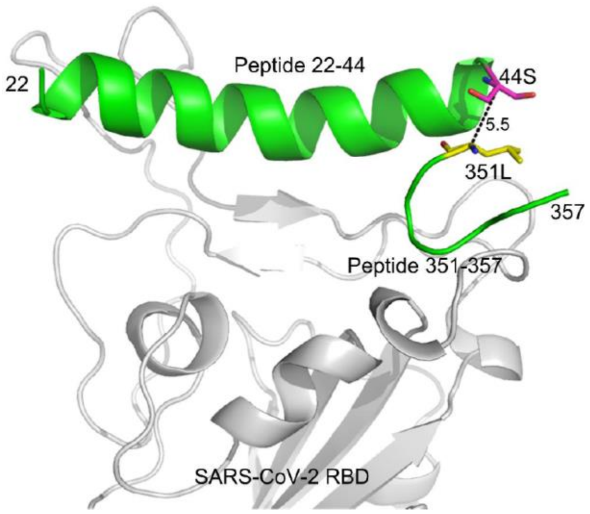

Supplementary Figure 1. Illustration of the scaffold fragments used for design. Two discontinuous peptide fragments (a.a. 22-44 and a.a. 351-357) were used for peptide scaffold construction. The hACE2 peptides and SARS-CoV-2 RBD are shown in green and gray, respectively. The residues Ser44 and Leu351 are shown in magenta and yellow sticks, respectively. The distance between the Cα atoms of Ser44 and Leu351 is 5.5 Å.

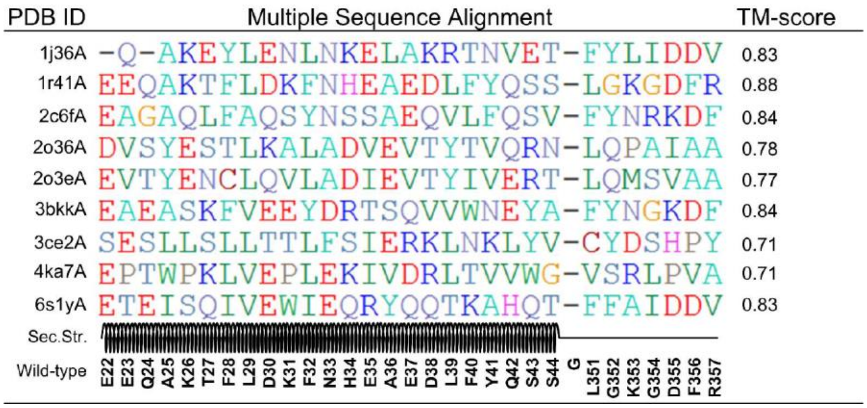

Supplementary Figure 2. Peptide multiple sequence alignment for evolutionary profile construction.
