# Supplementary material for: De novo design of protein peptides to block association of the SARS-CoV-2 spike protein with human ACE2
Source: Aging (Albany NY). 2020 Jun 16;12(12):11263–76. doi: 10.18632/aging.103416 (PMC7343451; doi:10.18632/aging.103416)
Supplement: Supplementary Tables [file aging-12-103416-s002..pdf]

Supplementary Tables

Table S1. Summary of 992 peptide sequences designed using EvoEF2 only. The table is sorted according to the binding score from the lowest to the highest. '#label' presents the index of the binder among the 1000 low total energy designs.

#label	#peptide	#binding(EEU)	#SeqID(%)	#Secondary structure
WT	EEQAKTFLDKFNHEAEDLFYQSSGLGKGDFR	-46.46	100.	HHHHHHHHHHHHHHHHHHHHHHHHCCCCCCC
728	EQEERIQQDKRKNEQEDKRYQRYGRGKGHQP	-53.35	38.7	CHHHHHHHHHHHHHHHHHHHHHHHCCCCCCC
513	EQQQRIQEDQYRNDWEDEEYQRKGRGKGHQP	-53.24	35.5	HHHHHHHHHHHHCHHHHHHHHHHHCCCCCCC
213	EQQQRIQQDQDSNDREDKEYQKRGKGKGHNH	-53.24	35.5	HHHHHHHHHHCCCCHHHHHHHHHHCCCCCCC
830	QEEQKIQEDQRRNDKEHKRKQRYGRGCGKQN	-53.20	25.8	CHHHHHHHHHHHHHHHHHHHHHHHCCCCCCC
138	EQEERIQRDKRKNEKEHEEKQRRGRGCGKQN	-53.20	29.0	CHHHHHHHHHHHHHHHHHHHHHHHCCCCCCC
585	EQEERIQQDKRKNENEDKRYQRYGRGKGHQP	-53.16	38.7	CHHHHHHHHHHHHHHHHHHHHHHHCCCCCCC
818	EQEERIQQDKRKNEWEDQYYQKYGQGKGHQP	-53.16	38.7	CHHHHHHHHHHHHHHHHHHHHHHHHCCCCCC
111	EQEERIQRDQYKNDYEDEEYQRKGRGKGHQP	-53.11	32.3	CHHHHHHHHHCCCHHHHHHHHHHHCCCCCCC
280	EQEQRIQQDKRSNEQEDKRYQREGKGKGHNN	-53.09	38.7	HHHHHHHHHHHHHHHHHHHHHHHHCCCCCCC
406	EQEERIQQDQRKNDKEDQRYQREGKGKGHNH	-53.04	32.3	CHHHHHHHHHHCHHHHHHHHHHHHCCCCCCC
297	EQEQRIQEDKRKNEEEDKRYQRYGDGKGHNP	-53.03	38.7	HHHHHHHHHHHHHHHHHHHHHHHHCCCCCCC
909	QDEKRIQEDKRKQEKEDEEYQRRGRGKGHQP	-52.90	35.5	CHHHHHHHHHHHHHHHHHHHHHHHCCCCCCC
794	ENEERIQQDQRKNDKEDKRYQREGKGKGHQP	-52.86	32.3	CHHHHHHHHHHCHHHHHHHHHHHHCCCCCCC
384	EQERRIQEDKERNEKEDEEYQRRGWGKGHYP	-52.80	38.7	HHHHHHHHHHHHHHHHHHHHHHHHCCCCCCC
643	EEEKKIQEDQRKNDKEDEEYQRRGRGKGHQP	-52.74	38.7	HHHHHHHHHHHHCCHHHHHHHHHHCCCCCCC
369	EQQQRIQEDQRRNDKEDRQYQQRGKGKGHNH	-52.61	35.5	HHHHHHHHHHHHHHHHHHHHHHHHHCCCCCC
659	EQEERIQRDKWRHEWEDKYYQKKGQGKGHQP	-52.58	41.9	CHHHHHHHHHHHHHHHHHHHHHHCCCCCCCC
546	EQEQRIQQDQYNNDWEDEEFQRKGRGKGKNN	-52.56	32.3	HHHHHHHHHHCCCCHHHHHHHHHHCCCCCCC
195	EQEQRIQEDKRRNEKEDKRYQREGKGKGHNH	-52.54	38.7	HHHHHHHHHHHHHHHHHHHHHHHHCCCCCCC
950	EQQQRIQQDQRNNEQEDQRYQREGRGKGHQP	-52.53	41.9	HHHHHHHHHHHHHHHHHHHHHHHHHCCCCCC
298	EQEERIQRDQQKNEREDKRYQRYGRGKGHQP	-52.46	35.5	HHHHHHHHHHHHHHHHHHHHHHHHHCCCCCC
394	EQEQRIQEDQRKNQKEHDRKQREGKGCGKNN	-52.45	22.6	HHHHHHHHHHHHHHHHHHHHHHHCCCCCCCC
017	EQQQRIQEDQRKNRKEDERYQREGRGKGHQP	-52.44	35.5	HHHHHHHHHHHHHHHHHHHHHHHHHCCCCCC
770	EQEQRIQQDQFSNDFEDKRYQQEGRGKGHQP	-52.43	35.5	HHHHHHHHHHHHHHHHHHHHHHHHHCCCCCC
235	EQEQRIQQDQYNNDYEDEEYQRKGRGKGHQP	-52.43	35.5	HHHHHHHHHHCCCCHHHHHHHHHHCCCCCCC
910	EQEERIQQDQRKNEKEDEEYQRRGRGKGHQP	-52.40	35.5	CHHHHHHHHHHHHHHHHHHHHHHHCCCCCCC
636	EEEKRIQEDQRKNDKEDEEYQRYGRGKGHQP	-52.38	35.5	HHHHHHHHHHHHCCHHHHHHHHHHHCCCCCC
377	QDQERINQDQQKNDKEDKRYQREGRGKGHQP	-52.38	32.3	CHHHHHHHHHHHHHHHHHHHHHHHCCCCCCC
382	EQEQRIQEDQRKHEKEDQRYQREGRGKGHQP	-52.36	38.7	HHHHHHHHHHHHHHHHHHHHHHHHHCCCCCC
639	EQEKRIQEDQRKNREEDERYQREGRGKGHQP	-52.31	32.3	HHHHHHHHHHHHHHHHHHHHHHHHHCCCCCC
648	EQEERIQQDQQKNRKEDEEYQRRGRGKGHQP	-52.30	32.3	CHHHHHHHHHHHHHHHHHHHHHHHCCCCCCC
050	EQEQRIQEDQRKNDKEDRRYQREGRGKGHQP	-52.29	32.3	HHHHHHHHHHHHCHHHHHHHHHHHCCCCCCC
361	EQEERIKQDKQNNEREDKRYQREGKGKGHNN	-52.29	41.9	CHHHHHHHHHHHHHHHHHHHHHHHCCCCCCC
658	EQEERIQRDQRKNRKEDEEYQKQGRGKGHQP	-52.28	32.3	CHHHHHHHHHHHHHHHHHHHHHHCCCCCCCC
519	EQEERIQQDQYKNEWEDEEYQRKGRGKGHQP	-52.27	35.5	CHHHHHHHHHHHHHHHHHHHHHHHCCCCCCC
831	EQEERIQRDQYKNEHEDERYQREGWGKGHYP	-52.27	35.5	CHHHHHHHHHHHHHHHHHHHHHHHCCCCCCC
483	EQEKRIQEDQRKNEEEDKRYQRYGQGKGHQS	-52.27	35.5	HHHHHHHHHHHHHHHHHHHHHHHHHCCCCCC
542	QEEEKIQRDQQRHEREDKRYQREGRGKGHQP	-52.26	41.9	CHHHHHHHHHHHHHHHHHHHHHHHCCCCCCC
669	EQEKRIQEDQRSHEEEDEYYQQYGQGKGHQP	-52.24	38.7	HHHHHHHHHHHHHHHHHHHHHHHHHCCCCCC
903	EQQQRIQQDQYSNEWEDEEYQRKGRGKGHQP	-52.23	38.7	HHHHHHHHHHCCCHHHHHHHHHHHCCCCCCC
411	EQEERIQQDQRKNEKEDEEYQRYGRGKGHQP	-52.21	35.5	CHHHHHHHHHHHHHHHHHHHHHHHHCCCCCC
868	EQEERIQQDQRKNEWEDKYYQKKGQGKGHQP	-52.21	35.5	CHHHHHHHHHHHHHHHHHHHHHHHCCCCCCC
992	EQEERIQQDQRKNEYEDEEYQRKGRGKGHTP	-52.21	35.5	CHHHHHHHHHHHHHHHHHHHHHHCCCCCCCC
771	EQEQRIQEDQRKNEKEDKRYQEQGRGKGHQP	-52.21	35.5	HHHHHHHHHHHHHHHHHHHHHHHHCCCCCCC
370	EQEERIQQDQRKNDNEDRRYQREGDGKGHQP	-52.10	32.3	CHHHHHHHHHHCHHHHHHHHHHHHCCCCCCC
901	EQEERIKQDKYSNEWEDKKYQREGRGKGHQP	-52.10	38.7	CHHHHHHHHCCCHHHHHHHHHHHHCCCCCCC
108	EQEKRIQEDQQKNEREDKRYQREGRGKGHQP	-52.09	35.5	HHHHHHHHHHHHHHHHHHHHHHHHCCCCCCC
821	KREEEIQQDQRRNEQEDQRYQKEGQGKGHQS	-52.02	32.3	CHHHHHHHHHHHHHHHHHHHHHHHCCCCCCC
962	EQEERIKEDQKRNDEEDKRYQRYGRGKGHQP	-51.98	32.3	HHHHHHHHHHHHHHHHHHHHHHHHHCCCCCC
217	QREQEIQQDQYNNEWEDERYQRQGRGKGHQP	-51.98	35.5	CHHHHHHHHHHCHHHHHHHHHHHHCCCCCCC
044	EQQERINQDQQRNEKEDERYQRQGRGKGHQP	-51.98	38.7	HHHHHHHHHHHHHHHHHHHHHHHHCCCCCCC
980	EEEKKIQEDQRKNDEEDRRYQREGRGKGHQP	-51.97	38.7	HHHHHHHHHHHHCHHHHHHHHHHHCCCCCCC
863	DQERRIQEDQWRNEYEDEEYQRQGRGKGHQP	-51.90	32.3	HHHHHHHHHHHHHHHHHHHHHHHHCCCCCCC
339	QDQKRIQEDQRKNREEDEYYQQKGQGKGHQP	-51.88	32.3	CHHHHHHHHHHHHHHHHHHHHHHHCCCCCCC
997	SDQERIQRDQYKNEYEDEEYQRKGRGKGHQP	-51.84	35.5	CHHHHHHHHHHHHHHHHHHHHHHHCCCCCCC
738	KREEEIKQDQYNNDYEDKEYQKEGRGKGHQP	-51.80	32.3	CHHHHHHHHHCCCCHHCHHHHHHHCCCCCCC
796	QDEERIQQDQRKNEYEDEEYQRKGRGKGHQP	-51.78	32.3	CHHHHHHHHHHHHHHHHHHHHHHHCCCCCCC
201	DEERKIQEDQRRNEKEDEEYQRYGRGKGHQP	-51.67	38.7	HHHHHHHHHHHHHHHHHHHHHHHHHCCCCCC
469	QDEERIKEDKYRNESEDQRYQRYGRGKGHQP	-51.67	35.5	CHHHHHHHHHCCCHHHHHHHHHHHCCCCCCC
827	QQEENIQRDQYKNEWEDERYQRQGRGKGHQP	-51.67	32.3	CHHHHHHHHHHHHHHHHHHHHHHHCCCCCCC

447	QDEKRIQEDQRKNEEEDQQYQKQGRGKGHQP	-51.65	32.3	CHHHHHHHHHHHHHHHHHHHHHHHCCCCCCC
522	EQQQRIQEDQRRNDKEHKYYQQKGQGQGHQP	-51.65	29.0	HHHHHHHHHHHHHHHHHHHHHHHHCCCCCCC
840	EQEQRIQQDQKNNEEEDERYQKQGRGKGHQP	-51.60	38.7	HHHHHHHHHHHHHHHHHHHHHHHCCCCCCCC
126	EQEERIKQDKMKNEKEDEEYQRRGQGKGHQS	-51.58	38.7	CHHHHHHHHHHHHHHHHHHHHHHHHCCCCCC
252	QDEERIKQDQESNDREDKRYQREGRGKGHQP	-51.55	29.0	CHHHHHHHHHHHHHHHHHHHHHHHCCCCCCC
055	QDQERIKKDQYDNDWEDERYQQQGRGKGHQP	-51.54	32.3	CHHHHHHHHCCCCHHHHHHHHHHHCCCCCCC
822	QRQQEIQEDAWRHRYEDEEFQRKGRGKGKQN	-51.50	32.3	HHHHHHHHHHHHHHHHHHHHHHHHCCCCCCC
092	EEEQRKQEDQRRQDKEDQRYQREGRGKGHQP	-51.44	35.5	HHHHHHHHHHHHHHHHHHHHHHHHCCCCCCC
899	EEEERKQQDKRKQEKEDEEYQRRGKGKGHNH	-51.43	41.9	HHHHHHHHHHHHHHHHHHHHHHHHCCCCCCC
536	EQEQRIQQDQKNNEEEDKRYQREGRGKGHQP	-51.42	38.7	HHHHHHHHHHHHHHHHHHHHHHHHCCCCCCC
250	KRQQEIQQDQYNNDYEHEEYQRKGRGQGHQP	-51.34	29.0	CHHHHHHHHHCCCHHHHHHHHHHHCCCCCCC
976	EQEERIQQDQRKNDKEHEEYQRRGWGQGHYP	-51.32	25.8	CHHHHHHHHHHHHHHHHHHHHHHCCCCCCCC
779	EQEERIQQDQRKNDKEHEYYQQYGQGQGHQP	-51.32	25.8	CHHHHHHHHHHHHHHHHHHHHHHHHCCCCCC
565	EQEERIQRDQRKNDQEHKRYQREGRGQGHQP	-51.32	25.8	CHHHHHHHHHHHHHHHHHHHHHHHCCCCCCC
854	EQQQRKQEDQRRNDKEHKRYQREGRGRGHQP	-51.31	29.0	HHHHHHHHHHHHHHHHHHHHHHHHCCCCCCC
227	EEEERKQQDKRQNEREDKYYQQKGQGKGHQP	-51.31	41.9	HHHHHHHHHHHHHHHHHHHHHHHHCCCCCCC
037	EEQQRKQEDKRRNEKEDEYYQRKGQGKGHQP	-51.26	45.2	HHHHHHHHHHHHHHHHHHHHHHHHCCCCCCC
591	EEEQRKQEDKRRNEKEDKYYQKYGQGKGHQP	-51.26	41.9	HHHHHHHHHHHHHHHHHHHHHHHHHCCCCCC
458	QEEQKKQEDQRRHDWEDSYYQQYGQGKGHQP	-51.24	38.7	HHHHHHHHHHHHHHHHHHHHHHHHHCCCCCC
266	EEEERKQRDQYKNDWEDEEYQRKGRGKGTNN	-51.21	35.5	HHHHHHHHHHHHHHHHHHHHHHHHCCCCCCC
077	EEEERKQRDKQKNEKEDKRYQREGRGKGHQP	-51.20	41.9	HHHHHHHHHHHHHHHHHHHHHHHHCCCCCCC
020	EQEERKQQDKQKNEREDKRYQREGKGKGHNH	-51.19	38.7	HHHHHHHHHHHHHHHHHHHHHHHCCCCCCCC
967	EQEERIKQDQYNNRYEDEEYQRQGQGKGHQS	-51.18	35.5	CHHHHHHHHHHCCCHHHHHHHHHHCCCCCCC
961	QREQEIQERQRRERKEDERYQREGQGKGHQS	-51.17	25.8	HHHHHHHHHHHHHHHHHHHHHHHHCCCCCCC
549	EQEERIKQDKYNNEYEDEEYQRKGRGKGNQN	-51.16	41.9	CHHHHHHHHHCCCCCHHHHHHHHHCCCCCCC
134	EQEERIKQDQEKNEKEDEEYQRRGRGKGHQP	-51.15	35.5	CHHHHHHHHHHHHHHHHHHHHHHHCCCCCCC
768	QQEENIKQDQKSNEEEDEYYQRKGQGKGHQP	-51.15	32.3	CHHHHHHHHHHHHHHHHHHHHHHHCCCCCCC
989	EQEERIQQDQRKNDKEHQRYQKQGRGQGHQP	-51.14	25.8	CHHHHHHHHHHHHHHHHHHHHHHHCCCCCCC
819	EQEERIKEDQYRNEWEDRRYQREGRGKGHQP	-51.14	35.5	HHHHHHHHHHHHHHHHHHHHHHHHCCCCCCC
764	EEQQRKQQDKYNNEWEDKKYQREGRGKGHNH	-51.13	48.4	HHHHHHHHHHHHHHHHHHHHHHHHCCCCCCC
883	EEEERKQRDKRKNEQEDKRYQREGKGKGHNH	-51.13	41.9	HHHHHHHHHHHHHHHHHHHHHHHCCCCCCCC
699	QEEEKKQQDKRKNENEDKRYQREGRGKGHQP	-51.13	41.9	CHHHHHHHHHHHHHHHHHHHHHHHCCCCCCC
086	QEEQKKQEDKRKNEKEDKRYQKQGWGKGHYP	-51.13	41.9	HHHHHHHHHHHHHHHHHHHHHHHCCCCCCCC
208	EEERRKQEDQERNDKEDKRYQREGRGKGHQP	-51.07	35.5	HHHHHHHHHHHHHHHHHHHHHHHHCCCCCCC
448	QEEQKKQYDQEKNDKEDEEYQRKGRGKGHQP	-51.07	35.5	HHHHHHHHHHHHHHHHHHHHHHHHCCCCCCC
142	EEERKKQEDQYRNDAEHKRYQREGKGRGHNH	-51.05	35.5	HHHHHHHHHHHHHHHHHHHHHHHCCCCCCCC
047	EEQQRKQEDQRRNDQEDKRYQRYGRGKGHQP	-51.02	38.7	HHHHHHHHHHHHHHHHHHHHHHHHCCCCCCC
313	EQQERKQQDQRRNDKEDEEYQRRGQGKGHNP	-51.02	35.5	HHHHHHHHHHHHHHHHHHHHHHHHCCCCCCC
485	QEQQKKQEDQRKNDKEDKRYQREGRGKGHQP	-51.02	38.7	HHHHHHHHHHHHHHHHHHHHHHHHCCCCCCC
757	EEEQRKQEDQRKNDREDKYYQREGKGKGHNH	-51.01	35.5	HHHHHHHHHHHHHHHHHHHHHHHCCCCCCCC
043	EEEEKKQRDKQKNEREDEYYQQYGQGKGHQP	-51.00	45.2	HHHHHHHHHHHHHHHHHHHHHHHHHCCCCCC
388	EEEQRKQEDKQKNEREDEEYQRRGKGKGHNN	-51.00	41.9	HHHHHHHHHHHHHHHHHHHHHHHCCCCCCCC
189	QRQQEINEDQRKNEKEDEEYQRRGRGKGHQP	-50.99	35.5	CHHHHCHHHHHHHHHHHHHHHHHHCCCCCCC
200	QQEEEIKQDQQNNEKEDEEYQRYGQGKGHQS	-50.97	35.5	CHHHHHHHHHHHHHHHHHHHHHHHHCCCCCC
982	DEERKKQEDKQRNEKEDKRYQREGKGKGHNH	-50.97	41.9	CHHHHHHHHHHHHHHHHHHHHHHCCCCCCCC
988	DDQRRIQEDQYRNDYEHEEYQRKGRGQGHQP	-50.96	25.8	CHHHHHHHHHHHHHHHHHHHHHHHCCCCCCC
707	DQERKKQEDKRRNEYEDEEYQRQGRGKGHQP	-50.94	38.7	HHHHHHHHHHHHHHHHHHHHHHHHCCCCCCC
709	EEEERKQQDKRKNEKEDEEYQRYGRGKGHQP	-50.94	41.9	HHHHHHHHHHHHHHHHHHHHHHHHHCCCCCC
914	EEEERKQQDKRKNENEDKRYQREGKGKGHNH	-50.94	41.9	HHHHHHHHHHHHHHHHHHHHHHHCCCCCCCC
759	EEEQRKQEDKRKNEKEDKRYQRYGQGKGHQS	-50.94	41.9	HHHHHHHHHHHHHHHHHHHHHHHHCCCCCCC
824	EEEQRKQEDKRKNENEDKSYQRHGQGKGHQS	-50.94	41.9	HHHHHHHHHHHHHHHHHHHHHHHCCCCCCCC
693	EQQERINQDQQKNDKEHEEYQRRGRGQGHQP	-50.91	29.0	HHHHHHHHHHHHHHHHHHHHHHHHHCCCCCC
629	QEEEKKNQDQQKNDKEDEEYQRYGQGKGHQS	-50.89	35.5	CHHHHHHHHHHHCHHHHHHHHHHHHCCCCCC
413	EEEKKKQEDQRKNDEEDEEYQRKGRGKGHQP	-50.89	38.7	HHHHHHHHHHHHHHHHHHHHHHHHCCCCCCC
972	EEEKRKQEDQRKNDEEDERYQKEGKGKGHNH	-50.89	35.5	HHHHHHHHHHHHHHHHHHHHHHHCCCCCCCC
265	EEEERKQRDQEKNDKEDEKYQREGRGKGHQP	-50.88	35.5	HHHHHHHHHHHHHHHHHHHHHHHHCCCCCCC
900	EEEQKKQEDKRRNEKEDEEYQRRGWGKGHYP	-50.87	45.2	HHHHHHHHHHHHHHHHHHHHHHHCCCCCCCC
479	QEEQKKQQDKRSNEFEDKYYQQKGQGKGHNH	-50.87	41.9	HHHHHHHHHHHHHHHHHHHHHHHHCCCCCCC
871	QQQQNIQEDQRRNRKEHEYYQKKGQGQGHQP	-50.84	25.8	HHHHHHHHHHHHHHHHHHHHHHHHCCCCCCC
434	EEEQRKNEDQRKNDKEDEEYQRRGRGKGHQP	-50.83	35.5	HHHHHHHHHHHHCHHHHHHHHHHHCCCCCCC
347	EQEERIKQDQWKNEWEDEYYQRKGQGKGHQP	-50.82	35.5	CHHHHHHHHHHHHHHHHHHHHHHHCCCCCCC
562	EEEEKKQQDQRKNDNEDKRYQREGQGKGHQS	-50.82	38.7	CHHHHHHHHHHHHHHHHHHHHHHHCCCCCCC
686	EEEERKQQDQRKNDKEDEEYQRYGKGKGHNH	-50.82	35.5	HHHHHHHHHHHHHHHHHHHHHHHHHCCCCCC
038	EEEERKQQDQRKNDKEDQRYQQQGRGKGHQP	-50.82	35.5	HHHHHHHHHHHHHHHHHHHHHHHHCCCCCCC
527	EEEERKQQDQRKNDNEDERYQQQGRGKGHQP	-50.82	35.5	HHHHHHHHHHHHHCHHHHHHHHHHCCCCCCC
570	EEEERKQQDQRKNDNEDKRYQREGRGKGHQP	-50.82	35.5	HHHHHHHHHHHHHHHHHHHHHHHHCCCCCCC
104	EEEERKQQDQRRNDQEDERYQKQGRGKGHQP	-50.82	35.5	HHHHHHHHHHHHHHHHHHHHHHHCCCCCCCC

254	EEEERKQQDQRRNDQEDKRYQREGRGKGHQP	-50.82	35.5	HHHHHHHHHHHHHHHHHHHHHHHHHCCCCCCC
778	QEEEKKQQDQRKNDWEDKYYQKKGQGKGHQP	-50.82	35.5	CHHHHHHHHHHHHHHHHHHHHHHHHCCCCCCC
315	QEEKKKQEDKRKNEEEDEQYQRYGRGKGHQP	-50.81	41.9	HHHHHHHHHHHHHHHHHHHHHHHHHHHCCCCC
416	NDQRRKQEDKERNEKEDKRYQREGRGKGHQP	-50.80	38.7	CHHHHHHHHHHHHHHHHHHHHHHHHCCCCCCC
109	EEEERKQQDQDKNDREHEEYQRYGRGRGHQP	-50.79	29.0	HHHHHHHHHHHHHHHHHHHHHHHHHHHCCCCC
019	EEEERKQQDQRKNDKEDKRYQREGRGKGHQP	-50.75	35.5	HHHHHHHHHHHHHHHHHHHHHHHHHCCCCCCC
893	EEEEKKQRDQWKNDWEHEYKQKYGQGCGKQN	-50.74	29.0	HHHHHHHHHHHHHHHHHHHHHHHHHHHCCCCC
554	QDEERKQQDKRRNEYEDKQYQKEGRGKGHQP	-50.73	35.5	CHHHHHHHHHHHHHHHHHHHHHHHHCCCCCCC
581	QDQERIQQDQRKNEYEDEEYQRKGRGKGDQR	-50.73	41.9	CHHHHHHHHHHHHHHHHHHHHHHHHCCCCCCC
960	QDEERKQRDKYKNEYEDEEFQRKGRGKGKNN	-50.72	32.3	CHHHHHHHHHHHHHHHHHHHHHHHHCCCCCCC
344	QQQQNINEDQRKNDKEHEEYQRRGWGQGHYP	-50.72	25.8	CHHHCHHHHHHHHHHHHHHHHHHHHCCCCCCC
859	EEEERKQQDKRKNEKEDEEYQRRGRGKGHQP	-50.70	41.9	HHHHHHHHHHHHHHHHHHHHHHHHHHCCCCCC
396	EEQQRKQQDQRSNDKEDREFQRRGQGKGKNN	-50.69	35.5	HHHHHHHHHHHHCHHHHHHHHHHHHCCCCCCC
739	DEERKKQEDQWRNDWEDEYYQRKGQGKGHQP	-50.69	35.5	HHHHHHHHHHHHHHHHHHHHHHHHHCCCCCCC
516	EEEERKQQDQRKNDYEHEEKQRKGRGCGKQN	-50.67	25.8	HHHHHHHHHHHHHHHHHHHHHHHHHCCCCCCC
015	EEEKRKQEDQRKNDKEDEEYQRRGKGKGHNN	-50.65	35.5	HHHHHHHHHHHHHHHHHHHHHHHHHCCCCCCC
550	EQEQRKQEDQRRNEKEDEEFQRRGKGKGKNN	-50.62	32.3	HHHHHHHHHHHHHHHHHHHHHHHHHCCCCCCC
736	NDQERIKQDQKKNEKEDEEYQRRGKGKGHQH	-50.59	35.5	CHHHHHHHHHHHHHHHHHHHHHHHHCCCCCCC
751	QREQEIQEQKRRNEKEDKRYQRYGRGKGHQP	-50.58	32.3	CHHHHHHHHHHHHHHHHHHHHHHHHCCCCCCC
168	EQQQRIQQDQDSNDREQEEYQRRGRGQGHQP	-50.58	29.0	HHHHHHHHHHHCHHHHHHHHHHHHHHCCCCCC
947	EQEQRIQEDQRKNDKEQKRYQREGRGQGHQP	-50.57	25.8	HHHHHHHHHHHHHHHHHHHHHHHHHHCCCCCC
462	EEEERKQQDQRRHDKEHEEKQRRGRGCGKQN	-50.56	29.0	HHHHHHHHHHHHHHHHHHHHHHHHHCCCCCCC
656	SDEERIKEDQKRNDEEDRRYQRYGRGKGHQP	-50.55	29.0	CHHHHHHHHHHHCHHHHHHHHHHHHCCCCCCC
318	QEEKKKQEDQRKNDEEHQRKQKQGKGCGKNN	-50.54	25.8	HHHHHHHHHHHHHHHHHHHHHHHHHCCCCCCC
944	EQQQRKNNDKYNNEWEDEEYQRKGRGKGHQP	-50.51	45.2	HHHHHHHCHHCCCHHHHHHHHHHHHCCCCCCC
844	SREEQIQREQQSNEREDKRYQREGRGKGHQP	-50.50	29.0	CHHHHHHHHHHHHHHHHHHHHHHHHHCCCCCC
497	EQQERKQQDKRKNEKEDEEYQRRGRGKGHQP	-50.50	41.9	HHHHHHHHHHHHHHHHHHHHHHHHHHCCCCCC
898	EEEQRKQQDQYNNDYEHEEKQRKGRGCGKQN	-50.48	29.0	HHHHHHHHHHHHHHHHHHHHHHHHHCCCCCCC
880	QRQKEIQEQQRSNDEEDEYYQQYGQGKGHQP	-50.46	29.0	HHHHHHHHHHHHCHHHHHHHHHHHHHCCCCCC
033	EEEERKQQDKRRHEYEDQEYQRQGRGKGHQP	-50.45	45.2	HHHHHHHHHHHHHHHHHHHHHHHHHHCCCCCC
177	DNERRIQEEQYRNDWEDERYQRQGKGKGHSN	-50.44	25.8	CHHHHHHHHHHHHHHHHHHHHHHCCCCCCCCC
867	EEEERKQQDKRRNEWEDKYYQKKGQGKGHQP	-50.42	41.9	HHHHHHHHHHHHHHHHHHHHHHHHHHCCCCCC
012	EEEQRKQQDQYNQDWEDERYQRQGWGKGHYP	-50.41	38.7	HHHHHHHHHHHHHHHHHHHHHHHHHHCCCCCC
572	EEEERKQRDKWKNEWEDKYYQKKGQGKGHQP	-50.39	41.9	HHHHHHHHHHHHHHHHHHHHHHHHHHCCCCCC
107	EEEQRKQEDKYRNEYEDKEYQRKGRGKGHQP	-50.39	41.9	HHHHHHHHHHHHHHHHHHHHHHHHHCCCCCCC
641	KRQEEIQRDQRKNDQEQQRYQREGKGQGHNH	-50.39	25.8	CHHHHHHHHHHHHHHHHHHHHHHHHHCCCCCC
123	QEQQKKQEDQRRNDKEDRRYQRYGKGKGHNN	-50.38	38.7	HHHHHHHHHHHHHHHHHHHHHHHHCCCCCCCC
968	EEEQRKQQDKYNNEWEDKKYQREGRGKGHQP	-50.33	45.2	HHHHHHHHHHHHHHHHHHHHHHHHHHCCCCCC
223	EEEQRKQQDKYNNEYEDEEYQRKGRGKGHQP	-50.33	45.2	HHHHHHHHHHHHHHHHHHHHHHHHHHCCCCCC
713	EQEQRIQEEQYRNEYEDEEYQRKGRGKGHQP	-50.32	32.3	HHHHHHHHHHHHHHHHHHHHHHHHHHCCCCCC
084	EEEERKQQDQQKNDREDRRYQRYGRGKGHQP	-50.31	35.5	HHHHHHHHHHHHHHHHHHHHHHHHHHCCCCCC
537	EEQERKQRDQEKNDKEDRRYQREGRGKGHQP	-50.31	38.7	HHHHHHHHHHHHHHHHHHHHHHHHHHCCCCCC
955	EEQQRKQQDQRNNEKEDEEYQRRGRGKGHQP	-50.31	45.2	HHHHHHHHHHHHHHHHHHHHHHHHHHCCCCCC
427	EEQQRKQQDQRSNEQEDKRYQKEGQGKGHQS	-50.31	41.9	HHHHHHHHHHHHHHHHHHHHHHHHHCCCCCCC
026	EQQQRKQEDQRRNEKEDEEYQRKGRGKGHQP	-50.31	38.7	HHHHHHHHHHHHHHHHHHHHHHHHHHCCCCCC
920	EEERRKQEDQERNDKEDRRYQREGKGKGHNN	-50.31	35.5	HHHHHHHHHHHHHHHHHHHHHHHHHCCCCCCC
586	ENEQRIQEDQRKNEKEHKRYQREGRGQGHQP	-50.31	29.0	CHHHHHHHHHHHHHHHHHHHHHHHHHCCCCCC
174	EQERRKQEDQWRNDYEDEEYQRKGRGKGHQP	-50.28	32.3	HHHHHHHHHHHHHHHHHHHHHHHHHHCCCCCC
428	EEQQRKQYDQESNRKEDERYQREGRGKGHQP	-50.28	38.7	HHHHHHHHHHHHHHHHHHHHHHHHHHCCCCCC
133	EQEERIKQDQKNNDEEHERYQQQGRGQGHQP	-50.26	29.0	CHHHHHHHHHHCHHHHHHHHHHHHHHCCCCCC
457	EEQQRKQEDQWRNDYEDREYQRKGRGKGHQP	-50.26	38.7	HHHHHHHHHHHHHHHHHHHHHHHHHHCCCCCC
352	EEQERKQQDQRKNDYEDREYQRKGRGKGHQP	-50.26	38.7	HHHHHHHHHHHHHHHHHHHHHHHHHHCCCCCC
373	EEEQRKQEDQRKNDKEDRRYQREGRGKGHQP	-50.25	35.5	HHHHHHHHHHHHHHHHHHHHHHHHHHCCCCCC
990	QREEQIQREQRSNEKEDQEYQKQGKGKGHNN	-50.25	29.0	CHHHHHHHHHHHHHHHHHHHHHHHHCCCCCCC
321	QREEQIQREQRSNEQEDKRYQKEGRGKGHQP	-50.25	29.0	CHHHHHHHHHHHHHHHHHHHHHHHHHCCCCCC
430	EEEERKQRDQRKQEKEDEEYQRYGRGKGHQP	-50.24	38.7	HHHHHHHHHHHHHHHHHHHHHHHHHHHCCCCC
657	EEEERKQQDQQKNEREDEEYQRRGRGKGHQP	-50.24	38.7	HHHHHHHHHHHHHHHHHHHHHHHHHHCCCCCC
853	EEEERKQRDQQKNEREDEEYQRRGRGKGHQP	-50.24	38.7	HHHHHHHHHHHHHHHHHHHHHHHHHHCCCCCC
552	EEEQKKQEDQQKNEKEDKRYQREGRGKGHQP	-50.24	41.9	HHHHHHHHHHHHHHHHHHHHHHHHHHCCCCCC
152	QQEKNKQEDQQKNEREDQRYQKEGQGKGHQS	-50.24	32.3	CHHHHHHHHHHHHHHHHHHHHHHHHCCCCCCC
594	EQQQRIQEDQRRNDKEQEEYQRKGRGQGHQP	-50.23	29.0	HHHHHHHHHHHHHHHHHHHHHHHHHHCCCCCC
452	EEEEKKRRDQEKNDKEDEYYQKYGQGKGHNP	-50.22	38.7	CHHHHHHHHHHHHHHHHHHHHHHHHHHCCCCC
106	QREEEIQQQRKQRNEDERFQREGQGKGKQS	-50.22	22.6	CHHHHHHHHHHHHHHHHHHHHHHHHHCCCCCC
207	EEEQRKQEDQRRNRKEDEEYQRSGRGKGHQP	-50.22	38.7	HHHHHHHHHHHHHHHHHHHHHHHHHCCCCCCC
039	EEEQKKQQDQDNNDREDEEYQRYGRGKGHQP	-50.21	41.9	HHHHHHHHHHCCCCHHHHHHHHHHHHCCCCCC
937	EQEERKNQDQQKNEKEDERYQRQGQGKGHQS	-50.19	35.5	HHHHHHHHHHHHHHHHHHHHHHHHHHCCCCCC
309	EEEEKKQQDKRRNEFEDKYYQQKGQGKGNQN	-50.19	45.2	HHHHHHHHHHHHHHHHHHHHHHHHHHCCCCCC

611	EEQQRKQEDQKRNEEEDERYQRYGRGKGHQP	-50.18	41.9	HHHHHHHHHHHHHHHHHHHHHHHHHCCCCCC
984	EEQQRKQEDQWRNEWEDEYYQKYGQGKGHQP	-50.18	41.9	HHHHHHHHHHHHHHHHHHHHHHHHHCCCCCC
559	EEQQRKQEDQWRNEWEDQKYQREGRGKGHQP	-50.18	41.9	HHHHHHHHHHHHHHHHHHHHHHHHCCCCCCC
282	EEQQRKQQDQYNNEWEDEEYQRKGRGKGHQP	-50.18	45.2	HHHHHHHHHHHHHHHHHHHHHHHHCCCCCCC
590	EEEQRKQQDQQSNEEEDERYQKQGRGKGHQP	-50.18	38.7	HHHHHHHHHHHHHHHHHHHHHHHCCCCCCCC
877	EEEERKQQDQRKNEQEDKRYQREGRGKGHQP	-50.18	38.7	HHHHHHHHHHHHHHHHHHHHHHHHCCCCCCC
971	EEEERKQQDQRKNEYEDEEYQRKGRGKGHQP	-50.18	38.7	HHHHHHHHHHHHHHHHHHHHHHHHCCCCCCC
599	EEEQRKQEDQRKNEKEDEKYQKEGRGKGHQP	-50.18	38.7	HHHHHHHHHHHHHHHHHHHHHHHHCCCCCCC
381	EEEQRKQEDQRKNEKEDKRYQREGRGKGHQP	-50.18	38.7	HHHHHHHHHHHHHHHHHHHHHHHHCCCCCCC
122	EEEQRKQEDQRRNEKEDEEYQRYGRGKGHQP	-50.18	38.7	HHHHHHHHHHHHHHHHHHHHHHHHHCCCCCC
681	EQEERKQRDQRKNEKEDEEYQRRGRGKGHQP	-50.18	35.5	HHHHHHHHHHHHHHHHHHHHHHHHCCCCCCC
332	QEEEKKQQDQRKNEKEDEEYQRYGWGKGHYP	-50.18	38.7	CHHHHHHHHHHHHHHHHHHHHHHCCCCCCCC
858	EQEKRKQEDQRKNEEEDEEFQRRGKGKGKNN	-50.17	32.3	HHHHHHHHHHHHHHHHHHHHHHHCCCCCCCC
953	EQEKRKQEDQRKNEEEDERFQQQGRGKGKQN	-50.17	32.3	HHHHHHHHHHHHHHHHHHHHHHHHCCCCCCC
468	QDQERIQQDQWKNEWEHKFYQKEGRGQGHQP	-50.12	32.3	CHHHHHHHHHHHHHHHHHHHHHHHCCCCCCC
647	EEEERKQQDQKKNDEEDRRYQRYGWGKGHYP	-50.12	35.5	HHHHHHHHHHHHHHHHHHHHHHHCCCCCCCC
132	EEEERKQQDQYRNDWEDRRYQREGRGKGHQP	-50.12	35.5	HHHHHHHHHHHHHHHHHHHHHHHHCCCCCCC
727	EEEKRKQEDQRKNDEEDRRYQREGRGKGHQP	-50.12	35.5	HHHHHHHHHHHHHHHHHHHHHHHHCCCCCCC
215	EEERRKQEDQWRNDWEDRYYQQQGQGKGHQP	-50.12	35.5	HHHHHHHHHHHHHHHHHHHHHHHHCCCCCCC
872	EEEEKKQRDQDKNRREDERYQREGKGKGHNH	-50.12	38.7	HHHHHHHHHHHHHHHHHHHHHHHCCCCCCCC
506	EEERRKQEDQYRNRYEDEEYQRKGRGKGHQP	-50.09	35.5	HHHHHHHHHHHHHHHHHHHHHHHHCCCCCCC
621	EQERRKQEDQKRNREEDERYQRYGRGKGHQP	-50.09	32.3	HHHHHHHHHHHHHHHHHHHHHHHHHCCCCCC
075	EQERRKQEDQYRNRYEDEEYQRKGRGKGHQP	-50.09	32.3	HHHHHHHHHHHHHHHHHHHHHHHHCCCCCCC
789	EEQERKRQDQEKNDKEDEEYQRYGQGKGHQS	-50.08	38.7	HHHHHHHHHHHHHCHHHHHHHHHHHCCCCCC
663	EQEERKQRDQQKNRREDEEYQRRGRGKGHQP	-50.08	32.3	HHHHHHHHHHHHHHHHHHHHHHHHCCCCCCC
410	EEEERKKQDQYNNDYEDEEFQRKGRGKGKQN	-50.07	35.5	HHHHHHHHHHHCCCHHHHHHHHHHCCCCCCC
085	DDQRRIQEDQYRNQWEHDRYQREGRGQGHQP	-50.06	25.8	CHHHHHHHHHHHHHHHHHHHHHHCCCCCCCC
939	EEEERKQQDQRKNDKEDRRYQREGKGKGHNH	-50.06	35.5	HHHHHHHHHHHHHHHHHHHHHHHCCCCCCCC
587	EQEERKQQDQRRNDKEDREYQRRGQGKGHNP	-50.06	32.3	HHHHHHHHHHHHHHHHHHHHHHHHCCCCCCC
316	QEEQKKQEDQRKNDKEDRRYQRYGKGKGHNN	-50.06	35.5	HHHHHHHHHHHHHHHHHHHHHHCCCCCCCCC
510	QEEEKKQQDQRKNRWEDEYYQKKGQGKGHQP	-50.06	35.5	CHHHHHHHHHHHHHHHHHHHHHHHCCCCCCC
583	EEEERKQRDQQKNEKEDKRYQREGRGKGHNP	-50.06	38.7	HHHHHHHHHHHHHHHHHHHHHHHCCCCCCCC
626	DEERKKQEDQYRNEWEDEEYQRKGRGKGHNP	-50.05	38.7	HHHHHHHHHHHHHHHHHHHHHHHCCCCCCCC
633	EEEKRKQEDQRKNEEEDEEYQRKGRGKGHQP	-50.05	38.7	HHHHHHHHHHHHHHHHHHHHHHHHCCCCCCC
525	EEEKRKQEDQRKNEEEDEQYQRYGWGKGHYP	-50.05	38.7	HHHHHHHHHHHHHHHHHHHHHHHCCCCCCCC
803	EEEKRKQEDQRKNEEEDERYQKQGRGKGHQP	-50.05	38.7	HHHHHHHHHHHHHHHHHHHHHHHCCCCCCCC
651	EEEKRKQEDQRKNEKEDKEYQKQGKGKGHQH	-50.05	38.7	HHHHHHHHHHHHHHHHHHHHHHHHCCCCCCC
916	EEEKRKQEDQRKNEKEDKEYQKQGWGKGHYP	-50.05	38.7	HHHHHHHHHHHHHHHHHHHHHHHHCCCCCCC
048	EEERRKQEDQYRNEWEDERYQQQGRGKGHQP	-50.05	38.7	HHHHHHHHHHHHHHHHHHHHHHHHCCCCCCC
523	EQEKRKQEDQRKNEEEDERYQQQGRGKGHQP	-50.05	35.5	HHHHHHHHHHHHHHHHHHHHHHHHCCCCCCC
838	QEEENKQRDQYKNEWEDEEYQRKGRGKGHQP	-50.05	35.5	CHHHHHHHHHHHHHHHHHHHHHHHCCCCCCC
933	QQEKEKQEDQRKNEEEDERYQKQGRGKGHQP	-50.05	32.3	CHHHHHHHHHHHHHHHHHHHHHHCCCCCCCC
744	EQEQRKNEDQQKNEREDKRYQRYGWGKGHYP	-50.05	35.5	HHHHHHHHHHHHHHHHHHHHHHHCCCCCCCC
804	EEEERKQRDQWKNDWEDQYYQKKGQGKGHNH	-50.04	35.5	HHHHHHHHHHHHHHHHHHHHHHHHCCCCCCC
262	EEEERKNQDQYKNRWEDERYQREGRGKGHQP	-50.03	35.5	HHHHHHHHHHHHHHHHHHHHHHHHCCCCCCC
783	EEEQKKQEDQRKNEYEDEEYQRKGRGKGHNP	-50.03	41.9	HHHHHHHHHHHHHHHHHHHHHHHCCCCCCCC
685	EEEERKQQDQRKNRKEDEEYQRRGRGKGHQP	-50.02	35.5	HHHHHHHHHHHHHHHHHHHHHHHHCCCCCCC
807	EEEERKQQDQRKNRWEDEYYQKYGQGKGHQP	-50.02	35.5	HHHHHHHHHHHHHHHHHHHHHHHHHCCCCCC
151	QEEEKKQQDQWKHEWEDQYYQKYGQGKGHQP	-50.01	41.9	CHHHHHHHHHHHHHHHHHHHHHHHHCCCCCC
153	EEEERKKEDKYRNEYEDEEYQRKGRGKGTQN	-50.00	41.9	HHHHHHHHHHHHHHHHHHHHHHHHCCCCCCC
205	EEEQRKNEDQRKNEKEDKEYQKQGQGKGHQS	-50.00	38.7	HHHHHHHHHHHHHHHHHHHHHHHHCCCCCCC
211	EEQEKKKRDQYKNDWEDERYQKQGRGKGHQP	-50.00	41.9	HHHHHHHHHHHHHHHHHHHHHHHCCCCCCCC
688	EEEQRKQEDQRRNDKEDRRYQRYGRGKGHQP	-49.99	35.5	HHHHHHHHHHHHHHHHHHHHHHHHCCCCCCC
547	EEEQRKQQDQRNNRQEDERYQQYGQGKGHQS	-49.99	38.7	HHHHHHHHHHHHHHHHHHHHHHHHHCCCCCC
892	DEERKKQEDQRRNEQEDKRYQREGKGKGHSH	-49.99	38.7	CHHHHHHHHHHHHHHHHHHHHHHCCCCCCCC
421	EEEERKQQDQRKNEKEDEEYQRYGQGKGHQS	-49.99	38.7	HHHHHHHHHHHHHHHHHHHHHHHHHCCCCCC
049	EEEERKQQDQRKNEKEDERYQQQGRGKGHQP	-49.99	38.7	HHHHHHHHHHHHHHHHHHHHHHHHCCCCCCC
726	EEEERKQQDQRKNEKEDKRYQREGKGKGHNH	-49.99	38.7	HHHHHHHHHHHHHHHHHHHHHHHCCCCCCCC
330	EEEERKQRDQRKNEEEDKKYQRYGWGKGHYP	-49.99	38.7	HHHHHHHHHHHHHHHHHHHHHHHCCCCCCCC
879	EQEERKQQDQRKNEHEDQRYQREGRGKGHQP	-49.99	35.5	HHHHHHHHHHHHHHHHHHHHHHHHCCCCCCC
919	EQEQRKQEDQRKNEKEDERYQKQGWGKGHYP	-49.99	35.5	HHHHHHHHHHHHHHHHHHHHHHHCCCCCCCC
487	QEEQKKQEDQRKNEYEDEEYQRKGRGKGHQP	-49.99	38.7	HHHHHHHHHHHHHHHHHHHHHHHHCCCCCCC
983	EEEQRKQQDQQNNEREDERYQRQGRGKGHQP	-49.98	41.9	HHHHHHHHHHHHHHHHHHHHHHHCCCCCCCC
592	EEEQRKQEDQRKNRKEDERYQREGRGKGHQP	-49.98	35.5	HHHHHHHHHHHHHHHHHHHHHHHHCCCCCCC
847	EEQEKINRDQKKNDEEHRRYQKEGQGQGHQS	-49.96	35.5	CHHHHHHHHHHCHHHHHHHHHHHHCCCCCCC
120	EEEQRKQEDQRRNRKEDERYQREGRGKGHQP	-49.95	35.5	HHHHHHHHHHHHHHHHHHHHHHHHCCCCCCC
190	EQEQRKQQDQRSNRQEDERYQRYGRGKGHQP	-49.95	32.3	HHHHHHHHHHHHHHHHHHHHHHHHHCCCCCC

340	EEEERKKQDQEKNDKEDEEYQRKGRGKGHNP	-49.95	35.5	HHHHHHHHHHHHHHHHHHHHHHHCCCCCCCC
635	EEEERKKQDQEKNDKEDKRYQREGKGKGHNN	-49.95	35.5	HHHHHHHHHHHHHHHHHHHHHHHCCCCCCCC
414	EEEERKQRDQWKNEWEHEYKQKYGQGCGKQN	-49.93	29.0	HHHHHHHHHHHHHHHHHHHHHHHHHCCCCCC
343	EEEQKKQEDQRRNEKEDKRYQREGRGKGHQP	-49.92	41.9	HHHHHHHHHHHHHHHHHHHHHHHHCCCCCCC
000	EEEQKKQRDQRKNEKEDKEYQKQGRGKGHQP	-49.92	41.9	HHHHHHHHHHHHHHHHHHHHHHHHCCCCCCC
024	EEEQKKQRDQRKNEQEDERYQRQGRGKGHQP	-49.92	41.9	HHHHHHHHHHHHHHHHHHHHHHHHCCCCCCC
365	EEEQRKQEDQRRNEKEDEYYQKYGQGKGHQP	-49.92	38.7	HHHHHHHHHHHHHHHHHHHHHHHHHCCCCCC
443	QDQQRKQEDQRRNEKEDEEYQRQGNGKGHQP	-49.92	35.5	CHHHHHHHHHHHHHHHHHHHHHHCCCCCCCC
166	QDQQRKQEDQRRNEKEDKRYQREGRGKGHQP	-49.92	35.5	CHHHHHHHHHHHHHHHHHHHHHHHCCCCCCC
913	QRQEEIQRQQRSNDKEDREYQRRGQGKGHQP	-49.92	29.0	CHHHHHHHHHHHCCHHHHHHHHHHCCCCCCC
963	REEERIQQDQRSQDKQDRRYQRYGRGKGHQP	-49.91	29.0	CHHHHHHHHHHHHHHHHHHHHHHHCCCCCCC
936	QKQQEIQEQQKRNEEEDKRYQRYGRGKGHQP	-49.90	32.3	CHHHHHHHHHHHHHHHHHHHHHHHCCCCCCC
042	EEEERKQRDQYKNRWEDERYQREGDGKGHQP	-49.89	35.5	HHHHHHHHHHHHHHHHHHHHHHHCCCCCCCC
644	EEEEKKKRDKYKNEYEDKEYQRKGRGKGHQP	-49.88	45.2	CHHHHHHHHHHHHHHHHHHHHHHCCCCCCCC
491	EEEERKKEDKYRNEWEDKRYQREGRGKGHQP	-49.88	41.9	HHHHHHHHHHHHHHHHHHHHHHHHCCCCCCC
717	EEEERKKQDKYNNEWEDEEYQRKGRGKGHQP	-49.88	45.2	HHHHHHHHHHHHHHHHHHHHHHHHCCCCCCC
627	EQEERKKQDKQSNEKEDEEYQRRGRGKGHQP	-49.88	38.7	HHHHHHHHHHHHHHHHHHHHHHHHCCCCCCC
811	DEERKKQEDQWRNEWEDEYYQKYGQGKGHNP	-49.86	38.7	HHHHHHHHHHHHHHHHHHHHHHHHHCCCCCC
385	EEEKRKQEDQRKNEEEDKRYQRYGRGKGHQP	-49.86	38.7	HHHHHHHHHHHHHHHHHHHHHHHHCCCCCCC
057	EEEQRKQEDQRKNEEEDKYYQQKGQGKGHQP	-49.86	38.7	HHHHHHHHHHHHHHHHHHHHHHHHCCCCCCC
735	QEEEKKQRDQYKNEWEDEEYQRKGRGKGHQP	-49.86	38.7	CHHHHHHHHHHHHHHHHHHHHHHHCCCCCCC
476	QEEKKKQEDQRKNEKEDEEYQRRGKGKGHQH	-49.86	38.7	HHHHHHHHHHHHHHHHHHHHHHHHCCCCCCC
420	QEEKKKQEDQRKNEKEDEEYQRRGQGKGHQS	-49.86	38.7	HHHHHHHHHHHHHHHHHHHHHHHHHCCCCCC
322	EEEQRKNEDQKRNREEHERKQKQGKGCGKNN	-49.82	25.8	HHHHHHHHHHHHHHHHHHHHHHHCCCCCCCC
131	EEEERKKQDQRKNDEEDQRYQKQGKGKGHNN	-49.81	35.5	HHHHHHHHHHHHHHHHHHHHHHHCCCCCCCC
673	EQEERIQRDQQKNEREQKRYQKEGRGQGHQP	-49.80	29.0	HHHHHHHHHHHHHHHHHHHHHHHHCCCCCCC
080	QREKEIQEQQRKNDEEDRRYQQQGKGKGHSN	-49.78	25.8	CHHHHHHHHHHHHHHHHHHHHHHCCCCCCCC
016	EEEEKKKRDQYKNDYEDEEYQRQGRGKGHQP	-49.76	38.7	CHHHHHHHHHHHHHHHHHHHHHHHCCCCCCC
857	EEEEKKKRDQYNNDAEDKRYQREGRGKGHQP	-49.76	45.2	CHHHHHHHHHHCHHHHHHHHHHHHCCCCCCC
921	EEEERKKQDQKNNDEEDERYQQQGWGKGHYP	-49.76	38.7	HHHHHHHHHHHHHHHHHHHHHHHHCCCCCCC
946	EEEERKKQDQQNNDKEDEEYQRRGRGKGHQP	-49.76	38.7	HHHHHHHHHHHCCCHHHHHHHHHHCCCCCCC
124	EEEERKKQDQYNNDWEDEEYQRKGRGKGHQP	-49.76	38.7	HHHHHHHHHHHCCCHHHHHHHHHHCCCCCCC
593	EQEERKKEDQYRNDYEDEEYQRKGRGKGHQP	-49.76	32.3	HHHHHHHHHHHHHHHHHHHHHHHHCCCCCCC
773	EEEERKQQDQRKNEKEDQRYQREGKGKGHNH	-49.75	38.7	HHHHHHHHHHHHHHHHHHHHHHHHCCCCCCC
130	EEEERKKQDQEKNDKEDERYQKQGKGKGHNH	-49.75	35.5	HHHHHHHHHHHHHHHHHHHHHHHCCCCCCCC
357	EEEERKKQDQEKNDKEDKRYQKEGKGKGHNH	-49.75	35.5	HHHHHHHHHHHHHHHHHHHHHHHCCCCCCCC
897	EEEQRKQEDQKRNREEDERYQKQGRGKGHQP	-49.73	35.5	HHHHHHHHHHHHHHHHHHHHHHHCCCCCCCC
926	EEQERKNQDQWKHEWEDEYYQKYGQGKGHQP	-49.71	45.2	HHHHHHHHHHHHHHHHHHHHHHHHHCCCCCC
125	EEQQRKQEDQYRNRYEDEEYQRKGRGKGHQP	-49.70	38.7	HHHHHHHHHHHHHHHHHHHHHHHHCCCCCCC
912	QDQERKKQDKYKNEWEHEEYQRKGRGRGHNP	-49.69	32.3	CHHHHHHHHHHHHHHHHHHHHHHCCCCCCCC
029	EQEKRIQEDQRKNDEEQRRYQNEGKGQGHNH	-49.68	25.8	HHHHHHHHHHHHHHHHHHHHHHHCCCCCCCC
059	EEEEKKKRDKYKNEWEDKRYQREGRGKGHQP	-49.68	45.2	CHHHHHHHHHHHHHHHHHHHHHHHCCCCCCC
179	QEEQKKQEDQRKNEKEDKRYQRYGRGKGHQP	-49.66	38.7	HHHHHHHHHHHHHHHHHHHHHHHHCCCCCCC
113	SDQERKQRDQKKNEEEDQRYQRYGWGKGHYP	-49.66	35.5	CHHHHHHHHHHHHHHHHHHHHHHCCCCCCCC
425	EEEERKKQDKYNNEWEHKRKQREGQGCGKQS	-49.66	35.5	HHHHHHHHHHHHHHHHHHHHHHHCCCCCCCC
729	EREKRYQEDQRKNDEEDKRYQRYGWGKGHYP	-49.66	32.3	CHHHHHHHHHHHHHHHHHHHHHHCCCCCCCC
878	EEEEKKKRDKKKNEEEDKRYQKEGRGKGHQP	-49.64	45.2	CHHHHHHHHHHHHHHHHHHHHHHHCCCCCCC
455	EQERRKQEDQQRNEREDKRYQREGQGKGHQS	-49.63	35.5	HHHHHHHHHHHHHHHHHHHHHHHCCCCCCCC
240	QQQQNIQEQQRRHEKEDERYQRQGWGKGHYP	-49.63	35.5	HHHHHHHHHHHHHHHHHHHHHHHHCCCCCCC
520	EEEERKQRDQWKNEWEDQYYQKKGQGKGHNH	-49.62	38.7	HHHHHHHHHHHHHHHHHHHHHHHHCCCCCCC
247	EEEEKKQRDKYKNEWEDRRYQREGKGKGHNN	-49.61	45.2	CHHHHHHHHHHHHHHHHHHHHHCCCCCCCCC
503	EEQQKKQRDQKKNEEEDKRYQRYGRGKGHQP	-49.59	45.2	HHHHHHHHHHHHHHHHHHHHHHHHCCCCCCC
139	EEEQRKQKDQESNEREDEYYQKYGQGKGHQP	-49.56	38.7	HHHHHHHHHHHHHHHHHHHHHHHHHCCCCCC
987	QREEEIQRDQRKNEKEQEEYQRRGRGQGHQP	-49.56	25.8	CHHHHHHHHHHHHHHHHHHHHHHHHCCCCCC
172	EQQQRKQEEKRRNEKEDKQYQERGRGKGHQP	-49.55	38.7	HHHHHHHHHHHHHHHHHHHHHHHHCCCCCCC
051	EQQQRKQEEKRRNEKEDKRYQREGRGKGHQP	-49.55	38.7	HHHHHHHHHHHHHHHHHHHHHHHHCCCCCCC
535	EEQQRKQQDKRNNENEHKRYQRYGRGQGHQP	-49.54	41.9	HHHHHHHHHHHHHHHHHHHHHHHHCCCCCCC
219	EEEKRKQEDQRKNEEEDKRYQKEGRGKGHQP	-49.53	38.7	HHHHHHHHHHHHHHHHHHHHHHHHCCCCCCC
069	EEEEKKQQDQRKNEKEDEEYQRRGQGKGHNP	-49.53	41.9	CHHHHHHHHHHHHHHHHHHHHHHHCCCCCCC
683	EEERRKQEDQYRNDSEDRRYQRYGRGKGHQP	-49.52	35.5	HHHHHHHHHHHHHHHHHHHHHHHHCCCCCCC
198	EEEERKQRDQRRHDQEHKRYQREGRGQGHQP	-49.52	32.3	HHHHHHHHHHHHHHHHHHHHHHHHCCCCCCC
001	QEEQKKQEDQRRHDKEHNRYQREGRGQGHQP	-49.52	32.3	HHHHHHHHHHHHHHHHHHHHHHHCCCCCCCC
400	EEEKRKQEDQRKNREEDEYYQKYGQGKGHQP	-49.52	35.5	HHHHHHHHHHHHHHHHHHHHHHHHHCCCCCC
745	EEEERKKQDQQKNEREDKRYQKEGRGKGHQP	-49.49	38.7	HHHHHHHHHHHHHHHHHHHHHHHCCCCCCCC
668	ENEQRKQQEKRSNEEEDKEYQRKGRGKGTQN	-49.48	35.5	CHHHHHHHHHHHHHHHHHHHHHHHCCCCCCC
825	EREERYQQDKRKNEWEDKYYQQQGQGKGHQS	-49.47	38.7	CHHHHHHHHHHHHHHHHHHHHHHHCCCCCCC
538	EEEERKQQDQRKNEKEDKEYQKRGDGKGHQP	-49.47	38.7	HHHHHHHHHHHHHHHHHHHHHHCCCCCCCCC

348	EEEERKQRDQYKNEYEDEEYQRKGRGKGHQP	-49.46	38.7	HHHHHHHHHHHHHHHHHHHHHHHHCCCCCCC
149	EEEQRKQQDQESNKREDEEYQRRGRGKGHQP	-49.45	35.5	HHHHHHHHHHHHHHHHHHHHHHHHCCCCCCC
787	EQQQRKQEDKRKNEEEDKYYQQKGQGKGNQN	-49.43	41.9	HHHHHHHHHHHHHHHHHHHHHHHCCCCCCCC
839	QQEKNIQEDQRKNEEEQERYQKQGRGQGHQP	-49.43	25.8	CHHHHHHHHHHHHHHHHHHHHHHHCCCCCCC
115	EEQQKKQRDQRKNDKEHQRYQRYGKGQGHNN	-49.43	35.5	HHHHHHHHHHHHHHHHHHHHHHHCCCCCCCC
624	DEERKKQEDQRRNEKEDEEYQRKGRGKGHQP	-49.42	38.7	CHHHHHHHHHHHHHHHHHHHHHHHCCCCCCC
614	EEEERKQQDKRKNEKEHKRYQREGRGQGHQP	-49.41	35.5	HHHHHHHHHHHHHHHHHHHHHHHHCCCCCCC
305	QEEQKKQEDKRKNEKEHKRYQREGKGQGHQP	-49.41	35.5	HHHHHHHHHHHHHHHHHHHHHHHCCCCCCCC
232	EQEQRKQEDQRRNEKEDKRYQREGRGKGHQP	-49.40	35.5	HHHHHHHHHHHHHHHHHHHHHHHHCCCCCCC
031	EQQQRKNEDQRKNDEEDRRYQNQGRGKGHQP	-49.40	35.5	HHHHHHHHHHHHHHHHHHHHHHCCCCCCCCC
454	EQEERIQQDDRKNRKEDEEYQRRGRGKGHQP	-49.39	32.3	CHHHHHHHHHHHHHHHHHHHHHHHCCCCCCC
470	EEEQRKQEDQKRNEEEDERYQRYGWGKGHYP	-49.38	38.7	HHHHHHHHHHHHHHHHHHHHHHHCCCCCCCC
364	EQEQRKQQDQKNNEEEDERYQQQGRGKGHQP	-49.38	38.7	HHHHHHHHHHHHHHHHHHHHHHHHCCCCCCC
073	EQEQRKQQDQYNNEWEDEEYQRKGRGKGHNP	-49.38	38.7	HHHHHHHHHHHHHHHHHHHHHHHCCCCCCCC
249	QQEENIQRDQRKNEKEQEEYQRRGRGQGHQP	-49.37	25.8	CHHHHHHHHHHHHHHHHHHHHHHHHCCCCCC
981	EEEERKQRDQQKNDREHKRYQREGRGQGHQP	-49.36	29.0	HHHHHHHHHHHHHHHHHHHHHHHHCCCCCCC
743	EEEKRKQEDQRKNEEEDERYQRQGKGKGTNR	-49.35	41.9	HHHHHHHHHHHHHHHHHHHHHHHCCCCCCCC
935	EEEEKKQRDQWKNEWEDEYYQRKGQGKGHQP	-49.34	41.9	HHHHHHHHHHHHHHHHHHHHHHHHCCCCCCC
209	EEEERKQQDKRKNEKEDRRFQRYGRGKGKQN	-49.33	38.7	HHHHHHHHHHHHHHHHHHHHHHHCCCCCCCC
366	EQQQRKQEDQWRNDYEHEEYQRKGRGQGHQP	-49.30	29.0	HHHHHHHHHHHHHHHHHHHHHHHHCCCCCCC
951	EEQQRKQEDQRKNDEEHKRYQRYGRGQGHQP	-49.30	32.3	HHHHHHHHHHHHHHHHHHHHHHHHCCCCCCC
437	EEEERKKQDQYKNEYEDEEYQRKGRGKGHQP	-49.30	38.7	HHHHHHHHHHHHHHHHHHHHHHHHCCCCCCC
985	EQEERKKRDQWKNEWEDKYYQKQGQGKGHQP	-49.30	35.5	HHHHHHHHHHHHHHHHHHHHHHHHCCCCCCC
790	EEEEKKQQDQRKNDNEHERYQQQGRGQGHQP	-49.29	32.3	CHHHHHHHHHHHHHHHHHHHHHHHHCCCCCC
096	EEEERKQQDQRKNDYEHEEYQRQGRGQGHQP	-49.29	29.0	HHHHHHHHHHHHHHHHHHHHHHHHCCCCCCC
870	EEEERKQRDQRKNDQEHKRYQRYGWGQGHYP	-49.29	29.0	HHHHHHHHHHHHHHHHHHHHHHHCCCCCCCC
224	EEEQKKQEDQRKNDKEHKRYQREGKGQGHNH	-49.29	32.3	HHHHHHHHHHHHHHHHHHHHHHHHCCCCCCC
389	EEEQRKQEDQRKNDKEHKRYQREGRGQGHQP	-49.29	29.0	HHHHHHHHHHHHHHHHHHHHHHHHCCCCCCC
814	EEQQRKQEDQKRNEEEDKRYQREGDGKGNQN	-49.25	41.9	HHHHHHHHHHHHHHHHHHHHHHHCCCCCCCC
776	EEEEKKKQDQKNNEEEDERFQKQGRGKGKQN	-49.23	41.9	HHHHHHHHHHHHHHHHHHHHHHHCCCCCCCC
074	EEERRKQEDAYRNRYEDEEYQRKGRGKGTQN	-49.22	35.5	HHHHHHHHHHHHHHHHHHHHHHHHCCCCCCC
993	EQEKRIQEDQRKNDEEQRRYQRYGRGQGHQP	-49.21	25.8	HHHHHHHHHHHHHHHHHHHHHHHHHCCCCCC
101	KQQQEKQEDQRRNDKEHEEYQRRGKGQGHNH	-49.20	25.8	CHHHHHHHHHHHHHHHHHHHHHHHCCCCCCC
682	EEEERKQRQKYSEEWQHKRYQREGRGQGHQP	-49.19	29.0	CHHHHHHHHHHHHHHHHHHHHHHHCCCCCCC
884	DEERKKQEDQYRNDWEHEEYQRKGRGQGHQP	-49.17	29.0	HHHHHHHHHHHHHHHHHHHHHHHHCCCCCCC
750	EEEKRKQEDQRKNDEEHKRYQREGWGQGHYP	-49.17	29.0	HHHHHHHHHHHHHHHHHHHHHHHHCCCCCCC
351	EQEERKQQDQKKNDEEHNRYQRYGQGQGHQS	-49.17	25.8	HHHHHHHHHHHHHHHHHHHHHHHHCCCCCCC
426	EEEQRKQQDKRNNEKEHKEYQKQGRGQGHQP	-49.15	38.7	HHHHHHHHHHHHHHHHHHHHHHHCCCCCCCC
374	EEEERKKQDQEKNRKEDERYQREGRGKGHQP	-49.15	35.5	HHHHHHHHHHHHHHHHHHHHHHHHCCCCCCC
192	EEEERKQRDQQKNDREHERYQQQGRGQGHQP	-49.13	29.0	HHHHHHHHHHHHHHHHHHHHHHHHCCCCCCC
558	EQEERKQQDQRKNDKEDRRYQREGKGKGNNN	-49.13	32.3	HHHHHHHHHHHHHHHHHHHHHHHCCCCCCCC
832	EQEQRKQEEQRSNDEEDEYYQKYGQGKGHQP	-49.12	29.0	HHHHHHHHHHHHHHHHHHHHHHHHHCCCCCC
541	DEERKKNEDKYRNEYEHEEYQRKGRGQGHQP	-49.11	35.5	HHHHHHHHHHHHHHHHHHHHHHHHCCCCCCC
998	EEEEKKKQDQEKNEKEDKRYQREGRGKGHQP	-49.11	41.9	CHHHHHHHHHHHHHHHHHHHHHHHCCCCCCC
304	EEEERKKKDQEKNEREDEEYQRRGRGKGHQP	-49.11	38.7	CHHHHHHHHHHHHHHHHHHHHHHHCCCCCCC
924	EEEQKKQEDQRKNDKEHEEYQRYGRGQGHQP	-49.10	32.3	HHHHHHHHHHHHHHHHHHHHHHHHHCCCCCC
253	EQEERKQQDQRKNEWEDEEYQRKGRGKGNQN	-49.05	35.5	HHHHHHHHHHHHHHHHHHHHHHHHCCCCCCC
786	EEEERKKRDQWKNRWEDEYYQKEGQGKGHQP	-49.05	35.5	HHHHHHHHHHHHHHHHHHHHHHHHCCCCCCC
267	EQEQRKQEDQRRNDKEHERYQQQGWGQGHYP	-49.03	25.8	HHHHHHHHHHHHHHHHHHHHHHHCCCCCCCC
283	EQEERIQRDQWKNEWEQQYYQKKGQGQGHQS	-49.00	29.0	HHHHHHHHHHHHHHHHHHHHHHHHCCCCCCC
948	EEEERKWRDQQSNDKEHEEYQRRGRGRGHQP	-49.00	29.0	CHHHHHHHHHHHCHHHHHHHHHHHCCCCCCC
474	EEEEKKKRDQYKNDWEDRRYQREGKGKGHNN	-49.00	38.7	CHHHHHHHHHHHHHHHHHHHHHCCCCCCCCC
368	EQEERKKQDQQNNDKEDREYQKRGWGKGHYP	-49.00	35.5	HHHHHHHHHHHHCHHHHHHHHHHHCCCCCCC
431	QEEENKKEDQRKNDKEDREYQKQGRGKGHQP	-49.00	32.3	CHHHHHHHHHHHHHHHHHHHHHHHCCCCCCC
379	EQEERKNQDKKKNEEEHKRYQKEGQGQGHQS	-49.00	32.3	HHHHHHHHHHHHHHHHHHHHHHHHCCCCCCC
164	EQEERKKEDQWRNRWEDEYYQKYGQGKGHQP	-49.00	32.3	HHHHHHHHHHHHHHHHHHHHHHHHHCCCCCC
338	QQEENKKQDQYNNRYEDEEYQRKGRGKGHQP	-49.00	32.3	CHHHHHHHHHHHHHHHHHHHHHHHCCCCCCC
004	QEQENKKQDQYNNDWEDRRYQREGRGKGHQP	-48.99	38.7	CHHHHHHHHHHCCHHHHHHHHHHCCCCCCCC
689	EEEERKQQDKRKNENEHKQYQQRGQGQGHQS	-48.99	35.5	HHHHHHHHHHHHHHHHHHHHHHHHHCCCCCC
860	EREERYQQDQRKNRKEDERYQREGRGKGHQP	-48.99	32.3	CHHHHHHHHHHHHHHHHHHHHHHHCCCCCCC
582	EEEERKKQDQKKNEEEDERYQQQGRGKGHQP	-48.98	38.7	HHHHHHHHHHHHHHHHHHHHHHHHCCCCCCC
119	EEEERKKQDQYKNEAEDERYQQQGQGKGHQS	-48.98	41.9	HHHHHHHHHHHHHHHHHHHHHHHHCCCCCCC
652	EEEERKKQDQYRNEWEDEEYQRKGRGKGHQP	-48.98	38.7	HHHHHHHHHHHHHHHHHHHHHHHHCCCCCCC
788	EEEKRKQEDQRKNDKEHKEYQKQGKGQGHNN	-48.97	29.0	HHHHHHHHHHHHHHHHHHHHHHHCCCCCCCC
734	EQEERKQRDQYKNDWEHEEYQRKGRGQGHQP	-48.97	25.8	HHHHHHHHHHHHHHHHHHHHHHHHCCCCCCC
440	EQEERIQQDQRKNDKEQRRYQREGKGQGHNH	-48.97	25.8	CHHHHHHHHHHHHHHHHHHHHHHHCCCCCCC
505	ERQQRYQQDQYNNEWEDKRYQREGRGKGHQP	-48.96	41.9	CHHHHHHHHHHCCHHHHHHHHHHHCCCCCCC

864	ERQQRYQQDQYNNEYEDEEYQRKGRGKGHNP	-48.96	41.9	CHHHHHHHHHHCCCHHHHHHHHHCCCCCCCC
970	EEQERKKEDQYRNRFEDEYYQQKGQGKGHQP	-48.95	38.7	HHHHHHHHHHHHHHHHHHHHHHHHCCCCCCC
571	EEEERKKEDKKRNEEEDKRYQRYGRGKGNQN	-48.94	41.9	HHHHHHHHHHHHHHHHHHHHHHHCCCCCCCC
359	EEEERKQRDQYKNEWEDEEYQRKGRGKGHQP	-48.93	38.7	HHHHHHHHHHHHHHHHHHHHHHHHCCCCCCC
645	EEEEKKKRDQYNNEWEDEYYQRKGQGKGHNH	-48.93	45.2	CHHHHHHHHHHHHHHHHHHHHHHHCCCCCCC
465	EEEERKKEDQWKNEWEDSYYQQYGQGKGHQP	-48.93	38.7	HHHHHHHHHHHHHHHHHHHHHHHHHCCCCCC
975	EEEERKKEDQYRNEYEDKSYQRYGRGKGHQP	-48.93	38.7	HHHHHHHHHHHHHHHHHHHHHHHCCCCCCCC
680	EEEERKKQDQYNNEWEDEEYQRKGRGKGHQP	-48.93	41.9	HHHHHHHHHHHHHHHHHHHHHHHHCCCCCCC
324	EEEERKKQDQYNNEYEDEEYQRKGRGKGHQP	-48.93	41.9	HHHHHHHHHHHHHHHHHHHHHHHHCCCCCCC
071	EQEERKKQDQYNNEYEDEEYQRKGRGKGHQP	-48.93	38.7	HHHHHHHHHHHHHHHHHHHHHHHHCCCCCCC
451	QEEEKKKEDQYRNEWEDERYQQEGRGKGHQP	-48.93	38.7	CHHHHHHHHHHHHHHHHHHHHHHHCCCCCCC
169	EEEEKKRQDMKNNREEDERYQRYGWGKGHYP	-48.91	41.9	CHHHHHHHHHHHHHHHHHHHHHHCCCCCCCC
515	EEEERKQRQQRKQDQEDRRYQREGKGKGHNH	-48.91	32.3	HHHHHHHHHHHHHHHHHHHHHHHCCCCCCCC
724	QDQERKQQDQRKNDYEHEEYQRKGRGQGHQP	-48.90	25.8	CHHHHHHHHHHHHHHHHHHHHHHHCCCCCCC
911	EEERRKQEDAYRNRYEDEEYQRKGRGKGHQP	-48.90	35.5	HHHHHHHHHHHHHHHHHHHHHHHHCCCCCCC
845	QEEKKKQEDARKNRQEDQRYQREGRGKGHQP	-48.90	35.5	HHHHHHHHHHHHHHHHHHHHHHHHCCCCCCC
170	EEEQRKNEQARQERREDEEYQRKGRGKGHQP	-48.87	32.3	HHHHHHHHHHHHHHHHHHHHHHHHCCCCCCC
696	EEQEKKNQDQQKNDKEHKRYQRYGRGQGHQP	-48.87	35.5	HHHHHHHHHHHHHHHHHHHHHHHHCCCCCCC
661	EEEERKQQDARKNRKEDEEYQRRGRGKGHQP	-48.83	35.5	HHHHHHHHHHHHHHHHHHHHHHHHCCCCCCC
902	EEEERKKQDQWKNEWEHKYKQKKGQGCGKQN	-48.83	29.0	HHHHHHHHHHHHHHHHHHHHHHCCCCCCCCC
337	EREERYQRDQYKNEYEDEEYQRKGRGKGHQP	-48.82	35.5	CHHHHHHHHHHHHHHHHHHHHHHHCCCCCCC
705	EQQERKNREKYKNESEDKRYQKEGKGKGHNN	-48.80	38.7	HHHHHHHHHHHHHHHHHHHHHHCCCCCCCCC
424	QDEERKQRDQQKNDKEHKRYQREGQGQGHQS	-48.77	22.6	CHHHHHHHHHHHHHHHHHHHHHHHCCCCCCC
945	DEERKRQEDQRRNDKEHEEYQRYGQGQGHQS	-48.77	29.0	HHHHHHHHHHHHHHHHHHHHHHHHHCCCCCC
766	EEEERKQQDQRRHRNEHERYQREGRGQGHQP	-48.75	32.3	HHHHHHHHHHHHHHHHHHHHHHHHCCCCCCC
354	EEQQRKQEQQRRNDKEDEEYQRYGWGKGHYP	-48.74	35.5	HHHHHHHHHHHHHHHHHHHHHHHCCCCCCCC
829	EEEEKKKRDQESNEKEDEEYQRRGQGKGHQP	-48.72	41.9	CHHHHHHHHHHHHHHHHHHHHHHHHCCCCCC
826	EQEERIKQDQQKNEKEQEEYQRRGRGQGHQP	-48.72	29.0	CHHHHHHHHHHHHHHHHHHHHHHHHCCCCCC
595	EEEQRKKEDQRKNDEEDEEYQRKGRGKGHNP	-48.71	35.5	HHHHHHHHHHHHHHHHHHHHHHHHCCCCCCC
753	EEEQRKKEDQRKNDEEDERYQQEGRGKGHQP	-48.71	35.5	HHHHHHHHHHHHHHHHHHHHHHHHCCCCCCC
110	QDEQRKQEDQRKNDKEHEEYQRRGRGQGHQP	-48.71	22.6	CHHHHHHHHHHHHHHHHHHHHHHHHCCCCCC
954	EEEERKSRDQYKNDYEDREYQRKGRGKGHQP	-48.70	35.5	CHHHHHHHHHHHHHHHHHHHHHHHCCCCCCC
191	EEERRKQEDKYRNEWEHEEYQRKGRGQGHQP	-48.67	35.5	HHHHHHHHHHHHHHHHHHHHHHHHCCCCCCC
046	QEQQNKQEDQRRNDKEHRRYQREGKGQGHNH	-48.66	29.0	HHHHHHHHHHHHHHHHHHHHHHHHCCCCCCC
866	DNERRIQEEKYRNEYEQEEYQRKGRGQGHQP	-48.66	25.8	CHHHHHHHHHHHHHHHHHHHHHHHCCCCCCC
642	EEEERKKQDKWKNEWEDEYYQRKGQGKGHNP	-48.65	41.9	HHHHHHHHHHHHHHHHHHHHHHHHCCCCCCC
556	EEEKKKQEDQRKNEEEHKYFQKEGQGQGKQN	-48.65	32.3	HHHHHHHHHHHHHHHHHHHHHHHHCCCCCCC
706	QREKQIQEEKRSNEEEQKRYQRYGWGQGHYP	-48.65	25.8	HHHHHHHHHHHHHHHHHHHHHHHCCCCCCCC
999	EEERRKQEDKERNEKEHKRYQREGRGRGHQP	-48.65	35.5	HHHHHHHHHHHHHHHHHHHHHHHHCCCCCCC
598	DNQRRKNHEQESNDKEDKRYQREGRGKGHQP	-48.63	29.0	CHHHHHHHHHHHCHHHHHHHHHHHCCCCCCC
679	EEEERKRQDKEKNEKEDKRYQREGRGKGHQP	-48.63	41.9	HHHHHHHHHHHHHHHHHHHHHHHHCCCCCCC
243	EEEERKKEDQRKNDEEDRRYQRYGRGKGHQP	-48.61	35.5	HHHHHHHHHHHHHHHHHHHHHHHHCCCCCCC
763	EEEERKQRDKEKNEKEHKRYQREGRGRGHQP	-48.57	35.5	HHHHHHHHHHHHHHHHHHHHHHHHCCCCCCC
808	QDQERKKQDQYNNRWEDERYQREGRGKGHQP	-48.56	35.5	CHHHHHHHHHHHHHHHHHHHHHHHCCCCCCC
501	EEEEKKQRDQYKNDSEHQRYQRYGRGQGHQP	-48.56	32.3	HHHHHHHHHHHCHHHHHHHHHHHHCCCCCCC
187	EEEERKQRDQYKNDWEHEEYQRKGRGQGHQP	-48.56	29.0	HHHHHHHHHHHHHHHHHHHHHHHHCCCCCCC
233	EEEERKQRDQYKNDWEHEEYQRQGRGQGHQP	-48.56	29.0	HHHHHHHHHHHHHHHHHHHHHHHHCCCCCCC
986	EEERRKQEDQYRNDYEHEEYQRKGRGQGHQP	-48.56	29.0	HHHHHHHHHHHHHHHHHHHHHHHHCCCCCCC
795	EQEQRINEDFRKNREEQERYQREGQGQGHQS	-48.55	25.8	HHHHHHHHHHHHHHHHHHHHHHHHHCCCCCC
930	EEERKKQEDQRSNDEEDERYQRQGRGKGHQP	-48.54	38.7	HHHHHHHHHHHHHHHHHHHHHHHHCCCCCCC
290	EEEERKKEDQRKNEEEDEYYQKYGQGKGHQP	-48.53	38.7	HHHHHHHHHHHHHHHHHHHHHHHHHCCCCCC
435	EEEERKQRDKQKNEREQEEYQRYGRGQGHQP	-48.53	35.5	HHHHHHHHHHHHHHHHHHHHHHHHHCCCCCC
261	QEEEKKQRDKQKNEREQKRYQRYGRGQGHQP	-48.53	35.5	CHHHHHHHHHHHHHHHHHHHHHHHHCCCCCC
875	EQEERKKEDQYRNESEHQRKQNQGKGCGKNH	-48.51	25.8	HHHHHHHHHHHHHHHHHHHHHHCCCCCCCCC
154	EEEQKKQQDQYSNDYEHEEYQRKGRGQGHQP	-48.49	32.3	HHHHHHHHHHHHHHHHHHHHHHHHCCCCCCC
248	EQEERIKKDQEKNEKEQEEYQRRGRGQGHQP	-48.49	29.0	CHHHHHHHHHHHHHHHHHHHHHHHHCCCCCC
070	EQEERIKQDQKSNEEEQQRYQRYGQGQGHQS	-48.49	29.0	CHHHHHHHHHHCHHHHHHHHHHHHHCCCCCC
613	EQEERIKQDQQNNEAEQKRYQREGKGQGHNN	-48.49	35.5	CHHHHHHHHHHHHHHHHHHHHHHHCCCCCCC
310	EEQQRKQEDQRRNDKEQEEYQRYGKGQGHNN	-48.49	32.3	HHHHHHHHHHHHHHHHHHHHHHHHHCCCCCC
701	EEEERKRQDKEKNEKEHKRKQRYGKGCGKSN	-48.48	32.3	HHHHHHHHHHHHHHHHHHHHHHCCCCCCCCC
387	EEQQRKQEDKWRNEWEQKYYQKKGQGQGHQP	-48.47	38.7	HHHHHHHHHHHHHHHHHHHHHHHHCCCCCCC
161	EEEQRKQQDKYNNEWEQKRYQREGRGQGHQP	-48.47	38.7	HHHHHHHHHHHHHHHHHHHHHHHHCCCCCCC
575	EEEERKQRDQEKNDKEDERYQREGKGKGHSH	-48.47	35.5	HHHHHHHHHHHHHHHHHHHHHHHCCCCCCCC
062	EEEEKKQQDQRKNEKEHEEYQRRGKGQGHNH	-48.46	35.5	CHHHHHHHHHHHHHHHHHHHHHHHCCCCCCC
022	EQEERKQQDQRRHENEHKSYQRYGRGQGHQP	-48.45	32.3	HHHHHHHHHHHHHHHHHHHHHHHHCCCCCCC
032	EQEQRKQEEQRENEREDKEYQREGRGKGHQP	-48.45	32.3	HHHHHHHHHHHHHHHHHHHHHHHHCCCCCCC
230	EEQEKKNQDQQKNDKEQEEYQRRGWGQGHYP	-48.44	35.5	HHHHHHHHHHHHHHHHHHHHHHHCCCCCCCC

299	EEEERKNQDQYKNEWEDEEYQRKGRGKGHQP	-48.44	38.7	HHHHHHHHHHHHHHHHHHHHHHHHCCCCCCC
978	EEEERKNQDQYKNEYEDEEYQRKGRGKGHQP	-48.44	38.7	HHHHHHHHHHHHHHHHHHHHHHHHCCCCCCC
888	EQEQRKKEDKRKNEEEHKQKQKQGQGCGKQS	-48.44	29.0	HHHHHHHHHHHHHHHHHHHHHHHCCCCCCCC
143	EEQRRKQEDQYRNDWEHQRKQREGRGCGKQN	-48.44	29.0	HHHHHHHHHHHHHHHHHHHHHHCCCCCCCCC
011	EEEQRKQEQQRKNDKEDEEYQRRGRGKGHQP	-48.42	32.3	HHHHHHHHHHHHHHHHHHHHHHHHCCCCCCC
691	EEEERKQRDAWKNRWEDQYYQKQGQGKGHQP	-48.38	35.5	HHHHHHHHHHHHHHHHHHHHHHHHCCCCCCC
848	EEEQRKQEDQRKNDKEQKRYQREGKGQGHQP	-48.35	29.0	HHHHHHHHHHHHHHHHHHHHHHHHCCCCCCC
371	QEEQNKQEDQRKNDKEQKRYQREGRGQGHQP	-48.35	25.8	HHHHHHHHHHHHHHHHHHHHHHHHCCCCCCC
843	EQEERKKQDKEKNEKEHEEYQRRGRGQGHQP	-48.34	32.3	HHHHHHHHHHHHHHHHHHHHHHHHCCCCCCC
419	EEEERKQQDQRKNDKEHRRYQREGRGQGHQP	-48.34	29.0	HHHHHHHHHHHHHHHHHHHHHHHHCCCCCCC
767	EEEERKKEDKRKNEKEHKRYQRYGRGQGHQP	-48.33	35.5	HHHHHHHHHHHHHHHHHHHHHHHHCCCCCCC
477	EEEQRKHEDQNRNRKEDERYQREGKGKGHNN	-48.33	35.5	HHHHHHHHHHHHHHHHHHHHHHHCCCCCCCC
553	QEQQKKQEDFRRNKKEDEEYQRRGRGKGNQN	-48.32	38.7	HHHHHHHHHHHHHHHHHHHHHHHHCCCCCCC
820	EEEQRKQEDQERNDKEHQRKQRYGKGCGKNN	-48.32	25.8	HHHHHHHHHHHHHHHHHHHHHHCCCCCCCCC
904	EEEQRKQHDQQKNEKEHEEYQRRGKGQGHNH	-48.32	32.3	HHHHHHHHHHHHHHHHHHHHHHHHCCCCCCC
399	EQEERIKEDQYRNEYEQKSYQRYGRGQGHQP	-48.31	29.0	HHHHHHHHHHHHHHHHHHHHHHHHCCCCCCC
392	EQEERKQQEQRKNDREDRYYQREGKGKGHNH	-48.29	29.0	HHHHHHHHHHHHHHHHHHHHHHCCCCCCCCC
493	EEQQRKQEQQRRNDKEDRRFQRYGRGKGKQN	-48.29	32.3	HHHHHHHHHHHHHHHHHHHHHHHHCCCCCCC
817	ENEERKKQEQFNQDFEDEYYQKYGQGKGHQP	-48.29	35.5	CHHHHHHHHHHHHHHHHHHHHHHHHCCCCCC
835	QEERKKQEDQRSNEKEHKEYQKQGKGRGTSN	-48.29	32.3	HHHHHHHHHHHHHHHHHHHHHHHCCCCCCCC
402	EEEKRKQEEQRSNEEEDKYYQKKGQGKGHQP	-48.29	35.5	HHHHHHHHHHHHHHHHHHHHHHHHCCCCCCC
662	EEEERKWQDQKRNREEDERFQKQGNGKGKQH	-48.28	32.3	CHHHHHHHHHHHHHHHHHHHHHHCCCCCCCC
850	EEEQRKQQDQRNNKKEHEEYQRRGWGQGHYP	-48.27	32.3	HHHHHHHHHHHHHHHHHHHHHHHCCCCCCCC
894	EEEERKRQDQESNDKEDEEYQRRGRGKGHQP	-48.26	35.5	HHHHHHHHHHHHCCHHHHHHHHHCCCCCCCC
460	EEEERKRQDQESNDREDERYQREGKGKGHNH	-48.26	35.5	HHHHHHHHHHHHCHHHHHHHHHHCCCCCCCC
030	EEQQRKQQDHYNQRYEDEEYQRKGRGKGHQP	-48.25	41.9	HHHHHHHHHHHHHHHHHHHHHHHHCCCCCCC
188	NDQRRIQEDQERNRKEDEEYQRRGQGKGHQS	-48.23	32.3	CHHHHHHHHHHHHHHHHHHHHHHHHCCCCCC
637	DQERKKQEDQYRNDYEQQEYQRKGRGQGHQP	-48.23	25.8	HHHHHHHHHHHHHHHHHHHHHHHHCCCCCCC
391	EQEERKRQDQEKNEKEDEEFQRRGRGKGKQN	-48.22	32.3	HHHHHHHHHHHHHHHHHHHHHHHHCCCCCCC
896	EEEERKREDKRKNEAEDKRYQREGRGKGHQP	-48.20	45.2	HHHHHHHHHHHHHHHHHHHHHHHHCCCCCCC
303	EEEERKRQDKENNEREDKRYQREGKGKGHNH	-48.20	45.2	HHHHHHHHHHHHHHHHHHHHHHHCCCCCCCC
206	EQEQRKQEEQRRNRKEDERYQREGRGKGHQP	-48.19	29.0	HHHHHHHHHHHHHHHHHHHHHHHHCCCCCCC
165	EEEQRKQQDKYNNEWEDKRYQREGRGKGHQP	-48.18	45.2	HHHHHHHHHHHHHHHHHHHHHHHHCCCCCCC
928	EQEERKRQDQEKNDKEDRRFQRYGQGKGKQS	-48.16	29.0	HHHHHHHHHHHHHHHHHHHHHHHHHCCCCCC
615	QEQEKKKQDQQKNDREHEEYQRRGRGQGHQP	-48.16	32.3	CHHHHHHHHHHHHHHHHHHHHHHHHCCCCCC
577	QEEQKKEEDQRKNDKEDEEYQRRGQGKGHQS	-48.16	35.5	CHHHHHHHHHHHHHHHHHHHHHHHHCCCCCC
194	EEEERKKQDKYNNEWEHKRYQREGRGQGHQP	-48.16	38.7	HHHHHHHHHHHHHHHHHHHHHHHHCCCCCCC
271	EQEQRKQEEQRRNEKEDEEYQRRGRGKGHQP	-48.15	32.3	HHHHHHHHHHHHHHHHHHHHHHHHCCCCCCC
929	EEEERKKQDQEKNEREDEEYQRRGQGKGHQS	-48.15	38.7	HHHHHHHHHHHHHHHHHHHHHHHHHCCCCCC
439	EEEERKKQDQEKNEREDEEYQRRGRGKGHQP	-48.15	38.7	HHHHHHHHHHHHHHHHHHHHHHHHCCCCCCC
398	EEEKKKEEDQQRHEREDEEYQRRGQGKGHQS	-48.14	45.2	HHHHHHHHHHHHHHHHHHHHHHHHHCCCCCC
010	EEEQKKEEDQKKNEQEDKRYQREGRGKGHQP	-48.13	41.9	CHHHHHHHHHHHHHHHHHHHHHHHCCCCCCC
286	EEEQRKEEDQKKNEKEDEEYQRRGWGKGHYP	-48.13	38.7	HHHHHHHHHHHHHHHHHHHHHHHCCCCCCCC
257	EEEQRKEEDQKKNEQEDQRYQREGQGKGHQS	-48.13	38.7	HHHHHHHHHHHHHHHHHHHHHHHHCCCCCCC
193	EEEEKKKRDQESNDKEHRRYQREGRGRGHQP	-48.12	32.3	CHHHHHHHHHHHHHHHHHHHHHHHCCCCCCC
481	QDQERKRQDQEKNDKEHKRKQRYGRGCGKQN	-48.12	22.6	CHHHHHHHHHHHHHHHHHHHHHHCCCCCCCC
147	EEEERKKQDQYRNEWEHEEYQRKGRGRGHQP	-48.11	32.3	HHHHHHHHHHHHHHHHHHHHHHHHCCCCCCC
957	EEQRRKQEDQERNDKEDKRYQREGKGKGHNH	-48.10	38.7	HHHHHHHHHHHHHHHHHHHHHHHHCCCCCCC
289	EQQERKNQEQWKNEWEDQYFQQKGQGKGKQN	-48.10	32.3	HHHHHHHHHHHHHHHHHHHHHHHHCCCCCCC
977	EEEQRKQEQQYRNEFEDQMYQRYGKGKGTQN	-48.10	35.5	HHHHHHHHHHHHHHHHHHHHHHHHHCCCCCC
319	EEEQRKQQDQRSNDKEQQEYQKQGRGQGHQP	-48.09	29.0	HHHHHHHHHHHHHHHHHHHHHHHHCCCCCCC
212	EEEERKKQDQWKNDWEHKYYQKKGQGQGHNH	-48.09	29.0	HHHHHHHHHHHHHHHHHHHHHHHHCCCCCCC
569	EEQERKKQDQKKNDEEHERYQQQGKGQGHNN	-48.08	32.3	HHHHHHHHHHHHHHHHHHHHHHHCCCCCCCC
088	EEEERKRQDQEKNEKEHSEYQQRGRGRGHQP	-48.08	32.3	HHHHHHHHHHHHHHHHHHHHHHHHCCCCCCC
529	EEERKKQEDQYRNEWEDEEFQRKGRGKGKQN	-48.08	38.7	HHHHHHHHHHHHHHHHHHHHHHHHCCCCCCC
159	DDERRKQHDQEKNDKEDEQYQRRGRGKGHQP	-48.07	29.0	CHHHHHHHHHHHCHHHHHHHHHHHCCCCCCC
444	EEERRKQEDQERQEKEDKRYQREGDGKGHQP	-48.07	38.7	HHHHHHHHHHHHHHHHHHHHHHHCCCCCCCC
630	QEERKKQEDQERQEKEDQYYQKEGQGKGHQP	-48.07	38.7	HHHHHHHHHHHHHHHHHHHHHHHHCCCCCCC
625	EEEERKKEDQKRNDKEHEEYQRRGRGQGHQP	-48.04	29.0	HHHHHHHHHHHHHHHHHHHHHHHHHCCCCCC
341	EEEERKKQDQYNNDWEHERYQREGWGQGHYP	-48.04	32.3	HHHHHHHHHHHCHHHHHHHHHHHHCCCCCCC
995	QEEEKKKEDQKRNDEEHKRYQREGRGQGHQP	-48.04	29.0	CHHHHHHHHHHHHHHHHHHHHHHHCCCCCCC
237	EEEERKRQDQDSNDREHEEKQRRGQGCGKQS	-48.04	25.8	HHHHHHHHHHCHCHHHHHHHHHHCCCCCCCC
353	QREKEIQEQQRSNREEHERYQKQGRGQGHQP	-48.03	19.4	CHHHHHHHHHHHHHHHHHHHHHHCCCCCCCC
931	NDEQRKKEDQRKNDEEHKRYQREGRGQGHQP	-48.02	22.6	CHHHHHHHHHHCHHHHHHHHHHHHCCCCCCC
148	QEEEKKQQDKRKNEQEHRRYQREGRGQGHQP	-48.02	35.5	CHHHHHHHHHHHHHHHHHHHHHHHCCCCCCC
317	EEQQRKQQDHRSNRQEDQRYQKEGWGKGHYP	-48.00	38.7	HHHHHHHHHHHHHHHHHHHHHHHHCCCCCCC
404	EEEKRKQEDFQKNRKEHERYQKEGQGQGHQS	-47.99	29.0	HHHHHHHHHHHHHHHHHHHHHHHHCCCCCCC

275	EEQQRKQEQQRRNDKEDRRYQREGRGKGHQP	-47.98	35.5	HHHHHHHHHHHHHHHHHHHHHHHHHCCCCCCC
810	QKEQEIQEQKRRNEKEQKRYQREGKGQGHNH	-47.97	25.8	CHHHHHHHHHHHHHHHHHHHHHHHHCCCCCCC
609	EEERRKQEDKERNEKEDKRYQREGKGKGHNH	-47.97	41.9	HHHHHHHHHHHHHHHHHHHHHHHHCCCCCCCC
530	EEEERKKQDQQKNDKEDREYQRRGQGKGHQP	-47.95	35.5	HHHHHHHHHHHHHHHHHHHHHHHHHCCCCCCC
887	EQEERKKQDQEKNDKEDRRYQREGRGKGHNH	-47.95	32.3	HHHHHHHHHHHHHHHHHHHHHHHHHCCCCCCC
617	EEERKKQEDQERNDKEHRRYQREGRGRGHQP	-47.95	32.3	HHHHHHHHHHHHHHHHHHHHHHHHHCCCCCCC
409	EEEEKKKQDQQKNEKEDEEYQRRGQGKGHQS	-47.94	41.9	CHHHHHHHHHHHHHHHHHHHHHHHHHCCCCCC
349	EEEERKKQDQIKNEKEDEEYQRYGRGKGHQP	-47.94	38.7	HHHHHHHHHHHHHHHHHHHHHHHHHHCCCCCC
045	EEEERKKQDQYKNEYEDQEYQRKGRGKGHQP	-47.94	38.7	HHHHHHHHHHHHHHHHHHHHHHHHHCCCCCCC
710	EEEQRKKEDQRKNEEEDKRYQREGRGKGHQP	-47.94	38.7	HHHHHHHHHHHHHHHHHHHHHHHHHCCCCCCC
375	EEEQRKKEDQRKNEEEDQRYQKEGRGKGHQP	-47.94	38.7	HHHHHHHHHHHHHHHHHHHHHHHHHCCCCCCC
548	EEEQRKKEDQRKNEEEDQRYQREGRGKGHQP	-47.94	38.7	HHHHHHHHHHHHHHHHHHHHHHHHHCCCCCCC
616	EQEERKKQDQEKNEREDERYQRQGRGKGHQP	-47.94	35.5	HHHHHHHHHHHHHHHHHHHHHHHHCCCCCCCC
259	EQEERKKQDQQKNEKEDEEYQQKGRGKGHQP	-47.94	35.5	HHHHHHHHHHHHHHHHHHHHHHHHHCCCCCCC
725	QEEEKKKQDQEKNEKEDKRYQREGRGKGHQP	-47.94	38.7	CHHHHHHHHHHHHHHHHHHHHHHHHCCCCCCC
792	QEEEKKKQDQQKNEKEDEEYQRRGKGKGHNN	-47.94	38.7	CHHHHHHHHHHHHHHHHHHHHHHHCCCCCCCC
362	QEEEKKKQDQYRNEWEDEEYQRQGRGKGHQP	-47.94	38.7	CHHHHHHHHHHHHHHHHHHHHHHHHCCCCCCC
823	QQEEEKKQDQYKNEWEDEEYQRKGRGKGHQP	-47.94	32.3	CHHHHHHHHHHHHHHHHHHHHHHHHCCCCCCC
566	EEERRKQEDQERNDKEQKRYQREGKGQGHNH	-47.94	29.0	HHHHHHHHHHHHHHHHHHHHHHHHHCCCCCCC
461	EEEERKRQDQEKNDKEDRRYQRYGQGKGHQS	-47.93	35.5	HHHHHHHHHHHCHHHHHHHHHHHHCCCCCCCC
612	EEQERKHQDQNKNRKEDEEYQKQGRGKGHQP	-47.92	38.7	HHHHHHHHHHHHHHHHHHHHHHHHCCCCCCCC
560	EQEQRKQEDKRRHEKEQKRYQREGRGQGHQP	-47.92	35.5	HHHHHHHHHHHHHHHHHHHHHHHHHCCCCCCC
714	EEQQKKNEEQQSNDREDRRYQREGRGKGHQP	-47.92	38.7	HHHHHHHHHHHHHHHHHHHHHHHHHCCCCCCC
417	EEERRKQEDQERHEKEDKRYQRYGRGKGHQP	-47.92	41.9	HHHHHHHHHHHHHHHHHHHHHHHHHCCCCCCC
539	EEERRKQEDQYRHEWEDEYYQRKGQGKGHQP	-47.92	41.9	HHHHHHHHHHHHHHHHHHHHHHHHHCCCCCCC
749	EEEEKKRQDQEKNEKEDEEYQRRGKGKGHNH	-47.91	41.9	CHHHHHHHHHHHHHHHHHHHHHHHHCCCCCCC
144	EEEEKKRQDQEKNEKEDKRYQREGRGKGHQP	-47.91	41.9	CHHHHHHHHHHHHHHHHHHHHHHHHCCCCCCC
356	EEEERKRQDQEKNEKEDERYQREGWGKGHYP	-47.91	38.7	HHHHHHHHHHHHHHHHHHHHHHHHHCCCCCCC
793	EEEERKRQDQEKNEKEDKRYQRYGRGKGHQP	-47.91	38.7	HHHHHHHHHHHHHHHHHHHHHHHHHCCCCCCC
403	EEEERKRQDQEKNEKEDKRYQRYGWGKGHYP	-47.91	38.7	HHHHHHHHHHHHHHHHHHHHHHHHCCCCCCCC
296	EEEERKRQDQEKNEKEDQRYQREGRGKGHQP	-47.91	38.7	HHHHHHHHHHHHHHHHHHHHHHHHHCCCCCCC
802	EQEERKRQDQEKNEKEDEEYQRKGRGKGHQP	-47.91	35.5	HHHHHHHHHHHHHHHHHHHHHHHHHCCCCCCC
874	QEEEKKRQDQEKNEKEDEEYQRRGRGKGHQP	-47.91	38.7	CHHHHHHHHHHHHHHHHHHHHHHHHCCCCCCC
666	QEEEKKRQDQEKNEKEDKRYQREGKGKGHNP	-47.91	38.7	CHHHHHHHHHHHHHHHHHHHHHHCCCCCCCCC
622	EEQQRKQQQQRSNEKEDEEYQRRGRGKGHQP	-47.91	38.7	HHHHHHHHHHHHHHHHHHHHHHHHHCCCCCCC
756	EEEQRKKQDQINNEKEDEEYQRRGRGKGHQP	-47.87	41.9	HHHHHHHHHHHHHHHHHHHHHHHHHCCCCCCC
518	EREQRYQEDQRKNDQEHKRYQRYGRGQGHQP	-47.87	25.8	HHHHHHHHHHHHHHHHHHHHHHHHHCCCCCCC
450	EEEKRKQEQQRENDREHREYQREGKGRGHNP	-47.86	25.8	HHHHHHHHHHHHHHHHHHHHHHHCCCCCCCCC
494	EEEERKQQDQRKHDYEQKEYQKEGRGQGHQP	-47.85	32.3	HHHHHHHHHHHHHHHHHHHHHHHHHCCCCCCC
882	EEQQRKQQQKYSNEYEDKEYQRKGRGKGNQN	-47.85	41.9	HHHHHHHHHHHHHHHHHHHHHHHHHCCCCCCC
378	EEEQRKQYDQKKNEEEDKRYQREGRGKGHQP	-47.84	38.7	HHHHHHHHHHHHHHHHHHHHHHHHHCCCCCCC
966	EEEKRKQLDQENHEKEDEEYQRRGRGKGHQP	-47.84	48.4	HHHHHHHHHHHHHHHHHHHHHHHHHCCCCCCC
733	EEQQRKQEDKRKNEKEQKRYQREGRGQGHQP	-47.84	38.7	HHHHHHHHHHHHHHHHHHHHHHHHHCCCCCCC
453	EEEERKSRDQQKNEKEDQEYQREGSGKGHQR	-47.83	41.9	CHHHHHHHHHHHHHHHHHHHHHHCCCCCCCCC
528	EEEERKKQDKKKNEEEHKRYQRYGQGQGHQS	-47.82	35.5	HHHHHHHHHHHHHHHHHHHHHHHHHCCCCCCC
229	EEQERKRQDQESNDREDQYYQKKGQGKGHQP	-47.82	38.7	HHHHHHHHHHHHHHHHHHHHHHHHHCCCCCCC
605	EEEQRKQEQQRKNRKEDERYQREGQGKGHQS	-47.81	32.3	HHHHHHHHHHHHHHHHHHHHHHHHHCCCCCCC
140	EEEKKKQEDQRKNDEEHRRYQRYGRGQGHQP	-47.80	32.3	HHHHHHHHHHHHHHHHHHHHHHHHHHCCCCCC
480	EEERKKQQDQYSNEYEDEEYQRKGRGKGHQP	-47.77	41.9	HHHHHHHHHHHHHHHHHHHHHHHHHCCCCCCC
273	EEERRKQEDQERNEKEDERYQQQGRGKGHQP	-47.77	38.7	HHHHHHHHHHHHHHHHHHHHHHHHHCCCCCCC
256	EEERRKQEDQERNEKEDKYYQKKGQGKGHQP	-47.77	38.7	HHHHHHHHHHHHHHHHHHHHHHHHHCCCCCCC
653	EEERRKQEDQERNEKEDQRYQNQGQGKGHQS	-47.77	38.7	HHHHHHHHHHHHHHHHHHHHHHHHCCCCCCCC
781	EEERRKQEDQRKNEEEDQRYQRYGRGKGHQP	-47.77	38.7	HHHHHHHHHHHHHHHHHHHHHHHHHCCCCCCC
741	EEERRKQLDQESNEREDEEYQRRGKGKGHNH	-47.77	41.9	HHHHHHHHHHHHHHHHHHHHHHHHHCCCCCCC
634	EEEERKQRDQYKNDYEQQEYQKQGQGQGHQS	-47.75	29.0	HHHHHHHHHHHHHHHHHHHHHHHHHCCCCCCC
231	QEEENKQRDQWKNDWEQQYYQKQGQGQGHQP	-47.75	25.8	CHHHHHHHHHHHHHHHHHHHHHHHHCCCCCCC
395	EQEERKQREQKSNREEDERYQRYGQGKGHQS	-47.75	29.0	HHHHHHHHHHHHHHHHHHHHHHHHHHCCCCCC
812	EEEERKQQQQRENEREDEEYQRKGQGKGHFS	-47.75	38.7	HHHHHHHHHHHHHHHHHHHHHHHHHCCCCCCC
068	QEERKKQEDQERNEKEHEEYQRRGRGRGHQP	-47.75	32.3	HHHHHHHHHHHHHHHHHHHHHHHHHCCCCCCC
137	EEEERKKQDQEKNRKEDERYQREGKGKGHNH	-47.73	35.5	HHHHHHHHHHHHHHHHHHHHHHHHCCCCCCCC
782	EEEQKIQEQQRRNQKEHERYQRQGRGQGHQP	-47.71	29.0	HHHHHHHHHHHHHHHHHHHHHHHHCCCCCCCC
567	EEEQRKKEDQRKNEKEDQEYQKQGQGKGHQS	-47.71	38.7	HHHHHHHHHHHHHHHHHHHHHHHHHCCCCCCC
099	DEERKKQHDQESNDKEDRRYQREGRGKGHQP	-47.70	35.5	HHHHHHHHHHHCHHHHHHHHHHHHHCCCCCCC
573	DEERKKQEDQERNEKEDERYQREGQGKGHNS	-47.69	38.7	CHHHHHHHHHHHHHHHHHHHHHHHCCCCCCCC
155	DEERKKQHDQESNEKEDKRYQRYGKGKGHNH	-47.69	38.7	HHHHHHHCHHHHHHHHHHHHHHHHCCCCCCCC
041	EEEERKQRDQESNEKEDERYQQQGRGKGHQP	-47.69	38.7	HHHHHHHHHHHHHHHHHHHHHHHHHCCCCCCC
895	EQEQRKQEEQYRNDSEDRRYQQQGRGKGHQP	-47.68	29.0	HHHHHHHHHHHHHHHHHHHHHHHHHCCCCCCC

502	EQEKRKQEDKRKNEEEQKRYQRQGRGQGHQP	-47.67	32.3	HHHHHHHHHHHHHHHHHHHHHHHHCCCCCCC
852	EEEERKQRDQEKNRKEDEYYQKYGQGKGHQP	-47.67	35.5	HHHHHHHHHHHHHHHHHHHHHHHHHCCCCCC
035	QEERKKQHDQESNRKEDERYQREGRGKGHQP	-47.67	35.5	HHHHHHHHHHHHHHHHHHHHHHHHCCCCCCC
376	EEEQKKQQDKYSNEYEQEEYQRQGRGQGHQP	-47.67	38.7	HHHHHHHHHHHHHHHHHHHHHHHHCCCCCCC
021	EEEEKKKRDQQKNDREQEEYQRRGRGQGHQP	-47.66	32.3	CHHHHHHHHHHHHHHHHHHHHHHHHCCCCCC
463	EEQQKKQEDQRRNEKEQQRYQREGRGQGHQP	-47.65	38.7	HHHHHHHHHHHHHHHHHHHHHHHHCCCCCCC
655	EEEKKKQEQQRSNEKEDEEYQRRGQGKGHQS	-47.65	38.7	HHHHHHHHHHHHHHHHHHHHHHHHHCCCCCC
162	QREEQIQREQYKNEYEQEEYQRKGRGQGHQP	-47.65	22.6	CHHHHHHHHHHHHHHHHHHHHHHHCCCCCCC
889	QREKQIQEEQRSNEEEQQRYQRQGKGQGHNN	-47.65	22.6	HHHHHHHHHHHHHHHHHHHHHHHCCCCCCCC
263	EQEQRKQEDQRRNEKEQKRYQREGKGQGHNN	-47.65	29.0	HHHHHHHHHHHHHHHHHHHHHHHCCCCCCCC
089	EEEERKREDQWRHEYEDEEYQRKGRGKGHQP	-47.63	41.9	CHHHHHHHHHHHHHHHHHHHHHHHCCCCCCC
979	EEEKRKEEDQRKNDEEDRRYQRYGRGKGHQP	-47.63	35.5	HHHHHHHHHHHHHHHHHHHHHHHHCCCCCCC
777	EEEERKSRDQQKNEREDEEYQRRGRGKGHQP	-47.59	38.7	CHHHHHHHHHHHHHHHHHHHHHHHCCCCCCC
007	EQEERLNRQQESNDKEDQRYQREGRGKGHQP	-47.59	29.0	HHHHHHHHHHHHCHHHHHHHHHHHCCCCCCC
620	EEEERKQRDQQKNEKEQKRYQREGRGQGHQP	-47.59	32.3	HHHHHHHHHHHHHHHHHHHHHHHHCCCCCCC
160	EEQERKQQDQQSNEREQERYQQQGRGQGHQP	-47.58	35.5	HHHHHHHHHHHHHHHHHHHHHHHHCCCCCCC
604	QDEQRKNEDQQKNDKEDRRYQREGRGKGHQP	-47.56	29.0	CHHHHHHHHHHHHHHHHHHHHHHHCCCCCCC
716	EEQQRKQEDQYRNRYEQEEYQRKGRGQGHQP	-47.56	32.3	HHHHHHHHHHHHHHHHHHHHHHHHCCCCCCC
755	EEERRKQEDQWRNDWEDRYYQKEGQGKGHQP	-47.54	35.5	HHHHHHHHHHHHHHHHHHHHHHHCCCCCCCC
721	EQQQRKQEEQYRNDWEHQRYQREGRGQGHQP	-47.53	25.8	HHHHHHHHHHHHHHHHHHHHHHHHCCCCCCC
674	EQEERKQREQRNNDYEHEEYQRKGRGQGHQP	-47.53	25.8	HHHHHHHHHHHHHHHHHHHHHHHHCCCCCCC
504	EEEERKKQQKYSNEYEDEEYQRKGRGKGHQP	-47.53	38.7	HHHHHHHHHHHHHHHHHHHHHHHHCCCCCCC
202	EEQQRKQEDQYRNEWEQKRYQREGKGQGHNN	-47.52	35.5	HHHHHHHHHHHHHHHHHHHHHHHCCCCCCCC
327	EEEQKKQRDQYKNEWEQEEYQRQGRGQGHQP	-47.52	35.5	HHHHHHHHHHHHHHHHHHHHHHHHCCCCCCC
432	EEEERKQQDQRKNEKEQEEYQRRGRGQGHQP	-47.52	32.3	HHHHHHHHHHHHHHHHHHHHHHHHHCCCCCC
514	QEEEKKQQDQRRNEKEQEEYQRRGRGQGHQP	-47.52	32.3	CHHHHHHHHHHHHHHHHHHHHHHHHCCCCCC
429	EEQQRKKEDQRKNEEEDERYQQQGKGKGHSS	-47.50	41.9	HHHHHHHHHHHHHHHHHHHHHHHCCCCCCCC
772	EEEERKREDQWRNDWEDRYYQKKGQGKGHQP	-47.49	35.5	HHHHHHHHHHHHHHHHHHHHHHHCCCCCCCC
855	EEEQKKQEDDRRNRKEDQRYQREGRGKGHQP	-47.49	38.7	HHHHHHHHHHHHHHHHHHHHHHHHCCCCCCC
555	EEEQRKEEDQKRNEREDEYYQQQGRGKGHQP	-47.49	38.7	HHHHHHHHHHHHHHHHHHHHHHHHCCCCCCC
128	EEEEKKRKDQYDNEWEDEEYQRKGRGKGHQP	-47.48	41.9	CHHHHHHHHHCCCHHHHHHHHHHHCCCCCCC
906	EEEEKKRRDQESNEEEDKRYQRYGRGKGHQP	-47.48	41.9	CHHHHHHHHHHHHHHHHHHHHHHHCCCCCCC
197	EEEERKRQDQENNEREDKRYQREGKGKGHNH	-47.48	41.9	HHHHHHHHHHHHHHHHHHHHHHHCCCCCCCC
342	EEEERKRQDQESNEREDQRYQREGRGKGHQP	-47.48	38.7	HHHHHHHHHHHHHHHHHHHHHHHHCCCCCCC
942	EEEERKRQDQESNEYEDEEYQRKGRGKGHQN	-47.48	38.7	HHHHHHHHHHHHHHHHHHHHHHHHCCCCCCC
141	EEEEKKKQDQQKNDKEDEEYQRYGRGKGDQR	-47.47	45.2	CHHHHHHHHHHHHHHHHHHHHHHCCCCCCCC
511	EEEKRKQEDQRKNDEEQRRYQRYGRGQGHQP	-47.46	29.0	HHHHHHHHHHHHHHHHHHHHHHHHHCCCCCC
484	EEEERKREDQQRNREEDERYQKQGRGKGHQP	-47.46	35.5	HHHHHHHHHHHHHHHHHHHHHHHCCCCCCCC
638	EEEKRKQEQQRSNEEEDKRYQREGRGKGHQP	-47.46	35.5	HHHHHHHHHHHHHHHHHHHHHHHHCCCCCCC
949	EEEKRKQEQQRSNEEEDKRYQRYGRGKGHQP	-47.46	35.5	HHHHHHHHHHHHHHHHHHHHHHHHCCCCCCC
066	EQEQRKQQDQQNNRREDERYQKQGRGKGHQP	-47.45	35.5	HHHHHHHHHHHHHHHHHHHHHHHCCCCCCCC
780	EEEQRKKNDQYNNDWEDRYYQQYGQGKGHQP	-47.44	38.7	HHHHHHHHHHCCHHHHHHHHHHHHHCCCCCC
216	EEEKRKQEDQRKNREEQEYYQKYGQGQGHQS	-47.43	29.0	HHHHHHHHHHHHHHHHHHHHHHHHHCCCCCC
167	EEEQRKQEDDQKNRKEDQRYQREGRGKGHQP	-47.43	35.5	HHHHHHHHHHHHHHHHHHHHHHHHCCCCCCC
355	EEQRRKQEDQQKNRREDEYYQKYGQGKGHQP	-47.42	38.7	HHHHHHHHHHHHHHHHHHHHHHHHHCCCCCC
475	EEEERKKQQQYKNDWEDEEYQRKGRGKGHQP	-47.41	32.3	HHHHHHHHHHHHHHHHHHHHHHHHCCCCCCC
079	EEEEKKKRDKQKNEREQEEYQRRGRGQGHQP	-47.41	38.7	CHHHHHHHHHHHHHHHHHHHHHHHHCCCCCC
264	QQQQNHQEEKRRNEKEDKRYQREGRGKGHQP	-47.40	35.5	CHHHHHHHHHHHHHHHHHHHHHHHCCCCCCC
464	EEEERKKQDKEKNEKEQKRYQREGRGQGHQP	-47.40	35.5	HHHHHHHHHHHHHHHHHHHHHHHHCCCCCCC
876	EEEKRKQEEQRKNDEEHKRYQKEGQGQGHQS	-47.40	25.8	HHHHHHHHHHHHHHHHHHHHHHHHCCCCCCC
175	EQEKRKQEEQRSNDEEHKRYQREGRGQGHQP	-47.40	22.6	HHHHHHHHHHHHHHHHHHHHHHHHCCCCCCC
405	EEEEKRRRDKEKNEREDKRYQREGRGKGHQP	-47.40	45.2	HHHHHHHHHHHHHHHHHHHHHHHHCCCCCCC
908	EEQKRKQEDQRKNEEEQERYQKQGRGQGHQP	-47.39	35.5	HHHHHHHHHHHHHHHHHHHHHHHHCCCCCCC
006	EEQKRKQEDQRKNEKEQEEYQRYGKGQGHNN	-47.39	35.5	HHHHHHHHHHHHHHHHHHHHHHHHHCCCCCC
563	EEEKKKEEDKRKNEQEDKRYQREGDGKGNSN	-47.39	45.2	HHHHHHHHHHHHHHHHHHHHHHHCCCCCCCC
588	EEEKRKQEDQRKNEEEQEYYQRKGQGQGHQP	-47.39	32.3	HHHHHHHHHHHHHHHHHHHHHHHHCCCCCCC
606	EEEKRKQEDQRSNEKEQEEYQRRGQGQGHQS	-47.39	32.3	HHHHHHHHHHHHHHHHHHHHHHHHHCCCCCC
526	EEEQRKQEDQRKNEEEQNYYQKEGQGQGHNH	-47.39	32.3	HHHHHHHHHHHHHHHHHHHHHHHHCCCCCCC
545	EEEERKQQDDRKNRKEDEEYQRYGKGKGHNN	-47.36	35.5	HHHHHHHHHHHHHHHHHHHHHHHHCCCCCCC
959	EQEQRKNEDQRNNEEEQERYQRYGKGQGHNH	-47.34	32.3	HHHHHHHHHHHCHHHHHHHHHHHHHCCCCCC
380	EQEERKQQEQRKNDKEHEEYQRRGRGQGHQP	-47.34	22.6	HHHHHHHHHHHHHHHHHHHHHHHHHCCCCCC
423	EQQERKKQDQQKNDREQEEYQRRGRGQGHQP	-47.33	29.0	HHHHHHHHHHHHHHHHHHHHHHHHHCCCCCC
112	EEEERKQQDQRKNEKEQKEYQRSGQGQGHQP	-47.33	35.5	HHHHHHHHHHHHHHHHHHHHHHHCCCCCCCC
856	EEEQKKQEDQRKNEKEQQRYQKEGRGQGHQP	-47.33	35.5	HHHHHHHHHHHHHHHHHHHHHHHHCCCCCCC
076	EEEQRKQEDQRKNEKEQQRYQREGRGQGHQP	-47.33	32.3	HHHHHHHHHHHHHHHHHHHHHHHHCCCCCCC
118	EQEERKQQDQRKNENEQKRYQREGKGQGHNN	-47.33	29.0	HHHHHHHHHHHHHHHHHHHHHHHCCCCCCCC
712	QEEEKKQQDQRKNEKEQEEYQRRGRGQGHQP	-47.33	32.3	CHHHHHHHHHHHHHHHHHHHHHHHHCCCCCC

551	EEEQKKQRQQRSHEKEDEEYQRRGWGKGHYP	-47.28	41.9	HHHHHHHHHHHHHHHHHHHHHHHCCCCCCCC
925	EEEQRKQEDDQKNRREHEEKQRRGQGCGKQN	-47.28	25.8	HHHHHHHHHHHHHHHHHHHHHHHCCCCCCCC
719	EQEERKKEDQRRNDKEQKYYQQQGQGQGHNP	-47.27	25.8	HHHHHHHHHHHHHHHHHHHHHHHHCCCCCCC
102	EQEQRHQEEKRKNEKEDEEYQRRGKGKGHNH	-47.27	35.5	HHHHHHHHHHHHHHHHHHHHHHHHCCCCCCC
492	EQEERKQRDQWKNDWEQRYYQKKGQGQGHNH	-47.27	25.8	HHHHHHHHHHHHHHHHHHHHHHHHCCCCCCC
579	EQEERKKQDQQKNEEEHKRYQRYGRGQGHQP	-47.26	29.0	HHHHHHHHHHHHHHHHHHHHHHHHCCCCCCC
486	NDQQRKQEDQRRNEKEQEEYQRRGRGQGHQP	-47.26	29.0	CHHHHHHHHHHHHHHHHHHHHHHHHCCCCCC
973	EEERKKQEDQERNEKEDQRYQRYGRGKGHQP	-47.25	41.9	HHHHHHHHHHHHHHHHHHHHHHHHCCCCCCC
210	EQEERHQQEKRENEREDKEYQRYGRGKGHQP	-47.24	35.5	CHHHHHHHHHHHHHHHHHHHHHHHCCCCCCC
568	EEEQRKNEDQRKNDEEHERYRQQGRGQGHQP	-47.22	25.8	HHHHHHHHHHHCHHHHHHHHHHHCCCCCCCC
938	EQQQRIQEQQRRHEKEQEEYQRRGRGQGHQP	-47.22	32.3	HHHHHHHHHHHHHHHHHHHHHHHHHCCCCCC
907	EEEERKKEDKRRNEEEQKRYQRYGRGQGHQP	-47.22	35.5	HHHHHHHHHHHHHHHHHHHHHHHHHCCCCCC
097	EEEERKRRDQERHDREHEYYQQQGRGQGHQP	-47.20	32.3	HHHHHHHHHHHHHHHHHHHHHHHHCCCCCCC
760	EEEKKKQEDQRKNEEEQEEYQRKGRGQGHQP	-47.20	35.5	HHHHHHHHHHHHHHHHHHHHHHHHCCCCCCC
094	EQEKRKQEDQRKNEEEQKSYQKEGRGQGHQP	-47.20	29.0	HHHHHHHHHHHHHHHHHHHHHHHHCCCCCCC
557	QEEKNKQEDQRKNEEEQERYQREGWGQGHYP	-47.20	29.0	CHHHHHHHHHHHHHHHHHHHHHHHCCCCCCC
281	EEEQRKEEDQKKNDKEHEEYQRYGRGQGHQP	-47.19	29.0	HHHHHHHHHHHHHHHHHHHHHHHHHCCCCCC
524	EEEKRKQEDARKNREEHERYQQQGRGQGHQP	-47.18	29.0	HHHHHHHHHHHHHHHHHHHHHHHHCCCCCCC
228	EEEERKQQDDRKNRWEDEYYQRKGQGKGHNH	-47.17	35.5	HHHHHHHHHHHHHHHHHHHHHHHHCCCCCCC
805	EQEERKQRDDRKNRQEDQRYQREGRGKGHQP	-47.17	32.3	HHHHHHHHHHHHHHHHHHHHHHHHCCCCCCC
564	EEERRKQEDQERNDKEDEEYQRRGRGKGNQN	-47.16	35.5	HHHHHHHHHHHHHHHHHHHHHHHHCCCCCCC
672	EQEKRHQEEKRSNEEEDKRYQREGQGKGHQS	-47.14	35.5	HHHHHHHHHHHHHHHHHHHHHHHHCCCCCCC
072	EQERRHQEEKKRNEEEDKRYQKQGRGKGHQP	-47.14	35.5	HHHHHHHHHHHHHHHHHHHHHHHCCCCCCCC
183	EQEKRIQEDHRKNREEQQRYQRYGRGQGHQP	-47.12	25.8	HHHHHHHHHHHHHHHHHHHHHHHHHCCCCCC
245	EEEERKQQDARKNRKEHEEYQRYGRGQGHQP	-47.11	29.0	HHHHHHHHHHHHHHHHHHHHHHHHHCCCCCC
813	EEEQRKNEDDRKNREEDQRYQREGDGKGHQP	-47.11	35.5	HHHHHHHHHHHHHHHHHHHHHHHCCCCCCCC
186	EQQQRHQQEQYSNDYEDEEYQRKGRGKGHQP	-47.11	32.3	HHHHHHHHHHHHCCHHHHHHHHHCCCCCCCC
507	EEEERKKQDQKNNDEEQKRYQQYGRGQGHQP	-47.10	32.3	HHHHHHHHHHHCHHHHHHHHHHHHHCCCCCC
013	EEQERKKQDQYNNDWEQKRYQREGRGQGHQP	-47.09	35.5	HHHHHHHHHHHHHHHHHHHHHHHHCCCCCCC
740	EEQQRKQEQKKRNEEEHEEYQRKGRGQGHQP	-47.06	35.5	HHHHHHHHHHHHHHHHHHHHHHHHCCCCCCC
956	EQEQRIQEDDRRNRKEQERYQQQGKGQGHNN	-47.05	25.8	HHHHHHHHHHHHHHHHHHHHHHHCCCCCCCC
415	EEQQRKQEDQYRNEWEQERYQQQGKGQGHNN	-47.05	35.5	HHHHHHHHHHHHHHHHHHHHHHHCCCCCCCC
135	EEEERKQRDDYKNRYEDEEYQRKGRGKGHQP	-47.04	35.5	HHHHHHHHHHHHHHHHHHHHHHHHCCCCCCC
531	EQEERKKQEQYSNDSEDRRYQRYGRGKGHQP	-47.04	29.0	HHHHHHHHHHHCCHHHHHHHHHHHCCCCCCC
060	EQEKRKQEDQRKNREEQERYQREGKGQGHNH	-47.01	25.8	HHHHHHHHHHHHHHHHHHHHHHHHCCCCCCC
865	EEEKRKQEDDRKNRAEDQRYQKEGRGKGHQP	-47.00	38.7	HHHHHHHHHHHHHHHHHHHHHHHHCCCCCCC
009	EEEQRKQEQQRRNEKEDQSYQRHGRGKGHQP	-47.00	35.5	HHHHHHHHHHHHHHHHHHHHHHHCCCCCCCC
276	SNEERLQRDQESNEKEDEEYQRRGQGKGNQN	-47.00	32.3	CHHHHHHHHHHHHHHHHHHHHHHHCCCCCCC
083	QEEEKKKQDQWRNDYEQEEYQRKGRGQGHQP	-47.00	29.0	CHHHHHHHHHHHHHHHHHHHHHHHCCCCCCC
692	QREEEIQQQQRKNEKEQKRYQREGKGQGHNH	-46.99	22.6	CHHHHHHHHHHHHHHHHHHHHHHHCCCCCCC
670	EQEERHQREQYSNDWEDEEYQRKGRGKGHQP	-46.98	29.0	CHHHHHHHHHHCCCHHHHHHHHHHCCCCCCC
578	QEEQNKQQQQYSNEYEDEEYQRKGRGKGHQP	-46.97	32.3	HHHHHHHHHHHHHHHHHHHHHHHHCCCCCCC
334	EEEERKQRQKYSNEFEHKYYQQKGQGQGHQP	-46.97	32.3	CHHHHHHHHHHHHHHHHHHHHHHHCCCCCCC
105	EEEERKRQDQEKNDREHQYYQREGQGQGHQS	-46.97	29.0	HHHHHHHHHHHHHHHHHHHHHHHHCCCCCCC
482	QEEEKKRQDQEKNDKEHKRYQKEGKGQGHNN	-46.97	29.0	CHHHHHHHHHHHHHHHHHHHHHHCCCCCCCC
163	EQEKRHQEEQRSNDKEHEEYQRRGRGRGHQP	-46.95	22.6	HHHHHHHHHHHHHHHHHHHHHHHHCCCCCCC
065	EEEKRKQEQQRSNEKEDEEYQRRGQGKGHQS	-46.94	35.5	HHHHHHHHHHHHHHHHHHHHHHHHHCCCCCC
801	EEEERKKQDKQKNEKEHEEYQRYGRGQGHQP	-46.93	35.5	HHHHHHHHHHHHHHHHHHHHHHHHHCCCCCC
798	EEEERKKQDKWKNEWEHKYYQKKGQGQGHQP	-46.93	35.5	HHHHHHHHHHHHHHHHHHHHHHHHCCCCCCC
325	EREERYQQDQRKNDKEQEEYQRRGRGQGHQP	-46.93	25.8	CHHHHHHHHHHHHHHHHHHHHHHHHCCCCCC
422	EEEERKQRQKKKNEEEHKRYQRYGRGQGHQP	-46.93	32.3	HHHHHHHHHHHHHHHHHHHHHHHHCCCCCCC
287	EREERYRQDKENNEREDKRYQREGRGKGHNP	-46.90	41.9	CHHHHHHHHHHHHHHHHHHHHHHCCCCCCCC
996	EEEQRKEEDMKKNRQEQQRYQREGRGQGHQP	-46.90	29.0	HHHHHHHHHHHHHHHHHHHHHHHHCCCCCCC
927	EEEQRKQKQQYDNDWEHEEYQRKGRGQGHNP	-46.89	25.8	HHHHHHHHHHHHHHHHHHHHHHHCCCCCCCC
258	EEEQKKYEDQRKNEEEHEEFQRKGRGQGKQN	-46.89	32.3	CHHHHHHHHHHCHHHHHHHHHHHHHCCCCCC
061	QEEKKKQEDQRSNDKEHEEYQRYGRGQGHQP	-46.82	29.0	HHHHHHHHHHHHHHHHHHHHHHHHHCCCCCC
623	EQERRKQEEKRSNEEEDKRYQKQGRGKGHQP	-46.77	35.5	HHHHHHHHHHHHHHHHHHHHHHHCCCCCCCC
708	EEERKKQEDKRKNEKEHEEYQRYGRGQGHQP	-46.76	38.7	HHHHHHHHHHHHHHHHHHHHHHHHHCCCCCC
489	EEERRKQEDKYRNEWEHKKYQREGWGQGHYP	-46.76	35.5	HHHHHHHHHHHHHHHHHHHHHHHHCCCCCCC
269	EEEEKRKRDQYKNESEHKRYQRYGKGQGHNH	-46.76	35.5	HHHHHHHHHHHCHHHHHHHHHHHHCCCCCCC
114	EEEEKKQQDQRKNDNEQRYYQEYGQGQGNQN	-46.75	32.3	CHHHHHHHHHHHHHHHHHHHHHHHHCCCCCC
809	EEEERKQRDQEKNDKEHEEYQRRGKGQGHNN	-46.75	29.0	HHHHHHHHHHHHHHHHHHHHHHHCCCCCCCC
078	EQQRRKQEDKERNEKEHKRYQREGKGQGHNH	-46.72	35.5	HHHHHHHHHHHHHHHHHHHHHHHHCCCCCCC
917	EEEQKKQRDQKKNEEEQERYQRYGTGQGHQP	-46.72	35.5	HHHHHHHHHHHHHHHHHHHHHHHCCCCCCCC
543	EEEQRKQEDQWRNEWEQEYYQRKGQGQGHQP	-46.72	32.3	HHHHHHHHHHHHHHHHHHHHHHHHCCCCCCC
236	EQEQRKQKDQEKNEREQKRYQREGRGQGHQP	-46.72	29.0	HHHHHHHHHHHHHHHHHHHHHHHHCCCCCCC
350	QDEERKKQDQDSNDREQKRYQREGRGQGHQP	-46.70	22.6	CHHHHHHHHHHHHHHHHHHHHHHHCCCCCCC

116	DNERRKQHEKESNEKEDKRYQRQGRGKGHQP	-46.69	32.3	CHHHHHHHHHHHHHHHHHHHHHHCCCCCCCC
498	EQEQRKEEEQKKNEKEDEEYQRRGKGKGTNN	-46.69	32.3	HHHHHHHHHHHHHHHHHHHHHHHHCCCCCCC
628	EEEQRKQHQQQSNDKEHEEYQRRGRGQGHQP	-46.69	25.8	HHHHHHHHHHHHHHHHHHHHHHHHHCCCCCC
675	EEEERKRRDAEKNRREDQRYQREGKGKGHNH	-46.69	35.5	HHHHHHHHHHHHHHHHHHHHHHHCCCCCCCC
676	EDEERRNQQQWKNDYEDEEYQRKGRGKGHNP	-46.68	29.0	CHHHHHHHHHHHHHHHHHHHHHHCCCCCCCC
631	EQEERKNQEQWKNEWEDEYYQQYGQGKGHQP	-46.67	32.3	HHHHHHHHHHHHHHHHHHHHHHHHHCCCCCC
301	EEQERKKQDQQSNDKEQKEYQKQGRGQGHQP	-46.66	32.3	HHHHHHHHHHHHHHHHHHHHHHHHCCCCCCC
145	EREQRYKEDQRKNDKEDREYQKQGRGKGHQP	-46.66	32.3	CHHHHHHHHHHHCHHHHHHHHHHHCCCCCCC
737	EEEERKRQDQEKNEKEHKRYQREGRGQGHQP	-46.64	32.3	HHHHHHHHHHHHHHHHHHHHHHHHCCCCCCC
664	EEQERKNREQYSNDWEDKRYQREGRGKGHQP	-46.64	35.5	HHHHHHHHHHHHHHHHHHHHHHHCCCCCCCC
698	EQEQRKQEDQRKNEKEQKRYQREGKGQGHNN	-46.60	29.0	HHHHHHHHHHHHHHHHHHHHHHHCCCCCCCC
495	EEEKRKEEDQKENEREHEEYQRKGRGQGHQP	-46.57	32.3	HHHHHHHHHHHHHHHHHHHHHHHHCCCCCCC
881	DRQRQYHEDQNRNEKEDEEYQRRGQGKGHQP	-46.57	35.5	CHHHHHHHHHHCCHHHHHHHHHHHHCCCCCC
091	EQEKRHQEEQRKNDKEHEEKQRRGQGCGKQS	-46.57	19.4	HHHHHHHHHHHHHHHHHHHHHHHCCCCCCCC
386	QEERKKQEDQERNQKEHDRYQREGRGQGHQP	-46.56	29.0	HHHHHHHHHHHHHHHHHHHHHHHCCCCCCCC
393	EEEEKKRQDQDNNDREHEEYQRRGRGQGHQP	-46.54	35.5	CHHHHHHHHHCCCHHHHHHHHHHHHCCCCCC
117	EEEEKKRRDQESNDKEHQRYQREGRGQGHQP	-46.54	32.3	CHHHHHHHHHHHHHHHHHHHHHHHCCCCCCC
302	EQEERKREDQKRNEEEQQRYQRYGRGQGHQP	-46.54	29.0	HHHHHHHHHHHHHHHHHHHHHHHHHCCCCCC
288	EEEERKKKQQYDNEYEDEEYQRKGRGKGHQP	-46.52	35.5	CHHHHHHHHHHHHHHHHHHHHHHHCCCCCCC
058	EEEERKKRDQYKNDSEHERYRQQGQGQGHQS	-46.52	25.8	HHHHHHHHHHCCCHHHHHHHHHHHCCCCCCC
799	EEEERKQQDQRKNENEQKRYQREGKGQGHNH	-46.50	32.3	HHHHHHHHHHHHHHHHHHHHHHHHCCCCCCC
584	QEQQKKQEDARRNRFEQQYYQQKGQGQGHQP	-46.50	32.3	HHHHHHHHHHHHHHHHHHHHHHHHCCCCCCC
601	QEEEKKKQDQEKNRKEQEEYQRRGRGQGHQP	-46.49	29.0	CHHHHHHHHHHHHHHHHHHHHHHHHCCCCCC
098	QEEQKKKEDQRKNDAEHRRFQREGQGQGKQS	-46.48	29.0	HHHHHHHHHHHHHHHHHHHHHHHHCCCCCCC
326	EEEEKKKQDDWRNRWEDQYYQKKGQGKGHQP	-46.48	38.7	CHHHHHHHHHHHHHHHHHHHHHHHCCCCCCC
687	EQQQRHQEEKRRNEKEDKYYQKEGQGKGNQN	-46.47	38.7	HHHHHHHHHHHHHHHHHHHHHHHCCCCCCCC
052	EQQQRHQQEQQSNEREDEEYQRRGKGKGHNN	-46.47	35.5	HHHHHHHHHHHHHHHHHHHHHHHCCCCCCCC
974	EEEERKLQDQQKNDKEQRRFQRYGKGQGKNN	-46.42	25.8	CHHHHHHHHHHHHHHHHHHHHHHHHCCCCCC
797	EEEEKKQQQQREDEYRDEEYQRKGRGKGHNP	-46.40	35.5	HHHHHHHHHHHHHHHHHHHHHHHCCCCCCCC
574	EQQQRHQEEQRQNEKEDKRYQKEGRGKGHQP	-46.40	35.5	HHHHHHHHHHHHHHHHHHHHHHHCCCCCCCC
040	QQEQNHQEEQRRNEKEDEEYQRRGRGKGHQP	-46.40	29.0	CHHHHHHHHHHHHHHHHHHHHHHHCCCCCCC
005	ENEERKNQEKQKHEKEQKRYQREGRGQGHQP	-46.39	32.3	CHHHHHHHHHHHHHHHHHHHHHHHCCCCCCC
255	EEEQRIQEQQRENEREQEEYQQKGWGQGHYP	-46.39	29.0	HHHHHHHHHHHHHHHHHHHHHHHCCCCCCCC
279	EQEERKWRDQKKNEEEHERYQQQGRGQGHQP	-46.34	29.0	CHHHHHHHHHHHHHHHHHHHHHHHCCCCCCC
646	QEEEKKRRDQESNEREHKYFQKKGQGQGKNN	-46.33	29.0	CHHHHHHHHHHHHHHHHHHHHHHHCCCCCCC
358	EEERRKQEDKKRNEEEDKRYQKQGRGKGHQP	-46.33	41.9	HHHHHHHHHHHHHHHHHHHHHHHCCCCCCCC
722	EEQERKKQDQYNNDWEQRRYQREGDGQGHQP	-46.33	35.5	HHHHHHHHHHHHHHHHHHHHHHHCCCCCCCC
014	EQEERKKQEQWKNDWEHEYYQKYGQGQGHQP	-46.33	22.6	HHHHHHHHHHHHHHHHHHHHHHHHHCCCCCC
018	EQERRKQEQKYKQEWEDEEYQRKGRGKGHQP	-46.28	35.5	HHHHHHHHHHHHHHHHHHHHHHHHCCCCCCC
833	EEQERKKQDQWKNDYEQRSYQREGRGQGHQP	-46.27	32.3	HHHHHHHHHHHHHHHHHHHHHHHCCCCCCCC
784	EQEQRHQEEQRKNEKEDKRYQREGRGKGHQP	-46.27	32.3	HHHHHHHHHHHHHHHHHHHHHHHHCCCCCCC
702	EQEQRHQEEQRKNEKEDKRYQRYGRGKGHQP	-46.27	32.3	HHHHHHHHHHHHHHHHHHHHHHHHCCCCCCC
436	DNQRRRNEEQWRNDWEDQYYQKKGQGKGHNH	-46.27	29.0	CHHHHHHHHHHHHHHHHHHHHHHHCCCCCCC
158	EEEEKKKRDQYKNESEQERYQRYGRGQGHQP	-46.27	35.5	CHHHHHHHHHHCHHHHHHHHHHHHHCCCCCC
649	EEEERKKEDQRKNEEEQQRYQRYGKGQGHNN	-46.27	32.3	HHHHHHHHHHHHHHHHHHHHHHHHCCCCCCC
746	EEEERKKEDQYRNEWEQEEYQRKGRGQGHQP	-46.27	32.3	HHHHHHHHHHHHHHHHHHHHHHHHCCCCCCC
412	EEEERKKQDQYNNEWEQKRYQREGQGQGHQS	-46.27	35.5	HHHHHHHHHHHHHHHHHHHHHHHHCCCCCCC
222	EQEERHQREQKSNEEEDKRFQREGDGKGKQN	-46.26	29.0	CHHHHHHHHHHHHHHHHHHHHHHCCCCCCCC
372	EQEKRHQEEQRSQEQEDEEYQRRGQGKGHQS	-46.24	32.3	HHHHHHHHHHHHHHHHHHHHHHHHHCCCCCC
496	EQEKRHQEEQRSNDEEDRRYQRYGQGKGHYS	-46.22	29.0	HHHHHHHHHHHHCHHHHHHHHHHHCCCCCCC
711	ENEERKQREQESQRKEDERYQREGRGKGHQP	-46.20	29.0	CHHHHHHHHHHHHHHHHHHHHHHHCCCCCCC
196	EEEERKKQDQYNNEWEQEEFQRKGRGQGKQN	-46.20	32.3	HHHHHHHHHHHHHHHHHHHHHHHHHCCCCCC
508	EQEERKRQDQEKNEKEHKRYQREGRGQGHQP	-46.19	29.0	HHHHHHHHHHHHHHHHHHHHHHHHCCCCCCC
036	EEEQRKEEDKKKNEREQKEYQQRGRGQGHQP	-46.19	35.5	CHHHHHHHHHHHHHHHHHHHHHHHCCCCCCC
311	EEEERKKQEQWKNEWEDQYYQKQGQGKGHQP	-46.18	35.5	HHHHHHHHHHHHHHHHHHHHHHHHCCCCCCC
932	EQEERKKQEQWKNEWEDQFYQKQGRGKGHQP	-46.18	35.5	HHHHHHHHHHHHHHHHHHHHHHHHCCCCCCC
390	EQEERHKEEQYRNDYEDEEYQRKGRGKGTQN	-46.17	29.0	HHHHHHHHHHHHHHHHHHHHHHHHCCCCCCC
121	EEEERKQQDARKNRYEQEEYQRKGRGQGHQP	-46.17	29.0	HHHHHHHHHHHHHHHHHHHHHHHHCCCCCCC
576	EEEQRKRYDQEKNRKEHENYQREGRGQGHQP	-46.17	29.0	HHHHHHHHHHHHHHHHHHHHHHHCCCCCCCC
081	EEERRKQEDQQKNEREHEEYQRKGRGQGHNP	-46.16	32.3	HHHHHHHHHHHHHHHHHHHHHHHCCCCCCCC
087	EEEERKQRQQYSNDWEHERYQREGRGQGHQP	-46.16	25.8	HHHHHHHHHHHHHHHHHHHHHHHHCCCCCCC
890	EQQQRHQEEQRRNDKEDERYQQQGWGKGHYP	-46.15	32.3	HHHHHHHHHHHHHHHHHHHHHHHCCCCCCCC
360	ENEERKRQEQEKNEKEDEEYQRRGWGKGHYP	-46.15	32.3	CHHHHHHHHHHHHHHHHHHHHHHCCCCCCCC
284	EQEKRHQEEQRSNEEEDKRYQKEGRGKGHQP	-46.14	32.3	HHHHHHHHHHHHHHHHHHHHHHHHCCCCCCC
136	QQEKNHQEEQRSNEAEDKQYQQRGRGKGHQP	-46.14	32.3	CHHHHHHHHHHHHHHHHHHHHHHHCCCCCCC
401	EQEERHQQEQRKNRWEDEYYQQKGQGKGHQP	-46.11	29.0	CHHHHHHHHHHHHHHHHHHHHHHHCCCCCCC
181	DEERKKQEEKERNEKEDKRYQQQGWGKGHYP	-46.10	38.7	HHHHHHHHHHHHHHHHHHHHHHHHCCCCCCC

834	EEEERKKQDQEKNDKEQRRYQREGRGQGHQP	-46.05	29.0	HHHHHHHHHHHHHHHHHHHHHHHHCCCCCCC
103	QEERKKQEDQWRNEWEHEYYQRKGQGQGHQP	-46.05	32.3	HHHHHHHHHHHHHHHHHHHHHHHHCCCCCCC
294	EQEERKKQEQFSNDFEHKYYQQKGQGQGHQP	-46.05	25.8	HHHHHHHHHHHHHHHHHHHHHHHHCCCCCCC
500	EHEERRQQEQRKNEYEDEEYQRKGRGKGHQP	-46.04	32.3	CHHHHHHHHHHHHHHHHHHHHHHHCCCCCCC
274	EQEERKRQDQERNDKEQEEYQRKGRGQGHQP	-46.03	25.8	HHHHHHHHHHHHHHHHHHHHHHHHCCCCCCC
774	EQEQRHNEEQRSNREEDERYQREGKGKGHNN	-46.01	29.0	HHHHHHHHHHHHHHHHHHHHHHHCCCCCCCC
418	EEERRKQEEQERNEKEDKRYQRYGRGKGHQP	-46.00	35.5	HHHHHHHHHHHHHHHHHHHHHHHHCCCCCCC
815	EEEQRKKEDKRKNEEEQKKYQEQGRGQGHQP	-45.99	35.5	HHHHHHHHHHHHHHHHHHHHHHHHCCCCCCC
748	EQERRKQEEQRSNRKEDEEYQRRGRGKGHQP	-45.98	29.0	HHHHHHHHHHHHHHHHHHHHHHHHCCCCCCC
220	EEEERKQRDQEKNEKEHERYQKQGRGQGHQP	-45.97	32.3	HHHHHHHHHHHHHHHHHHHHHHHCCCCCCCC
891	QEEEKKRQDKEKNEKEQKRYQRYGRGQGHQP	-45.97	35.5	CHHHHHHHHHHHHHHHHHHHHHHHHCCCCCC
723	ERQERYQRDQESNEKEDKRYQRYGRGKGHQP	-45.96	38.7	CHHHHHHHHHHHHHHHHHHHHHHHCCCCCCC
769	EQEQRKQEEQRRNDKEQRRYQREGKGQGHNN	-45.96	22.6	HHHHHHHHHHHHHHHHHHHHHHHCCCCCCCC
521	EQEERIQRDDYKHRWWHEEYQRKGRGQGHQP	-45.95	25.8	CHHHHHHHHHHHHHHHHHHHHHHCCCCCCCC
023	EQEKRHQEEQRKNEEEDERYQRQGQGKGHQS	-45.95	32.3	HHHHHHHHHHHHHHHHHHHHHHHHCCCCCCC
407	EEEQKKRQDQENNEKEHEEYQRRGRGQGHQP	-45.94	38.7	HHHHHHHHHHHHHHHHHHHHHHHHCCCCCCC
607	EEEQRKHEQQNRNEKEDKRYQRYGQGKGHQS	-45.91	35.5	HHHHHHHHHHHHHHHHHHHHHHHHCCCCCCC
408	EEEQKKEEDQKSNEQEHERYQREGQGQGHQP	-45.89	35.5	CHHHHHHHHHHHHHHHHHHHHHHHCCCCCCC
226	EEERKKQRDQEKNDKEQEEYQRRGQGQGHNP	-45.88	32.3	HHHHHHHHHHHHHHHHHHHHHHHHHCCCCCC
184	EQEERHKKEQEKNDKEDEEYQRYGRGKGHQP	-45.85	29.0	CHHHHHHHHHHHCHHHHHHHHHHHHCCCCCC
905	EEERRKQEDKERNEKEQKQYQQRGKGQGHNN	-45.82	35.5	HHHHHHHHHHHHHHHHHHHHHHHCCCCCCCC
180	ERQQRYQEDQYRNEYEQEEYQRKGRGQGHQP	-45.82	32.3	HHHHHHHHHHHHHHHHHHHHHHHHCCCCCCC
791	EEEERKRQDQEKNDREQKRYQREGRGQGHQP	-45.76	29.0	HHHHHHHHHHHHHHHHHHHHHHHHCCCCCCC
886	EEEERKRQDQESNEREHKRYQREGRGQGHQP	-45.76	32.3	HHHHHHHHHHHHHHHHHHHHHHHHCCCCCCC
964	EEERRKQEDKERNEKEQKRYQREGRGQGHQP	-45.74	35.5	HHHHHHHHHHHHHHHHHHHHHHHHCCCCCCC
034	EQEKRHQEEQRSNEKEDEEYQRRGKGKGHNH	-45.72	32.3	HHHHHHHHHHHHHHHHHHHHHHHHCCCCCCC
095	QQQQNHQEEKRRNEKEHKRYQRYGKGQGHNN	-45.68	29.0	CHHHHHHHHHHHHHHHHHHHHHHCCCCCCCC
345	EQERRLQEDQYRNEWEQEEYQRKGRGQGHQP	-45.66	29.0	HHHHHHHHHHHHHHHHHHHHHHHHCCCCCCC
800	EEEQRKQEDDRKNRKEHERYQREGRGQGHQP	-45.64	29.0	HHHHHHHHHHHHHHHHHHHHHHHHCCCCCCC
632	EQEERHQQEQRKNDKEDREYQKQGRGKGHQP	-45.63	29.0	CHHHHHHHHHHHCHHHHHHHHHHHCCCCCCC
241	EQEERHKEEQYRNDWEDERYQREGQGKGHQS	-45.61	29.0	HHHHHHHHHHHHHHHHHHHHHHHHCCCCCCC
752	EQEKRHQEEQRKNDEEHEEFQRKGRGQGKQN	-45.58	19.4	HHHHHHHHHHHCHHHHHHHHHHHHHCCCCCC
025	EQEERHQREQWKNRWEDEFYQKQGRGKGHQP	-45.57	32.3	CHHHHHHHHHHHHHHHHHHHHHHHCCCCCCC
941	EQEQRHQQEQDSNRREDEEYQRRGQGKGHQP	-45.54	29.0	HHHHHHHHHHHHHHHHHHHHHHHHCCCCCCC
246	EEEERKKQQQYKNEWEDKRYQREGDGKGHNP	-45.54	35.5	HHHHHHHHHHHHHHHHHHHHHHCCCCCCCCC
678	EEEQRKQEDQQKNEREDEEYQRRGQGKGHQS	-45.50	38.7	HHHHHHHHHHHHHHHHHHHHHHHHHCCCCCC
602	EQEERKQQQKRKNEKEQKRYQREGRGQGHQP	-45.49	29.0	HHHHHHHHHHHHHHHHHHHHHHHHCCCCCCC
533	QEERKKQHDQEKNQKEQDRYQREGRGQGHQP	-45.41	29.0	HHHHHHHHHHHHHHHHHHHHHHHHCCCCCCC
544	QEQEKKKEDQNRNRKEQERYQREGRGQGHQP	-45.39	32.3	CHHHHHHHHHHHHHHHHHHHHHHHCCCCCCC
671	EQEQRHQEEQRKNDEEHRQYQERGRGQGHQP	-45.38	22.6	HHHHHHHHHHHHHHHHHHHHHHHHCCCCCCC
336	EQEERHQQEQRSNEKEDEEYQRRGQGKGNQN	-45.33	32.3	CHHHHHHHHHHHHHHHHHHHHHHHCCCCCCC
715	DEERKKQEDQERNDKEQKRYQREGQGQGHQP	-45.33	29.0	CHHHHHHHHHHHHHHHHHHHHHHHCCCCCCC
816	EEEQRKQHDQEKNDKEQEEYQKRGRGQGHQP	-45.33	29.0	HHHHHHHHHHHHHHHHHHHHHHHHCCCCCCC
182	EEERRRQEEQYRNEWEDERYQRQGKGKGHNN	-45.32	35.5	HHHHHHHHHHHHHHHHHHHHHHHCCCCCCCC
090	EQQERKRQDQESNRREHERYQKQGRGQGHQP	-45.30	29.0	HHHHHHHHHHHHHHHHHHHHHHHCCCCCCCC
459	EQEQRHQQEKRSNEKEHEEYQRRGRGQGHQP	-45.29	29.0	HHHHHHHHHHHHHHHHHHHHHHHHHCCCCCC
314	EEEERKRQDQEKNEKEQKRYQREGRGQGHQP	-45.25	32.3	HHHHHHHHHHHHHHHHHHHHHHHHCCCCCCC
214	EEEQRKQEQQRRNEKEQERYQRQGWGQGHYP	-45.25	29.0	HHHHHHHHHHHHHHHHHHHHHHHCCCCCCCC
129	QREKEKEEQQRSNEKEDEEYQRRGRGKGHQP	-45.24	29.0	CHHHHHHHHHHHHHHHHHHHHHHHCCCCCCC
063	EEEQRKQEQHRKNRKEDEEYQRRGRGKGHQP	-45.23	32.3	HHHHHHHHHHHHHHHHHHHHHHHHCCCCCCC
225	EEEKRKEEQQRKNEEEDQRYQRYGRGKGHQP	-45.21	35.5	HHHHHHHHHHHHHHHHHHHHHHHHCCCCCCC
234	EQEQRKQEEQKRNEEEQKRFQREGKGQGKNH	-45.20	22.6	HHHHHHHHHHHHHHHHHHHHHHHHCCCCCCC
064	EEEERKRQQQESNEREDRRYQREGRGKGHQP	-45.19	35.5	HHHHHHHHHHHHHHHHHHHHHHHHCCCCCCC
650	EQEERHKQEQYKNDYEDREYQRKGRGKGHQP	-45.14	29.0	HHHHHHHHHHHHHHHHHHHHHHHHCCCCCCC
178	EEEERKRQDDEKQRKEDEEYQRRGRGKGHQP	-45.13	35.5	HHHHHHHHHHHHHHHHHHHHHHHHCCCCCCC
660	EQEQRHQEEQRRNDKEHQRYQREGRGQGHQP	-45.12	22.6	HHHHHHHHHHHHHHHHHHHHHHHHCCCCCCC
600	EEEERKRQDQENNDREQERYQRQGRGQGHQP	-45.12	32.3	HHHHHHHHHHHHHHHHHHHHHHHHCCCCCCC
597	EREQRYEEDQKKNEQEHSRYQQQGKGQGHNH	-45.12	29.0	HHHHHHHHHHHHHHHHHHHHHHHCCCCCCCC
270	EEERKKQEDQERNEKEQQRYQREGKGQGHNH	-45.11	35.5	HHHHHHHHHHHHHHHHHHHHHHHHCCCCCCC
054	EEERKKQEDQYRNEYEQEEYQRKGRGQGHQP	-45.11	35.5	HHHHHHHHHHHHHHHHHHHHHHHHCCCCCCC
171	EEEKRKQEDHRKNREEQEKYQREGKGQGHNH	-45.08	29.0	HHHHHHHHHHHHHHHHHHHHHHHHCCCCCCC
695	EEEERKRRDQEKNEKEQEEYQRRGRGQGHQP	-45.07	32.3	HHHHHHHHHHHHHHHHHHHHHHHHHCCCCCC
561	EEEERKRRDQESNEKEQKEYQKQGRGQGHQP	-45.07	32.3	HHHHHHHHHHHHHHHHHHHHHHHHCCCCCCC
610	EQEERHKQEQWKNEWEDKYYQKKGQGKGHQS	-45.07	32.3	HHHHHHHHHHHHHHHHHHHHHHHHCCCCCCC
785	QQEQNKEEDDKRHRYEDEEYQRKGRGKGHQP	-45.03	32.3	CHHHHHHHHHHHHHHHHHHHHHHHCCCCCCC
150	DEERKKQHDQESNEKEQKRYQRYGQGQGHQS	-45.03	32.3	HHHHHHHCHHHHHHHHHHHHHHHHHCCCCCC
278	EEEERKKQDDRKNREEHQRYQREGWGRGHYP	-45.02	29.0	HHHHHHHHHHHHHHHHHHHHHHHHCCCCCCC

608	EQEERHKEEQRSNEKEDEEYQRRGRGKGHQP	-45.02	32.3	HHHHHHHHHHHHHHHHHHHHHHHHHCCCCCC
204	EQEERHKQEQYNNEYEDEEYQRKGRGKGHQP	-45.02	35.5	HHHHHHHHHHHHHHHHHHHHHHHHHCCCCCC
700	EEEERKQQDHRKNRYEQQRYQREGKGQGHSN	-45.01	29.0	HHHHHHHHHHHHHHHHHHHHHHHHCCCCCCC
293	EEEQRKEEDQKKNEKEQEEYQRRGQGQGHQP	-45.00	32.3	HHHHHHHHHHHHHHHHHHHHHHHHHHCCCCC
346	DNERRKQHEQESNDKEHKRYQREGKGQGHNH	-44.98	19.4	CHHHHHHHHHHHHHHHHHHHHHHHCCCCCCC
331	EEQRRKQEDQYRNDWEQHRYQNEGRGQGHQP	-44.97	32.3	HHHHHHHHHHHHHHHHHHHHHCCCCCCCCCC
694	EQEKRHQEEQREDEKRDEEYQRRGQGKGHQS	-44.96	29.0	HHHHHHHHHHHHHHHHHHHHHHHHHHCCCCC
441	EQQERHNQEQQKNDKEHQRYQREGKGQGHNH	-44.96	25.8	HHHHHHHHHHHHHHHHHHHHHHHHHCCCCCC
473	QEEKKKEEDQRKNEEEQKRYQREGRGQGHQP	-44.95	32.3	HHHHHHHHHHHHHHHHHHHHHHHHHCCCCCC
146	EQEQRKQEEQWRNEWEQQYYQKQGQGQGHQP	-44.95	25.8	HHHHHHHHHHHHHHHHHHHHHHHHHCCCCCC
176	EEERRKQEDQERQEKEQQEYQQRGQGQGHQS	-44.94	32.3	HHHHHHHHHHHHHHHHHHHHHHHHHHCCCCC
433	EEQQRKQKDHQDNRREQEEYQRRGKGQGHNN	-44.93	32.3	HHHHHHHHHHHHHHHHHHHHHHHHCCCCCCC
596	QQEQNHEEEKKSNEKEDEEYQRRGKGKGHNH	-44.92	32.3	CHHHHHHHHHHHHHHHHHHHHHHHHCCCCCC
312	EEEERRKEEQRRNDEEDRRYQRYGQGKGHQS	-44.88	32.3	HHHHHHHHHHHHHHHHHHHHHHHHHHCCCCC
837	EEEERKQQDDRENRREQQEYQREGKGQGHNH	-44.87	29.0	HHHHHHHHHHHHHHHHHHHHHHHHHCCCCCC
603	EEEERKKEQKYRNEWEQKRYQREGRGQGHQP	-44.87	32.3	HHHHHHHHHHHHHHHHHHHHHHHHHCCCCCC
203	EEEEKKKQDDEKNRKEDEQYQRYGRGKGHQP	-44.86	38.7	CHHHHHHHHHHHHHHHHHHHHHHHHHCCCCC
100	EEEQKKKEDDRKNREEDQRYQREGQGKGHQS	-44.86	38.7	HHHHHHHHHHHHHHHHHHHHHHHHHCCCCCC
918	EEEERKRQDQESNDKEQREYQKQGQGQGHQS	-44.83	29.0	HHHHHHHHHHHHHHHHHHHHHHHHHCCCCCC
093	EEEERKKQQQYSNDWEQEEFQRKGRGQGKQN	-44.82	22.6	HHHHHHHHHHHHHHHHHHHHHHHHHHCCCCC
008	EQEERHKQEQKSNEEEDKRYQRYGRGKGHQP	-44.82	32.3	HHHHHHHHHHHHHHHHHHHHHHHHHCCCCCC
684	EEEEKKKQDQQKNEKEQEEYQRRGRGQGHQP	-44.81	35.5	CHHHHHHHHHHHHHHHHHHHHHHHHCCCCCC
003	EEEERKQRQQESNEREQERYQKQGRGQGHQP	-44.80	29.0	HHHHHHHHHHHHHHHHHHHHHHHHCCCCCCC
329	EQQERHKEEQWRNEYEDEEYQRKGRGKGHQP	-44.77	35.5	CHHHHHHHHHHHHHHHHHHHHHHHHCCCCCC
239	QEEEKKQRDDEKHRKEDQRYQREGRGKGHQP	-44.73	38.7	CHHHHHHHHHHHHHHHHHHHHHHHHCCCCCC
509	EQQERKREDQWRNEYEHEEYQRKGRGQGHQP	-44.73	32.3	HHHHHHHHHHHHHHHHHHHHHHHHHCCCCCC
238	QQQQNHQEEQRRNEKEHQRYQREGRGQGHQP	-44.68	25.8	CHHHHHHHHHHHHHHHHHHHHHHHHCCCCCC
532	EQEERHRQEKEKNEKEDKRYQREGKGKGHNH	-44.68	35.5	HHHHHHHHHHHHHHHHHHHHHHHHCCCCCCC
934	QDEERKQRDQEKNEKEQEEYQRRGKGQGHSS	-44.64	25.8	CHHHHHHHHHHHHHHHHHHHHHHHHCCCCCC
994	EEERRKQEDQERNEKEQEEYQRYGWGQGHFP	-44.63	35.5	HHHHHHHHHHHHHHHHHHHHHHHHCCCCCCC
471	EQERRHQHEQESNDKEHEEYQRRGWGRGHYP	-44.63	22.6	HHHHHHHHHHHHHHHHHHHHHHHHCCCCCCC
923	ENEERRKREQYSNDWEDEEYQRKGRGKGHQP	-44.59	29.0	CHHHHHHHHHCCHHHHHHHHHHHHCCCCCCC
703	EQEERHRQEQEKNDKEHERYQRQGWGQGHYP	-44.59	22.6	HHHHHHHHHHHHHHHHHHHHHHHHCCCCCCC
456	EQEERKKQEQYSNRYEQEEYQRKGRGQGHQP	-44.54	22.6	HHHHHHHHHHHHHHHHHHHHHHHHCCCCCCC
367	EEEERKKRDQWKNEWEQKYYQQKGQGQGHQP	-44.44	32.3	HHHHHHHHHHHHHHHHHHHHHHHHCCCCCCC
438	EEEEKKKQEKEKNEKEHKRYQREGRGQGHQP	-44.43	35.5	HHHHHHHHHHHHHHHHHHHHHHHHHCCCCCC
251	EEEERKKEDDRKNREEHERYQQQGKGQGHNN	-44.39	29.0	CHHHHHHHHHHHHHHHHHHHHHHHCCCCCCC
244	EQEERKKEDDWKNRWEHEYYQRKGQGQGHQP	-44.39	25.8	HHHHHHHHHHHHHHHHHHHHHHHHHCCCCCC
333	QQEENHRQEQEKNRKEDERYQREGKGKGHNN	-44.37	25.8	CHHHHHHHHHHHHHHHHHHHHHHHCCCCCCC
540	EEERRRQQEQQSNDKEDEEYQRYGRGKGHQP	-44.37	32.3	HHHHHHHHHHHCCHHHHHHHHHHHHCCCCCC
067	EEEQKKQEQQKRNEEEQKRYQQYGKGQGHNN	-44.31	32.3	HHHHHHHHHHHHHHHHHHHHHHHHHCCCCCC
873	EQEKRHNQEKYSNEWEQEEYQRQGRGQGHQP	-44.31	29.0	HHHHHHHHHHHHHHHHHHHHHHHHHCCCCCC
446	EEEERKQQDDRSNRKEQQEYQRSGKGQGHNN	-44.28	32.3	HHHHHHHHHHHHHHHHHHHHHHHHCCCCCCC
720	EEEQRKEEQQKKNEQEDQRYQRYGRGKGNQN	-44.27	35.5	HHHHHHHHHHHHHHHHHHHHHHHHCCCCCCC
242	EEEERKQQQQREDDWRQEYYQKYGQGQGHQP	-44.17	22.6	HHHHHHHHHHHHHHHHHHHHHHHHHHCCCCC
306	EEERKKQRDQESNEKEQEKYQQEGKGQGHNH	-44.16	35.5	HHHHHHHHHHHHHHHHHHHHHHHHHCCCCCC
499	EEEERKREQQERNDKEHEEYQRYGRGQGHQP	-44.13	25.8	HHHHHHHHHHHHHHHHHHHHHHHHHHCCCCC
958	EQEERHKEEQWRNDYEHEEYQRKGRGQGHQP	-44.13	22.6	HHHHHHHHHHHHHHHHHHHHHHHHHCCCCCC
885	QQEENHKEEQRSNDKEHEEYQRRGRGQGHQP	-44.13	19.4	CHHHHHHHHHHHHHHHHHHHHHHHHHCCCCC
667	EEEERKKKQQEKDEKRDEEYQRRGRGKGHQP	-44.12	32.3	CHHHHHHHHHHHHHHHHHHHHHHHHCCCCCC
775	EEEERKKQQVQKNRKEHEEYQRRGRGQGHQP	-44.12	25.8	HHHHHHHHHHHHHHHHHHHHHHHHHCCCCCC
449	EQEERHRQEQEKNEKEHKRYQREGKGRGHNN	-44.08	25.8	HHHHHHHHHHHHHHHHHHHHHHHHCCCCCCC
965	EQEERHKQEQIKNEKEDEEYQRRGRGKGHQP	-43.94	32.3	HHHHHHHHHHHHHHHHHHHHHHHHHCCCCCC
383	EQEERHKQEQYKNEWEDKRYQREGRGKGHQP	-43.94	32.3	HHHHHHHHHHHHHHHHHHHHHHHHHCCCCCC
862	QQQENHWREQESNDREDRRYQREGQGKGHQS	-43.94	29.0	CHHHHHHHHHHCCHHHHHHHHHHHCCCCCCC
861	ENEERRKEEQKRNDKEHEEYQRRGRGQGHQP	-43.91	22.6	CHHHHHHHHHHHHHHHHHHHHHHHHHCCCCC
185	EQEERHRREQESNRKEDERYQREGRGKGHQP	-43.89	29.0	HHHHHHHHHHHHHHHHHHHHHHHHHCCCCCC
943	QQEQNKEEEQKEEEKRHEEKQRRGKGCGKNN	-43.88	16.1	CHHHHHHHHHHHHHHHHHHHHHHHCCCCCCC
589	EEEERKKQEDQSNRREDEEYQRRGRGKGHQP	-43.87	32.3	HHHHHHHHHHHHHHHHHHHHHHHHHCCCCCC
260	EEQEKKKRDQYKEEWRHEEYQRKGRGQGHQP	-43.86	35.5	HHHHHHHHHHHHHHHHHHHHHHHHCCCCCCC
697	EEEERKQQQKRDQENRQKRYQKEGWGQGHYP	-43.82	29.0	HHHHHHHHHHHHHHHHHHHHHHHHCCCCCCC
619	EQERRHQEEQRSNDEEDRRYQREGRGKGHQP	-43.78	29.0	HHHHHHHHHHHCHHHHHHHHHHHHCCCCCCC
952	QQERNHQEEQRSNDEEDRRYQREGRGKGHQP	-43.78	25.8	CHHHHHHHHHHCHHHHHHHHHHHHCCCCCCC
156	EQERRHQEEQERNRKEDERYQREGQGKGHQS	-43.74	29.0	HHHHHHHHHHHHHHHHHHHHHHHHHCCCCCC
754	EQEERHKQEQYKFDWWHEEYQRKGRGQGHQP	-43.73	19.4	CHHHHHHHHHHHHHHHHHHHHHHHCCCCCCC
842	EQEKRHQEEQRNNQEEQDRYQREGKGQGHSH	-43.73	25.8	HHHHHHHHHHHHHHHHHHHHHHHCCCCCCCC
490	EQEQRKEEEQKKNEYEQEEYQRKGRGQGHQP	-43.71	25.8	HHHHHHHHHHHHHHHHHHHHHHHHHCCCCCC

027	EQEQRHQEEKRRNEKEQEEYQRKGRGQGHQP	-43.69	29.0	HHHHHHHHHHHHHHHHHHHHHHHHCCCCCCC
654	QQQENHQQEQQKNEKEQEEYQRRGKGQGHQH	-43.68	25.8	CHHHHHHHHHHHHHHHHHHHHHHHHCCCCCC
488	QWERWLQELQERNDKEQQRYQKQGRGQGHQP	-43.62	19.4	CHHHHHHHHHHHHHHHHHHHHHHHCCCCCCC
618	EQEQRHQEEQRKNEREQEEYQQRGRGQGHQP	-43.61	25.8	HHHHHHHHHHHHHHHHHHHHHHHHHCCCCCC
478	EQERRHQEEQRRNEQEQKRYQREGRGQGHNP	-43.61	25.8	HHHHHHHHHHHHHHHHHHHHHHHHCCCCCCC
841	NDEERKRQDDEKNRKEDERYQRQGRGKGNQN	-43.50	29.0	CHHHHHHHHHHHHHHHHHHHHHHCCCCCCCC
335	EQQERHQREQWKNEWEQKYYQKEGQGQGHQS	-43.49	29.0	CHHHHHHHHHHHHHHHHHHHHHHHCCCCCCC
445	EQEERHREEQYRNEWEDEEYQRKGRGKGHSN	-43.48	32.3	HHHHHHHHHHHHHHHHHHHHHHHCCCCCCCC
732	EQEERHRQEQESNEREDKRYQREGRGKGHQP	-43.48	32.3	HHHHHHHHHHHHHHHHHHHHHHHHCCCCCCC
272	EQEKREQEKQRSEEEEQEEYQRKGRGQGHNP	-43.46	25.8	HHHHHHHHHHHHHHHHHHHHHHHHCCCCCCC
320	EQQQRHQQEDRSNRQEDQRYQRYGRGKGHQP	-43.44	32.3	HHHHHHHHHHHHHHHHHHHHHHHHCCCCCCC
758	EEQERKKEDDWRNRWEQEYYQRKGQGQGHQP	-43.44	32.3	HHHHHHHHHHHHHHHHHHHHHHHHCCCCCCC
277	EQEQRHQEEQRRNDKEQRRYQRYGRGQGHQP	-43.42	22.6	HHHHHHHHHHHHHHHHHHHHHHHHHCCCCCC
761	EQEERHQQEQRKNEKEQKRYQREGRGQGHQP	-43.42	25.8	CHHHHHHHHHHHHHHHHHHHHHHHCCCCCCC
082	EEEERKQRQQESNDKEQKRYQREGWGQGHYP	-43.40	25.8	HHHHHHHHHHHHHHHHHHHHHHHHCCCCCCC
969	DNERRKHEEDNKNRKEDEEYQRYGQGKGHQS	-43.39	25.8	CHHHHHHHHHHHHHHHHHHHHHHHHCCCCCC
053	EQQERHKEEKQRNEKEQKRYQRYGRGQGHQP	-43.38	32.3	CHHHHHHHHHHHHHHHHHHHHHHHHCCCCCC
300	EEQERKKQDDYKNRWEQEYYQQYGQGQGHQP	-43.38	32.3	HHHHHHHHHHHHHHHHHHHHHHHHHCCCCCC
308	EQEERHKEEQRSNDEEHRYYQKYGRGQGHQP	-43.37	22.6	HHHHHHHHHHHHHHHHHHHHHHHHCCCCCCC
665	EQEERHKEEQWRNRWEHEYYQKKGQGQGHNH	-43.37	22.6	HHHHHHHHHHHHHHHHHHHHHHHHCCCCCCC
328	EQEERKKQEKYKNEYEQEEYQRKGRGQGNQN	-43.35	29.0	HHHHHHHHHHHHHHHHHHHHHHHHCCCCCCC
806	EQERRKQEEQRSNEEEQQRYQREGWGQGHYP	-43.34	25.8	HHHHHHHHHHHHHHHHHHHHHHHHCCCCCCC
704	EQQQRHQEEQRRNEKEQEEYQRRGWGQGHYP	-43.27	29.0	HHHHHHHHHHHHHHHHHHHHHHHCCCCCCCC
765	QQQENHQREQESNEKEDKRYQKEGRGKGHQP	-43.25	32.3	CHHHHHHHHHHHHHHHHHHHHHHCCCCCCCC
363	EQEQRHEEEQKSNDWEHKYYQQKGQGQGHQP	-43.21	22.6	HHHHHHHHHHHHHHHHHHHHHHHHCCCCCCC
285	EEQERKQRDDQKNRREQERYQRQGRGQGHQP	-43.13	32.3	HHHHHHHHHHHHHHHHHHHHHHHHCCCCCCC
291	EQEERHQQEDRSNRQEDEQYQRYGKGKGHNN	-43.12	29.0	CHHHHHHHHHHHHHHHHHHHHHHHCCCCCCC
292	EEEERRRQEKEKNEKEDRRYQRYGRGKGHQP	-43.11	38.7	HHHHHHHHHHHHHHHHHHHHHHHHCCCCCCC
849	QQQENHRQEQQSNEWEDKYYQKKGQGKGHQP	-43.04	32.3	CHHHHHHHHHHHHHHHHHHHHHHHCCCCCCC
869	EEEERRQQEDRKNRHEDERYQRQGRGKGHQP	-42.90	32.3	HHHHHHHHHHHHHHHHHHHHHHHHCCCCCCC
157	EQERRHQEEQERNDKEHEEYQRRGKGQGHNN	-42.82	22.6	HHHHHHHHHHHHHHHHHHHHHHHCCCCCCCC
828	EQEERHQREQESNDKEHKRYQRYGRGQGHQP	-42.75	22.6	CHHHHHHHHHHHHHHHHHHHHHHHCCCCCCC
730	EEEERKRQDDESNRKEHEEYQRRGQGQGHQS	-42.68	29.0	CHHHHHHHHHHHHHHHHHHHHHHHHCCCCCC
846	QQEENHHQEQNKNDQEDRRYQRYGQGKGNQN	-42.67	25.8	CHHHHHHHHHHHCHHHHHHHHHHCCCCCCCC
580	EQQQRHNEEQRSNDKEQREYQRRGRGQGHQP	-42.59	25.8	HHHHHHHHHHHHCHHHHHHHHHHHCCCCCCC
268	EQEERKRQEQESNEKEQKEYQRSGKGQGHNN	-42.58	29.0	HHHHHHHHHHHHHHHHHHHHHHHCCCCCCCC
762	NQERQKQEEKREDEERQKSYQREGKGQGHNN	-42.39	22.6	CHHHHHHHHHHHHHHHHHHHHHHCCCCCCCC
915	EQQRRHQEEKYRNEYEHEEYQRKGRGQGHQP	-42.38	32.3	HHHHHHHHHHHHHHHHHHHHHHHHCCCCCCC
002	EQQERKRQQQEKNEKEQEEYQRRGKGQGHNH	-42.18	29.0	HHHHHHHHHHHHHHHHHHHHHHHHCCCCCCC
512	EQEERHREEQREEDERHRRKQKEGRGCGKQN	-41.94	16.1	HHHHHHHHHHHHHHHHHHHHHHHCCCCCCCC
534	EQERRHQEEQERNDKEQQKYQKEGRGQGHQP	-41.88	22.6	HHHHHHHHHHHHHHHHHHHHHHHHCCCCCCC
295	EQEERHNQEQQKNEREQKRYQREGKGQGHNN	-41.83	25.8	HHHHHHHHHHHHHHHHHHHHHHHCCCCCCCC
397	EEQERKKQEDQSNRREQERYQQQGRGQGHQP	-41.72	29.0	HHHHHHHHHHHHHHHHHHHHHHHHCCCCCCC
677	EEEERKKQQQKEDEERHKRYQRYGRGQGNQN	-41.71	25.8	HHHHHHHHHHHHHHHHHHHHHHHHCCCCCCC
442	QQQENHKEEDYRNRWEDQRYQREGRGKGHQP	-41.62	29.0	CHHHHHHHHHHHHHHHHHHHHHHHCCCCCCC
218	QQEENHQQEQERNRKEDERYQREGWGKGHYP	-41.52	25.8	CHHHHHHHHHHHHHHHHHHHHHHHCCCCCCC
836	EEQRRKQEQQYRDDFRQRMYQQYGKGQGHQP	-41.46	25.8	HHHHHHHHHHHHHHHHHHHHHHHHHCCCCCC
173	EQEERHRQEQEKNEKEQEEYQRQGRGQGHQP	-41.25	25.8	HHHHHHHHHHHHHHHHHHHHHHHHCCCCCCC
690	EQERRHQEEQQKNDREQRRYQREGRGQGHQP	-41.23	22.6	HHHHHHHHHHHHHHHHHHHHHHHHCCCCCCC
466	EQERRHQEEQERNEKEQEEYQRRGRGQGHQP	-41.11	25.8	HHHHHHHHHHHHHHHHHHHHHHHHHCCCCCC
467	QQEENHREEQERNEKEQEEYQRRGRGQGHQP	-40.82	22.6	CHHHHHHHHHHHHHHHHHHHHHHHHCCCCCC
127	EQQERHRQEQEKNEKEQKRYQREGQGQGHQS	-40.81	29.0	HHHHHHHHHHHHHHHHHHHHHHHHCCCCCCC
199	EQEERHQREQEEDDKRHRRYQREGKGQGHNN	-40.81	19.4	CHHHHHHHHHHHHHHHHHHHHHHCCCCCCCC
922	EQEQRHEEEQRKNEKEQEEYQRYGRGQGHQP	-40.72	25.8	HHHHHHHHHHHHHHHHHHHHHHHHHCCCCCC
747	QQEENHKQEDYKNRYEDEEYQRKGRGKGHQP	-40.71	25.8	CHHHHHHHHHHHHHHHHHHHHHHHCCCCCCC
640	EQQRRHQEEQYRNEYEQEEYQRKGRGQGHQP	-40.67	29.0	HHHHHHHHHHHHHHHHHHHHHHHHCCCCCCC
851	EQERRHQEEVWRNRYEQEEYQRKGRGQGHQP	-40.49	22.6	HHHHHHHHHHHHHHHHHHHHHHHHCCCCCCC
517	EQEERHRQEQESNEREQQRYQREGRGQGHQP	-40.35	25.8	HHHHHHHHHHHHHHHHHHHHHHHHCCCCCCC
028	EQQQRHNEEHRKNREEQEEYQRKGRGQGHNP	-39.86	25.8	HHHHHHHHHHHHHHHHHHHHHHHHCCCCCCC
221	EEERRKQEQDESNRKEQERYQKQGRGQGHQP	-39.48	25.8	HHHHHHHHHHHHHHHHHHHHHHHCCCCCCCC
307	EQEERHKQEDQKNRREHHEYQNRGRGQGNQN	-38.05	22.6	HHHHHHHHHHHHHHHHHHHHHHCCCCCCCCC

Table S2. Summary of 991 peptide sequences designed using EvoEF2 and the evolutionary profile (weight = 0.25).

#label	#peptide	#binding(EEU)	#SeqID(%)	#Secondary structure
WT	EEQAKTFLDKFNHEAEDLFYQSSGLGKGDFR	-46.46	100.	HHHHHHHHHHHHHHHHHHHHHHHCCCCCCCC
185	EQEQRIQQDQFSNEFEDKYYQQKGQGKGHSH	-52.40	38.7	HHHHHHHHHHHHHHHHHHHHHHHHCCCCCCC
416	EEERRKQLDQKKQDEEDSRFQQQGRGKGKQN	-51.50	35.5	HHHHHHHHHHHHHHHHHHHHHHHHCCCCCCC
731	EEEQRKQQDKYNQESEDKRYQRYGWGKGHNP	-51.43	45.2	HHHHHHHHHHHHHHHHHHHHHHHCCCCCCCC
208	EEQQRKQQDKYNNESEDKRFQRYGKGKGKSR	-51.41	48.4	HHHHHHHHHHHCHHHHHHHHHHHCCCCCCCC
148	EEEQKKQQDKDNNEREDKRYQREGQGKGHQS	-51.13	48.4	HHHHHHHHHHHHHHHHHHHHHHHCCCCCCCC
765	EEEQKKQQDKYNNEWEDKKYQREGRGKGHQP	-51.13	48.4	HHHHHHHHHHHHHHHHHHHHHHHHCCCCCCC
859	EEEQKKQQDKYNNEYEDKQYQKEGWGKGHYP	-51.13	48.4	HHHHHHHHHHHHHHHHHHHHHHHHCCCCCCC
840	EEEQKKQRDKYNNEHEDKRYQQQGKGKGHNH	-51.13	48.4	HHHHHHHHHHHHHHHHHHHHHHHCCCCCCCC
350	EEEQKKQRDKYNNESEDKRYQRYGWGKGHNP	-51.13	48.4	HHHHHHHHHHHCHHHHHHHHHHCCCCCCCCC
666	EEEQRKQQDKKNNEEEDKRYQRYGKGKGHSN	-51.13	45.2	HHHHHHHHHHHHHHHHHHHHHHHCCCCCCCC
725	EEEQRKQQDKYNNEEEDKRYQREGWGKGHNP	-51.13	45.2	HHHHHHHHHHHCHHHHHHHHHHHHCCCCCCC
830	EEEQRKQQDKYNNEYEDEEYQRKGRGKGHQP	-51.13	45.2	HHHHHHHHHHHHHHHHHHHHHHHHCCCCCCC
119	EEEQRKQQDKYNNEYEDKEYQRKGRGKGHQP	-51.13	45.2	HHHHHHHHHHHHHHHHHHHHHHHHCCCCCCC
835	EQEQRKQQDKYNNEYEDKEYQRKGRGKGHQP	-51.13	41.9	HHHHHHHHHHHHHHHHHHHHHHHHCCCCCCC
609	EEEQRKQEDKRNNEQEDKRYQRYGRGKGHQP	-51.13	45.2	HHHHHHHHHHHHHHHHHHHHHHHHCCCCCCC
697	EEEEKKQQDKFKNEFEDKYYQQKGQGKGHNH	-51.00	48.4	CHHHHHHHHHHHHHHHHHHHHHHHCCCCCCC
791	EEEERKQRDKFNNEYEDKYYQQKGQGKGHQS	-51.00	48.4	HHHHHHHHHHHHHHHHHHHHHHHHCCCCCCC
342	EEEERKQRDKYNNEAEDKRYQREGRGKGHQP	-51.00	48.4	HHHHHHHHHHHHHHHHHHHHHHHHCCCCCCC
359	EEEERKQRDKYNNEYEDKQYQKEGRGKGHQP	-51.00	45.2	HHHHHHHHHHHHHHHHHHHHHHHHCCCCCCC
499	EEERKKQLDKWKNEFEDKYYQQQGQGKGHQS	-51.00	48.4	HHHHHHHHHHHHHHHHHHHHHHHHCCCCCCC
659	EEERRKQEDKYRNEWEDEEYQRKGRGKGHQP	-51.00	41.9	HHHHHHHHHHHHHHHHHHHHHHHHCCCCCCC
393	EEERRKQLDKFKNEFEDKYYQQQGQGKGHSS	-51.00	48.4	HHHHHHHHHHHHHHHHHHHHHHHHCCCCCCC
453	EEERRKQLDKQSNEEEDKRYQRYGQGKGHQS	-51.00	45.2	HHHHHHHHHHHHHHHHHHHHHHHHHCCCCCC
368	EEERRKQLDKWKNEFEDKRYQQEGRGKGHQP	-51.00	45.2	HHHHHHHHHHHHHHHHHHHHHHHHCCCCCCC
085	EEERRKQLDKYKNEAEDKRYQRYGKGKGHSN	-51.00	48.4	HHHHHHHHHHHHHHHHHHHHHHHCCCCCCCC
384	EEERRKQLDKYNNEAEDKRYQREGQGKGHQS	-51.00	51.6	HHHHHHHHHHHHHHHHHHHHHHHHCCCCCCC
683	EEEKRKQEDKRRHEKEDEEYQRRGWGKGHNP	-50.98	45.2	HHHHHHHHHHHHHHHHHHHHHHHCCCCCCCC
805	EEEERKQQDKRNNEKEDKRYQRYGWGKGHYP	-50.94	45.2	HHHHHHHHHHHHHHHHHHHHHHHCCCCCCCC
959	DEERKKQEDQYRNDWEDEEYQRKGRGKGHQP	-50.89	35.5	HHHHHHHHHHHHHHHHHHHHHHHHCCCCCCC
186	EEEERKQRDQMKNDKEDKEYQKQGRGKGHQP	-50.89	35.5	HHHHHHHHHHHHHHHHHHHHHHHHCCCCCCC
471	EQEERKNQDKYNNEYEDEEYQRKGRGKGHNP	-50.83	41.9	HHHHHHHHHHHHHHHHHHHHHHHCCCCCCCC
726	EEEKRKQEDKRNNEEEDKRYQREGWGKGHNP	-50.81	45.2	HHHHHHHHHHHHHHHHHHHHHHHHCCCCCCC
321	EEEKRKQLDKYKNEAEDKRYQQQGWGKGHNP	-50.81	48.4	HHHHHHHHHHHHHHHHHHHHHHHCCCCCCCC
279	EEEQRKQQDKYNNEYEDKMYQREGKGKGHSH	-50.74	45.2	HHHHHHHHHHHHHHHHHHHHHHHCCCCCCCC
407	EEERRKQLDQESQEKEDEQYQRYGMGKGHQP	-50.54	41.9	HHHHHHHHHHHHHHHHHHHHHHHHHCCCCCC
669	EEEQRKQQDQYNNDWEDQRYQREGRGKGHQP	-50.50	38.7	HHHHHHHHHHHHHHHHHHHHHHHHCCCCCCC
793	EEEQKKQRDQYNNDSEDRRFQREGKGKGKNH	-50.49	38.7	HHHHHHHHHHCCCCHHHHHHHHHCCCCCCCC
087	EEEQRKQQDQYNQEWEDEYYQRKGQGKGHQP	-50.48	41.9	HHHHHHHHHHHHHHHHHHHHHHHHCCCCCCC
909	EEEQRKQQDQYNQEWEDEKYQREGKGKGHSS	-50.48	41.9	HHHHHHHHHHHHHHHHHHHHHHHCCCCCCCC
467	EEEQRKQQDQYNQEYEDEEYQRKGRGKGHQP	-50.48	41.9	HHHHHHHHHHHHHHHHHHHHHHHHCCCCCCC
943	EEEERKQQDQRNQEKEDERYQQQGRGKGHQP	-50.48	41.9	HHHHHHHHHHHHHHHHHHHHHHHHCCCCCCC
993	EEEQRKQQDQQNNEREDEYYQQQGRGKGHQP	-50.37	41.9	HHHHHHHHHHHHHHHHHHHHHHHHCCCCCCC
658	EEEQKKQRDQFNNEFEHEYYQQKGQGRGHNH	-50.35	41.9	HHHHHHHHHHHHHHHHHHHHHHHHCCCCCCC
962	EEEQKKQQDKYNNESEDKRYQRYGRGKGHQP	-50.33	48.4	HHHHHHHHHHHCHHHHHHHHHHHHCCCCCCC
296	EEEQKKQRDKYNNEYEDEQYQRKGKGKGHNN	-50.33	48.4	HHHHHHHHHHHHHHHHHHHHHHHCCCCCCCC
102	EEEQRKQQDKYNNEWEDEEYQRKGRGKGHSN	-50.33	45.2	HHHHHHHHHHHHHHHHHHHHHHHCCCCCCCC
438	EEEERKKQDKQNNEKEDKRFQREGQGKGKQS	-50.31	41.9	HHHHHHHHHHHHHHHHHHHHHHHHCCCCCCC
526	EEEQKKQRDQRNNEQEDKRYQRYGRGKGHQP	-50.31	45.2	HHHHHHHHHHHHHHHHHHHHHHHHCCCCCCC
677	EEEQRKQEDQRKNEKEDKRYQREGKGKGHSH	-50.31	38.7	HHHHHHHHHHHHHHHHHHHHHHHCCCCCCCC
893	EEERRKQLDQKNNDEEHRRYQRYGWGRGHNP	-50.29	35.5	HHHHHHHHHHHCHHHHHHHHHHHCCCCCCCC
776	EEEQRKQLDKKNNEEEDKRYQREGKGKGHSH	-50.29	48.4	HHHHHHHHHHHHHHHHHHHHHHHCCCCCCCC
966	EEEEKKQQDQRKQEEEDERYQQQGRGKGHQP	-50.29	41.9	CHHHHHHHHHHHHHHHHHHHHHHHCCCCCCC
429	EEQQRKQEDQYRNDHEDRKYQQEGRGKGHQP	-50.26	38.7	HHHHHHHHHHHHHHHHHHHHHHHHCCCCCCC
343	EEEQRKQQDQRNQRQEDERYQREGKGKGHNH	-50.26	38.7	HHHHHHHHHHHHHHHHHHHHHHHCCCCCCCC
808	EEEQKKQRDQYNNDWEDRRYQREGKGKGHSS	-50.25	41.9	HHHHHHHHHHHHCHHHHHHHHHCCCCCCCCC
377	EEEERKKQDKYKNESEDKRYQQQGKGKGHNH	-50.25	41.9	HHHHHHHHHHHHHHHHHHHHHHHHCCCCCCC
380	EEERKKQLDQQKNEREDSRYQQQGKGKGHSN	-50.24	45.2	HHHHHHHHHHHHHHHHHHHHHHHCCCCCCCC
614	EEERRKQLDQENNEKEDQRYQREGKGKGHNH	-50.24	45.2	HHHHHHHHHHHHHHHHHHHHHHHCCCCCCCC
202	EEEQKKQRDQRNQEKEDEEYQRYGWGKGHYP	-50.22	45.2	HHHHHHHHHHHHHHHHHHHHHHHCCCCCCCC
442	EEEQKKQRDQYNNRYEDEEYQRKGRGKGHQP	-50.22	41.9	HHHHHHHHHHHHHHHHHHHHHHHHCCCCCCC
918	EQQQRKQQDQYNNESEDQRYQRYGWGKGHNP	-50.18	41.9	HHHHHHHHHHHCHCHHHHHHHHCCCCCCCCC
328	EEEERKNRDQYNQEWEDEEYQRKGRGKGHQP	-50.18	41.9	HHHHHHHHHHHHHHHHHHHHHHHHCCCCCCC
092	EEEQKKQRDQFNNEEEDKRYQREGRGKGHQP	-50.18	48.4	HHHHHHHHHHHCHHHHHHHHHHHHCCCCCCC

530	EEEQKKQRDQYNNEAEDERYQRQGNGKGHSH	-50.18	48.4	HHHHHHHHHHHHHHHHHHHHHHHCCCCCCCC
262	EEEQKKQRDQYNNEAEDKRYQRYGWGKGHNP	-50.18	48.4	HHHHHHHHHHHHHHHHHHHHHHCCCCCCCCC
820	EEEQKKQRDQYNNEWEDEEYQRKGRGKGHQP	-50.18	45.2	HHHHHHHHHHHHHHHHHHHHHHHHCCCCCCC
678	EEEQKKQRDQYNNEWEDERYQRQGWGKGHYP	-50.18	45.2	HHHHHHHHHHHHHHHHHHHHHHHHCCCCCCC
418	EEEQKKQRDQYNNEYEDEEYQRKGRGKGHNP	-50.18	45.2	HHHHHHHHHHHHHHHHHHHHHHHCCCCCCCC
130	EEEQKKQRDQYNNEYEDQEYQRTGRGKGHQP	-50.18	45.2	HHHHHHHHHHHHHHHHHHHHHHCCCCCCCCC
914	EEEQRKQEDQYRNEYEDQEYQRKGRGKGHQP	-50.18	38.7	HHHHHHHHHHHHHHHHHHHHHHHHCCCCCCC
014	EEEQRKQQDQKNNEEEDERYQQQGWGKGHYP	-50.18	41.9	HHHHHHHHHHHHHHHHHHHHHHHCCCCCCCC
802	EEEQRKQQDQKNNEEEDKRYQRYGRGKGHQP	-50.18	41.9	HHHHHHHHHHHHHHHHHHHHHHHHCCCCCCC
487	EEEQRKQQDQYNNEAEDKRYQRYGRGKGHQP	-50.18	45.2	HHHHHHHHHHHHHHHHHHHHHHHHCCCCCCC
144	EEEQRKQQDQYNNEAEDQRYQQQGRGKGHQP	-50.18	45.2	HHHHHHHHHHHHHHHHHHHHHHHHCCCCCCC
055	EEEQRKQQDQYNNEAEDQRYQREGKGKGHNH	-50.18	45.2	HHHHHHHHHHHHHHHHHHHHHHHHCCCCCCC
861	EEEQRKQQDQYNNEFEDQMYQRYGKGKGHQP	-50.18	41.9	HHHHHHHHHHHHHHHHHHHHHHHHHCCCCCC
941	EEEQRKQQDQYNNESEDQRYQQYGWGKGHYP	-50.18	41.9	HHHHHHHHHHHCHCHHHHHHHHHCCCCCCCC
196	EEEQRKQQDQYNNEWEDKRYQREGRGKGHQP	-50.18	41.9	HHHHHHHHHHHHHHHHHHHHHHHHCCCCCCC
767	EEEQRKQQDQYNNEYEDEEYQRKGRGKGHQP	-50.18	41.9	HHHHHHHHHHHHHHHHHHHHHHHHCCCCCCC
833	EEEQRKQQDQYNNEYEDKYYQKQGQGKGHQS	-50.18	41.9	HHHHHHHHHHHHHHHHHHHHHHHHCCCCCCC
829	EEEQRKQQDQYNNEYEDQQYQKEGQGKGHQS	-50.18	41.9	HHHHHHHHHHHHHHHHHHHHHHHHCCCCCCC
040	EEEQRKQQDQYSNEYEDEEYQRKGRGKGHQP	-50.18	38.7	HHHHHHHHHHHHHHHHHHHHHHHHCCCCCCC
382	EEEQRKQQDQYSNEYEDKEYQKEGKGKGHNH	-50.18	38.7	HHHHHHHHHHHHHHHHHHHHHHHCCCCCCCC
379	EEEERKQQDQRSNEKEDEEYQRRGKGKGHNH	-50.18	38.7	HHHHHHHHHHHHHHHHHHHHHHHHCCCCCCC
929	EEEERKQQDQRSNEQEDQRYQREGKGKGHSS	-50.18	38.7	HHHHHHHHHHHHHHHHHHHHHHHCCCCCCCC
167	EQEQRKQEDQRKNEKEDKRYQREGKGKGHSH	-50.18	35.5	HHHHHHHHHHHHHHHHHHHHHHHCCCCCCCC
063	EEEARKQQDQYNQEWEDQRYQREGKGKGHNH	-50.16	45.2	HHHHHHHHHHHHHHHHHHHHHHHHCCCCCCC
094	DEERKKQEDQYRNDYEDREYQRKGRGKGHQP	-50.12	35.5	HHHHHHHHHHHHHHHHHHHHHHHHCCCCCCC
509	EEERRKQLDQMKNDKEDREYQRSGQGKGHQS	-50.12	41.9	HHHHHHHHHHHHHHHHHHHHHHHCCCCCCCC
173	EEERRKQLDQWKNDYEDRYYQQQGQGKGHQP	-50.12	38.7	HHHHHHHHHHHHHHHHHHHHHHHHCCCCCCC
632	EEERRKQQDQYSNDYEDREYQRKGRGKGHQP	-50.12	35.5	HHHHHHHHHHHCCHHHHHHHHHHCCCCCCCC
657	EQEERKQRDQYNNEAEDQRFQREGKGKGKNH	-50.10	38.7	HHHHHHHHHHHHHHHHHHHHHHHHCCCCCCC
934	EEEKKKQEDQRNNREEDERYQREGKGKGHSS	-50.09	41.9	HHHHHHHHHHHHHHHHHHHHHHHCCCCCCCC
860	EEEERKQQDQRKNRKEDEEYQKQGRGKGHQP	-50.06	35.5	HHHHHHHHHHHHHHHHHHHHHHHCCCCCCCC
690	EQEERKQQDQRKNEQEDEEYQRKGRGKGHQP	-50.05	35.5	HHHHHHHHHHHHHHHHHHHHHHHHCCCCCCC
336	DEERKKQEDQYRNEWEDEEYQRKGRGKGHNP	-50.05	38.7	HHHHHHHHHHHHHHHHHHHHHHHCCCCCCCC
243	EEEERKQQDQYNNESEDKRYQRYGWGKGHNP	-50.05	41.9	HHHHHHHHHHHCHHHHHHHHHHCCCCCCCCC
968	EEEERKQRDQFNNEFEDQYYQRYGQGKGHNP	-50.05	45.2	HHHHHHHHHHHHHHHHHHHHHHHHHCCCCCC
383	EEEERKQRDQQSNEEEDQRYQRYGWGKGHNP	-50.05	38.7	HHHHHHHHHHHHHHHHHHHHHHHCCCCCCCC
661	EEEERKQRDQYNNEAEDKRYQREGKGKGHSS	-50.05	45.2	HHHHHHHHHHHHHHHHHHHHHHHCCCCCCCC
149	EEEERKQRDQYNNEYEDEEYQRKGRGKGHQP	-50.05	41.9	HHHHHHHHHHHHHHHHHHHHHHHHCCCCCCC
203	EEEKKKQEDQRNNEEEDKRYQREGRGKGHQP	-50.05	45.2	HHHHHHHHHHHHHHHHHHHHHHHHCCCCCCC
994	EEEKRKQEDQRNNEEEDERYQRYGWGKGHYP	-50.05	41.9	HHHHHHHHHHHHHHHHHHHHHHHCCCCCCCC
054	EEEKRKQEDQRNNEEEDKRYQREGKGKGHNN	-50.05	41.9	HHHHHHHHHHHHHHHHHHHHHHHCCCCCCCC
200	EEERKKQLDQQNNEEEDKRYQRYGKGKGHSS	-50.05	48.4	HHHHHHHHHHHHHHHHHHHHHHHCCCCCCCC
713	EEERKKQLDQYNNEWEDEEYQRKGRGKGHQP	-50.05	48.4	HHHHHHHHHHHHHHHHHHHHHHHHCCCCCCC
583	EEERKKQLDQYNNEYEDEYYQQKGQGKGHSH	-50.05	48.4	HHHHHHHHHHHHHHHHHHHHHHHHCCCCCCC
058	EEERRKQLDQKNNEEEDQRYQKQGWGKGHNP	-50.05	45.2	HHHHHHHHHHHHHHHHHHHHHHHCCCCCCCC
596	EEERRKQLDQQSNEEEDSRYQQQGKGKGHSN	-50.05	41.9	HHHHHHHHHHHHHHHHHHHHHHHCCCCCCCC
366	EEERRKQLDQYNNEEEDKRYQREGKGKGHNH	-50.05	45.2	HHHHHHHHHHHCCHHHHHHHHHHHCCCCCCC
510	EEERRKQLDQYNNEYEDEEYQRKGRGKGHQP	-50.05	45.2	HHHHHHHHHHHHHHHHHHHHHHHHCCCCCCC
740	EEEERKNQDQQKNEEEDQRYQREGRGKGHQP	-50.00	38.7	HHHHHHHHHHHHHHHHHHHHHHHHCCCCCCC
867	DEERKKQEDQRRNEKEDKRYQREGKGKGHNH	-49.99	38.7	CHHHHHHHHHHHHHHHHHHHHHHCCCCCCCC
386	EEEEKKQRDQRSNEQEDQRYQRYGRGKGHQP	-49.99	41.9	CHHHHHHHHHHHHHHHHHHHHHHHCCCCCCC
266	EEEERKQQDQRKNEKEDEEYQRRGQGKGHQS	-49.99	38.7	HHHHHHHHHHHHHHHHHHHHHHHHHCCCCCC
255	EEEERKQQDQRKNEKEDEEYQRRGRGKGHQP	-49.99	38.7	HHHHHHHHHHHHHHHHHHHHHHHHCCCCCCC
400	EEEERKQQDQRNNEAEDKRYQKEGWGKGHNP	-49.99	45.2	HHHHHHHHHHHHHHHHHHHHHHHHCCCCCCC
038	EEEQKKQEDQRKNEKEDKRYQRYGWGKGHNP	-49.99	41.9	HHHHHHHHHHHHHHHHHHHHHHCCCCCCCCC
110	EEEQRKQMDQMKNEKEDEEYQRRGRGKGHQP	-49.94	38.7	HHHHHHHHHHHHHHHHHHHHHHHHCCCCCCC
494	EEEQRKQQDQFNHEFEDQYYQQKGQGKGHSH	-49.94	48.4	HHHHHHHHHHHHHHHHHHHHHHHHCCCCCCC
898	EQEERKQRDQYNNDWEDRRYQREGKGKGHNH	-49.93	35.5	HHHHHHHHHHHCCHHHHHHHHHCCCCCCCCC
406	EEEQRKQQDQRNNEEEDKKYQRYGKGKGHSS	-49.92	41.9	HHHHHHHHHHHHHHHHHHHHHHHHCCCCCCC
206	EEEQRKQQDQRNNEQEDQRYQRYGRGKGHQP	-49.92	41.9	HHHHHHHHHHHHHHHHHHHHHHHHCCCCCCC
884	EEEEKKNRDQYNNEYEDEEYQRKGRGKGHNP	-49.88	45.2	HHHHHHHHHHHHHHHHHHHHHHHCCCCCCCC
370	EEEQRKNEDQFRNEFEDEFYQQQGKGKGHNH	-49.88	45.2	HHHHHHHHHHHHHHHHHHHHHHHHCCCCCCC
458	EEEEKKKRDKYNNEWEDEEYQRKGRGKGHQP	-49.88	48.4	CHHHHHHHHHHHHHHHHHHHHHHHCCCCCCC
839	EEEERKKEDKKRNEEEDKRYQRYGRGKGHQP	-49.88	41.9	HHHHHHHHHHHHHHHHHHHHHHHHCCCCCCC
762	EEEERKKQDKFNNEFEDKFYQQQGKGKGHSH	-49.88	51.6	HHHHHHHHHHHHHHHHHHHHHHHHCCCCCCC
333	EEEERKKQDKYNNEYEDEEYQRKGRGKGHQP	-49.88	45.2	HHHHHHHHHHHHHHHHHHHHHHHHCCCCCCC
771	DEERKKQEDQYRNESEDQRYQRYGRGKGHQP	-49.86	38.7	HHHHHHHHHHHHHHHHHHHHHHHHCCCCCCC

907	EEEAKKQRDQYNNEYEDEEYQRQGRGKGHQS	-49.86	48.4	HHHHHHHHHHHHHHHHHHHHHHHHHCCCCCCC
750	EEEARKQLDQWKNEFEDKYYQKEGQGKGHQP	-49.86	45.2	HHHHHHHHHHHHHHHHHHHHHHHHHCCCCCCC
979	EEEEKKQRDQKNNEEEDERYQKQGRGKGHQP	-49.86	45.2	HHHHHHHHHHHHHHHHHHHHHHHHCCCCCCCC
062	EEEERKQQDQQKNEEEDSRYQQEGKGKGHSH	-49.86	38.7	HHHHHHHHHHHHHHHHHHHHHHHHCCCCCCCC
032	EEEERKQRDQFNNEKEDQRYQQQGQGKGHQS	-49.86	45.2	HHHHHHHHHHHHHHHHHHHHHHHHHCCCCCCC
591	EEEERKQRDQYNNEAEDQRYQREGRGKGHQP	-49.86	45.2	HHHHHHHHHHHHHHHHHHHHHHHHHCCCCCCC
976	EEEERKQRDQYNNESEDKRYQRYGWGKGHNP	-49.86	41.9	HHHHHHHHHHHCCHHHHHHHHHHCCCCCCCCC
913	EEEKRKQLDQWKNEYEDKSYQREGKGKGHNH	-49.86	41.9	HHHHHHHHHHHHHHHHHHHHHHHCCCCCCCCC
760	EDEQRKQQDQYNNRAEDERYQKEGQGKGHQS	-49.86	38.7	CHHHHHHHHHHHHHHHHHHHHHHHHCCCCCCC
170	EEEERKQQDQRNNEKEHKRKQRYGRGCGKQN	-49.84	32.3	HHHHHHHHHHHHHHHHHHHHHHHHCCCCCCCC
423	EEEERKQRDQFNNEFEDQYYQQKGQGKGHSH	-49.82	45.2	HHHHHHHHHHHHHHHHHHHHHHHHHCCCCCCC
460	EEEQRKQEDQYRNEHEDERYQQQGRGKGHQP	-49.79	38.7	HHHHHHHHHHHHHHHHHHHHHHHHHCCCCCCC
610	EEEQRKQQDQRNNEKEDQEYQRSGKGKGHSS	-49.79	45.2	HHHHHHHHHHHHHHHHHHHHHHHCCCCCCCCC
781	EQEERKQRDKYNQEYEDEEYQRKGRGKGNQN	-49.76	41.9	HHHHHHHHHHHHHHHHHHHHHHHHHCCCCCCC
078	EEEQRKNEDQRNNEKEDQEYQKQGKGKGHSS	-49.76	41.9	HHHHHHHHHHHHHHHHHHHHHHHHCCCCCCCC
222	EQEQRKQQEKYSQEYEDKEYQRQGRGKGHQP	-49.71	35.5	HHHHHHHHHHHHHHHHHHHHHHHHHCCCCCCC
900	EEEERKIQDQQKNEKEDEEYQRRGRGKGHQP	-49.71	38.7	CHHHHHHHHHHHHHHHHHHHHHHHHCCCCCCC
463	EEEERKSRDKFNQEFEDKYYQKEGQGKGHSH	-49.71	48.4	CHHHHHHHHHHHHHHHHHHHHHHHCCCCCCCC
937	EEEQKKQQDQKNNREEDERYQKEGKGKGHNH	-49.70	41.9	HHHHHHHHHHHHHHHHHHHHHHHHCCCCCCCC
702	EEEQRKQQDQYNQEWEDKRYQREGRGKGHQP	-49.68	41.9	HHHHHHHHHHHHHHHHHHHHHHHHHCCCCCCC
153	EEEQRKQQDQYSNEFEDQYYQQQGQGKGHQS	-49.66	38.7	HHHHHHHHHHHHHHHHHHHHHHHHHCCCCCCC
890	EEEEKKKRDKYNNEWEDEYYQRKGQGKGHQH	-49.64	48.4	CHHHHHHHHHHHHHHHHHHHHHHHHCCCCCCC
313	EEEEKKQQDQRNNDKEHQYKQKYGQGSGKQN	-49.62	32.3	CHHHHHHHHHHHHHHHHHHHHHHHCCCCCCCC
489	EEEAKKFRDMFNNRFEDQYYQQQGQGKGHQP	-49.61	51.6	HHHHHHHHHHHHHHHHHHHHHHHHHCCCCCCC
143	EEERRKQLDQWKNEWEDRYYQQKGQGKGHNH	-49.61	41.9	HHHHHHHHHHHHHHHHHHHHHHHHHCCCCCCC
164	EEEQRKQQDQRNNDKEDRRYQREGKGKGHNH	-49.58	38.7	HHHHHHHHHHHHCHHHHHHHHHHHCCCCCCCC
667	EEEERKQRDQFNNRFEDEFYQQKGRGKGHQP	-49.52	45.2	HHHHHHHHHHHHHHHHHHHHHHHHHCCCCCCC
125	EEEQRKQEDKRRNEKEDRRYQREGRGKGHQP	-49.47	41.9	HHHHHHHHHHHHHHHHHHHHHHHHHCCCCCCC
777	EEEQRKQQDQYNNDWEDRKYQREGRGKGHQP	-49.45	38.7	HHHHHHHHHHHCCHHHHHHHHHHHHCCCCCCC
000	EEERRKQLDQFKNEYEDQSYQREGRGKGHQP	-49.44	45.2	HHHHHHHHHHHHHHHHHHHHHHHHCCCCCCCC
613	EEERRKQLDQWKNEFEDKYYQQKGQGKGHQP	-49.44	41.9	HHHHHHHHHHHHHHHHHHHHHHHHHCCCCCCC
733	EQERRKQLDQMKNEKEDEEYQRRGRGKGHQP	-49.44	38.7	HHHHHHHHHHHHHHHHHHHHHHHHHCCCCCCC
674	EEEQKKQQEKYNNESEDKRYQRYGWGKGHNP	-49.42	45.2	HHHHHHHHHHHHHHHHHHHHHHHCCCCCCCCC
939	EEEQKKQQEKYSNEYEDKEYQRNGRGKGHQY	-49.42	41.9	HHHHHHHHHHHHHHHHHHHHHHHCCCCCCCCC
216	EQEQRKQEEKRNNEKEDKRYQREGRGKGHQP	-49.42	38.7	HHHHHHHHHHHHHHHHHHHHHHHHHCCCCCCC
331	EEEERKQRDKYNNESEDRRYQRYGRGKGHQP	-49.41	45.2	HHHHHHHHHHHCCHHHHHHHHHHHHCCCCCCC
050	EEEQKKQQDQYNNEEEDKKYQREGHGKGHQR	-49.38	48.4	HHHHHHHHHHHCHHHHHHHHHHHHHCCCCCCC
699	EEEQKKQRDQYNNESEDQRYQRYGRGKGHQP	-49.38	45.2	HHHHHHHHHHHCCCHHHHHHHHHHHCCCCCCC
735	EEEQKKQRDQYNNEYEDQEYQRQGRGKGHQP	-49.38	45.2	HHHHHHHHHHHHHHHHHHHHHHHHHCCCCCCC
045	EEEQRKQQDQKNNEEEDKRYQQEGKGKGHNH	-49.38	41.9	HHHHHHHHHHHHHHHHHHHHHHHHHCCCCCCC
177	EEEQRKQQDQYNNESEDERYQQQGKGKGHNH	-49.38	41.9	HHHHHHHHHHHCCCHHHHHHHHHHHCCCCCCC
705	EEEQRKQQDQYNNESEDERYQQQGWGKGHYP	-49.38	41.9	HHHHHHHHHHHCCCHHHHHHHHHHHCCCCCCC
572	EEEQRKQQDQYNNESEDQRYQRYGRGKGHQP	-49.38	41.9	HHHHHHHHHHHCCCHHHHHHHHHHHCCCCCCC
549	EEEQRKQQDQYNNEWEDKRYQREGKGKGHSS	-49.38	41.9	HHHHHHHHHHHHHHHHHHHHHHHCCCCCCCCC
511	EEEERKKQDQWKNDWEDRYYQQKGQGKGHNH	-49.37	35.5	HHHHHHHHHHHHHHHHHHHHHHHHHCCCCCCC
800	DEERKKQEDQQRNDKEHKRYQREGKGQGHNH	-49.36	29.0	CHHHHHHHHHHHHHHHHHHHHHHHCCCCCCCC
935	EEEEKKKRDQWKNRWEDEYYQQKGQGKGHNH	-49.33	38.7	HHHHHHHHHHHHHHHHHHHHHHHHHCCCCCCC
687	EEEERKYQDKYNQESEDKRYQRYGWGKGHNP	-49.30	45.2	CHHHHHHHHHHHHHHHHHHHHHHCCCCCCCCC
529	EEEERKKQDQYKNEYEDEEYQRKGRGKGHQP	-49.30	38.7	HHHHHHHHHHHHHHHHHHHHHHHHHCCCCCCC
844	EEEERKKRDQYNNEAEDQRYQREGWGKGHYP	-49.30	45.2	HHHHHHHHHHHHHHHHHHHHHHHHHCCCCCCC
107	EQERRKQLEKWKNEWEDKYYQKEGQGKGHQP	-49.29	38.7	HHHHHHHHHHHHHHHHHHHHHHHHCCCCCCCC
540	EEEQKKQQDQYNNEYEDEEYQRKGRGKGNQN	-49.24	45.2	HHHHHHHHHHHHHHHHHHHHHHHHHCCCCCCC
001	EEEERKKQDQYNNESEDQRFQKEGKGKGKSN	-49.23	38.7	HHHHHHHHHHHCCCHHHHHHHHHHCCCCCCCC
084	EEEEKKKRDQYNQESEDERYQQQGWGKGHNP	-49.23	45.2	CHHHHHHHHHHHHHHHHHHHHHHHCCCCCCCC
573	EEEERKKLDQWKQEYEDEEYQRNGSGKGHQY	-49.23	41.9	CHHHHHHHHHHHHHHHHHHHHHHCCCCCCCCC
067	EEEERKKQDQYNQEYEDEEYQRKGRGKGHQP	-49.23	41.9	HHHHHHHHHHHHHHHHHHHHHHHHHCCCCCCC
891	EEEEKKQREKRSNEKEDEEYQRRGRGKGHQP	-49.22	41.9	CHHHHHHHHHHHHHHHHHHHHHHHHCCCCCCC
214	EEEKKKMLDMKNNREEDERYQQQGWGKGHNP	-49.22	45.2	HHHHHHHHHHHHHHHHHHHHHHHHCCCCCCCC
498	EEEKRKMLDMYKNRYEDEEYQRKGRGKGHQP	-49.22	38.7	HHHHHHHHHHHHHHHHHHHHHHHHHCCCCCCC
473	EEERRKQLDMINNRQEDQRYQREGKGKGHSS	-49.20	41.9	HHHHHHHHHHHHHHHHHHHHHHHHCCCCCCCC
623	EQEQRKQQEKYSNEYEDEEYQRKGRGKGHSN	-49.18	35.5	HHHHHHHHHHHHHHHHHHHHHHHHCCCCCCCC
451	EEEMRKLQDQINNEEEDERYQQQGWGKGHYP	-49.17	41.9	HHHHHHHHHHHHHHHHHHHHHHHHCCCCCCCC
592	EDEQRKQQDQRSNRKEDEEYQKRGWGKGHYP	-49.15	32.3	CHHHHHHHHHHHHHHHHHHHHHHHCCCCCCCC
325	EEEKRKQLEKYNNESEDKRYQQYGQGKGHQS	-49.10	45.2	HHHHHHHHHHHHHHHHHHHHHHHHHCCCCCCC
480	EEEQRKHEDKNRHEQEDKRYQREGKGKGHSH	-49.08	45.2	HHHHHHHHHHHHHHHHHHHHHHHHCCCCCCCC
204	EQQQRKQQEKYSNEYEQKEYQKEGRGRGHQP	-49.06	32.3	HHHHHHHHHHHHHHHHHHHHHHHHHCCCCCCC
034	EEEEKKYQDQYNNDYEDEEYQRKGRGKGHQP	-49.06	41.9	CHHHHHHHHHHHCCCHHHHHHHHHHCCCCCCC

231	EEEERKNRDKYNNEAEDKRYQREGKGKGHSS	-49.05	48.4	HHHHHHHHHHHCHHHHHHHHHHHCCCCCCCC
502	EEEERKKQDQYNNEYEDEEFQRKGRGKGKQN	-49.04	38.7	HHHHHHHHHHHHHHHHHHHHHHHHCCCCCCC
068	EEEQRKQQDAYNNRAEDERYQRYGRGKGHQP	-49.03	41.9	HHHHHHHHHHHHHHHHHHHHHHHHCCCCCCC
320	EQERRKQQEKYSNESEDKRYQRYGKGKGHNS	-49.02	35.5	HHHHHHHHHHHHHHHHHHHHHHHHCCCCCCC
069	EEEKRKWLDKLNNEKEDEKFQREGQGKGKQS	-49.01	45.2	HHHHHHHHHHHHHHHHHHHHHHHHCCCCCCC
599	EEEERKYQDKKNNEEEDKRYQRYGKGKGHSS	-49.00	45.2	CHHHHHHHHHHHHHHHHHHHHHHCCCCCCCC
175	EEEERKYQDKYKNEWEDKKYQEEGKGKGHNH	-49.00	41.9	CHHHHHHHHHHHHHHHHHHHHHHHCCCCCCC
426	EEEERKQQDQRNNDSEHRRKQKEGWGSGKYP	-48.99	29.0	HHHHHHHHHHHHHHHHHHHHHHHHCCCCCCC
281	EEEQRKQQDQYNNEWEHEEKQRKGRGSGKQN	-48.98	32.3	HHHHHHHHHHHHHHHHHHHHHHHCCCCCCCC
799	EEEERKKQDQYNNEAEDQRYQREGKGKGHNH	-48.98	45.2	HHHHHHHHHHHHHHHHHHHHHHHHCCCCCCC
395	EEEERKKQDQYNNRYEDEEYQRKGRGKGHQP	-48.96	38.7	HHHHHHHHHHHHHHHHHHHHHHHHCCCCCCC
420	EEEARKNQDQYNQESEDERYQQQGKGKGDSR	-48.94	51.6	HHHHHHHHHHHHHHHHHHHHHHHCCCCCCCC
369	EEEQRKQQDQYNNEAEDQRYQREGKGKGDSR	-48.94	51.6	HHHHHHHHHHHHHHHHHHHHHHHCCCCCCCC
345	EEEERKQQQKKNNEEEDKRFQRYGKGKGKNR	-48.93	41.9	HHHHHHHHHHHHHHHHHHHHHHHCCCCCCCC
653	EEEEKKKRDQYNNEWEDQKYQREGWGKGHNP	-48.93	45.2	CHHHHHHHHHHHHHHHHHHHHHHHCCCCCCC
753	EEEEKKKRDQYNNEYEDEEYQRKGRGKGHQP	-48.93	45.2	CHHHHHHHHHHHHHHHHHHHHHHHCCCCCCC
348	EEEEKKKRDQYNNEYEDQEYQRKGRGKGHQP	-48.93	45.2	CHHHHHHHHHHHHHHHHHHHHHHHCCCCCCC
636	EEEERKKEDQWRNEEEDKKYQREGRGKGHQP	-48.93	38.7	HHHHHHHHHHHCHHHHHHHHHHHHCCCCCCC
845	EEEERKKQDQYNNESEDQRYQQQGWGKGHNP	-48.93	41.9	HHHHHHHHHHHCCCHHHHHHHHHHCCCCCCC
588	EEEERKKQDQYNNESEDQRYQRYGWGKGHYP	-48.93	41.9	HHHHHHHHHHHCCCHHHHHHHHHCCCCCCCC
565	EEEERKKQDQYNNESEDSRYQQQGKGKGHNH	-48.93	41.9	HHHHHHHHHHHCCCHHHHHHHHHCCCCCCCC
166	EEEERKKQDQYNNEWEDQRYQREGWGKGHYP	-48.93	41.9	HHHHHHHHHHHHHHHHHHHHHHHHCCCCCCC
305	EEEERKKQDQYSNEYEDEEYQRKGRGKGHQP	-48.93	38.7	HHHHHHHHHHHHHHHHHHHHHHHHCCCCCCC
156	QEEEKKKQDQYNNEHEDERYQRQGWGKGHYP	-48.93	41.9	CHHHHHHHHHHHHHHHHHHHHHHHCCCCCCC
822	EEEKRKQEDQRNNEEEDQYYQQKGQGKGNQN	-48.92	41.9	HHHHHHHHHHHHHHHHHHHHHHHHCCCCCCC
543	EEEKRKLLDQIENEREDQYYQQKGQGKGHQP	-48.91	41.9	HHHHHHHHHHHHHHHHHHHHHHHHCCCCCCC
049	EEEQRKQEQKRRNEKEDKQYQRSGRGKGHQP	-48.91	41.9	HHHHHHHHHHHHHHHHHHHHHHCCCCCCCCC
104	EEEQRKFEDKRNNEEEDKRYQQQGRGKGHQP	-48.90	48.4	CHHHHHHHHHHHHHHHHHHHHHHHCCCCCCC
459	EEEEKKQRDQYNNEYEHQEKQRKGRGSGKSN	-48.86	35.5	HHHHHHHHHHHHHHHHHHHHHHCCCCCCCCC
165	EEEERKQRDQYNNEAEDQRYQREGKGKGHNH	-48.85	45.2	HHHHHHHHHHHHHHHHHHHHHHHHCCCCCCC
536	EEEERKKQDQYNNRYEHEEKQKEGRGCGKQN	-48.85	29.0	HHHHHHHHHHHHHHHHHHHHHHHCCCCCCCC
385	EQEERKQQEQRSQEKEDKEFQKQGRGKGKQN	-48.82	29.0	HHHHHHHHHHHHHHHHHHHHHHHHCCCCCCC
183	EEEARKSQDKQNNEREDKRFQREGKGKGKNH	-48.78	45.2	HHHHHHHHHHHHHHHHHHHHHHHCCCCCCCC
264	EEEQRKQQQKYNNEYEDEEYQRKGRGKGHQP	-48.78	41.9	HHHHHHHHHHHHHHHHHHHHHHHHCCCCCCC
712	EEEQRKQQQKYSNEWEDEEYQRKGRGKGHQP	-48.78	38.7	HHHHHHHHHHHHHHHHHHHHHHHHCCCCCCC
933	EEEERKQQQKRSNEQEDKRYQREGRGKGHQP	-48.78	38.7	HHHHHHHHHHHHHHHHHHHHHHHHCCCCCCC
041	DEERKKWEDKKRNEEEDKRYQRYGKGKGHSH	-48.78	41.9	CHHHHHHHHHHHHHHHHHHHHHHHCCCCCCC
047	EEEQKKYEDKRNNEEEDKSYQRHGKGKGHSH	-48.77	48.4	CHHHHHHHHHHHHHHHHHHHHHHCCCCCCCC
436	EEEQRKLLDQINNDAEDRRYQREGRGKGHQP	-48.76	45.2	HHHHHHHHHHHHHHHHHHHHHHHHCCCCCCC
028	EEQRRKQLDMINNRAEDQRYQRYGRGKGHQP	-48.76	48.4	HHHHHHHHHHHHHHHHHHHHHHHHCCCCCCC
598	EEEERKLQDQKKNEAEDQRYQREGRGKGHQP	-48.75	41.9	CHHHHHHHHHHHHHHHHHHHHHHHCCCCCCC
519	EEEKRKLLDQINNEQEDQRYQREGKGKGHNH	-48.75	45.2	HHHHHHHHHHHHHHHHHHHHHHHHCCCCCCC
557	EEEQRKLEDQKKNEQEDQRYQRYGRGKGHQP	-48.75	38.7	HHHHHHHHHHHHHHHHHHHHHHHHCCCCCCC
225	EEEQRKLEDQKSNEKEDEEYQRRGKGKGHNH	-48.75	38.7	HHHHHHHHHHHHHHHHHHHHHHHHCCCCCCC
757	EEEQRKLYDQINNEKEDEEYQRYGWGKGHNP	-48.75	41.9	HHHHHHHHHHHHHHHHHHHHHHHCCCCCCCC
244	EEEQRKQEEQRSQRKEDERYQREGQGKGHQS	-48.75	32.3	HHHHHHHHHHHHHHHHHHHHHHHHCCCCCCC
738	EEEERKKQDQQNNEREDEEYQRKGRGKGHNP	-48.74	41.9	HHHHHHHHHHHHHHHHHHHHHHHCCCCCCCC
455	EEEERKKQDQYNNEAEDQRYQREGKGKGHSH	-48.73	45.2	HHHHHHHHHHHHHHHHHHHHHHHCCCCCCCC
322	EEEEKKKRDQQNNEREDEEYQRRGRGKGHQP	-48.72	45.2	CHHHHHHHHHHHHHHHHHHHHHHHCCCCCCC
746	EEEERKKQEKQSNEREDEEYQRKGRGKGHQP	-48.72	38.7	HHHHHHHHHHHHHHHHHHHHHHHHCCCCCCC
848	EEEKRKQEEKRNNEEEDKRYQREGKGKGHNN	-48.69	41.9	HHHHHHHHHHHHHHHHHHHHHHHCCCCCCCC
938	DEERKKSEDQYNNEAEDERYQQQGKGKGHNH	-48.69	45.2	CHHHHHHHHHHHHHHHHHHHHHHHCCCCCCC
274	EEERKKQLEKYNNESEDKRYQRYGRGKGHQP	-48.68	48.4	HHHHHHHHHHHHHHHHHHHHHHHHCCCCCCC
742	EEEERKKQDKYKNEYEDKEYQRKGRGKGHQP	-48.65	41.9	HHHHHHHHHHHHHHHHHHHHHHHCCCCCCCC
555	EEEERKQRQKYNNEWEDKRYQREGRGKGHQP	-48.65	41.9	CHHHHHHHHHHHHHHHHHHHHHHHCCCCCCC
696	EEERRKQLQKYKNEYEDEEYQRKGRGKGHQP	-48.65	41.9	HHHHHHHHHHHHHHHHHHHHHHHHCCCCCCC
970	EEERRKQLQKYNNESEDKRYQRYGRGKGHQP	-48.65	45.2	HHHHHHHHHHHHHHHHHHHHHHHHCCCCCCC
745	EEERRKQQQKYSNEYEDEEYQRKGRGKGHQP	-48.65	38.7	HHHHHHHHHHHHHHHHHHHHHHHHCCCCCCC
975	EQEERKKQDKIRHEEEDKRYQQQGKGKGHSH	-48.63	41.9	HHHHHHHHHHHHHHHHHHHHHHHCCCCCCCC
478	EEEQRKQQQQYSNDAEDKQYQQRGRGKGHQP	-48.61	35.5	HHHHHHHHHHHHHHHHHHHHHHHHCCCCCCC
945	EQEQRKQQEKYNNEYEDKQYQKEGKGKGHSS	-48.61	38.7	HHHHHHHHHHHHHHHHHHHHHHCCCCCCCCC
184	EEEEKKKRDQFNNDFEDRYYQQQGQGKGHQS	-48.61	45.2	CHHHHHHHHHHHHHHHHHHHHHHHCCCCCCC
988	EEEERKKQDQQNNDEEDRRYQRYGWGKGHYP	-48.61	38.7	HHHHHHHHHHHHCHHHHHHHHHHCCCCCCCC
011	EEQQKKQQDQRSNEKEHEEYQRRGQGQGHQS	-48.59	38.7	HHHHHHHHHHHHHHHHHHHHHHHHHCCCCCC
656	EEEERKYQDQYKQEWEDKKYQREGKGKGHNH	-48.59	38.7	CHHHHHHHHHHHHHHHHHHHHHHCCCCCCCC
969	EEEQRKYEDQRNQEKEDERYQQQGWGKGHNP	-48.59	41.9	CHHHHHHHHHHHHHHHHHHHHHHCCCCCCCC
303	EEERKKQLEQYKQEWEDQRYQREGQGKGHQS	-48.58	41.9	HHHHHHHHHHHHHHHHHHHHHHHHCCCCCCC

607	EEEERKMRDQYNNDWEDQRYQREGKGKGHNH	-48.57	38.7	HHHHHHHHHHHHCHHHHHHHHHHCCCCCCCC
353	EEEERKKQDQYNNEYEDEEYQRKGRGKGHQP	-48.53	41.9	HHHHHHHHHHHHHHHHHHHHHHHHCCCCCCC
734	EEEERKKQDQYNNEYEDQEYQRNGRGKGHQY	-48.53	41.9	HHHHHHHHHHHHHHHHHHHHHHHCCCCCCCC
951	EEEEKKKREKFSNEFEDKYYQQKGQGKGHQH	-48.53	45.2	CHHHHHHHHHHHHHHHHHHHHHHHCCCCCCC
245	EEQRRKQLDKINNEAEDKRYQREGKGKGHSS	-48.52	54.8	HHHHHHHHHHHHHHHHHHHHHHHCCCCCCCC
020	EEEERKYQDQYNNEYEDQYFQKKGQGKGKNH	-48.52	38.7	CHHHHHHHHHHHHHHHHHHHHHHHCCCCCCC
339	EEEQRKQEQKRRNEKEDKRYQRYGRGKGHQP	-48.52	38.7	HHHHHHHHHHHHHHHHHHHHHHHHCCCCCCC
603	EEEKKKLLDQINNEAEDKRYQRYGQGKGHQS	-48.52	51.6	HHHHHHHHHHHHHHHHHHHHHHHHHCCCCCC
770	EQEERKKQDQYNQEYEDEEYQRKGRGKGHQP	-48.51	38.7	HHHHHHHHHHHHHHHHHHHHHHHHCCCCCCC
991	DEERKKMLDKKNNEEEDERYQQQGRGKGHQP	-48.51	48.4	HHHHHHHHHHHHHHHHHHHHHHHHCCCCCCC
652	EEERRKQLDQWKNEYEDQSYQREGRGKGNQN	-48.51	41.9	HHHHHHHHHHHHHHHHHHHHHHHCCCCCCCC
318	EEEQKKQEEQYRNDYEDREYQRKGRGKGHNP	-48.49	35.5	HHHHHHHHHHHHHHHHHHHHHHCCCCCCCCC
139	EEEERKQQEQRSNDQEDRRYQKEGRGKGHQP	-48.49	32.3	HHHHHHHHHHHHHHHHHHHHHHHHCCCCCCC
434	EEEQRKQEEQRRNDQEDRRYQQYGRGKGHQP	-48.49	32.3	HHHHHHHHHHHHHHHHHHHHHHHHCCCCCCC
522	EQEERKQQEQRSNRQEDERYQREGRGKGHQP	-48.49	29.0	HHHHHHHHHHHHHHHHHHHHHHHHCCCCCCC
584	EEERRKQLDKIKNEQEDKSYQRYGQGKGHQS	-48.48	45.2	HHHHHHHHHHHHHHHHHHHHHHHHCCCCCCC
691	EEERRKQLDKKNNEEEDKRYQQQGRGKGHQP	-48.48	48.4	HHHHHHHHHHHHHHHHHHHHHHHHCCCCCCC
301	EEEERKKQDKYNNESEDRRYQRYGRGKGHQP	-48.48	45.2	HHHHHHHHHHHCCHHHHHHHHHHHCCCCCCC
561	EEEQKKQQDKYSNEYEQKQYQKEGQGQGHSS	-48.47	38.7	HHHHHHHHHHHHHHHHHHHHHHHCCCCCCCC
774	EEEQKKQRDKFNNEFEQKYYQQKGQGQGHNH	-48.47	45.2	HHHHHHHHHHHHHHHHHHHHHHHHCCCCCCC
521	EEEQKKQRDKYNNEWEQKRYQREGKGQGHNH	-48.47	41.9	HHHHHHHHHHHHHHHHHHHHHHHHCCCCCCC
689	EEEQRKQQDKFNNEKEQQRYQQQGRGQGHQP	-48.47	41.9	HHHHHHHHHHHHHHHHHHHHHHHHCCCCCCC
875	EEEQRKQQDKYSNEYEQKEYQKEGRGQGHQP	-48.47	35.5	HHHHHHHHHHHHHHHHHHHHHHHHCCCCCCC
737	EEEERKYQDQYNNEYEHEEYQRKGRGRGHQP	-48.45	35.5	CHHHHHHHHHHHHHHHHHHHHHHHCCCCCCC
874	EQEQRKQQDHYNNRWEDEEYQRKGRGKGHQP	-48.45	35.5	HHHHHHHHHHHHCHHHHHHHHHHHCCCCCCC
747	EEEQRKRQDQYNNDWEDEEYQRKGRGKGHNP	-48.44	38.7	HHHHHHHHHHHCCCHHHHHHHHHCCCCCCCC
782	EEEERKFQDQWKNEFEDEYFQRKGQGKGKSH	-48.42	38.7	CHHHHHHHHHHHHHHHHHHHHHHHCCCCCCC
075	EQQQRKQQEQYSNEYEDEEYQRKGRGKGHNP	-48.42	35.5	HHHHHHHHHHHHHHHHHHHHHHHCCCCCCCC
405	EEEQRKQQEQFSNEYEDQYYQQQGQGKGHNH	-48.42	38.7	HHHHHHHHHHHHHHHHHHHHHHHHCCCCCCC
432	EEEEKKQQEQRSNEKEDEEYQRRGQGKGHQS	-48.41	38.7	HHHHHHHHHHHHHHHHHHHHHHHHHCCCCCC
567	EEEERKQQEQRSNEQEDKRYQREGKGKGHNH	-48.41	35.5	HHHHHHHHHHHHHHHHHHHHHHHCCCCCCCC
792	EEEERKKRDQYNNDYEHEEYQRKGRGQGHQP	-48.41	32.3	HHHHHHHHHHHCHHHHHHHHHHHHCCCCCCC
189	EEEEKKKRDQYNNEFEDEYYQQKGQGKGHQH	-48.41	45.2	CHHHHHHHHHHHHHHHHHHHHHHHCCCCCCC
488	EEEERKQRDKESNEKEDKRYQREGWGKGHYP	-48.41	41.9	HHHHHHHHHHHHHHHHHHHHHHHHCCCCCCC
818	EEEERKYQDQYKNEWEDRRYQREGNGKGHSH	-48.39	38.7	CHHHHHHHHHHHHHHHHHHHHHCCCCCCCCC
928	EEEQKKQEQKYRNEWEDKKYQREGRGKGHQP	-48.39	41.9	HHHHHHHHHHHHHHHHHHHHHHHHCCCCCCC
626	EEEQRKQQDQYNNDWEQQRYQREGRGQGHQP	-48.35	32.3	HHHHHHHHHHHHHHHHHHHHHHHHCCCCCCC
355	EEEQRKQQDQYNNDYEQEEYQQQGRGQGHQP	-48.35	32.3	HHHHHHHHHHHHHHHHHHHHHHHHCCCCCCC
670	EEEERKKQDQFNNEFENKYYQQKGQGKGHQH	-48.34	41.9	HHHHHHHHHHHHHHHHHHHHHHHHCCCCCCC
879	EEEERKQRDKYNNESEQKRYQRYGRGQGHQP	-48.34	38.7	HHHHHHHHHHHCHHHHHHHHHHHHHCCCCCC
862	EEERRKQLDKWKNEWEQKYYQQKGQGQGHSH	-48.34	38.7	HHHHHHHHHHHHHHHHHHHHHHHHCCCCCCC
329	EEERRKQLDKYKNEYEQKEYQRKGRGQGHQP	-48.34	38.7	HHHHHHHHHHHHHHHHHHHHHHHHCCCCCCC
520	EEEERKMRDQYNNDYEDEEYQRKGRGKGHSN	-48.33	38.7	HHHHHHHHHHHCCCHHHHHHHHHCCCCCCCC
002	EEEERKYQDQRNNDKEDREYQRRGRGKGHQP	-48.30	38.7	CHHHHHHHHHHCCHHHHHHHHHHHCCCCCCC
129	EEEERKYQDQYNNDSEDRRYQRYGRGKGHQP	-48.30	38.7	CHHHHHHHHHCCCCHHHHHHHHHHCCCCCCC
464	EEEERKQQEQRNNRKEDERYQRYGRGKGHQP	-48.29	35.5	HHHHHHHHHHHHHHHHHHHHHHHHCCCCCCC
801	EEEEKKQREQYNNESEDERYQKQGRGKGHQP	-48.29	41.9	CHHHHHHHHHHCCHHHHHHHHHHCCCCCCCC
954	EEEERKQQEQYSNEWEDERYQQQGWGKGHYP	-48.29	35.5	HHHHHHHHHHHHHHHHHHHHHHHHCCCCCCC
570	EEEERKQREQFNNEFEDKYYQQKGQGKGHQP	-48.29	41.9	HHHHHHHHHHHHHHHHHHHHHHHHCCCCCCC
044	EEERKKQLEQWKNEWEDQYYQKEGQGKGHQH	-48.29	41.9	HHHHHHHHHHHHHHHHHHHHHHHHCCCCCCC
230	EEERKKQLEQYKNEAEDKRYQQYGWGKGHNP	-48.29	45.2	HHHHHHHHHHHHHHHHHHHHHHHCCCCCCCC
146	EEERKKQLEQYNNESEDQRYQRYGRGKGHQP	-48.29	45.2	HHHHHHHHHHHHHHHHHHHHHHHHCCCCCCC
985	EEERRKQEEQQRNEEEDQRYQRYGRGKGHQP	-48.29	35.5	HHHHHHHHHHHHHHHHHHHHHHHHCCCCCCC
832	EEERRKQLEQYNNEYEDEEYQRKGRGKGHQP	-48.29	41.9	HHHHHHHHHHHHHHHHHHHHHHHHCCCCCCC
732	EEERRKQLEQYSNEYEDQEYQRVGRGKGHQP	-48.29	38.7	HHHHHHHHHHHHHHHHHHHHHHHHCCCCCCC
780	EQEERKQREQYNNEAEDQRYQREGKGKGHSH	-48.29	38.7	HHHHHHHHHHHHHHHHHHHHHHHCCCCCCCC
417	EEEEKKYQDQYNNESEDQRYQRYGRGKGHQP	-48.28	45.2	CHHHHHHHHHHCCCHHHHHHHHHHCCCCCCC
157	EEEERKYQDQKNNEEEDQRYQRYGRGKGHQP	-48.28	41.9	CHHHHHHHHHHHHHHHHHHHHHHHCCCCCCC
415	EEEERKYQDQYKNEWEDKRYQREGRGKGHQP	-48.28	38.7	CHHHHHHHHHHHHHHHHHHHHHHHCCCCCCC
600	EEEERKYQDQYKNEYEDQYYQKKGQGKGHSH	-48.28	38.7	CHHHHHHHHHHHHHHHHHHHHHHHCCCCCCC
421	EEEERKYQDQYNNESEDKRYQKEGWGKGHYP	-48.28	41.9	CHHHHHHHHHHCCHHHHHHHHHHHCCCCCCC
698	EEEERKYQDQYNNEYEDQEYQRNGRGKGHQY	-48.28	41.9	CHHHHHHHHHHHHHHHHHHHHHCCCCCCCCC
984	EEEQKKYEDQRNNEEEDEEYQRKGRGKGHQP	-48.28	45.2	CHHHHHHHHHHCHHHHHHHHHHHHCCCCCCC
341	EEEQRKYEDQRNNEAEDQRYQRQGWGKGHNP	-48.28	45.2	CHHHHHHHHHHHHHHHHHHHHHHHCCCCCCC
587	EEEQRKYEDQRNNEEEDEEYQRKGRGKGHQP	-48.28	41.9	CHHHHHHHHHHCHHHHHHHHHHHHCCCCCCC
286	EEEQRKYEDQRNNEEEDERYQQQGKGKGHSS	-48.28	41.9	CHHHHHHHHHHHHHHHHHHHHHHCCCCCCCC
290	EEEQRKYEDQRNNEEEDKRYQREGKGKGHSS	-48.28	41.9	CHHHHHHHHHHHHHHHHHHHHHHCCCCCCCC

790	EEEQRKYEDQRNNEEEDQQYQKQGRGKGHQP	-48.28	41.9	CHHHHHHHHHHHHHHHHHHHHHHHHCCCCCCC
027	EEEQRKYEDQRNNEEEDQRYQREGWGKGHNP	-48.28	41.9	CHHHHHHHHHHHHHHHHHHHHHHHHCCCCCCC
198	EEEQRKYEDQRNNEEEDQRYQRYGRGKGHQP	-48.28	41.9	CHHHHHHHHHHHHHHHHHHHHHHHHCCCCCCC
784	KQEEEKYQDQYNNEWEDEEYQRKGRGKGHQP	-48.28	35.5	CHHHHHHHHHHHHHHHHHHHHHHHHCCCCCCC
841	EEEERKRQDQENNDKEDEEYQRRGWGKGHNP	-48.26	38.7	HHHHHHHHHHHHCHHHHHHHHHHHHCCCCCCC
273	EEQARKSQDQYNNEHEDERYQQQGRGKGHQP	-48.25	48.4	HHHHHHHHHHHHHHHHHHHHHHHHHCCCCCCC
036	EEERKKQLDKESNEKEDKRYQREGQGKGHQS	-48.25	48.4	HHHHHHHHHHHHHHHHHHHHHHHHHCCCCCCC
496	EEEERKFQQQYSEEYEDEEYQRKGRGKGHQP	-48.25	38.7	CHHHHHHHHHHHHHHHHHHHHHHHHCCCCCCC
887	EEERRKQLDKKNNEEEDKRYQQQGKGKGHNH	-48.25	48.4	HHHHHHHHHHHHHHHHHHHHHHHHCCCCCCCC
121	EEEQRKLYDQINNRKEDEEYQRAGRGKGHQP	-48.24	38.7	HHHHHHHHHHHHHHHHHHHHHHHHHHCCCCCC
115	EEEQRKQQEQRSNDAEDRQYQQRGRGKGHQP	-48.23	35.5	HHHHHHHHHHHHCHHHHHHHHHHHHCCCCCCC
606	EQEERKQQEQRSNEEEDKRYQREGWGKGHYP	-48.22	32.3	HHHHHHHHHHHHHHHHHHHHHHHHHCCCCCCC
012	EQEERKKQEKYNQEAEDKRYQRYGRGKGHQP	-48.22	41.9	HHHHHHHHHHHHHHHHHHHHHHHHHCCCCCCC
477	EEEERKREDKYRNEYEDEYYQQQGQGKGHQP	-48.20	41.9	HHHHHHHHHHHHHHHHHHHHHHHHHCCCCCCC
213	EEEERKRQDKENNEREDKRYQREGQGKGHQS	-48.20	45.2	HHHHHHHHHHHHHHHHHHHHHHHHCCCCCCCC
924	EEEERKRQDKQNNESEDKSYQREGRGKGHQP	-48.20	45.2	HHHHHHHHHHHHHHHHHHHHHHHHCCCCCCCC
905	EEEERKKQDQYNNEYEDEEYQRKGRGKGDQR	-48.19	48.4	HHHHHHHHHHHHHHHHHHHHHHHHCCCCCCCC
081	EEEEKKFRDQYNNEYEDQEYQRKGRGKGHQP	-48.19	48.4	CHHHHHHHHHHHHHHHHHHHHHHHHCCCCCCC
195	EEEERKFRDQYNNEYEDQQYQKEGRGKGHQP	-48.19	45.2	CHHHHHHHHHHHHHHHHHHHHHHHHCCCCCCC
548	EEEKKKFLDQYNNEFEDKMYQRYGKGKGHQP	-48.19	51.6	HHHHHHHHHHHHHHHHHHHHHHHHHHCCCCCC
338	EEEKRKFLDQYKNEYEDEEYQRKGRGKGHQP	-48.19	45.2	HHHHHHHHHHHHHHHHHHHHHHHHHCCCCCCC
638	EEEQRKFEDQRNNEEEDKRYQREGRGKGHQP	-48.19	45.2	CHHHHHHHHHHHHHHHHHHHHHHHHCCCCCCC
917	EEEQKKQRDQFNNEWEHKRKQREGRGSGKQN	-48.18	38.7	HHHHHHHHHHHHHHHHHHHHHHHHCCCCCCCC
718	DEERKKQEEQYRNDYEDREYQRKGRGKGHQP	-48.17	32.3	HHHHHHHHHHHHHHHHHHHHHHHHHCCCCCCC
294	EEEQKKQRQQYNNDAEDRRFQRYGRGKGKQN	-48.16	38.7	HHHHHHHHHHHHHHHHHHHHHHHHCCCCCCCC
685	EEEQRKQQDQYNNEWEDKRYQREGKGKGDNR	-48.13	48.4	HHHHHHHHHHHHHHHHHHHHHHHCCCCCCCCC
501	EEERRKQLQVESERKEDQRYQRYGWGKGHYP	-48.13	35.5	HHHHHHHHHHHHHHHHHHHHHHHHCCCCCCCC
931	EEEQKKFRDQFNNEFEDEFYQQQGRGKGHQP	-48.12	54.8	HHHHHHHHHHHHHHHHHHHHHHHHHCCCCCCC
986	EEEQKKFRDQYNNEWEDEEYQRKGRGKGHQP	-48.12	48.4	HHHHHHHHHHHHCHHHHHHHHHHHHCCCCCCC
263	EEEQRKFQDQYNNEEEDKRYQREGKGKGHSS	-48.12	45.2	HHHHHHHHHHHHHHHHHHHHHHHHHCCCCCCC
425	EEEERKKQDQIENEREDSRYQQQGWGKGHYP	-48.10	38.7	HHHHHHHHHHHHHHHHHHHHHHHHCCCCCCCC
664	DEERKKMLDQKNNEEEDQRFQREGKGKGKNN	-48.10	41.9	HHHHHHHHHHCCHHHHHHHHHHHHCCCCCCCC
617	EEEKKKMLDQFNQEFEDQYYQQKGQGKGHNH	-48.09	51.6	HHHHHHHHHHHHHHHHHHHHHHHHHCCCCCCC
404	QEEEKKMRDQYNQEWEDKRYQREGKGKGHNH	-48.09	41.9	CHHHHHHHHHHHHHHHHHHHHHHCCCCCCCCC
247	EEEARKQQEQYSNEWEDKRYQREGRGKGHQP	-48.09	38.7	HHHHHHHHHHHHHHHHHHHHHHHHHCCCCCCC
534	EEEWKKQMEQKNNEEEDKRYQREGRGKGHQP	-48.09	41.9	HHHHHHHHHHHHHHHHHHHHHHHHHCCCCCCC
122	EEEQRKQQDQYNNEYEHQEYQKEGRGQGHQP	-48.07	35.5	HHHHHHHHHHHHHHHHHHHHHHHHHCCCCCCC
217	EEEERKQQQKRSNEKEDQRYQQQGWGKGHNP	-48.07	38.7	HHHHHHHHHHHHHHHHHHHHHHHHCCCCCCCC
051	EEEERKWRDQKNNEEEDERYQRYGWGKGHNP	-48.06	41.9	CHHHHHHHHHCHHHHHHHHHHHHHCCCCCCCC
372	EEEYRKWQDQKNNEEEDERYQQQGRGKGHQP	-48.06	41.9	HHHHHHHHHHHHHHHHHHHHHHHHHCCCCCCC
812	EEEWRKWIDQLKNEKEDKRYQREGRGKGHQP	-48.05	38.7	HHHHHHHHHHHHHHHHHHHHHHHHHCCCCCCC
337	EEERKKQLDQWKNRWEDEYFQQQGRGKGKQN	-48.05	38.7	HHHHHHHHHHHHHHHHHHHHHHHHHCCCCCCC
224	EEEKKKMLDQINNEKEDEYFQQYGQGKGKSH	-48.03	45.2	HHHHHHHHHHHHHHHHHHHHHHHHHHCCCCCC
741	EEEQRKYEDQRNNREEDERYQRYGRGKGHQP	-48.00	38.7	CHHHHHHHHHHHHHHHHHHHHHHHHHCCCCCC
813	EEEQKKYEDQRNQEEEDKRYQREGQGKGHQS	-47.99	45.2	CHHHHHHHHHHHHHHHHHHHHHHHHCCCCCCC
424	EEEERKKMQKMEQEREDKEYQQKGRGKGHQP	-47.98	38.7	CHHHHHHHHHHHHHHHHHHHHHHHHCCCCCCC
031	EEERRKQEEQYRQEAEDQRYQREGWGKGHYP	-47.97	38.7	HHHHHHHHHHHHHHHHHHHHHHHHHCCCCCCC
287	EEEQRKQQDKYNQESEQKRYQRYGQGQGHQS	-47.97	38.7	HHHHHHHHHHHHHHHHHHHHHHHHHHCCCCCC
229	EEEERKKQDKYNNEFEHKYYQQKGQGQGHSH	-47.96	38.7	HHHHHHHHHHHHHHHHHHHHHHHHHCCCCCCC
665	EEEERKQRQQYNNEFEDQYFQQKGQGKGKQN	-47.96	35.5	HHHHHHHHHHHHHHHHHHHHHHHHHCCCCCCC
486	EEERRKQLEQQSNDREDRYYQREGKGKGHSH	-47.95	35.5	HHHHHHHHHHHHHHHHHHHHHHHCCCCCCCCC
066	EEEEKKKQDQINNDKEDREYQRYGKGKGHSS	-47.95	41.9	CHHHHHHHHHHHHHHHHHHHHHHHCCCCCCCC
413	EEEERKKQDQWKNDWEDRFYQKEGKGKGHNH	-47.95	38.7	HHHHHHHHHHHHHHHHHHHHHHHHCCCCCCCC
877	EEQQRKQKQQFDNEKEHQRYQREGQGRGHQS	-47.95	35.5	HHHHHHHHHHHCHHHHHHHHHHHHHCCCCCCC
387	EEEKRKFLDQYNNESEDERYQQQGRGKGHQP	-47.95	48.4	HHHHHHHHHHHCCCHHHHHHHHHHHCCCCCCC
127	EEEERKQQQQYKQEWEDEEYQRKGRGKGHQP	-47.94	35.5	HHHHHHHHHHHHHHHHHHHHHHHHHCCCCCCC
352	EEEEKKKQDQEKNEREDKRYQRYGRGKGHQP	-47.94	41.9	CHHHHHHHHHHHHHHHHHHHHHHHHCCCCCCC
717	EEEEKKKQDQIKNEKEDEEYQRRGRGKGHQP	-47.94	41.9	CHHHHHHHHHHHHHHHHHHHHHHHHCCCCCCC
112	EEEERKKQDQIKNEEEDKKYQREGRGKGHQP	-47.94	38.7	HHHHHHHHHHHHHHHHHHHHHHHHHCCCCCCC
363	EEEERKKQDQIKNEEEDQRYQQYGRGKGHQP	-47.94	38.7	HHHHHHHHHHHHHHHHHHHHHHHHHHCCCCCC
727	EEEERKKQDQIKNEKEDEEYQRYGKGKGHSS	-47.94	38.7	HHHHHHHHHHHHHHHHHHHHHHHHHCCCCCCC
644	EEEERKKQDQIKNEKEDQEYQKQGKGKGHSH	-47.94	38.7	HHHHHHHHHHHHHHHHHHHHHHHHCCCCCCCC
147	EEEERKKQDQINNEAEDKRYQKEGKGKGHNH	-47.94	45.2	HHHHHHHHHHHHHHHHHHHHHHHHCCCCCCCC
285	EEEWKKKMDQFNNEKEDKRYQQQGWGKGHNP	-47.94	48.4	HHHHHHHHHHHHHHHHHHHHHHHHCCCCCCCC
989	EEEERKKQDQLNNRKEDERYQREGRGKGHQP	-47.92	38.7	HHHHHHHHHHHHHHHHHHHHHHHHHCCCCCCC
783	EEEERKRQDQEKNEKEDKRYQREGKGKGHNH	-47.91	38.7	HHHHHHHHHHHHHHHHHHHHHHHHCCCCCCCC
516	EEEERKRQDQEKNEKEDKRYQRYGRGKGHQP	-47.91	38.7	HHHHHHHHHHHHHHHHHHHHHHHHHCCCCCCC

091	EEEERKRQDQEKNEKEDQRYQREGKGKGHSH	-47.91	38.7	HHHHHHHHHHHHHHHHHHHHHHHHCCCCCCCC
550	EEEEKKQQEQRNNEKEQEEYQRRGRGRGHQP	-47.86	35.5	HHHHHHHHHHHHHHHHHHHHHHHHHCCCCCCC
601	EEEERKQQQQRNNDREDREYQQRGRGKGHQP	-47.85	35.5	HHHHHHHHHHHHHHHHHHHHHHHHHCCCCCCC
089	EEEQRKQEQQRNNDKEDRRYQREGKGKGHNH	-47.85	35.5	HHHHHHHHHHHHHHHHHHHHHHHHCCCCCCCC
660	EEEQRKQEQQRNNDKEDRRYQRYGRGKGHQP	-47.85	35.5	HHHHHHHHHHHHHHHHHHHHHHHHHCCCCCCC
220	EEEYRKWLDQKKNEEEDQRYQKQGRGKGHQP	-47.83	41.9	HHHHHHHHHHHHHHHHHHHHHHHHCCCCCCCC
469	EEEQRKQQDQYSQEYEQQEYQKEGRGQGHQP	-47.82	32.3	HHHHHHHHHHHHHHHHHHHHHHHHHCCCCCCC
289	DEERKKMLDQIKNEQEDQRYQREGRGKGHQP	-47.79	41.9	HHHHHHHHHHHHHHHHHHHHHHHHHCCCCCCC
484	DEERKKMLDQYKNEYEDEYYQQNGSGKGHQY	-47.79	41.9	HHHHHHHHHHHHHHHHHHHHHHHHCCCCCCCC
199	EEEERKMRDQYNNEAEDERYQRKGMGKGHQP	-47.79	45.2	HHHHHHHHHHHHHHHHHHHHHHHHHHCCCCCC
923	EEEERKMRDQYNNEFEDQYYQKEGQGKGHSH	-47.79	41.9	HHHHHHHHHHHHHHHHHHHHHHHHCCCCCCCC
766	EEEKKKMLDQMKNEKEDEEYQRYGRGKGHQP	-47.79	45.2	HHHHHHHHHHHHHHHHHHHHHHHHHHCCCCCC
978	EEEKKKMLDQWKNEWEDQFYQKQGRGKGHQP	-47.79	48.4	HHHHHHHHHHHHHHHHHHHHHHHHHCCCCCCC
608	EEEKKKMLDQYKNEWEDEEYQRKGRGKGHQP	-47.79	45.2	HHHHHHHHHHHHHHHHHHHHHHHHHCCCCCCC
722	EEEKKKMLDQYKNEYEDEQYQQQGQGKGHQS	-47.79	45.2	HHHHHHHHHHHHHHHHHHHHHHHHHCCCCCCC
997	EEEKRKMLDQIKNEKEDQEYQKQGRGKGHQP	-47.79	41.9	HHHHHHHHHHHHHHHHHHHHHHHHHCCCCCCC
960	EEEKRKMLDQMKNEKEDEEYQRRGKGKGHSH	-47.79	41.9	HHHHHHHHHHHHHHHHHHHHHHHHHCCCCCCC
639	EEEKRKMLDQMKNEKEDKEYQKQGRGKGHQP	-47.79	41.9	HHHHHHHHHHHHHHHHHHHHHHHHHCCCCCCC
647	EEEKRKMLDQMKNEKEDQEYQKQGRGKGHQP	-47.79	41.9	HHHHHHHHHHHHHHHHHHHHHHHHHCCCCCCC
008	EEEKRKMLDQYNNESEDKRYQRYGRGKGHQP	-47.79	45.2	HHHHHHHHHHHHHHHHHHHHHHHHHCCCCCCC
553	EEERRKQLDQIKNDKEDRDYQREGKGKGHNH	-47.78	38.7	HHHHHHHHHHHHHHHHHHHHHHHHCCCCCCCC
064	EEERRKQLDQIKNDQEDRRYQREGRGKGHQP	-47.78	38.7	HHHHHHHHHHHHHHHHHHHHHHHHHCCCCCCC
514	EEERRKQLDQKNNDEEDRRYQRYGQGKGHQS	-47.78	41.9	HHHHHHHHHHHCCHHHHHHHHHHHHHCCCCCC
246	EEERKKQLDQLKNRQEDERYQREGKGKGHNH	-47.78	41.9	HHHHHHHHHHHHHHHHHHHHHHHHCCCCCCCC
545	EEEQRKQEQQYRNEWEDERYQRQGRGKGHQP	-47.78	35.5	HHHHHHHHHHHHHHHHHHHHHHHHHCCCCCCC
908	EEEQRKQQQQYSNEWEDEEYQRKGRGKGHNP	-47.78	35.5	HHHHHHHHHHHHHHHHHHHHHHHHHCCCCCCC
113	EEEQRKQEQQRNNEKEDKRYQREGKGKGHSS	-47.78	38.7	HHHHHHHHHHHHHHHHHHHHHHHHCCCCCCCC
192	EEEEKKKRDAKNNREEDERYQQQGRGKGHQP	-47.77	41.9	CHHHHHHHHHHHHHHHHHHHHHHHHCCCCCCC
005	EEEERKKQDAYNNRAEDQRYQREGWGKGHYP	-47.77	41.9	HHHHHHHHHHHHHHHHHHHHHHHHHCCCCCCC
886	EEEKRKQEQQRNEEAEDQRYQREGKGKGHNH	-47.77	41.9	HHHHHHHHHHHHHHHHHHHHHHHHHCCCCCCC
752	EEERKKQLDQINNEKEDEEYQRYGRGKGHQP	-47.77	48.4	HHHHHHHHHHHHHHHHHHHHHHHHHHCCCCCC
293	EEERKKQLDQYKNEWEDEEYQRKGRGKGHQP	-47.77	45.2	HHHHHHHHHHHHHHHHHHHHHHHHHCCCCCCC
215	EEERRKQLDQEKNEKEDERYQQQGKGKGHSS	-47.77	41.9	HHHHHHHHHHHHHHHHHHHHHHHHCCCCCCCC
360	EEERRKQLDQEKNEKEDQRYQREGRGKGHQP	-47.77	41.9	HHHHHHHHHHHHHHHHHHHHHHHHHCCCCCCC
070	EEERRKQLDQIKNEKEDEEYQRRGQGKGHQS	-47.77	41.9	HHHHHHHHHHHHHHHHHHHHHHHHHHCCCCCC
826	EEERRKQLDQWKNEFEDKYYQEYGQGKGHQP	-47.77	41.9	HHHHHHHHHHHHHHHHHHHHHHHHHHCCCCCC
507	EEERRKQLDQWKNEYEDEEYQRKGRGKGHQP	-47.77	41.9	HHHHHHHHHHHHHHHHHHHHHHHHHCCCCCCC
077	EEERRKQLDQYKNEYEDQEYQRQGRGKGHQP	-47.77	41.9	HHHHHHHHHHHHHHHHHHHHHHHHHCCCCCCC
163	EEERRKQLDQYNNEYEDEEYQRKGRGKGHNP	-47.77	45.2	HHHHHHHHHHHHHHHHHHHHHHHHCCCCCCCC
729	EEEERKYQDQRNNEEEDQRYQREGRGKGHQP	-47.77	41.9	CHHHHHHHHHHHHHHHHHHHHHHHHCCCCCCC
340	EQEERKYQDQYNNEAEDKRYQREGKGKGHSH	-47.77	41.9	CHHHHHHHHHHHHHHHHHHHHHHHCCCCCCCC
672	EREQRYQQDQRSNEKEDEEYQRRGQGKGNSN	-47.75	35.5	HHHHHHHHHHHHHHHHHHHHHHHHHCCCCCCC
505	EEEQRKLEEKKSNEQEDKRYQRYGRGKGHQP	-47.75	38.7	HHHHHHHHHHHHHHHHHHHHHHHHHCCCCCCC
827	EQEERKRQEQYNNDWEDKRYQREGDGKGHQP	-47.75	32.3	HHHHHHHHHHHHHHHHHHHHHHHCCCCCCCCC
347	EEEERKQRQQRENEREDQEYQRYGRGKGHQP	-47.75	35.5	HHHHHHHHHHHHHHHHHHHHHHHHHHCCCCCC
571	EEEERKQRDKFNNEKEQQRYQQQGKGQGHSS	-47.73	41.9	HHHHHHHHHHHHHHHHHHHHHHHHCCCCCCCC
261	EEEERKKQDQYNNEWEDEEYQRKGRGKGDQR	-47.73	48.4	HHHHHHHHHHHHHHHHHHHHHHHHCCCCCCCC
100	EEEQKKQQDQKNNDEEHRRYQQYGRGQGHQP	-47.73	35.5	HHHHHHHHHHHCHHHHHHHHHHHHHHCCCCCC
535	EEQEKKKREKYNNEFEDKYYQQKGQGKGHQP	-47.72	48.4	HHHHHHHHHHHHHHHHHHHHHHHHHCCCCCCC
648	EEERRKQLQQYKNRYEDEEYQRQGRGKGHQP	-47.72	35.5	HHHHHHHHHHHHHHHHHHHHHHHHHCCCCCCC
227	EEERRKQQEQYSNRYEDEEYQREGRGKGHSR	-47.72	35.5	HHHHHHHHHHHHHHHHHHHHHHHHCCCCCCCC
651	EEEERKKQDQIKNEKEDQEYQRSGKGKGHNH	-47.71	41.9	HHHHHHHHHHHHHHHHHHHHHHHCCCCCCCCC
868	EEEERKQRDQEKNDKEDRRYQREGKGKGHSH	-47.70	35.5	HHHHHHHHHHHHHHHHHHHHHHHHCCCCCCCC
715	DEERKKQEDQERNEKEDEEYQRYGWGKGHNP	-47.69	38.7	CHHHHHHHHHHHHHHHHHHHHHHHCCCCCCCC
779	DEERKKQHDQESNEKEDKRYQREGKGKGHNH	-47.69	38.7	HHHHHHHCHHHHHHHHHHHHHHHHCCCCCCCC
394	EEEQRKQYDQEKNEKEDERYQQQGWGKGHNP	-47.69	38.7	HHHHHHHHHHHHHHHHHHHHHHHHCCCCCCCC
398	EEEERKQRQQYNNRAEDERYQREGRGKGHQP	-47.69	38.7	HHHHHHHHHHHHHHHHHHHHHHHHHCCCCCCC
541	EEEEKKKQDQYNNEFEDEYYQQYGQGKGDSR	-47.68	51.6	CHHHHHHHHHHHHHHHHHHHHHHHHHCCCCCC
086	EEERKKQLEQYKNEWEDEEYQRKGRGKGHQP	-47.68	41.9	HHHHHHHHHHHHHHHHHHHHHHHHHCCCCCCC
866	EEERKKQLEQYKNEWEDKRYQREGRGKGHQP	-47.68	41.9	HHHHHHHHHHHHHHHHHHHHHHHHHCCCCCCC
093	EEEERKQQDQRNHEKEQERYQQQGRGQGHQP	-47.67	38.7	HHHHHHHHHHHHHHHHHHHHHHHHHCCCCCCC
846	EEEQRKFYDQYNNEYEDQEYQRKGRGKGHQP	-47.67	45.2	HHHHHHHHHHHHHHHHHHHHHHHHHCCCCCCC
278	EEEQKKQRDKFNNEFEQKFYQKEGRGQGHQP	-47.67	48.4	HHHHHHHHHHHHHHHHHHHHHHHHHCCCCCCC
461	EEEERKNQEKYSNEWEDKRYQREGRGKGHQP	-47.66	38.7	HHHHHHHHHHHHHHHHHHHHHHHHHCCCCCCC
744	EEERRKQLDQWKNEWEDQYYQQQGQGKGHSS	-47.66	41.9	HHHHHHHHHHHHHHHHHHHHHHHHHCCCCCCC
716	EEEQRKQQDQYNNEWEHEYYQRKGQGQGHQH	-47.66	35.5	HHHHHHHHHHHHHHHHHHHHHHHHHCCCCCCC
357	DEQRKKMLDQYKNEYEDEEYQRQGRGKGHQP	-47.66	45.2	HHHHHHHHHHHHHHHHHHHHHHHHHCCCCCCC

479	EEEERKQRQQYNNEAEDQRYQREGRGKGHQP	-47.65	41.9	HHHHHHHHHHHHHHHHHHHHHHHHHCCCCCCC
977	EEEERKQRQQYNNESEDERYQQQGKGKGHNH	-47.65	38.7	HHHHHHHHHHHCCCHHHHHHHHHHHCCCCCCC
579	EEEERKQRQQYNNEYEDEEYQRKGRGKGHQP	-47.65	38.7	HHHHHHHHHHHHHHHHHHHHHHHHHCCCCCCC
720	EEEKRKQEQQRNNEEEDERYQRQGRGKGHQP	-47.65	38.7	HHHHHHHHHHHHHHHHHHHHHHHHHCCCCCCC
180	EEERRKQEQQYRNEFEDQYYQQKGQGKGHSH	-47.65	35.5	HHHHHHHHHHHHHHHHHHHHHHHHHCCCCCCC
123	EEERRKQLQQKNNEEEDQRYQRYGWGKGHNP	-47.65	41.9	HHHHHHHHHHHHHHHHHHHHHHHHCCCCCCCC
257	EEEQRKQEDKRRNEKEQKRYQREGKGQGHSH	-47.65	35.5	HHHHHHHHHHHHHHHHHHHHHHHHHCCCCCCC
374	EEEQRKLQDAINNRAEDQRYQREGRGKGHQP	-47.64	41.9	HHHHHHHHHHHHHHHHHHHHHHHHHCCCCCCC
462	EEEQRKHHEKESNEREDEEYQRKGRGKGHQP	-47.62	38.7	HHHHHHHHHHHHHHHHHHHHHHHHHCCCCCCC
037	EEEQRKQQQQYNHDWEDRRYQREGWGKGHNP	-47.61	38.7	HHHHHHHHHHHHCHHHHHHHHHHHHCCCCCCC
141	EEEQKKQQEQYSNESEDQRYQRYGQGKGHQS	-47.61	38.7	HHHHHHHHHHHHCHHHHHHHHHHHHHCCCCCC
927	EQEQRKQQEQYNNESEDERYQQQGRGKGHQP	-47.61	35.5	HHHHHHHHHHHHCHHHHHHHHHHHHCCCCCCC
967	EEEERKQQQQRNNEKEDQRYQREGKGKGHNN	-47.58	38.7	HHHHHHHHHHHHHHHHHHHHHHHHCCCCCCCC
154	EEEERKQQQQRNNEKEDQRYQREGKGKGHSN	-47.58	38.7	HHHHHHHHHHHHHHHHHHHHHHHHCCCCCCCC
795	EEEERKKQQKKNNEEEDKRYQREGWGKGHNP	-47.58	41.9	HHHHHHHHHHHHHHHHHHHHHHHHHCCCCCCC
039	EEEQRKYEDQRNNEEEDQRYQRYGKGKGDSR	-47.55	48.4	CHHHHHHHHHHHHHHHHHHHHHHHCCCCCCCC
876	EQEERKKQEQYKNEWEDKRYQREGKGKGHNH	-47.53	32.3	HHHHHHHHHHHHHHHHHHHHHHHCCCCCCCCC
430	EEERRKQLDQYNNESEDQRYQRYGRGKGHQP	-47.53	45.2	HHHHHHHHHHHHCHHHHHHHHHHHHCCCCCCC
620	EEEERKKQQKFSNEAEDEEYQRRGKGKGHSS	-47.53	45.2	HHHHHHHHHHHHHHHHHHHHHHHHHCCCCCCC
381	EEEQKKQRDQYNNEAEQKRYQREGKGQGHNN	-47.52	41.9	HHHHHHHHHHCHHHHHHHHHHHHHCCCCCCCC
622	EEEQRKQQDQYNNEAEQQRYQREGRGQGHQP	-47.52	38.7	HHHHHHHHHHHHHHHHHHHHHHHHHCCCCCCC
940	EEEQRKQQDQYNNEHEQERYQKQGWGQGHNP	-47.52	35.5	HHHHHHHHHHHHHHHHHHHHHHHHCCCCCCCC
949	EEEQRKQQDQYNNESEQKRYQKEGKGQGHSS	-47.52	35.5	HHHHHHHHHHCHHHHHHHHHHHHHCCCCCCCC
021	EEEQRKQQDQYNNEWEQKRYQREGKGQGHNH	-47.52	35.5	HHHHHHHHHHHHHHHHHHHHHHHHHCCCCCCC
300	EEEQRKQQDQYNNEYEQEEYQRQGRGQGHQP	-47.52	35.5	HHHHHHHHHHHHHHHHHHHHHHHHHCCCCCCC
631	EEEQRKQEQKRRNEKEDRRYQKEGRGKGHQP	-47.51	38.7	HHHHHHHHHHHHHHHHHHHHHHHHHCCCCCCC
242	EEEKRKFLDKMKNEAEDRRYQRYGRGKGHQP	-47.51	51.6	HHHHHHHHHHHHHHHHHHHHHHHHHCCCCCCC
150	EEEERKRQDQYNNDSEDRRYQRYGRGKGHQP	-47.49	38.7	HHHHHHHHHCCCCHHHHHHHHHHHHCCCCCCC
754	EEEEKKRQDQYNNEWEDEEYQRKGRGKGHQP	-47.48	45.2	CHHHHHHHHHHHHHHHHHHHHHHHHCCCCCCC
982	EEEERKRQDQYNNEWEDKRYQEEGRGKGHQP	-47.48	41.9	HHHHHHHHHHHHHHHHHHHHHHHHHCCCCCCC
391	EEEERKRQDQYNNEWEDKRYQREGRGKGHQP	-47.48	41.9	HHHHHHHHHHHHHHHHHHHHHHHHHCCCCCCC
619	EQEERKRQDQYNNEWEDEEYQRKGRGKGHQP	-47.48	38.7	HHHHHHHHHHHHHHHHHHHHHHHHHCCCCCCC
528	EEEERKKQEQYSNDYEDEEYQRKGRGKGHQP	-47.48	32.3	HHHHHHHHHHHHHHHHHHHHHHHHHCCCCCCC
472	QDQQRKQQDHYNNRWEDEEYQRKGRGKGHQP	-47.47	35.5	CHHHHHHHHHHHCHHHHHHHHHHHHCCCCCCC
513	EEERRKQLDQQNNDEEQRRYQQYGWGQGHYP	-47.46	35.5	HHHHHHHHHHHCCHHHHHHHHHHHHCCCCCCC
992	EEEERKQRQQFNNEFEDQYYQQKGQGKGHSH	-47.46	41.9	HHHHHHHHHHHHHHHHHHHHHHHHHCCCCCCC
259	EEERRKQLDQYNNRSEQERYQKQGKGQGHSN	-47.43	35.5	HHHHHHHHHHHHHHHHHHHHHHHHCCCCCCCC
447	EEEERKQKQKWKNEWEDKFYQKEGKGKGDSR	-47.41	48.4	CHHHHHHHHHHHHHHHHHHHHHHHCCCCCCCC
390	EEEERKRQDQEKNRKEDERYQREGRGKGHQP	-47.41	35.5	HHHHHHHHHHHHHHHHHHHHHHHHHCCCCCCC
103	EEEKRKQEDQRNNEKEQEEYQRRGKGQGHSS	-47.40	35.5	HHHHHHHHHHHHHHHHHHHHHHHHHCCCCCCC
304	EEEERKQQDQYKNEWEQEYYQRKGQGQGHSH	-47.39	32.3	HHHHHHHHHHHHHHHHHHHHHHHHHCCCCCCC
856	EEEERKQQDQYNNEWEQEEYQRKGRGQGHQP	-47.39	35.5	HHHHHHHHHHHHHHHHHHHHHHHHHCCCCCCC
577	EEEERKQRDQYNNESEQKRYQRYGQGQGHQS	-47.39	35.5	HHHHHHHHHHCHHHHHHHHHHHHHHHCCCCCC
332	EEEERKQRDQYNNEWEQEEYQRKGRGQGHQP	-47.39	35.5	HHHHHHHHHHHHHHHHHHHHHHHHHCCCCCCC
646	EEERRKQLDQYNNEHEQQRYQREGKGQGHNH	-47.39	38.7	HHHHHHHHHHHHHHHHHHHHHHHHHCCCCCCC
542	EEEKKKQEDQRNNEEEQRRYQRYGRGQGHQP	-47.37	38.7	HHHHHHHHHHHHHHHHHHHHHHHHHHCCCCCC
676	EEEQKKQREQREDEKRDQEYQRSGRGKGHQP	-47.36	38.7	HHHHHHHHHHHHHHHHHHHHHHHHCCCCCCCC
302	EEEERKQQDQRKNRKEQEEYQRRGRGQGHQP	-47.36	29.0	HHHHHHHHHHHHHHHHHHHHHHHHHHCCCCCC
749	EEERRKQLQQYKQESEDQRYQRYGRGKGHQP	-47.34	38.7	HHHHHHHHHHHHHHHHHHHHHHHHHCCCCCCC
371	EEQRKKQLDQKNNEEEDKRYQKEGWGKGHYP	-47.33	51.6	HHHHHHHHHHHHHHHHHHHHHHHHHCCCCCCC
878	EEEKRKQEQQQNNEKEDSRYQQQGWGKGHNP	-47.32	38.7	HHHHHHHHHHHHHHHHHHHHHHHHCCCCCCCC
897	EEEEKKYQEQYNNDYEDEEYQRKGRGKGHQP	-47.29	38.7	CHHHHHHHHHHHCCCHHHHHHHHHHCCCCCCC
972	EQEQRKYEEQRNNDEEDQRYQREGHGKGHQR	-47.29	35.5	HHHHHHHHHHHHHHHHHHHHHHHHHCCCCCCC
990	EEEEKKYQEKYKNEFEDKYYQQKGQGKGHQP	-47.29	41.9	CHHHHHHHHHHHHHHHHHHHHHHHHCCCCCCC
401	EEEERKYQEKYNNESEDKRYQQQGRGKGHQP	-47.29	41.9	CHHHHHHHHHHHHHHHHHHHHHHHHCCCCCCC
073	EQEERKYQEKRNNEEEDKRYQRYGWGKGHYP	-47.29	38.7	CHHHHHHHHHHHHHHHHHHHHHHHCCCCCCCC
450	EQEQRHQQEKFSNEFEDQYYQKYGQGKGHQP	-47.27	38.7	HHHHHHHHHHHHHHHHHHHHHHHHHHCCCCCC
311	EQEQRHQQEKYSNEYEDEQYQRKGKGKGHSH	-47.27	35.5	HHHHHHHHHHHHHHHHHHHHHHHCCCCCCCCC
527	EEEEKKRQDQENNEREDKRYQREGKGKGHSH	-47.25	45.2	CHHHHHHHHHHHHHHHHHHHHHHHCCCCCCCC
327	EQEERKFRDQYNNDYEHEEYQRKGRGQGHQP	-47.24	32.3	CHHHHHHHHHHCHHHHHHHHHHHHHCCCCCCC
399	EEEERKQQDDYNNRWEDQRYQREGKGKGHSH	-47.24	38.7	HHHHHHHHHHHHHHHHHHHHHHHHCCCCCCCC
145	EEERRKQLDDYKNRSEDQRYQRYGRGKGHQP	-47.24	38.7	HHHHHHHHHHHCCCHHHHHHHHHHHCCCCCCC
930	EQEERKKEEQFRNDFEDRRYQQEGRGKGHQP	-47.23	32.3	HHHHHHHHHHHHHHHHHHHHHHHHHCCCCCCC
517	EEEKRKQLDQYKNRYEQEEYQRKGRGQGHQP	-47.23	32.3	HHHHHHHHHHHHHHHHHHHHHHHHHCCCCCCC
211	EEEMRKLQDKINNEKEQEEYQRRGRGQGHQP	-47.23	38.7	HHHHHHHHHHHHHHHHHHHHHHHHHHCCCCCC
232	EEEEKKKRDKYNNEWEQKRYQREGRGQGHQP	-47.22	41.9	CHHHHHHHHHHHHHHHHHHHHHHHCCCCCCCC
440	EEEERKFQEKWKNEWEDKYYQQKGQGKGHSH	-47.19	41.9	CHHHHHHHHHHHHHHHHHHHHHHHCCCCCCCC

474	EEEERKQQDDRNNRKEDERYQQQGWGKGHNP	-47.17	38.7	HHHHHHHHHHHHHHHHHHHHHHHCCCCCCCC
952	EEEERKQQDDRNNRKEDQRYQREGKGKGHNH	-47.17	38.7	HHHHHHHHHHHHHHHHHHHHHHHCCCCCCCC
221	EEERRKQLEQYKNEWEDEEYQRKGRGKGHQP	-47.16	38.7	HHHHHHHHHHHHHHHHHHHHHHHHCCCCCCC
556	EEEEKKKREQYNNEHEDQRYQQQGRGKGHQP	-47.16	41.9	CHHHHHHHHHHHHHHHHHHHHHHHCCCCCCC
375	EEEEKKKREQYNNEYEDEEYQRKGRGKGHQP	-47.16	41.9	CHHHHHHHHHHHHHHHHHHHHHHHCCCCCCC
334	EEEERKKEEQKRNEEEDQRYQRYGWGKGHNP	-47.16	35.5	HHHHHHHHHHHHHHHHHHHHHHHCCCCCCCC
981	EEQERKNQQQYNNEYEDEEYQRKGRGKGHNP	-47.16	41.9	HHHHHHHHHHHHHHHHHHHHHHHCCCCCCCC
736	EEEQKKQREQFNNEYEHQYKQQQGQGSGKQS	-47.15	35.5	HHHHHHHHHHHHHHHHHHHHHHHCCCCCCCC
668	EQERRHQLEKYKNEWEDEYYQQYGQGKGHQP	-47.14	38.7	HHHHHHHHHHHHHHHHHHHHHHHHHCCCCCC
209	EEERRKQLDKYKNEYEHEEKQRKGRGSGKQN	-47.14	35.5	HHHHHHHHHHHHHHHHHHHHHHHCCCCCCCC
444	EEEEKKKQEKFSNEFEDRRYQQEGQGKGHQS	-47.14	45.2	HHHHHHHHHHHHHHHHHHHHHHHHCCCCCCC
010	EEQRRKQLQQWKNEYEDQSYQREGRGKGHQP	-47.14	41.9	HHHHHHHHHHHHHHHHHHHHHHHCCCCCCCC
483	EEEQRKQQDQYNNEWEQEEYQRKGRGQGHQP	-47.13	35.5	HHHHHHHHHHHHHHHHHHHHHHHHCCCCCCC
903	EEEQRKQQDQYSNDFEQRYYQQQGQGQGHQS	-47.12	29.0	HHHHHHHHHHHHHHHHHHHHHHHHCCCCCCC
268	EQEERKQQEQYEDEYRDEQYQQQGQGKGHQS	-47.11	29.0	HHHHHHHHHHHHHHHHHHHHHHHHCCCCCCC
581	EQEQRHQQEQYNNDYEDEEYQRKGRGKGHQP	-47.11	32.3	HHHHHHHHHHHHCCCHHHHHHHHHCCCCCCC
282	EQEERHNQEKFNNEYEDKSYQREGRGKGHQP	-47.09	41.9	CHHHHHHHHHHHHHHCHHHHHHCCCCCCCCC
389	EQEERHQQEKRSNEKEDKEYQKQGRGKGHQP	-47.08	35.5	CHHHHHHHHHHHHHHHHHHHHHHHCCCCCCC
794	EEEERKWREQKNNDEEDKRYQRYGRGKGHQP	-47.07	35.5	CHHHHHHHHHHCHHHHHHHHHHHHCCCCCCC
612	EEEERKQQQQRNNEEEDQRYQREGKGKGHSS	-47.07	38.7	HHHHHHHHHHHHHHHHHHHHHHHCCCCCCCC
019	EEEKKKWLEKLNNEKEDKRYQREGRGKGHQP	-47.06	48.4	HHHHHHHHHHHHHHHHHHHHHHHHCCCCCCC
641	EEEARKQQEQYSNEHEDQRYQRQGKGKGDSR	-47.04	45.2	HHHHHHHHHHHHHHHHHHHHHHHCCCCCCCC
863	EEERRKQQQQYSNEYEDQYYQQKGQGKGHQP	-47.04	35.5	HHHHHHHHHHHHHHHHHHHHHHHHCCCCCCC
065	EEEERKYQDQKNNEEEDQRYQRYGKGKGDSR	-47.04	48.4	CHHHHHHHHHHHHHHHHHHHHHHCCCCCCCC
637	EEEERKQRDQYNQEAEQERYQQQGKGQGHNH	-47.03	38.7	HHHHHHHHHHHHHHHHHHHHHHHHCCCCCCC
798	EEEQKKQRDQFNQEKEQERYQQQGRGQGHQP	-47.02	41.9	HHHHHHHHHHHHHHHHHHHHHHHCCCCCCCC
602	EEEKRKFLQKMEQEREDKEYQQKGRGKGHQP	-47.01	45.2	HHHHHHHHHHHHHHHHHHHHHHHHCCCCCCC
769	EEEEKKKRDQQKNEEEHERYQQQGKGQGHNH	-47.01	35.5	CHHHHHHHHHHHHHHHHHHHHHHHCCCCCCC
168	EEEERKKQDQINNEAEDKRYQREGRGKGNQN	-47.00	45.2	HHHHHHHHHHHHHHHHHHHHHHHHCCCCCCC
365	QDEERKQQDQRNNDNEQRQYQRSGRGQGHQP	-47.00	29.0	CHHHHHHHHHHHHHHHHHHHHHHCCCCCCCC
412	EEEKKKLLEQISNDKEDREYQRRGRGKGHQP	-47.00	38.7	HHHHHHHHHHHHHHHHHHHHHHHHCCCCCCC
205	EEEQRKLYEQISNEKEDQEYQKQGRGKGHQP	-46.99	35.5	HHHHHHHHHHHHHHHHHHHHHHHHCCCCCCC
506	EEEEKKQRDQQNNEKEQKRYQREGRGQGHQP	-46.98	38.7	HHHHHHHHHHHHHHHHHHHHHHHHCCCCCCC
284	EQEERHQQEQYNNDWEDQRYQREGWGKGHNP	-46.98	32.3	CHHHHHHHHHHHCHHHHHHHHHHHCCCCCCC
319	EEEQRKQEQQWQNEWEDQYYQQKGQGKGHNH	-46.97	35.5	HHHHHHHHHHHHHHHHHHHHHHHHCCCCCCC
169	EEEERKKRQQYSNDFEDRYYQQKGQGKGHNH	-46.97	32.3	CHHHHHHHHHHHHHHHHHHHHHHHCCCCCCC
850	EEEEKKKREQYNNEAEDQRYQREGRGKGHQP	-46.96	45.2	CHHHHHHHHHHHHHHHHHHHHHHHCCCCCCC
838	EEEERKREDQFRQEFEDEYYQQKGQGKGHNH	-46.95	41.9	HHHHHHHHHHHHHHHHHHHHHHHHCCCCCCC
456	EEEERKKQEKWKNEFEDKYYQQKGQGKGHSH	-46.94	38.7	HHHHHHHHHHHHHHHHHHHHHHHHCCCCCCC
778	EEERRKQLQKMKNEKEHEEYQRRGRGQGHQP	-46.93	35.5	HHHHHHHHHHHHHHHHHHHHHHHHHCCCCCC
174	EEERKKQLDQYNNESEQQRYQKQGKGQGHSS	-46.92	41.9	HHHHHHHHHHHHCHHHHHHHHHCCCCCCCC
837	EEERRKQLDQMKNEKEQEEYQRYGRGQGHQP	-46.92	35.5	HHHHHHHHHHHHHHHHHHHHHHHHHCCCCCC
090	EEEERKRQEKENNEKEDKRYQREGRGKGHQP	-46.91	41.9	HHHHHHHHHHHHHHHHHHHHHHHHCCCCCCC
309	EEEERKRQEKENNEKEDKRYQRYGKGKGHNS	-46.91	41.9	HHHHHHHHHHHHHHHHHHHHHHHCCCCCCCC
650	EEEEKKNREQYNNESEDQRYQRYGKGKGHSS	-46.90	41.9	HHHHHHHHHHHCCCHHHHHHHHHCCCCCCCC
234	EEEERKNQEQFSNEFEDQYYQQKGQGKGHQP	-46.90	38.7	HHHHHHHHHHHHHHHHHHHHHHHHCCCCCCC
963	EEEERKKQDKFNNEFEQKFYQQKGRGQGHQP	-46.82	45.2	HHHHHHHHHHHHHHHHHHHHHHHHCCCCCCC
362	EEEERKQRQQYNNEWEDKRYQREGKGKGHSH	-46.81	38.7	HHHHHHHHHHHHHHHHHHHHHHCCCCCCCCC
276	EQEKRKMLEKISNEKEDEEYQRRGQGKGHQS	-46.79	38.7	HHHHHHHHHHHHHHHHHHHHHHHHHCCCCCC
597	EEEQRKQQDQYNNDYEQREYQRKGRGQGHQP	-46.79	32.3	HHHHHHHHHHHHHHHHHHHHHHHHCCCCCCC
815	EEEEKKQRDQFNNEYEQQSYQREGRGQGHQP	-46.78	41.9	HHHHHHHHHHHHHHHHHHHHHHHCCCCCCCC
191	EEEKRKQEDQRNNEKEQEEYQRYGRGQGHQP	-46.78	35.5	HHHHHHHHHHHHHHHHHHHHHHHHHCCCCCC
015	EEERRKQLEKISNEEEDKEYQRQGRGKGHQP	-46.77	41.9	HHHHHHHHHHHHHHHHHHHHHHHHCCCCCCC
942	EEERRKQLEKYNNESEDKRYQRYGRGKGHQP	-46.77	45.2	HHHHHHHHHHHHHHHHHHHHHHHHCCCCCCC
237	EQERRKQLEKESNEKEDKSYQRHGWGKGHNP	-46.77	38.7	HHHHHHHHHHHHHHHHHHHHHHCCCCCCCCC
825	EEERRKQLDKESNEKEHKRYQREGRGQGHQP	-46.76	38.7	HHHHHHHHHHHHHHHHHHHHHHHHCCCCCCC
580	EEERRKQLDKINNEQEHKRYQREGWGQGHYP	-46.76	41.9	HHHHHHHHHHHHHHHHHHHHHHHHCCCCCCC
902	EQQQRKQQEKYNNEWEQKRYQREGRGQGHQP	-46.76	35.5	HHHHHHHHHHHHHHHHHHHHHHHHCCCCCCC
431	EEEQRKQEEKRNNEKEQKRYQKEGWGQGHNP	-46.76	35.5	HHHHHHHHHHHHHHHHHHHHHHHHCCCCCCC
161	EEEQRRQEDQRNNEEEQKRYQREGRGQGHQP	-46.75	35.5	HHHHHHHHHHHHHHHHHHHHHHHHCCCCCCC
361	EEEQKKQRDQYNNEWEQERYQQQGRGQGHQP	-46.72	38.7	HHHHHHHHHHHHHHHHHHHHHHHHCCCCCCC
114	EQEERKKQEQYNNDWEDRRYQREGRGKGHQP	-46.70	32.3	HHHHHHHHHHHHHHHHHHHHHHHHCCCCCCC
016	EEEQRKQQDDYNHRWEDQRYQREGRGKGHQP	-46.68	41.9	HHHHHHHHHHHHHHHHHHHHHHHHCCCCCCC
604	QQEEEKRQRQYNEEYEHEEYQRKGRGRGHQP	-46.66	25.8	CHHHHHHHHHHHHHHHHHHHHHHHCCCCCCC
936	EEERRKQLDQYKNEFEQQYYQQKGQGQGHQP	-46.65	35.5	HHHHHHHHHHHHHHHHHHHHHHHHCCCCCCC
249	EQEQRHQQEKYNNEWEDKRYQREGQGKGHQS	-46.65	38.7	HHHHHHHHHHHHHHHHHHHHHHHHCCCCCCC
563	EEEERKYQQKYNNEEEDKKYQKEGRGKGHQP	-46.65	41.9	CHHHHHHHHHHHHHHHHHHHHHHHCCCCCCC

283	EEEERKYQQKYNNESEDKRYQQQGRGKGHQP	-46.65	41.9	CHHHHHHHHHHHHHHHHHHHHHHHHCCCCCCC
854	EEEERKQQDAEKNRKEDQRYQREGRGKGHQP	-46.65	35.5	HHHHHHHHHHHHHHHHHHHHHHHHHCCCCCCC
809	EEEERKRQDQYNNEYEDEEYQRKGRGKGDSR	-46.64	48.4	HHHHHHHHHHHHHHHHHHHHHHHHCCCCCCCC
468	EEEQKKYEDKRNQEEEQKRYQQQGWGQGHNP	-46.64	41.9	CHHHHHHHHHHHHHHHHHHHHHHHCCCCCCCC
126	EEEERKQRDDYNNRWEDERYQRQGRGKGHQP	-46.63	38.7	HHHHHHHHHHHHHHHHHHHHHHHHHCCCCCCC
346	EEEEKKQREKFSNEFEQKYYQQKGQGQGHQP	-46.63	38.7	CHHHHHHHHHHHHHHHHHHHHHHHHCCCCCCC
662	EEEERKKQDKYNNEAEQKRYQREGKGQGHSH	-46.62	41.9	HHHHHHHHHHHHHHHHHHHHHHHHHCCCCCCC
983	EQEQRKQQDQYNNEYEQEQYQRQGRGQGNQN	-46.58	32.3	HHHHHHHHHHHHHHHHHHHHHHHHCCCCCCCC
880	EEEERKKQQQWKNEWEDKYYQQQGQGKGHSH	-46.58	35.5	HHHHHHHHHHHHHHHHHHHHHHHHHCCCCCCC
492	EEERKKQLDQIKNEKEHEEKQRRGKGSGKSN	-46.57	35.5	HHHHHHHHHHHHHHHHHHHHHHHHCCCCCCCC
758	EQEQRKQEEQRNNEEEHKRYQQQGWGQGHNP	-46.57	29.0	HHHHHHHHHHHHHHHHHHHHHHHHCCCCCCCC
201	EQEQRHQQEQYNQEAEDQRYQREGKGKGHSH	-46.57	38.7	HHHHHHHHHHHHHHHHHHHHHHHHCCCCCCCC
367	EEEERKYRDQKNNEEEHERYQQQGRGQGHQP	-46.56	35.5	CHHHHHHHHHCHHHHHHHHHHHHHCCCCCCCC
240	EEEQRKYEDQRNNEEEHEEYQRKGRGQGHQP	-46.56	35.5	CHHHHHHHHHCHHHHHHHHHHHHHCCCCCCCC
142	EEEERKKQEQQSNEKEDEEYQRRGWGKGHYP	-46.56	35.5	HHHHHHHHHHHHHHHHHHHHHHHHCCCCCCCC
435	EEEERKKQEQYNNEWEDEEYQRKGRGKGHQP	-46.56	38.7	HHHHHHHHHHHHHHHHHHHHHHHHHCCCCCCC
414	EEEERKSRDQQKNEKEHEEKQRRGQGSGKQS	-46.56	29.0	CHHHHHHHHHHHHHHHHHHHHHHHCCCCCCCC
503	EEEEKKRQDQYNNEAEDQRYQREGKGKGNSN	-46.55	48.4	CHHHHHHHHHHHHHHHHHHHHHHHCCCCCCCC
764	EEERRKQLDQKNNDEEDRRYQQYGKGKGDSR	-46.54	48.4	HHHHHHHHHHCCHHHHHHHHHHHHCCCCCCCC
649	EEERRKQLDMIKNRKEQEEYQRYGRGQGHQP	-46.54	32.3	HHHHHHHHHHHHHHHHHHHHHHHHHHCCCCCC
701	EEEERKKEQQYRNEYEDEEYQRKGRGKGHQP	-46.52	35.5	HHHHHHHHHHHHHHHHHHHHHHHHHCCCCCCC
810	EDERRKQLDQKNNEEEQQRYQRYGRGQGHQP	-46.52	35.5	HHHHHHHHHHCHHHHHHHHHHHHHHHCCCCCC
096	EEEEKKYQEQFNNEFEDQYYQQQGRGKGHQP	-46.52	45.2	CHHHHHHHHHHHHHHHHHHHHHHHHCCCCCCC
611	EEEEKKYQEQYNNEWEDKRYQREGRGKGHQP	-46.52	41.9	CHHHHHHHHHHHHHHHHHHHHHHHHCCCCCCC
693	EEEERKYQEQYKNEYEDEEYQRKGRGKGHQP	-46.52	35.5	CHHHHHHHHHHHHHHHHHHHHHHHHCCCCCCC
009	EEEERKYQEQYNNEYEDEEYQRKGRGKGHQP	-46.52	38.7	CHHHHHHHHHHHHHHHHHHHHHHHHCCCCCCC
356	EEEQKKYEEQRNNEAEDKRYQRYGRGKGHQP	-46.52	45.2	HHHHHHHHHHHHHHHHHHHHHHHHHCCCCCCC
152	EEEERKLRQQIENEREDKYYQQKGQGKGHSH	-46.51	35.5	HHHHHHHHHHHHHHHHHHHHHHHHHCCCCCCC
376	EEERRKQLDQFNNEFEHKYKQQKGQGSGKSH	-46.50	38.7	HHHHHHHHHHHHHHHHHHHHHHHHCCCCCCCC
128	EEEQKKQQDQYNNEAEDQRYQRYGRGKGHQP	-46.50	48.4	HHHHHHHHHHHHHHHHHHHHHHHHHCCCCCCC
824	EEEERKQRDQYNNEFEQQYYQQKGQGQGHNH	-46.49	35.5	HHHHHHHHHHHHHHHHHHHHHHHHHCCCCCCC
373	EEEEKKRQEKDSNEREDKRYQREGRGKGHQP	-46.48	41.9	HHHHHHHHHHHHHHHHHHHHHHHHHCCCCCCC
828	EEEERKREEKYRNEHEDKRYQRQGWGKGHFP	-46.48	41.9	HHHHHHHHHHHHHHHHHHHHHHHHHCCCCCCC
258	EEQEKKRQEKEKNEKEDKRYQREGRGKGHQP	-46.48	45.2	HHHHHHHHHHHHHHHHHHHHHHHHHCCCCCCC
188	EEEERKKQEQIKQEAEDQRYQREGKGKGHNH	-46.47	38.7	HHHHHHHHHHHHHHHHHHHHHHHHHCCCCCCC
476	EEEERKQQQQKEDEERDQRYQRYGRGKGHQP	-46.47	32.3	HHHHHHHHHHHHHHHHHHHHHHHHHCCCCCCC
007	EEEERKQRQQYEDEWRDERYQREGQGKGHSS	-46.47	32.3	HHHHHHHHHHHHHHHHHHHHHHHHCCCCCCCC
560	EEEAKKFREQYNNEWEDEEYQRKGRGKGHQP	-46.42	48.4	HHHHHHHHHHHHHHHHHHHHHHHHHCCCCCCC
239	EEEERKFQEQYRNEWEDKRYQREGKGKGHNH	-46.42	38.7	CHHHHHHHHHHHHHHHHHHHHHHCCCCCCCCC
006	EEEERKFQEQYRNEYEDEEYQRKGRGKGHQP	-46.42	38.7	CHHHHHHHHHHHHHHHHHHHHHHHHCCCCCCC
408	EEEERKFREQKNNEEEDERYQQQGKGKGHNH	-46.42	41.9	CHHHHHHHHHHHHHHHHHHHHHHHHCCCCCCC
786	EEEQRKFEEQRNNEEEDEEYQRKGRGKGHQP	-46.42	41.9	HHHHHHHHHHHHHHHHHHHHHHHHHCCCCCCC
605	EEEQRKFEEQYRNEYEDQEYQKEGQGKGHQS	-46.42	38.7	HHHHHHHHHHHHHHHHHHHHHHHHHCCCCCCC
682	EEEQRKFYEQFNNEFEDQYYQQKGQGKGHNH	-46.42	45.2	HHHHHHHHHHHHHHHHHHHHHHHHHCCCCCCC
176	EEEKRKLLEQIKNEKEDEEYQRRGQGKGHSS	-46.39	38.7	HHHHHHHHHHHHHHHHHHHHHHHHHHCCCCCC
559	EEQQRKQQDAFSNRFEQEYYQRKGQGQGHNH	-46.37	35.5	HHHHHHHHHHHHHHHHHHHHHHHHHCCCCCCC
101	EEEERKKQEQYNNEAEDERYQRQGRGKGHQP	-46.37	41.9	HHHHHHHHHHHHHHHHHHHHHHHHHCCCCCCC
118	EEEQKKLEDKKKNEQEHRRYQREGKGQGHNH	-46.35	38.7	HHHHHHHHHHHHHHHHHHHHHHHHHCCCCCCC
763	EEEKRKLLQQISNEKEDEEYQRRGWGKGHNP	-46.35	38.7	HHHHHHHHHHHHHHHHHHHHHHHHCCCCCCCC
901	EEERRKQLQQMKDEKRDEEYQRYGQGKGHQS	-46.35	35.5	HHHHHHHHHHHHHHHHHHHHHHHHHHCCCCCC
881	EQEQRHQKEQEKNRREDEEYQRKGRGKGHQP	-46.34	29.0	HHHHHHHHHHHHHHHHHHHHHHHHHCCCCCCC
095	EEEKRKLLDKIKNEKEQKEYQKQGRGQGHQP	-46.33	38.7	HHHHHHHHHHHHHHHHHHHHHHHHHCCCCCCC
193	KQEQEKQYEQEKQEKEDRRYQREGRGKGHQP	-46.33	29.0	HHHHHHHHHHHHHHHHHHHHHHHHHCCCCCCC
140	EEEQRKREQKLRQEKEDKRYQREGRGKGHQP	-46.32	38.7	HHHHHHHHHHHHHHHHHHHHHHHHHCCCCCCC
396	EQEERKWQEQRSNDEEDRRYQRYGRGKGHQP	-46.31	29.0	CHHHHHHHHHHCHHHHHHHHHHHHHCCCCCCC
807	EEEERKQRDQYNNESEQQRYQRYGRGQGHQP	-46.31	35.5	HHHHHHHHHHCHHHHHHHHHHHHHHHCCCCCC
445	EEEERKKQQKINNEAEDKRYQRYGQGKGHQS	-46.30	45.2	HHHHHHHHHHHHHHHHHHHHHHHHHHCCCCCC
814	EEEERKKQQKINNEAEDKRYQRYGRGKGHQP	-46.30	45.2	HHHHHHHHHHHHHHHHHHHHHHHHHCCCCCCC
419	EEEERKKQDQFNNRYEQESYQRYGKGQGHSS	-46.30	35.5	HHHHHHHHHHHHHHHHHHHHHHHHHCCCCCCC
159	EQEYRKWQEQYNNEFEDQYYQQKGQGKGHQP	-46.30	35.5	HHHHHHHHHHHHHHHHHHHHHHHHHCCCCCCC
710	EQEYRKWQEQYSNESEDQRYQRYGQGKGHQS	-46.30	32.3	HHHHHHHHHHHCCHHHHHHHHHHHHCCCCCCC
235	EEEKKKWLEQLSNEKEDKRYQREGKGKGHQP	-46.29	41.9	HHHHHHHHHHHHHHHHHHHHHHHHCCCCCCCC
190	EQQQRHQQEQYSNESEDQRYQKQGRGKGHQP	-46.28	35.5	HHHHHHHHHHCCHHHHHHHHHHHHCCCCCCCC
855	EQEQRHQQEQYSNEYEDEEYQRKGRGKGHQP	-46.27	32.3	HHHHHHHHHHHHHHHHHHHHHHHHHCCCCCCC
388	EEEEKKKRDQYNNEWEQEEYQRKGRGQGHQP	-46.27	38.7	CHHHHHHHHHHHHHHHHHHHHHHHHCCCCCCC
743	EEEERKKQDQYNNESEQQRYQRYGQGQGHQS	-46.27	35.5	HHHHHHHHHHCHHHHHHHHHHHHHHHCCCCCC
292	KQEEEKKQDQYNNEYEQEEYQRKGRGQGHQP	-46.27	29.0	CHHHHHHHHHHHHHHHHHHHHHHHHCCCCCCC

512	EEEEKKNQDQWKNEFEQKYFQQKGQGQGKSH	-46.25	32.3	HHHHHHHHHHHHHHHHHHHHHHHHCCCCCCC
574	EEERKKQLDAYNNRYEQQYYQKKGQGQGHQP	-46.24	38.7	HHHHHHHHHHHHHHHHHHHHHHHHCCCCCCC
308	EEEQRRQQEQRNNEKEDEEYQRRGMGKGHQP	-46.19	38.7	HHHHHHHHHHHHHHHHHHHHHHHHHCCCCCC
437	EEEERKKQQQYNNEAEDQRYQRYGRGKGHQP	-46.18	41.9	HHHHHHHHHHHHHHHHHHHHHHHHCCCCCCC
655	EEEERKYQQQQKQEEEDKRYQRYGRGKGHQP	-46.18	35.5	HHHHHHHHHHHHHHHHHHHHHHHHCCCCCCC
882	EEEEKKKQEQESNEKEDKRYQKQGQGKGHQS	-46.18	38.7	HHHHHHHHHHHHHHHHHHHHHHHCCCCCCCC
590	EEEEKKKREQINNEAEDQRYQREGRGKGHQP	-46.18	45.2	HHHHHHHHHHHHHHHHHHHHHHHHCCCCCCC
932	EQEERKKLEQISNEKEDQEYQKQGWGKGHNP	-46.18	35.5	CHHHHHHHHHHHHHHHHHHHHHHCCCCCCCC
083	EEEERKKQDDFKNRKEDERYQQQGRGKGHQP	-46.16	38.7	HHHHHHHHHHHHHHHHHHHHHHHHCCCCCCC
595	EEEERKMRQKYNNESEDKRYQRYGKGKGHSS	-46.16	41.9	HHHHHHHHHHHHHHHHHHHHHHHCCCCCCCC
920	EEEKRKMLQKKKNEEEDKRYQQQGWGKGHNP	-46.16	41.9	HHHHHHHHHHHHHHHHHHHHHHHCCCCCCCC
525	EEEKRKMLQKMKNEKEDEEYQRRGKGKGHSH	-46.16	41.9	HHHHHHHHHHHHHHHHHHHHHHHHCCCCCCC
719	EEQRRKQLDQYKNESEQQRYQRYGKGQGDSR	-46.16	45.2	HHHHHHHHHHHHHHHHHHHHHHHCCCCCCCC
728	DEERKKMLDKKNQEEEQKRYQQYGRGQGHQP	-46.15	41.9	HHHHHHHHHHHHHHHHHHHHHHHHHCCCCCC
998	EEEERKRQEQEKNEKEDQRYQQQGWGKGHNP	-46.15	35.5	HHHHHHHHHHHHHHHHHHHHHHHCCCCCCCC
761	EQEERHQQEQYSNEYEDQEYQKQGRGKGHQP	-46.14	32.3	CHHHHHHHHHHHHHHHHHHHHHHHCCCCCCC
847	EQERRHQLEQMKNEKEDQEYQRAGRGKGHQP	-46.14	35.5	HHHHHHHHHHHHHHHHHHHHHHHHHCCCCCC
618	EQERRHQLEQQKNEEEDKRYQRYGRGKGHQP	-46.14	35.5	HHHHHHHHHHHHHHHHHHHHHHHHCCCCCCC
457	EQERRHQLEQWKNEWEDKYYQKEGQGKGHQS	-46.14	35.5	HHHHHHHHHHHHHHHHHHHHHHHCCCCCCCC
218	EEERRKQLQKIKNEKEDEEYQRRGQGKGHQS	-46.13	41.9	HHHHHHHHHHHHHHHHHHHHHHHHHCCCCCC
061	EEERRKQLQKINNEAEDKRYQREGRGKGHQP	-46.13	48.4	HHHHHHHHHHHHHHHHHHHHHHHHCCCCCCC
297	EEERRKQLQKYKNEFEDKYYQQKGQGKGHSH	-46.13	41.9	HHHHHHHHHHHHHHHHHHHHHHHHCCCCCCC
944	EEERRKQLEQQNNEKEDQRYQREGRGKGHQP	-46.12	41.9	HHHHHHHHHHHHHHHHHHHHHHHHCCCCCCC
915	EEEQRKQQDQYNNEWEQEEYQRKGRGQGNQN	-46.11	35.5	HHHHHHHHHHHHHHHHHHHHHHHHCCCCCCC
739	EQQQRKYEEQRNNREEDERYQRYGQGKGHQS	-46.09	35.5	HHHHHHHHHHHHHHHHHHHHHHHHHCCCCCC
135	EEEERKLRDQINNEKEQEEYQRRGKGQGHNH	-46.09	35.5	HHHHHHHHHHHHHHHHHHHHHHHHCCCCCCC
378	EEEERKLRDQINNEKEQEEYQRYGRGQGHQP	-46.09	35.5	HHHHHHHHHHHHHHHHHHHHHHHHHCCCCCC
817	EQEERHQQEQRNNEKEDEEYQRRGWGKGHNP	-46.08	35.5	CHHHHHHHHHHHHHHHHHHHHHHCCCCCCCC
315	EEEKRKMLDQINNEKEHEEYQRRGQGQGHQS	-46.07	38.7	HHHHHHHHHHHHHHHHHHHHHHHHHCCCCCC
443	EEEQRKQYQKESNEKEDEEYQRKGRGKGHQP	-46.06	38.7	HHHHHHHHHHHHHHHHHHHHHHHHCCCCCCC
853	EEEQRKQYQKESNEKEDKRYQREGKGKGHQP	-46.06	38.7	HHHHHHHHHHHHHHHHHHHHHHHCCCCCCCC
796	EEEERKYQQQKENEREDQEYQRKGRGKGHQP	-46.05	35.5	CHHHHHHHHHHHHHHHHHHHHHHHCCCCCCC
270	EEEERKKQDQFSNEFEQQYYQQKGQGQGHSH	-46.04	35.5	HHHHHHHHHHHHHHHHHHHHHHHHCCCCCCC
136	DEERKKMLEQIKNEAEDQRYQRYGWGKGHNP	-46.03	41.9	HHHHHHHHHHHHHHHHHHHHHHHCCCCCCCC
098	EEEKRKMLEQKNNEEEDQRYQRYGQGKGHQS	-46.03	41.9	HHHHHHHHHHHCHHHHHHHHHHHHHCCCCCC
022	EQEKRKMLEQWKNEWEDKYYQQQGQGKGHQS	-46.03	35.5	HHHHHHHHHHHHHHHHHHHHHHHHCCCCCCC
323	EQEKRHQLEQMKNDKEDREYQRSGKGKGHSN	-46.02	35.5	HHHHHHHHHHHHHHHHHHHHHHCCCCCCCCC
576	EQERRKQLEQINNDAEDRRYQQYGKGKGHSS	-46.02	38.7	HHHHHHHHHHHHHHHHHHHHHHHCCCCCCCC
775	EEEERKKEQQKRNEEEDQRYQQYGRGKGHQP	-46.01	35.5	HHHHHHHHHHHHHHHHHHHHHHHHHCCCCCC
823	EEERKKQLEQESNEKEDQKYQRQGQGKGHQS	-46.00	41.9	HHHHHHHHHHHHHHHHHHHHHHHHCCCCCCC
490	EEERKKQLEQINNEAEDQRYQREGWGKGHYP	-46.00	48.4	HHHHHHHHHHHHHHHHHHHHHHHHCCCCCCC
946	EEERKKQLEQWKNEWEDKYYQQKGQGKGHSH	-46.00	41.9	HHHHHHHHHHHHHHHHHHHHHHHHCCCCCCC
392	EEERRKQLEQINNEAEDKRYQREGKGKGHSS	-46.00	45.2	HHHHHHHHHHHHHHHHHHHHHHHCCCCCCCC
621	EEERRKQLEQYKNEHEDRRYQEQGKGKGHSS	-46.00	38.7	HHHHHHHHHHHHHHHHHHHHHHHCCCCCCCC
071	EQEERKYQEQKNNEEEDERYQRYGQGKGHQS	-46.00	35.5	CHHHHHHHHHHHHHHHHHHHHHHHHCCCCCC
288	EEEERKKQDKIKNEKEQEEYQRRGQGQGHQS	-45.99	35.5	HHHHHHHHHHHHHHHHHHHHHHHHHCCCCCC
029	EEEEKKRQEQENNDKEDRRYQREGQGKGHSS	-45.99	38.7	HHHHHHHHHHHHCHHHHHHHHHHCCCCCCCC
804	EEERRKQLQKWKNEYEQKSYQREGKGQGHNH	-45.99	35.5	HHHHHHHHHHHHHHHHHHHHHHCCCCCCCCC
465	EQEERKKQEQINNEAEDKRYQRYGQGKGHQS	-45.98	38.7	HHHHHHHHHHHHHHHHHHHHHHHHCCCCCCC
558	EEEEKKRQDKEKNEKEQKRYQREGRGQGHQP	-45.97	38.7	CHHHHHHHHHHHHHHHHHHHHHHHCCCCCCC
508	EEEERKRQEQFSNEFEDQYYQQKGQGKGHSH	-45.97	38.7	HHHHHHHHHHHHHHHHHHHHHHHHCCCCCCC
470	EQEQRKRQEQYNNEYEDEEYQRKGRGKGHQP	-45.97	35.5	HHHHHHHHHHHHHHHHHHHHHHHHCCCCCCC
964	EQEERHQQEQYNNEWEDKRYQREGRGKGHQP	-45.95	35.5	CHHHHHHHHHHHHHHHHHHHHHHHCCCCCCC
233	EEEERKRQDQEKNEKEHQRYQREGRGQGHQP	-45.93	32.3	HHHHHHHHHHHHHHHHHHHHHHHHCCCCCCC
497	EEEKRKQEQQRNNEEEHQRYQREGKGQGHSH	-45.93	32.3	HHHHHHHHHHHHHHHHHHHHHHHHCCCCCCC
133	EEEQKKQREQYNNRYEHEYYQEKGQGQGHQP	-45.93	32.3	HHHHHHHHHHHHHHHHHHHHHHHHCCCCCCC
072	EEEEKKQREQESNEKEDERYQREGQGKGHQS	-45.93	38.7	HHHHHHHHHHHHHHHHHHHHHHHHCCCCCCC
564	EQEERKQREQENNEKEDRRYQRYGWGKGHNP	-45.93	35.5	HHHHHHHHHHHHHHHHHHHHHHCCCCCCCCC
482	EQEERKQREQWKQDWEQQYYQQQGQGQGHQS	-45.93	22.6	HHHHHHHHHHHHHHHHHHHHHHHHCCCCCCC
252	EEEQRKYEDQRNQEEEQQRYQREGKGQGHQS	-45.93	35.5	CHHHHHHHHHHHHHHHHHHHHHHHCCCCCCC
634	EEEQRKYEDQRSQEEEQKRYQREGRGQGHQP	-45.93	32.3	CHHHHHHHHHHHHHHHHHHHHHHHCCCCCCC
899	EEEEKKYQEQYSNEWEDEEYQRKGRGKGHQP	-45.92	38.7	CHHHHHHHHHHHHHHHHHHHHHHHCCCCCCC
250	EEEEKKKREQYNNEAEDKRYQREGKGKGDSR	-45.92	51.6	CHHHHHHHHHHHHHHHHHHHHHHCCCCCCCC
108	EQEERKQREQESNRKEDERYQREGKGKGHNH	-45.90	29.0	HHHHHHHHHHHHHHHHHHHHHHHCCCCCCCC
026	EQEQRKQEEQRSQEKEQEEYQRYGRGQGHQP	-45.90	25.8	HHHHHHHHHHHHHHHHHHHHHHHHHCCCCCC
500	EEEERKIQQQLNNEKEDEEYQRKGRGKGDSR	-45.89	45.2	HHHHHHHHHHHHHHHHHHHHHHHCCCCCCCC
219	EEEERKYQQQKNNEEEDEEYQRKGRGKGHQP	-45.88	38.7	CHHHHHHHHHHHHHHHHHHHHHHHCCCCCCC

896	EEEERKYQQQKNNEEEDERYQQQGRGKGHQP	-45.88	38.7	CHHHHHHHHHHHHHHHHHHHHHHHCCCCCCC
082	EEEERKYQQQKNNEEEDQRYQKQGWGKGHNP	-45.88	38.7	CHHHHHHHHHHHHHHHHHHHHHHCCCCCCCC
117	EEEKRKYLQQYNNESEDERYQQQGKGKGHNH	-45.88	41.9	HHHHHHHHHHHHCCHHHHHHHHHHCCCCCCC
759	EEEQRKYEQQRNNEEEDQRYQKQGRGKGHQP	-45.88	38.7	HHHHHHHHHHHHHHHHHHHHHHHCCCCCCCC
568	EQEQRHQQEQYSNEWEDEEYQRKGRGKGHQP	-45.88	32.3	HHHHHHHHHHHHHHHHHHHHHHHHCCCCCCC
441	EEEERKKQDQYNNESEQQRYQRYGRGQGHQP	-45.87	35.5	HHHHHHHHHHHCHHHHHHHHHHHHHCCCCCC
013	EEEERKQQQKKKQEEEQKRYQREGRGQGHQP	-45.86	32.3	HHHHHHHHHHHHHHHHHHHHHHHHCCCCCCC
692	EEEERKRQQQFSNDYEDKFYQQQGRGKGHQP	-45.85	38.7	HHHHHHHHHHHHHHHHHHHHHHHHCCCCCCC
182	EEEERKKEDQYRNEWEQKRYQREGRGQGHQP	-45.79	32.3	HHHHHHHHHHHHHHHHHHHHHHHHCCCCCCC
306	EEEERKFQQQYKNEHEDQRYQQQGWGKGHNP	-45.78	38.7	CHHHHHHHHHHHHHHHHHHHHHHCCCCCCCC
594	EEERKKQLEQESNEKEDERYQQQGQGKGHQS	-45.77	41.9	HHHHHHHHHHHHHHHHHHHHHHHHCCCCCCC
074	EEEKRKFLDKYKNEYEQEEYQRKGRGQGHQP	-45.77	41.9	HHHHHHHHHHHHHHHHHHHHHHHHCCCCCCC
842	EQEERHQQEQRSNEQEDKRYQRYGRGKGHQP	-45.75	32.3	CHHHHHHHHHHHHHHHHHHHHHHHCCCCCCC
883	EEEQRKEEQQKNNDSEDRRYQREGQGKGHQS	-45.74	35.5	HHHHHHHHHHHHCHHHHHHHHHHHCCCCCCC
312	EEEQRKQEEQRRNDKEQKRYQREGRGQGHQP	-45.73	25.8	HHHHHHHHHHHHHHHHHHHHHHHHCCCCCCC
566	EEERRKQLEQESNDKEDRYFQQYGQGKGKQN	-45.73	32.3	HHHHHHHHHHHHHHHHHHHHHHHHHCCCCCC
097	EEEERKRQEQYSNEYEDEEYQRKGRGKGHQP	-45.72	35.5	HHHHHHHHHHHHHHHHHHHHHHHHCCCCCCC
706	EEERRKQLQQISQEKEDEEYQRYGKGKGHNH	-45.66	38.7	HHHHHHHHHHHHHHHHHHHHHHHHCCCCCCC
181	EEEERKKQEQINNEEEDKRYQREGRGKGHQP	-45.66	38.7	HHHHHHHHHHHCHHHHHHHHHHHHCCCCCCC
060	EEEKRKWLQQLSNEKEDEQYQQRGQGKGHQS	-45.65	38.7	HHHHHHHHHHHHHHHHHHHHHHHHHCCCCCC
454	EEEERKYQQQYNNEWEDKRYQREGRGKGHQP	-45.65	38.7	CHHHHHHHHHHHHHHHHHHHHHHHCCCCCCC
132	EEEKRKYLDQYNNDYEQREYQRKGRGQGHQP	-45.64	35.5	HHHHHHHHHHHHHHHHHHHHHHHHCCCCCCC
686	EEEKRKWLDKLSNEKEQKRYQRQGRGQGHQP	-45.63	38.7	HHHHHHHHHHHHHHHHHHHHHHHHCCCCCCC
043	EQEKRHQEEQRNNEEEDQRYQREGQGKGHQS	-45.62	35.5	HHHHHHHHHHHHHHHHHHHHHHHHCCCCCCC
785	EEEERKYQDQKNNEEEQQRYQKQGRGQGHQP	-45.62	35.5	CHHHHHHHHHHCHHHHHHHHHHHHCCCCCCC
106	EEEERKYQDQYNNEYEQEEYQRKGRGQGHQP	-45.62	35.5	CHHHHHHHHHHHHHHHHHHHHHHHCCCCCCC
197	EEEQKKYEDQRNNEEEQQYYQKYGQGQGHNH	-45.62	38.7	CHHHHHHHHHHCHHHHHHHHHHHHHCCCCCC
971	EEEERRKKEKESNEKEDKRYQREGKGKGHSS	-45.60	38.7	HHHHHHHHHHHHHHHHHHHHHHHCCCCCCCC
448	EEEERKQQEQRSNEKEQEEYQRRGRGQGHQP	-45.56	29.0	HHHHHHHHHHHHHHHHHHHHHHHHHCCCCCC
885	EEEEKKKQEKYKNEYEQEEYQRKGRGQGHQP	-45.55	35.5	HHHHHHHHHHHHHHHHHHHHHHHHCCCCCCC
851	EEEERKKQQQINNDKEDREYQRRGRGKGHQP	-45.55	35.5	HHHHHHHHHHHHHHHHHHHHHHHHCCCCCCC
475	EEEQKKLQDQINNEQEQERYQRQGWGQGHNP	-45.55	38.7	HHHHHHHHHHHHHHHHHHHHHHHCCCCCCCC
295	EEERRKQLEKINNEEEHKRKQQQGKGSGKSR	-45.54	38.7	HHHHHHHHHHHHHHHHHHHHHHHCCCCCCCC
892	EEEERKRQDKYNNEYEHEEYQRKGRGQGNSN	-45.54	38.7	HHHHHHHHHHHHHHHHHHHHHHHCCCCCCCC
466	EEEERKKQQQENNEKEDQRYQREGKGKGHSS	-45.54	38.7	HHHHHHHHHHHHHHHHHHHHHHHCCCCCCCC
870	EEEERKKQQQINNEAEDKRYQREGKGKGHQH	-45.54	41.9	HHHHHHHHHHHHHHHHHHHHHHHHCCCCCCC
755	EEEERKKQQQINNEEEDKRYQREGRGKGHQP	-45.54	38.7	HHHHHHHHHHHCHHHHHHHHHHHHCCCCCCC
524	EEEERKKQQQINNEEEDQKYQREGQGKGHQS	-45.54	38.7	HHHHHHHHHHHHHHHHHHHHHHHHCCCCCCC
025	EEEERKKQQQISNEKEDEEYQRRGKGKGHSS	-45.54	35.5	HHHHHHHHHHHHHHHHHHHHHHHHCCCCCCC
625	EEEERKRQDKENNEREQKRYQREGQGQGHQS	-45.54	38.7	HHHHHHHHHHHHHHHHHHHHHHHHCCCCCCC
210	EQERRKQLEQINNDAEDKRYQKEGKGKGDSR	-45.53	45.2	HHHHHHHHHHHHHHHHHHHHHHHCCCCCCCC
950	EEEEKKFRDQYNNEYEQEEYQRKGRGQGHQP	-45.52	41.9	CHHHHHHHHHHHHHHHHHHHHHHHCCCCCCC
629	EEEKRKFLDQYKNEWEQKRYQREGRGQGHQP	-45.52	38.7	HHHHHHHHHHHHHHHHHHHHHHHHCCCCCCC
871	EEEQRKFEDQYRNEWEQKRYQREGWGQGHYP	-45.52	35.5	CHHHHHHHHHHHHHHHHHHHHHHHCCCCCCC
552	EEEKRKMLDKWKNEWEQKYYQQKGQGQGHQP	-45.52	38.7	HHHHHHHHHHHHHHHHHHHHHHHHCCCCCCC
953	EEERRKQLEQINNDAEDRRYQRYGKGKGHNH	-45.50	41.9	HHHHHHHHHHHHHHHHHHHHHHHCCCCCCCC
645	EEERRKQLDAINNRKEDEEYQRRGKGKGDSR	-45.48	48.4	HHHHHHHHHHHHHHHHHHHHHHHCCCCCCCC
269	EEEKRKQEEQRNNEEEQQRYQRYGRGQGHQP	-45.43	32.3	HHHHHHHHHHHHHHHHHHHHHHHHHCCCCCC
056	EEEQRKQEQQYRQEWEQEEYQRKGRGQGHQP	-45.41	29.0	HHHHHHHHHHHHHHHHHHHHHHHHCCCCCCC
615	EEEERKMRDQYNQEYEQQQYQKEGKGQGHSH	-45.41	35.5	HHHHHHHHHHHHHHHHHHHHHHHHCCCCCCC
048	EEERRKQLEQYKNEWEDEEYQRKGRGKGHNP	-45.41	38.7	HHHHHHHHHHHHHHHHHHHHHHHHCCCCCCC
633	EEEERKQQQQRSNEEEHKQYQQRGWGQGHYP	-45.41	29.0	HHHHHHHHHHHHHHHHHHHHHHHCCCCCCCC
681	EEEYRKWLDQKKNEEEQERYQQQGRGQGHQP	-45.40	35.5	HHHHHHHHHHHHHHHHHHHHHHHHCCCCCCC
921	EQEERKWRDQKNNEEEQQRYQRYGKGQGHSS	-45.40	32.3	CHHHHHHHHHHCHHHHHHHHHHHHCCCCCCC
207	EEEERKMRQQYNNEYEDQEYQRNGRGKGHQY	-45.39	38.7	HHHHHHHHHHHHHHHHHHHHHHHCCCCCCCC
904	EEEKRKMLQQYKNEYEDEEYQRKGRGKGHQP	-45.39	38.7	HHHHHHHHHHHHHHHHHHHHHHHHCCCCCCC
427	EEERRKQLDQEKQEKEQEEYQRRGKGQGHSS	-45.39	35.5	HHHHHHHHHHHHHHHHHHHHHHHCCCCCCCC
124	EEERKKQLQQKNNEEEDKRYQREGKGKGHNH	-45.37	45.2	HHHHHHHHHHHHHHHHHHHHHHHHCCCCCCC
099	EEERRKQEQQLRNEKEDSRYQQQGWGKGHNP	-45.37	35.5	HHHHHHHHHHHHHHHHHHHHHHHCCCCCCCC
111	EEERRKQLQQIKNEKEDEEYQRRGQGKGHQS	-45.37	38.7	HHHHHHHHHHHHHHHHHHHHHHHHHCCCCCC
551	EEERRKQLQQIKNEKEDQEYQKQGRGKGHQP	-45.37	38.7	HHHHHHHHHHHHHHHHHHHHHHHHCCCCCCC
643	EEERRKQLQQINNEEEDKRYQREGMGKGHQP	-45.37	41.9	HHHHHHHHHHHHHHHHHHHHHHHHCCCCCCC
042	EEERRKQLQQWKNEWEDEYYQQYGQGKGHSH	-45.37	38.7	HHHHHHHHHHHHHHHHHHHHHHHHHCCCCCC
995	EEERRKQLQQYKNEWEDKRYQREGQGKGHQS	-45.37	38.7	HHHHHHHHHHHHHHHHHHHHHHHHCCCCCCC
708	EEEERKQRQQESNEKEDEEYQRKGRGKGHQP	-45.29	35.5	HHHHHHHHHHHHHHHHHHHHHHHHCCCCCCC
965	EEEQRKQHQQESNEKEDKRYQREGRGKGHQP	-45.29	35.5	HHHHHHHHHHHHHHHHHHHHHHHHCCCCCCC
703	EEEKRKMLDQYKHEWEQEEYQRKGRGQGHQP	-45.28	38.7	HHHHHHHHHHHHHHHHHHHHHHHHCCCCCCC

194	EEEERKKQDQIKNEEEQKRYQRYGKGQGHSS	-45.28	32.3	HHHHHHHHHHHHHHHHHHHHHHHHCCCCCCC
291	EEEERKKQDQIKNEKEQEEYQRYGWGQGHYP	-45.28	32.3	HHHHHHHHHHHHHHHHHHHHHHHCCCCCCCC
254	EEEERKKQDQINNEKEQEEYQRRGWGQGHNP	-45.28	35.5	HHHHHHHHHHHHHHHHHHHHHHHCCCCCCCC
138	EEEERKKQDQINNEQEQERYQQQGRGQGHQP	-45.28	35.5	HHHHHHHHHHHHHHHHHHHHHHHHCCCCCCC
238	EEERRKQLDQINHREEQERYQREGRGQGHQP	-45.27	38.7	HHHHHHHHHHHHHHHHHHHHHHHHHCCCCCC
212	EEEERKKQQKYKNEWEQKRYQREGRGQGHQP	-45.24	32.3	HHHHHHHHHHHHHHHHHHHHHHHHCCCCCCC
919	EEEQRKYEDDRNNREEDQRYQRYGRGKGHQP	-45.20	38.7	HHHHHHHHHHHHHHHHHHHHHHHHCCCCCCC
616	EEEKRKQEEQRNNEEEQQRYQRQGWGQGHNP	-45.15	32.3	HHHHHHHHHHHHHHHHHHHHHHHCCCCCCCC
586	EEEERKYQDQKNNEEEQKRYQRYGWGQGHNP	-45.15	35.5	CHHHHHHHHHHCHHHHHHHHHHHCCCCCCCC
351	DEERKKMEDQFRNEYEQQSYQREGKGQGHSS	-45.13	35.5	HHHHHHHHHHHHHHHHHHHHHHCCCCCCCCC
671	EEEERKMRDQYNNEYEQEEYQRKGRGQGHQP	-45.13	35.5	HHHHHHHHHHHHHHHHHHHHHHHHCCCCCCC
926	EEEEKKKREKYNNEAEQKRYQRYGQGQGHQS	-45.11	41.9	CHHHHHHHHHHCHHHHHHHHHHHHHCCCCCC
872	EEEEKKIQEQISNEKEHEEYQRRGKGQGNSN	-45.11	32.3	CHHHHHHHHHHHHHHHHHHHHHHCCCCCCCC
956	EEERRKQLDQEKNEREQQYYQREGKGQGHNH	-45.11	35.5	HHHHHHHHHHHHHHHHHHHHHHHHCCCCCCC
948	EEERRKQLDQIKNEQEQQRYQREGKGQGHSS	-45.11	35.5	HHHHHHHHHHHHHHHHHHHHHHHCCCCCCCC
428	EEERRKQLDQINNEAEQKRYQRYGKGQGHNH	-45.11	41.9	HHHHHHHHHHHHHHHHHHHHHHHHCCCCCCC
873	EEERRKQLDQYNNEWEQEEYQRKGRGQGHQP	-45.11	38.7	HHHHHHHHHHHHHHHHHHHHHHHHCCCCCCC
888	EEEKRKFLDDYNNRSEDQRYQRYGQGKGHQS	-45.10	45.2	HHHHHHHHHHHCCCHHHHHHHHHHHCCCCCC
709	EEEQRKYEDKRNNEEEQKRYQRYGKGQGDSR	-45.10	45.2	CHHHHHHHHHHCHHHHHHHHHHHCCCCCCCC
260	EQEKRKLLEKISNEKEQKEYQKQGWGQGHNP	-45.09	32.3	HHHHHHHHHHHHHHHHHHHHHHHCCCCCCCC
134	EEEEKKKRDQENNEKEQQRYQREGQGQGHQS	-45.09	38.7	CHHHHHHHHHHHHHHHHHHHHHHHCCCCCCC
539	EEEERKRQQQESNEREDKRYQREGQGKGHSS	-45.08	35.5	HHHHHHHHHHHHHHHHHHHHHHHCCCCCCCC
849	EQEERKWREQKNNEEEDKRYQREGKGKGDSR	-45.05	41.9	CHHHHHHHHHHHHHHHHHHHHHHCCCCCCCC
018	EQEERHYQEKKNNEEEDKRYQRYGQGKGHQS	-45.05	38.7	CHHHHHHHHHHHHHHHHHHHHHHHHCCCCCC
869	EEEEKKQQEQRSNDQEQRRYQREGRGQGHQP	-45.05	29.0	HHHHHHHHHHHHHHHHHHHHHHHHCCCCCCC
409	EQEQRHQQEQYNNEYEDQEYQRKGRGKGDSR	-45.03	41.9	HHHHHHHHHHHHHHHHHHHHHHHCCCCCCCC
889	EEEEKKQREQYNNEYEQEEYQRKGRGQGHQP	-45.02	35.5	CHHHHHHHHHHHHHHHHHHHHHHHCCCCCCC
120	EEERKKQLEQYKNENEQKQYQRSGRGQGHQP	-45.02	38.7	HHHHHHHHHHHHHHHHHHHHHHHCCCCCCCC
017	EQEERHKQEQKNNEEEDERYQQQGKGKGHSS	-45.02	35.5	HHHHHHHHHHHHHHHHHHHHHHHCCCCCCCC
635	EQEERHKQEQYNNEYEDEYYQQKGQGKGHSH	-45.02	35.5	HHHHHHHHHHHHHHHHHHHHHHHHCCCCCCC
314	EEEERKQQDHRKNRKEQEEYQRRGRGQGHQP	-45.01	29.0	HHHHHHHHHHHHHHHHHHHHHHHHHCCCCCC
695	QDEQRKYEDQRNNEEEQQRYQQYGKGQGHNN	-45.00	29.0	CHHHHHHHHHHCHHHHHHHHHHHHHCCCCCC
723	EEQERKFQDQYNNEFEQKYYQQQGQGQGHSH	-45.00	41.9	CHHHHHHHHHHHHHHHHHHHHHHHCCCCCCC
532	EEEERKQRDHESNRKEDQRYQRQGKGKGHSH	-44.99	35.5	HHHHHHHHHHHHHHHHHHHHHHHCCCCCCCC
955	EEEKKKMLQQIEDDKRDEEYQRRGWGKGHNP	-44.99	35.5	HHHHHHHHHHHHHHHHHHHHHHHCCCCCCCC
253	EEEERKRQEQENNEKEDQRYQREGKGKGDSR	-44.90	45.2	HHHHHHHHHHHHHHHHHHHHHHHCCCCCCCC
307	EEQQRRLYEKISNEKEDEEYQRRGRGKGHQP	-44.90	41.9	HHHHHHHHHHHHHHHHHHHHHHHHCCCCCCC
433	EEERRKQLDQIKNEKEQEEYQRYGKGQGHNH	-44.88	35.5	HHHHHHHHHHHHHHHHHHHHHHHHHCCCCCC
179	EEERRKQLDQKNNEEEQERYQQQGRGQGHQP	-44.88	38.7	HHHHHHHHHHHCHHHHHHHHHHHHCCCCCCC
187	EEEERRYQEKYNNEWEDKRYQREGQGKGHQS	-44.88	41.9	CHHHHHHHHHHHHHHHHHHHHHHHCCCCCCC
711	EEEERKKQDDYRNRWEDEEYQRKGRGKGHQP	-44.86	35.5	HHHHHHHHHHHHHHHHHHHHHHHHCCCCCCC
811	EEQKRKQLQQESNEKEDKRYQRYGRGKGHQP	-44.85	41.9	HHHHHHHHHHHHHHHHHHHHHHHHCCCCCCC
894	EEEQKKYEEQRSNEEEHKRYQRYGWGQGHNP	-44.80	32.3	HHHHHHHHHHHHHHHHHHHHHHHCCCCCCCC
241	EEEERKFRQAFNNRFEDQYFQQQGQGKGKQS	-44.75	38.7	CHHHHHHHHHHHHHHHHHHHHHHHCCCCCCC
748	EQEERHKQEKYNNEAEHKRKQREGKGSGKNH	-44.75	32.3	HHHHHHHHHHHHHHHHHHHHHHHCCCCCCCC
947	EEEKRKLLQKIKQEKEQKEYQRNGSGQGHQY	-44.75	35.5	HHHHHHHHHHHHHHHHHHHHHHHCCCCCCCC
105	EQEERHLQEQQSNEKEDEKYQQEGMGKGHQP	-44.75	32.3	HHHHHHHHHHHHHHHHHHHHHHHHCCCCCCC
797	EQERRKQLEAKNNREEDERYQQQGWGKGHNP	-44.73	35.5	HHHHHHHHHHHHHHHHHHHHHHHCCCCCCCC
912	EEEEKKYQDQYNNEYEQEEYQRKGRGQGHQP	-44.73	38.7	CHHHHHHHHHHHHHHHHHHHHHHHCCCCCCC
226	EEERKKQLDQFNQEFEQQYYQQKGQGQGHNH	-44.71	45.2	HHHHHHHHHHHHHHHHHHHHHHHHCCCCCCC
999	EEEEKKQQEKRNNEKEQRRYQRYGRGQGHQP	-44.71	38.7	HHHHHHHHHHHHHHHHHHHHHHHHHCCCCCC
515	EQEQRHQEEQERNDKEDKRFQREGKGKGKNH	-44.70	25.8	HHHHHHHHHHHHHHHHHHHHHHHHCCCCCCC
684	EEEQRKYEEQREEEERDERYQQQGRGKGHQP	-44.69	32.3	HHHHHHHHHHHHHHHHHHHHHHHCCCCCCCC
046	EQEERKKQEQESNEKEQKRYQREGKGQGHSH	-44.69	25.8	HHHHHHHHHHHHHHHHHHHHHHHCCCCCCCC
857	EEERKKQLDDYNNRWEDQRYQREGRGKGHQP	-44.69	45.2	HHHHHHHHHHHHHHHHHHHHHHHHCCCCCCC
751	EEEERKMRDQYNNEHEQSRYQQQGRGQGHQP	-44.66	35.5	HHHHHHHHHHHHHHHHHHHHHHHHCCCCCCC
922	EEEKKKMLDQINNEAEQKRYQREGKGQGHSH	-44.66	45.2	HHHHHHHHHHHHHHHHHHHHHHHHCCCCCCC
162	EEEQRKEEDQRRNEKEQKRYQREGKGQGHNH	-44.66	32.3	HHHHHHHHHHHHHHHHHHHHHHHHCCCCCCC
663	EEEKRKYEQQRNNEAEDKRYQREGKGKGDSR	-44.64	48.4	HHHHHHHHHHHHHHHHHHHHHHHCCCCCCCC
223	EEERRKQLDQIKNEEEQQRYQRYGMGQGHQP	-44.63	35.5	HHHHHHHHHHHHHHHHHHHHHHHCCCCCCCC
030	EEERRKQLDQIKNEKEQKEYQRSGRGQGHQP	-44.63	38.7	HHHHHHHHHHHHHHHHHHHHHHHCCCCCCCC
980	EEEERKYQEKYNNEAEQKRYQRYGKGQGHNH	-44.63	38.7	CHHHHHHHHHHHHHHHHHHHHHHHCCCCCCC
724	EEEERKKQDQESNRKEQERYQRQGRGQGHQP	-44.63	29.0	HHHHHHHHHHHHHHHHHHHHHHHHCCCCCCC
155	EEEERKKQEKINNEAEHKRYQREGKGQGHSS	-44.63	38.7	HHHHHHHHHHHHHHHHHHHHHHHCCCCCCCC
052	EQERRHQLEQYKNEYEDEEYQRKGRGKGNQN	-44.60	35.5	HHHHHHHHHHHHHHHHHHHHHHHHCCCCCCC
957	EEERRKQLQQWKNDWEQRYYQQKGQGQGHSH	-44.59	29.0	HHHHHHHHHHHHHHHHHHHHHHHHCCCCCCC
582	EQEKRHMLEKYKNEYEDEEYQRKGRGKGHQP	-44.56	38.7	HHHHHHHHHHHHHHHHHHHHHHHHCCCCCCC

585	EQEERKKQEQQNNEREQEEYQRRGRGQGHQP	-44.55	29.0	HHHHHHHHHHHHHHHHHHHHHHHHHCCCCCC
789	EEERKKQLDDYKNRWEDEEYQRKGRGKGHQP	-44.54	41.9	HHHHHHHHHHHHHHHHHHHHHHHHCCCCCCC
316	EQERRHQLEKYKNEYEDEEYQRKGRGKGHQP	-44.53	38.7	HHHHHHHHHHHHHHHHHHHHHHHHCCCCCCC
554	EEEQRRQEEKRRNEKEQKYYQQKGQGQGHNH	-44.53	32.3	HHHHHHHHHHHHHHHHHHHHHHHHCCCCCCC
787	EEEERKKEEQWRNEWEQQMYQRYGKGQGHQP	-44.50	29.0	HHHHHHHHHHHHHHHHHHHHHHHHHCCCCCC
996	EEEKRKLLQQIEERKRDEEYQRRGRGKGHQP	-44.49	32.3	HHHHHHHHHHHHHHHHHHHHHHHHCCCCCCC
158	EEQERRKQEQYNNRWEDERYQREGWGKGHNP	-44.45	38.7	HHHHHHHHHHHHHHHHHHHHHHHHCCCCCCC
640	EQERRHLLEQISNEKEDEEYQRRGQGKGHQS	-44.43	35.5	HHHHHHHHHHHHHHHHHHHHHHHHHCCCCCC
531	EEEERKWREKKNNEEEQKRYQRYGRGQGHQP	-44.40	35.5	CHHHHHHHHHHCHHHHHHHHHHHHHCCCCCC
533	EEEARKSEQKYRNEAEQKRYQREGKGQGHNH	-44.39	38.7	HHHHHHHHHHHHHHHHHHHHHHHHCCCCCCC
806	EEEQRKQEQQYRNDSEQRRYQRYGQGQGHQS	-44.39	25.8	HHHHHHHHHHHHHHHHHHHHHHHHHCCCCCC
925	EEEKRKQEDDRNNREEQQRYQRYGRGQGHQP	-44.38	32.3	HHHHHHHHHHHHHHHHHHHHHHHHHCCCCCC
172	EEEKRKQEQQRNNEKEQEEYQRRGQGQGHQS	-44.38	32.3	HHHHHHHHHHHHHHHHHHHHHHHHHCCCCCC
364	EEEERKKEQQREEERRDEEYQRRGWGKGHNP	-44.37	32.3	HHHHHHHHHHHHHHHHHHHHHHHCCCCCCCC
397	EEEQRKQQQQYSNRFEQEYYQQKGQGQGHSH	-44.35	25.8	HHHHHHHHHHHHHHHHHHHHHHHHCCCCCCC
911	EEEQRKQQQQYSNESEQERYQQQGWGQGHNP	-44.31	29.0	HHHHHHHHHHHCHHHHHHHHHHHCCCCCCCC
326	EQEQRHYEEQRNNEEEDQRYQRYGKGKGHSS	-44.28	35.5	HHHHHHHHHHHHHHHHHHHHHHHHCCCCCCC
265	EEEEKKRQEKESNEKEQEEYQRKGRGQGHQP	-44.25	35.5	HHHHHHHHHHHHHHHHHHHHHHHHCCCCCCC
493	EEQRRKQLDDIKNRKEDEEYQRRGWGKGHNP	-44.25	41.9	HHHHHHHHHHHHHHHHHHHHHHHCCCCCCCC
452	EQEERHQQEQYNNESEHERYQQQGQGQGHSS	-44.23	29.0	CHHHHHHHHHHCHHHHHHHHHHHCCCCCCCC
057	EEERRRQLEKIKNEKEDEEYQRYGKGKGHSN	-44.21	41.9	HHHHHHHHHHHHHHHHHHHHHHHCCCCCCCC
578	EEERRKQLDQESNEKEQKRYQREGQGQGHSS	-44.21	35.5	HHHHHHHHHHHHHHHHHHHHHHHCCCCCCCC
504	EEEQKKMYEKINNEKEQEEYQRRGRGQGHQP	-44.20	38.7	HHHHHHHHHHHHHHHHHHHHHHHHHCCCCCC
654	EEERRKQLQKISNEEEHKRYQREGKGQGHSH	-44.18	35.5	HHHHHHHHHHHCHHHHHHHHHHHHCCCCCCC
547	EEERRKQLEQISNEEEHKRKQREGKGSGKSR	-44.18	32.3	HHHHHHHHHHHHHHHHHHHHHHHCCCCCCCC
624	EEEKKKMLEKINNEAEQKRYQREGRGQGHQP	-44.13	45.2	HHHHHHHHHHHHHHHHHHHHHHHHCCCCCCC
310	EEEERRYQEQYNNDYEDREYQRKGRGKGHQP	-44.12	35.5	CHHHHHHHHHHHCHHHHHHHHHHHCCCCCCC
852	EEERRKQLQQESNEKEHEQKQQRGQGSGKQS	-44.10	29.0	HHHHHHHHHHHHHHHHHHHHHHHCCCCCCCC
171	EEEEKKKREHYNNRWEDERYQQQGQGKGHQS	-44.07	38.7	HHHHHHHHHHHHHHHHHHHHHHHHCCCCCCC
895	EEEERKFQQQFNNEFEHEYYQQKGQGQGHNH	-44.06	38.7	CHHHHHHHHHHHHHHHHHHHHHHHCCCCCCC
730	EQERRHQLEQIKQEKEDEEYQRRGRGKGHQP	-44.06	35.5	HHHHHHHHHHHHHHHHHHHHHHHHCCCCCCC
906	EQERRHQLEQWKQEWEDEYYQRKGQGKGHNH	-44.06	35.5	HHHHHHHHHHHHHHHHHHHHHHHHCCCCCCC
858	EQEERHWQEQMKNEKEDEEYQRRGWGKGHNP	-44.06	32.3	CHHHHHHHHHHHHHHHHHHHHHHCCCCCCCC
151	EEEQRKYEEKRNNEEEQKRYQREGWGQGHNP	-44.03	35.5	CHHHHHHHHHHHHHHHHHHHHHHHCCCCCCC
987	EQEKRKLLEQIENEREQQEYQREGRGQGHQP	-44.01	29.0	HHHHHHHHHHHHHHHHHHHHHHHHCCCCCCC
544	EEEKRKLLQQIKNEEEQKRFQRYGRGQGKQN	-44.01	29.0	HHHHHHHHHHHHHHHHHHHHHHHHHCCCCCC
721	EEEERKKEQKYRNEAEQKRYQREGKGQGHSS	-43.97	35.5	HHHHHHHHHHHHHHHHHHHHHHHCCCCCCCC
109	EEERRKQLQKESQEKEQKRYQREGRGQGHQP	-43.95	35.5	HHHHHHHHHHHHHHHHHHHHHHHHCCCCCCC
537	EQEQRKEEEKKRNEKEQKRYQREGRGQGHQP	-43.93	29.0	HHHHHHHHHHHHHHHHHHHHHHHHCCCCCCC
236	EEEERKKQEQYNNEYEQEEYQRKGRGQGHQP	-43.90	32.3	HHHHHHHHHHHHHHHHHHHHHHHHCCCCCCC
481	EEEQRKQQQQQSNEEEQQRYQRYGKGQGDSR	-43.88	35.5	HHHHHHHHHHHHHHHHHHHHHHHCCCCCCCC
714	EEEERKKQQQYNNEYEQEEYQRQGRGQGHQP	-43.86	32.3	HHHHHHHHHHHHHHHHHHHHHHHHCCCCCCC
958	EEQERKKQQQINNEKEDEEYQRRGKGKGDSR	-43.86	48.4	HHHHHHHHHHHHHHHHHHHHHHHCCCCCCCC
836	EQEERKYQEQFNNRFEQEYYQQKGQGQGHQP	-43.84	29.0	CHHHHHHHHHHHHHHHHHHHHHHHCCCCCCC
485	EEERRKQLQAEKNRKEDQRYQREGKGKGHSH	-43.83	35.5	HHHHHHHHHHHHHHHHHHHHHHHCCCCCCCC
756	EEEERKKQQQINNEKEHQEYQKQGQGQGHQS	-43.82	32.3	HHHHHHHHHHHHHHHHHHHHHHHCCCCCCCC
275	EQEKRHMLEQKNNEEEDKRYQREGRGKGHQP	-43.79	38.7	HHHHHHHHHHHHHHHHHHHHHHHHCCCCCCC
569	EQERRHQLEQESNDKEDRRYQREGRGKGHQP	-43.78	32.3	HHHHHHHHHHHHCCHHHHHHHHHHCCCCCCC
916	EEEQRKFEEQYRNDAEQRRYQREGRGQGHQP	-43.77	32.3	HHHHHHHHHHHHHHHHHHHHHHHHCCCCCCC
324	EQEKRHMLEQINNRAEDERYQREGKGKGHNH	-43.77	38.7	HHHHHHHHHHHHHHHHHHHHHHHCCCCCCCC
280	EQERRHQLEQQSNEEEDERYQQQGWGKGHNP	-43.77	35.5	HHHHHHHHHHHHHHHHHHHHHHHCCCCCCCC
694	EQERRHQLEQYKNEWEDEEYQRKGRGKGHQP	-43.77	35.5	HHHHHHHHHHHHHHHHHHHHHHHHCCCCCCC
248	EQERRHQLEQYSNEYEDKQYQKEGKGKGHNH	-43.77	35.5	HHHHHHHHHHHHHHHHHHHHHHHCCCCCCCC
546	EQEERHKQEQFSNRFEDEYYQKYGQGKGHSH	-43.74	32.3	HHHHHHHHHHHHHHHHHHHHHHHHHCCCCCC
344	EEEQKKQEEQYRNEYEQEEYQRKGRGQGDSR	-43.71	38.7	HHHHHHHHHHHHHHHHHHHHHHHCCCCCCCC
834	EEEQRKLYQQLNNEKEQKRYQREGRGQGHQP	-43.69	32.3	HHHHHHHHHHHHHHHHHHHHHHHHCCCCCCC
088	EEERRKLLQQISNEKEQEEYQRRGWGQGHYP	-43.69	32.3	HHHHHHHHHHHHHHHHHHHHHHHCCCCCCCC
354	EQEERHQREQQSNEREQKRYQREGRGQGHQP	-43.67	25.8	CHHHHHHHHHHHHHHHHHHHHHHHCCCCCCC
410	EQERRHQLEQISNEEEDRRYQREGKGKGHSH	-43.64	35.5	HHHHHHHHHHHHHHHHHHHHHHHCCCCCCCC
523	EEERKKQLEQIKQEAEQQRYQREGKGQGHSS	-43.64	38.7	HHHHHHHHHHHHHHHHHHHHHHHCCCCCCCC
700	EEERKKQLEKIKNEKEQKEYQRSGRGQGHQP	-43.64	41.9	HHHHHHHHHHHHHHHHHHHHHHHCCCCCCCC
330	EQERRHQLEQISNEKEDERYQQQGRGKGHQP	-43.62	35.5	HHHHHHHHHHHHHHHHHHHHHHHHCCCCCCC
023	EQQQRHQEEQYRNEEEQQRYQRQGRGQGHQP	-43.61	29.0	HHHHHHHHHHHHHHHHHHHHHHHHCCCCCCC
961	EQEQRHQEEQWRNEYEQEEYQRKGRGQGHQP	-43.61	25.8	HHHHHHHHHHHHHHHHHHHHHHHHCCCCCCC
298	EEEERKRQDQENNEREQKRYQREGKGQGDSR	-43.58	41.9	HHHHHHHHHHHHHHHHHHHHHHHCCCCCCCC
768	EEEKRRQLEQESNDKEDRRYQREGWGKGHNP	-43.53	35.5	HHHHHHHHHHHHHHHHHHHHHHHHCCCCCCC
704	EQQERHKQEQINNEKEDEEYQRRGRGKGHQP	-43.50	38.7	CHHHHHHHHHHHHHHHHHHHHHHHCCCCCCC

642	EEEERKMRQKFNNEYEQKSYQREGRGQGHQP	-43.50	38.7	HHHHHHHHHHHHHHHHHHHHHHHHCCCCCCC
024	EEEKRKMLQKKNNEEEQKRYQRYGRGQGHQP	-43.50	38.7	HHHHHHHHHHHCCHHHHHHHHHHHHCCCCCC
628	EQEERHRLEQMKNEKEDEEYQRRGRGKGHQP	-43.48	35.5	HHHHHHHHHHHHHHHHHHHHHHHHHCCCCCCC
449	EEEQRKQQEQYSEEWRQKRYQREGRGQGHQP	-43.40	25.8	HHHHHHHHHHHHHHHHHHHHHHHHHCCCCCCC
974	EEEERKMREQYNNDYEQRQYQQQGRGQGHQP	-43.38	29.0	HHHHHHHHHHHHHHHHHHHHHHHHHCCCCCCC
059	EQEARHQQEQYSNDAEQRRYQREGRGQGHQP	-43.36	29.0	HHHHHHHHHHHCHHHHHHHHHHHHHCCCCCCC
317	EEERKKQLEQYKNEWEQEEYQRKGRGQGHQP	-43.34	35.5	HHHHHHHHHHHHHHHHHHHHHHHHHCCCCCCC
819	EEEERRRQEQYNNEYEDEEYQRKGRGKGHQP	-43.31	38.7	HHHHHHHHHHHHHHHHHHHHHHHHHCCCCCCC
053	EEEERKYQEDYNNREEDQKYQREGRGKGHQP	-43.29	35.5	HHHHHHHHHHHHHHHHHHHHHHHHHCCCCCCC
403	EQEERHQREQYNNEYEQQQYQKEGRGQGHQP	-43.29	29.0	CHHHHHHHHHHHHHHHHHHHHHHHHCCCCCCC
491	EQEKRHMLEQISNEKEDEEYQRYGRGKGHQP	-43.27	35.5	HHHHHHHHHHHHHHHHHHHHHHHHHHCCCCCC
803	EEEQRKYEQQRNNDEEQRRYQRYGWGQGHNP	-43.24	29.0	HHHHHHHHHHHHHHHHHHHHHHHHCCCCCCCC
773	EQEARHNQEDYNQRSEDERYQQQGRGKGHQP	-43.24	35.5	HHHHHHHHHHHHHHHHHHHHHHHHHCCCCCCC
411	EEEERKYQQQFKNEFEQKYYQQKGQGQGHNH	-43.22	32.3	CHHHHHHHHHHHHHHHHHHHHHHHHCCCCCCC
772	EEEEKKFQQKYNHEYEQEEYQRKGRGQGHQP	-43.18	45.2	CHHHHHHHHHHHHHHHHHHHHHHHHCCCCCCC
033	EQEQRHYEEQRSNDEEHRRKQREGRGSGKQN	-43.10	19.4	HHHHHHHHHHHHHHHHHHHHHHHHCCCCCCCC
358	EQEERHKQEQYSNEYEDEEYQRKGRGKGHSN	-43.09	32.3	HHHHHHHHHHHHHHHHHHHHHHHHCCCCCCCC
518	EEEEKKREEQYRNEWEQKRYQREGKGQGHNH	-43.06	32.3	HHHHHHHHHHHHHHHHHHHHHHHHHCCCCCCC
973	EEEEKKRQEQQSNEEEQQRYQRYGWGQGHYP	-43.06	32.3	HHHHHHHHHHHHHHHHHHHHHHHHCCCCCCCC
831	EEERRKQLQQESQEKEQQRYQREGKGQGHNH	-43.00	32.3	HHHHHHHHHHHHHHHHHHHHHHHHHCCCCCCC
816	EEEERKKEQQYRNEYEQKQYQQQGQGQGHQS	-43.00	29.0	HHHHHHHHHHHHHHHHHHHHHHHHHCCCCCCC
131	EEEEKKYQQQFNHEYEQQSYQREGRGQGHQP	-42.98	41.9	CHHHHHHHHHHHHHHHHHHHHHHHCCCCCCCC
675	EEEKKKMLEQYKNEWEQEEYQRKGRGQGHQP	-42.92	35.5	HHHHHHHHHHHHHHHHHHHHHHHHHCCCCCCC
495	EEEERKKMEQMKNEAEQQSYQQYGRGQGHQP	-42.92	32.3	CHHHHHHHHHHHHHHHHHHHHHHHHHCCCCCC
267	EEEERKMREQFNNEYEQQRYQQEGQGQGHQS	-42.89	35.5	HHHHHHHHHHHHHHHHHHHHHHHHHCCCCCCC
335	EQERRKQLEQYKNEWEQEYYQRKGQGQGHNH	-42.87	29.0	HHHHHHHHHHHHHHHHHHHHHHHHHCCCCCCC
679	EEEERKKRQAYNNRSEQQRYQRYGRGQGHQP	-42.86	29.0	HHHHHHHHHHHHHHHHHHHHHHHHHHCCCCCC
422	EEEEKKQQEDRSNRKEQEEYQRRGRGQGHQP	-42.79	29.0	HHHHHHHHHHHHHHHHHHHHHHHHHHCCCCCC
137	EEEEKKKREQINNEEEQQRYQRYGRGQGHQP	-42.73	35.5	HHHHHHHHHHHCHHHHHHHHHHHHHHCCCCCC
277	EEERRKQEQQRNNEEEQQRYQRYGQGQGHQS	-42.71	32.3	HHHHHHHHHHHHHHHHHHHHHHHHHHCCCCCC
003	EEERRKQLQQESNEKEQKRYQREGKGQGHQP	-42.71	32.3	HHHHHHHHHHHHHHHHHHHHHHHHHCCCCCCC
080	EEEKKRQEEQRSNDEEQRRYQREGQGQGHQS	-42.67	29.0	HHHHHHHHHHHCHHHHHHHHHHHHCCCCCCCC
251	EQERRHQLEQYKNESEDERYQQQGKGKGDSR	-42.52	41.9	HHHHHHHHHHHHHHHHHHHHHHHHCCCCCCCC
788	EEERRKQLQHYKNRWEQEEYQRKGRGQGHQP	-42.52	29.0	HHHHHHHHHHHHHHHHHHHHHHHHHCCCCCCC
035	EQERRHQLEQIKNDKEHREKQRNGSGSGKQY	-42.51	22.6	HHHHHHHHHHHHHHHHHHHHHHHCCCCCCCCC
627	EQEQRKQQEAYSERYRQEEYQRKGRGQGHQP	-42.43	19.4	HHHHHHHHHHHHHHHHHHHHHHHHHCCCCCCC
272	EEEERKRQEQESNRKEQERYQREGRGQGHQP	-42.39	25.8	HHHHHHHHHHHHHHHHHHHHHHHHHCCCCCCC
160	EQEERHNQEQYNNESEQQRYQRYGRGQGNQN	-42.38	29.0	CHHHHHHHHHHCCHHHHHHHHHHHHCCCCCCC
538	EQEERHKKEQYDNEWEQEKYQKEGRGQGHQP	-42.36	25.8	CHHHHHHHHHHHHHHHHHHHHHHHHCCCCCCC
402	EEEQRKYEQQRNNEEEQEEYQRKGRGQGNQN	-42.29	32.3	HHHHHHHHHHHHHHHHHHHHHHHHHCCCCCCC
079	EQEERHKQEQIKNEKEDEEYQRRGKGKGDSR	-42.18	38.7	HHHHHHHHHHHHHHHHHHHHHHHHCCCCCCCC
228	EEEERKMRDDYNNRYEQEEYQRKGRGQGHQP	-42.05	32.3	HHHHHHHHHHHHHHHHHHHHHHHHHCCCCCCC
864	EQSERHKQEKWKNEWEQKYYQQQGRGQGHQP	-41.89	29.0	CHHHHHHHHHHHHHHHHHHHHHHHHCCCCCCC
116	EEQERKARQQFNNEFEQQYYQKYGQGQGHNH	-41.85	38.7	HHHHHHHHHHHHHHHHHHHHHHHHHHCCCCCC
299	EEEKRKYLQDYNNRWEDQRYQREGRGKGHQP	-41.81	38.7	HHHHHHHHHHHHHHHHHHHHHHHHHCCCCCCC
446	EQEQRHYEEQRNNEEEQQRYQREGWGQGHNP	-41.62	29.0	HHHHHHHHHHHHHHHHHHHHHHHHHCCCCCCC
593	EEERKKQLEDYNNRSEQERYQQQGQGQGHQS	-41.58	35.5	HHHHHHHHHHHHHHHHHHHHHHHHHHCCCCCC
271	EEEERKRQQQESNEKEQKRYQREGRGQGNQN	-41.48	29.0	HHHHHHHHHHHHHHHHHHHHHHHHHCCCCCCC
910	EQERRHQLEKINNEAEQKRYQRYGRGQGHQP	-41.24	38.7	HHHHHHHHHHHHHHHHHHHHHHHHHHCCCCCC
178	EQQQRHYEEQRSNDEEQRYYQQQGQGQGHNH	-41.20	25.8	HHHHHHHHHHHHHHHHHHHHHHHHHCCCCCCC
673	EQEERHMREQYNNEYEQEEYQQQGRGQGHQP	-41.13	29.0	HHHHHHHHHHHHHHHHHHHHHHHHHCCCCCCC
349	EQERRHQLEQINNDQEQREYQRRGRGQGHQP	-41.12	29.0	HHHHHHHHHHHHHHHHHHHHHHHHHHCCCCCC
707	EQEERKYQEDRNNREEQQRYQREGRGQGHQP	-40.63	25.8	CHHHHHHHHHHHHHHHHHHHHHHHHCCCCCCC
821	EEEERRRQEQYNNRWEQERYQREGKGQGHSH	-40.62	29.0	HHHHHHHHHHHHHHHHHHHHHHHHCCCCCCCC
439	EEEERKRQQDFSNRYEQQYYQKQGQGQGHSH	-39.19	29.0	HHHHHHHHHHHHHHHHHHHHHHHHHCCCCCCC
843	EEEERKYQQHFEERFRQQYYQQKGQGQGHNH	-38.10	25.8	HHHHHHHHHHHHHHHHHHHHHHHHHCCCCCCC
680	EQEERHYQEDYNNRYEQEEYQRKGRGQGHSN	-37.92	25.8	CHHHHHHHHHHHHHHHHHHHHHHHCCCCCCCC

Table S3. Summary of 966 peptide sequences designed using EvoEF2 and the evolutionary profile (weight = 0.50).

#label	#peptide	#binding(EEU)	#SeqID(%)	#Secondary structure
WT	EEQAKTFLDKFNHEAEDLFYQSSGLGKGDFR	-46.46	100.	HHHHHHHHHHHHHHHHHHHHHHHCCCCCCCC
866	EEERRKQLDKYNNEHEDKRYQREGMGKGHQP	-51.00	48.4	HHHHHHHHHHHHHHHHHHHHHHHHCCCCCCC
986	EEEARKQLDKYKNEEEDKRYQREGQGKGHQS	-50.81	48.4	HHHHHHHHHHHHHHHHHHHHHHHHCCCCCCC
473	EEEKKKILDKINQEAEDKRYQREGWGKGHNP	-50.78	54.8	HHHHHHHHHHHHHHHHHHHHHHHHCCCCCCC
266	EEEKRKILDKINNEAEDKRYQREGMGKGHQP	-50.48	51.6	HHHHHHHHHHHHHHHHHHHHHHHHHCCCCCC
685	EEEQRKQQDKYNNEYEDKYYQQKGFGKGHQS	-50.33	45.2	HHHHHHHHHHHHHHHHHHHHHHHCCCCCCCC
477	EEERKKQLDQYNNEYEDREYQRNGRGKGHQY	-50.22	48.4	HHHHHHHHHHHHHHHHHHHHHHCCCCCCCCC
130	EEERRKQLDQFNNRKEDERYQQQGMGKGHQP	-50.09	45.2	HHHHHHHHHHHHHHHHHHHHHHHHCCCCCCC
514	EEERKKQLDQYNNEHEDQRYQREGQGKGHSS	-50.05	48.4	HHHHHHHHHHHHHHHHHHHHHHHCCCCCCCC
440	EEERRKQLDQFNNEYEDSFYQQKGFGKGHQS	-50.05	51.6	HHHHHHHHHHHHHHHHHHHHHHHCCCCCCCC
824	EEERRKQLDQYNNEEEDKRYQREGMGKGHQP	-50.05	45.2	HHHHHHHHHHHCHHHHHHHHHHHHCCCCCCC
565	EEELKKILDQINQEEEDRRYQRYGFGKGHQS	-50.00	48.4	HHHHHHHHHHHHHHHHHHHHHHHCCCCCCCC
448	EEEEKKKRDKYNNEYEDEEYQRKGRGKGHQP	-49.88	48.4	CHHHHHHHHHHHHHHHHHHHHHHHCCCCCCC
591	EEEKRKILDQLNQEKEDQRYQREGMGKGHQP	-49.83	45.2	HHHHHHHHHHHHHHHHHHHHHHHHHCCCCCC
776	EEELRKILDQINQEEEDQRYQQYGFGKGHQN	-49.83	45.2	HHHHHHHHHHHHHHHHHHHHHHHCCCCCCCC
884	EEEKRKILDQINNEQEDQSFQRHGFGKGKSR	-49.81	45.2	HHHHHHHHHHHHHHHHHHHHHHHCCCCCCCC
426	EEEKRKLLDKINNEEEDKRFQREGFGKGKQN	-49.77	45.2	HHHHHHHHHHHHHHHHHHHHHHHHCCCCCCC
190	EEERKKQLEKYNQEHEDKRYQREGMGKGHQP	-49.58	48.4	HHHHHHHHHHHHHHHHHHHHHHHHCCCCCCC
031	EEEKRKILDQINNEEEDKRYQRYGFGKGHQS	-49.53	45.2	HHHHHHHHHHHHHHHHHHHHHHHCCCCCCCC
080	EEEKRKILDQYNNEYEDQEYQRNGRGKGHSY	-49.53	45.2	HHHHHHHHHHHHHHHHHHHHHHCCCCCCCCC
566	EEERRKQLDQFNNRFEDEYYQQYGFGKGHQS	-49.48	45.2	HHHHHHHHHHHHHHHHHHHHHHHCCCCCCCC
205	EEEKKKLLDKINNEEEDKRYQQYGFGKGHQS	-49.46	51.6	HHHHHHHHHHHHHHHHHHHHHHHCCCCCCCC
283	EEEKRKLLDKINNEAEDKRYQKEGMGKGHQP	-49.46	51.6	HHHHHHHHHHHHHHHHHHHHHHHHCCCCCCC
207	EEEKRKLLDKINNEEEDKRYQREGFGKGHQS	-49.46	48.4	HHHHHHHHHHHHHHHHHHHHHHHHCCCCCCC
969	EEEKRKLLDKLNNEKEDKRYQREGQGKGHQS	-49.46	48.4	HHHHHHHHHHHHHHHHHHHHHHHHCCCCCCC
179	EEERRKQLDQYNNEYEDEYYQQQGQGKGHSY	-49.44	45.2	HHHHHHHHHHHHHHHHHHHHHHHHCCCCCCC
724	EEEQRKQQDQYNNEYEDQKYQEQGFGKGHQS	-49.38	41.9	HHHHHHHHHHHHHHHHHHHHHHHCCCCCCCC
297	EEERRKQLEKFNNEFEDKYYQQQGFGKGHSS	-49.29	48.4	HHHHHHHHHHHHHHHHHHHHHHHCCCCCCCC
066	EEERRKQLEKFNNEYEDKYYQQQGFGKGHQS	-49.29	48.4	HHHHHHHHHHHHHHHHHHHHHHHCCCCCCCC
933	EEERKKQLDQYNNEEEDMRYQREGMGKGHQP	-49.21	48.4	HHHHHHHHHHHCCHHHHHHHHHHHHCCCCCC
732	EQEKRKLLDQINQEKEDREYQRYGFGKGHQS	-49.16	41.9	HHHHHHHHHHHHHHHHHHHHHHHCCCCCCCC
956	EEERKKQLEKMKNEKEDKEYQRNGSGKGHSY	-49.10	45.2	HHHHHHHHHHHHHHHHHHHHHHCCCCCCCCC
745	EEEAKKQQEKYSNEYEDKEYQRNGRGKGHSY	-49.10	45.2	HHHHHHHHHHHHHHHHHHHHHHCCCCCCCCC
148	EEEKRKQLEKYNNEEEDKRYQREGMGKGHQP	-49.10	45.2	HHHHHHHHHHHCHHHHHHHHHHHHCCCCCCC
303	EEEKRKLLDQINQEAEDERYQQQGMGKGHQP	-49.05	48.4	HHHHHHHHHHHHHHHHHHHHHHHHCCCCCCC
689	EEEKKKLLDQIENEREDQEYQRYGFGKGHQS	-48.91	45.2	HHHHHHHHHHHHHHHHHHHHHHHCCCCCCCC
238	EEEKKKFLDKYNNEEEDMRYQREGMGKGHQP	-48.90	54.8	HHHHHHHHHHHCCHHHHHHHHHHHHCCCCCC
688	EEEKRKFLDKYNNEYEDKYYQQKGFGKGHSS	-48.90	51.6	HHHHHHHHHHHHHHHHHHHHHHHCCCCCCCC
836	EEEKRKLLDQINNEEEDRRYQREGFGKGHQS	-48.86	45.2	HHHHHHHHHHHHHHHHHHHHHHHHCCCCCCC
653	EEEEKKIQEKRNNEEEDKQYQRSGMGKGHQP	-48.77	48.4	CHHHHHHHHHHHHHHHHHHHHHHHCCCCCCC
293	EEEKKKILEKINNEAEDKRYQRQGFGKGHSS	-48.77	51.6	HHHHHHHHHHHHHHHHHHHHHHHCCCCCCCC
001	EEEKRKILEKINNEEEDKRYQREGMGKGHQP	-48.77	45.2	HHHHHHHHHHHHHHHHHHHHHHHHCCCCCCC
861	EEEKRKILEKINNEKEDKEYQRAGRGKGHQP	-48.77	45.2	HHHHHHHHHHHHHHHHHHHHHHHHCCCCCCC
379	EEEKRKILEKINNEKEDKEYQRSGMGKGHQP	-48.77	48.4	HHHHHHHHHHHHHHHHHHHHHHHHCCCCCCC
664	EEEKRKILEKLNNEKEDKRYQRYGFGKGHQS	-48.77	45.2	HHHHHHHHHHHHHHHHHHHHHHHCCCCCCCC
580	EEEKKKLLDQINNEAEDKRYQRYGQGKGHQS	-48.75	51.6	HHHHHHHHHHHHHHHHHHHHHHHHCCCCCCC
326	EEEKKKLLDQINNEKEDEEYQRYGFGKGHSS	-48.75	48.4	HHHHHHHHHHHHHHHHHHHHHHHCCCCCCCC
469	EEEKKKLLDQINNEKEDQEYQKQGMGKGHQP	-48.75	48.4	HHHHHHHHHHHHHHHHHHHHHHHHCCCCCCC
413	EEEKKKLLDQLNNEKEDKRYQREGRGKGHQP	-48.75	48.4	HHHHHHHHHHHHHHHHHHHHHHHHCCCCCCC
530	EEEKRKLLDQINNEAEDKRYQREGKGKGHSA	-48.75	48.4	HHHHHHHHHHHHHHHHHHHHHHHCCCCCCCC
160	EEEKRKLLDQINNEAEDQRYQREGMGKGHQP	-48.75	48.4	HHHHHHHHHHHHHHHHHHHHHHHHHCCCCCC
803	EEEKRKLLDQINNEEEDERYQQQGMGKGHQP	-48.75	45.2	HHHHHHHHHHHHHHHHHHHHHHHHCCCCCCC
142	EEEKRKLLDQINNEEEDKRYQQQGFGKGHQS	-48.75	45.2	HHHHHHHHHHHHHHHHHHHHHHHHCCCCCCC
046	EEEKRKLLDQINNEEEDQRYQQQGFGKGHQS	-48.75	45.2	HHHHHHHHHHHHHHHHHHHHHHHCCCCCCCC
394	EEEKRKLLDQINNEEEDQRYQQYGFGKGHQS	-48.75	45.2	HHHHHHHHHHHHHHHHHHHHHHHCCCCCCCC
733	EEEKRKLLDQINNEEEDQRYQRYGMGKGHQP	-48.75	45.2	HHHHHHHHHHHHHHHHHHHHHHHHCCCCCCC
858	EEEKRKLLDQLNNEKEDQRYQRYGFGKGHSS	-48.75	45.2	HHHHHHHHHHHHHHHHHHHHHHHCCCCCCCC
282	EEERRKLLDQINNEEEDSRYQQQGMGKGHQP	-48.75	45.2	HHHHHHHHHHHHHHHHHHHHHHHHCCCCCCC
792	EEERRKQLEKFNNEFEDKYYQQKGFGKGHQS	-48.69	48.4	HHHHHHHHHHHHHHHHHHHHHHHCCCCCCCC
746	EEERKKQLEQFNQREEDERYQREGFGKGHSS	-48.65	45.2	HHHHHHHHHHHHHHHHHHHHHHHHCCCCCCC
094	EQEKRKILEQYNNDYEDEEYQRKGRGKGHQP	-48.60	35.5	HHHHHHHHHHHHHHHHHHHHHHHHCCCCCCC
362	EQEQRKQQEQFSNEYEDRFYQQKGFGKGHSS	-48.58	38.7	HHHHHHHHHHHHHHHHHHHHHHHCCCCCCCC
601	EEEKRKFLDKKNNEEEQKRYQREGKGRGHSH	-48.54	45.2	HHHHHHHHHHCCCHHHHHHHHHHCCCCCCCC
049	EEEKRKLLDQINNEAEDKRYQREGKGKGHSH	-48.52	48.4	HHHHHHHHHHHHHHHHHHHHHHHCCCCCCCC

352	DEERKKMLDKINNEAEDKRYQREGKGKGHSH	-48.51	51.6	HHHHHHHHHHHHHHHHHHHHHHHCCCCCCCC
414	EEEKKKMLDKKNNEEEDKRYQREGMGKGHQP	-48.51	51.6	HHHHHHHHHHCCHHHHHHHHHHHHCCCCCCC
044	EEEKRKMLDKINNEEEDKRYQREGMGKGHQP	-48.51	48.4	HHHHHHHHHHHHHHHHHHHHHHHHCCCCCCC
344	EEEKRKMLDKINNEQEDKRYQQQGFGKGHQS	-48.51	48.4	HHHHHHHHHHHHHHHHHHHHHHHHCCCCCCC
248	EEEKRKFLDQFNQEYEDEEYQRKGRGKGHQS	-48.49	51.6	HHHHHHHHHHHHHHHHHHHHHHHHCCCCCCC
177	EEEKRKFLDQYNQESEDERYQQQGFGKGHQS	-48.49	48.4	HHHHHHHHHHHHHHHHHHHHHHHCCCCCCCC
461	EEERKKQLDKENNEKEDKRYQRYGFGKGHQS	-48.48	51.6	HHHHHHHHHHHHHHHHHHHHHHHCCCCCCCC
102	EEERRKQLDKINNEQEDEEYQRKGRGKGHQS	-48.48	48.4	HHHHHHHHHHHHHHHHHHHHHHHHCCCCCCC
705	EEERRKQLDKYNNEYEDKYYQQKGFGKGHQS	-48.48	48.4	HHHHHHHHHHHHHHHHHHHHHHHCCCCCCCC
254	EQERRKQLDKFNNEYEDKFYQQKGFGKGHQS	-48.48	51.6	HHHHHHHHHHHHHHHHHHHHHHHCCCCCCCC
292	EEEWRKILEKLNNEKEDKRYQREGMGKGHQP	-48.44	45.2	HHHHHHHHHHHHHHHHHHHHHHHHCCCCCCC
670	EEEQKKQREQFNNEYEDQRYQQEGFGKGHSS	-48.42	45.2	HHHHHHHHHHHHHHHHHHHHHHHHCCCCCCC
908	EEEERKFRDKYNNEYEDKEYQRTGRGKGHQF	-48.38	48.4	CHHHHHHHHHHHHHHCCHHHHHCCCCCCCCC
810	EEEKKKWLDQLNQEKEDQRYQREGMGKGHQP	-48.36	48.4	HHHHHHHHHHHHHHHHHHHHHHHHHCCCCCC
481	EQERRKQLEKYNNEYEDKEYQRTGRGKGNSN	-48.35	41.9	HHHHHHHHHHHHHHHHHHHHHHCCCCCCCCC
005	EEERRKQLDKYNNEAEQKRYQREGMGQGHQP	-48.34	45.2	HHHHHHHHHHHHHHHHHHHHHHHHHCCCCCC
527	EEEKRKFLDQFNNEYEDRYYQQKGFGKGHQS	-48.29	51.6	HHHHHHHHHHHHHHHHHHHHHHHCCCCCCCC
161	EEEKRKFLDQYNNEYEDRYYQQKGQGKGHSH	-48.29	48.4	HHHHHHHHHHHHHHHHHHHHHHHHCCCCCCC
304	EEEKRKQLEQFNNEYEDQFYQQKGFGKGHSS	-48.29	48.4	HHHHHHHHHHHHHHHHHHHHHHHCCCCCCCC
189	EEERKKQLEQKNNEEEDQRYQREGMGKGHQP	-48.29	45.2	HHHHHHHHHHHHHHHHHHHHHHHHCCCCCCC
091	EEERKKQLEQYNNEAEDERYQQQGFGKGHQS	-48.29	48.4	HHHHHHHHHHHHHHHHHHHHHHHCCCCCCCC
458	EEERKKQLEQYNNEAEDQRYQRQGFGKGHQN	-48.29	48.4	HHHHHHHHHHHHHHHHHHHHHHHHCCCCCCC
062	EEERKKQLEQYNNEAEDQRYQRYGFGKGHSS	-48.29	48.4	HHHHHHHHHHHHHHHHHHHHHHHCCCCCCCC
937	EEERKKQLEQYNNEEEDMRYQREGMGKGHQP	-48.29	45.2	HHHHHHHHHHHCHHHHHHHHHHHHHCCCCCC
048	EEERKKQLEQYNNEYEDEEYQRTGRGKGHQF	-48.29	45.2	HHHHHHHHHHHHHHHHHHHHHHHCCCCCCCC
312	EEERKKQLEQYNNEYEDQFYQQKGFGKGHQS	-48.29	48.4	HHHHHHHHHHHHHHHHHHHHHHHCCCCCCCC
020	EEERKKQLEQYSNEEEDMRYQREGKGKGHSS	-48.29	41.9	HHHHHHHHHHHHHHHHHHHHHHHCCCCCCCC
552	EEERRKQLEQFNNEFEDQYYQQKGQGKGHSH	-48.29	45.2	HHHHHHHHHHHHHHHHHHHHHHHHCCCCCCC
520	EEERRKQLEQINNEEEDQRYQRYGFGKGHQS	-48.29	41.9	HHHHHHHHHHHHHHHHHHHHHHHCCCCCCCC
634	EEERRKQLEQYNNESEDQRYQQYGFGKGHSS	-48.29	41.9	HHHHHHHHHHHHHHHHHHHHHHHCCCCCCCC
790	EEERRKQLEQYNNEYEDEEYQRKGRGKGHQP	-48.29	41.9	HHHHHHHHHHHHHHHHHHHHHHHHCCCCCCC
561	EQERRKQLEQYNNEAEDQRYQRYGFGKGHQS	-48.29	41.9	HHHHHHHHHHHHHHHHHHHHHHHCCCCCCCC
510	EEERRKQLEQYNNQSEDERYQRYGFGKGHQA	-48.27	38.7	HHHHHHHHHHHHHCCHHHHHHHHCCCCCCCC
298	EEEQKKQREKYNNEAEQKRYQREGKGRGHSH	-48.26	41.9	HHHHHHHHHHHHHHHHHHHHHHHCCCCCCCC
502	EEEKRKMLDQINQEEEDRRYQQQGMGKGHQP	-48.20	45.2	HHHHHHHHHHHHHHHHHHHHHHHHCCCCCCC
735	EEEERKFRDQFNNEAEDEEYQRRGMGKGHQP	-48.19	51.6	CHHHHHHHHHHHHHHHHHHHHHHHHCCCCCC
300	EEEKKKFLDQFNNEYEDQFYQQKGFGKGHQS	-48.19	58.1	HHHHHHHHHHHHHHHHHHHHHHHCCCCCCCC
863	EEEKKKFLDQFNNEYEDQYYQQKGQGKGHSH	-48.19	54.8	HHHHHHHHHHHHHHHHHHHHHHHHCCCCCCC
614	EEEKKKFLDQYNNEEEDQRYQREGMGKGHQP	-48.19	51.6	HHHHHHHHHHHCCHHHHHHHHHHHCCCCCCC
076	EEEKKKFLDQYNNESEDERYQQQGWGKGHNP	-48.19	51.6	HHHHHHHHHHHCCCHHHHHHHHHCCCCCCCC
704	EEEKRKFLDQFNNEEEDKRYQREGFGKGHQS	-48.19	51.6	HHHHHHHHHHHHHHHHHHHHHHHHCCCCCCC
759	EEEKRKFLDQFNNEFEDQYYQQKGQGKGHSH	-48.19	51.6	HHHHHHHHHHHHHHHHHHHHHHHHCCCCCCC
267	EEEKRKFLDQFNNEYEDKYYQQQGFGKGHQS	-48.19	51.6	HHHHHHHHHHHHHHHHHHHHHHHCCCCCCCC
574	EEEKRKFLDQFNNEYEDQYYQQKGFGKGHQS	-48.19	51.6	HHHHHHHHHHHHHHHHHHHHHHHCCCCCCCC
771	EEEKRKFLDQFNNEYEDQYYQQKGQGKGHSH	-48.19	51.6	HHHHHHHHHHHHHHHHHHHHHHHHCCCCCCC
642	EEEKRKFLDQYNNEAEDQRYQREGMGKGHQP	-48.19	51.6	HHHHHHHHHHHHHHHHHHHHHHHHHCCCCCC
146	EEEKRKFLDQYNNESEDQRYQQYGFGKGHQS	-48.19	48.4	HHHHHHHHHHHCCCHHHHHHHHHCCCCCCCC
657	EEEKRKFLDQYNNESEDQRYQRYGFGKGHSS	-48.19	48.4	HHHHHHHHHHHCCCCHHHHHHHHCCCCCCCC
368	EEEKRKFLDQYNNEYEDEEYQQTGRGKGHSS	-48.19	48.4	HHHHHHHHHHHHHHHHHHHHHHCCCCCCCCC
825	EEEKRKFLDQYNNEYEDEEYQRNGRGKGHSY	-48.19	48.4	HHHHHHHHHHHHHHHHHHHHHHCCCCCCCCC
874	EEEKRKFLDQYNNEYEDKEYQKEGRGKGHQP	-48.19	48.4	HHHHHHHHHHHHHHHHHHHHHHHHCCCCCCC
498	EEEQRKFEDQRNNEEEDKRYQQYGFGKGHQS	-48.19	45.2	CHHHHHHHHHHHHHHHHHHHHHHCCCCCCCC
783	EEEKKKFLDQMKNRKEDEEYQKQGWGKGHNP	-48.16	45.2	HHHHHHHHHHHHHHHHHHHHHHHCCCCCCCC
123	EEEKRKMLDQINNEKEDEEYQRYGFGKGTQN	-48.12	45.2	HHHHHHHHHHHHHHHHHHHHHHHCCCCCCCC
321	EEEERKQREQYNNEAEDQRYQREGKGKGHSS	-48.09	41.9	HHHHHHHHHHHHHHHHHHHHHHHCCCCCCCC
622	EEEKRKLLDKINNEAEDRRYQKEGMGKGHQP	-48.07	51.6	HHHHHHHHHHHHHHHHHHHHHHHHCCCCCCC
995	EEEKRKLLDKINNEEEDRRYQREGVGKGHNP	-48.07	48.4	HHHHHHHHHHHHHHHHHHHHHHHHCCCCCCC
750	EEEYRKWLDQYKNEAEDQRYQREGQGKGHQS	-48.06	45.2	HHHHHHHHHHHHHHHHHHHHHHHCCCCCCCC
488	EQEKRKILEQLNQEKEDKRYQREGKGKGHSS	-48.06	38.7	HHHHHHHHHHHHHHHHHHHHHHHCCCCCCCC
671	EEEKKKLLEKINNEEEDKRFQQQGFGKGKQN	-48.06	45.2	HHHHHHHHHHHHHHHHHHHHHHHCCCCCCCC
350	EEERRKQLEKYNNEEEDKRYQREGFGKGDSR	-48.04	51.6	HHHHHHHHHHHCHHHHHHHHHHHHCCCCCCC
957	EEEKRKLLDQINNEEEDQRYQQQGFGKGDSR	-48.01	51.6	HHHHHHHHHHHHHHHHHHHHHHHCCCCCCCC
096	EEEKRKLLDQINNEEEDQRYQQYGFGKGDSR	-48.01	51.6	HHHHHHHHHHHHHHHHHHHHHHHCCCCCCCC
357	EEEKKKFLDQYNNEWEDMRYQREGKGKGHSH	-47.95	51.6	HHHHHHHHHHHHHHHHHHHHHHHCCCCCCCC
285	EEEKRKLLEKLNNEKEDKRYQQQGRGKGHQP	-47.94	45.2	HHHHHHHHHHHHHHHHHHHHHHHHCCCCCCC
719	EEEARKNQEKYNNEYEDEEYQRKGRGKGDSR	-47.87	51.6	HHHHHHHHHHHHHHHHHHHHHHHCCCCCCCC
173	EEELKKILEQISQEEEDKRYQREGFGKGHSN	-47.83	41.9	HHHHHHHHHHHHHHHHHHHHHHHHCCCCCCC

960	EEEKRKLLDQINNEQEDEEYQRKGRGKGNQN	-47.81	45.2	HHHHHHHHHHHHHHHHHHHHHHHHCCCCCCC
665	EEEKRKLLDQINQEEEDQRYQQQGFGKGDSR	-47.81	51.6	HHHHHHHHHHHHHHHHHHHHHHHCCCCCCCC
968	EEEKKKMLDQFNNEYEDQFYQQKGFGKGHQN	-47.79	54.8	HHHHHHHHHHHHHHHHHHHHHHHCCCCCCCC
243	EEEKKKMLDQINNEAEDKRYQKEGFGKGHSS	-47.79	51.6	HHHHHHHHHHHHHHHHHHHHHHHHCCCCCCC
870	EEEKKKMLDQINNEEEDERYQQQGFGKGHQS	-47.79	48.4	HHHHHHHHHHHHHHHHHHHHHHHCCCCCCCC
087	EEEKKKMLDQINNEEEDKRYQQYGMGKGHQP	-47.79	48.4	HHHHHHHHHHHHHHHHHHHHHHHHCCCCCCC
325	EEEKRKMLDQINNEAEDKRYQREGKGKGHSH	-47.79	48.4	HHHHHHHHHHHHHHHHHHHHHHHCCCCCCCC
311	EEEKRKMLDQINNEAEDKRYQREGMGKGHQP	-47.79	48.4	HHHHHHHHHHHHHHHHHHHHHHHHHCCCCCC
416	EEEKRKMLDQINNEAEDKRYQRYGFGKGHQS	-47.79	48.4	HHHHHHHHHHHHHHHHHHHHHHHCCCCCCCC
571	EEEKRKMLDQINNEKEDEEYQRRGKGKGHSS	-47.79	45.2	HHHHHHHHHHHHHHHHHHHHHHHCCCCCCCC
615	EEEKRKMLDQINNEKEDKEYQRSGMGKGHQP	-47.79	48.4	HHHHHHHHHHHHHHHHHHHHHHHHCCCCCCC
931	EEEKRKMLDQINNREEDERYQQQGFGKGHQS	-47.77	41.9	HHHHHHHHHHHHHHHHHHHHHHHCCCCCCCC
902	EEERKKQLDQINNEKEDEEYQRYGFGKGHQN	-47.77	48.4	HHHHHHHHHHHHHHHHHHHHHHHCCCCCCCC
221	EEERRKQLDQINNEEEDERYQQQGMGKGHQP	-47.77	45.2	HHHHHHHHHHHHHHHHHHHHHHHHCCCCCCC
006	EEERRKQLDQINNEEEDKRYQQYGFGKGHSS	-47.77	45.2	HHHHHHHHHHHHHHHHHHHHHHHCCCCCCCC
455	EEERRKQLDQINNEEEDKRYQREGFGKGHQS	-47.77	45.2	HHHHHHHHHHHHHHHHHHHHHHHHCCCCCCC
098	EEERRKQLDQINNEEEDKRYQREGMGKGHQP	-47.77	45.2	HHHHHHHHHHHHHHHHHHHHHHHHCCCCCCC
181	EEERRKQLDQINNEKEDEEYQRRGWGKGHNP	-47.77	45.2	HHHHHHHHHHHHHHHHHHHHHHHCCCCCCCC
909	EEERRKQLDQINNEKEDEEYQRRGWGKGHTP	-47.77	45.2	HHHHHHHHHHHHHHHHHHHHHHHCCCCCCCC
725	EEERRKQLDQYNNEYEDEEYQRTGRGKGHQF	-47.77	45.2	HHHHHHHHHHHHHHHHHHHHHHHCCCCCCCC
758	EEEKKKILEQINNEEEDQRYQQYGWGKGHNP	-47.77	45.2	HHHHHHHHHHHHHHHHHHHHHHHCCCCCCCC
537	EEEKKKILEQINNEKEDEEYQRYGQGKGHQS	-47.77	45.2	HHHHHHHHHHHHHHHHHHHHHHHHHCCCCCC
953	EEEKRKILEQINNEAEDQRYQREGMGKGHQP	-47.77	45.2	HHHHHHHHHHHHHHHHHHHHHHHHHCCCCCC
794	EEEKRKILEQINNEEEDERYQRYGFGKGHQS	-47.77	41.9	HHHHHHHHHHHHHHHHHHHHHHHCCCCCCCC
236	EEEKRKILEQINNEEEDQRYQQYGFGKGHSS	-47.77	41.9	HHHHHHHHHHHHHHHHHHHHHHHCCCCCCCC
443	EEEKRKILEQINNEQEDQSYQRYGFGKGHQS	-47.77	41.9	HHHHHHHHHHHHHHHHHHHHHHHCCCCCCCC
749	EEEKRKILEQLNNEKEDKRYQREGMGKGHQP	-47.77	41.9	HHHHHHHHHHHHHHHHHHHHHHHHCCCCCCC
489	EEELKKILEQINNEEEDERYQQQGWGKGHTP	-47.77	45.2	HHHHHHHHHHHHHHHHHHHHHHHCCCCCCCC
738	EEELKKILEQLNNEQEDQKYQRYGFGKGHQS	-47.77	45.2	HHHHHHHHHHHHHHHHHHHHHHHCCCCCCCC
122	EEELRKILEQINNEEEDKRYQREGMGKGHQP	-47.77	41.9	HHHHHHHHHHHHHHHHHHHHHHHHCCCCCCC
731	EEEWRKILEQLNNEKEDQRYQREGMGKGHQP	-47.77	41.9	HHHHHHHHHHHHHHHHHHHHHHHHHCCCCCC
891	EQEKRKILEQINNEEEDQRYQRYGMGKGHQP	-47.77	38.7	HHHHHHHHHHHHHHHHHHHHHHHHCCCCCCC
403	EEEERKLQEKINNEAEDKRYQREGWGKGHNP	-47.75	45.2	HHHHHHHHHHHHHHHHHHHHHHHHCCCCCCC
407	EEEKKKLLEKINNEAEDKRYQREGMGKGHQP	-47.75	51.6	HHHHHHHHHHHHHHHHHHHHHHHHHCCCCCC
721	EEEKKKLLEKINNEEEDKRYQQQGFGKGHQS	-47.75	48.4	HHHHHHHHHHHHHHHHHHHHHHHHCCCCCCC
700	EEEKKKLLEKINNEEEDKRYQQQGFGKGHSS	-47.75	48.4	HHHHHHHHHHHHHHHHHHHHHHHCCCCCCCC
774	EEEKKKLLEKLNNEKEDEEYQRRGMGKGHQP	-47.75	48.4	HHHHHHHHHHHHHHHHHHHHHHHHHCCCCCC
010	EEEKKKLLEKLNNEKEDKRYQRQGFGKGHQS	-47.75	48.4	HHHHHHHHHHHHHHHHHHHHHHHHCCCCCCC
912	EEEKRKLLEKINNEAEDKRYQKEGFGKGHQS	-47.75	48.4	HHHHHHHHHHHHHHHHHHHHHHHHCCCCCCC
934	EEEKRKLLEKINNEAEDKRYQQQGMGKGHQP	-47.75	48.4	HHHHHHHHHHHHHHHHHHHHHHHHCCCCCCC
391	EEEKRKLLEKINNEAEDKRYQREGMGKGHQP	-47.75	48.4	HHHHHHHHHHHHHHHHHHHHHHHHHCCCCCC
569	EEEKRKLLEKINNEAEDKRYQRYGFGKGHQS	-47.75	48.4	HHHHHHHHHHHHHHHHHHHHHHHCCCCCCCC
660	EEEKRKLLEKINNEAEDKRYQRYGFGKGHSS	-47.75	48.4	HHHHHHHHHHHHHHHHHHHHHHHCCCCCCCC
880	EEEKRKLLEKINNEEEDKRYQQQGMGKGHQP	-47.75	45.2	HHHHHHHHHHHHHHHHHHHHHHHHCCCCCCC
674	EEEKRKLLEKINNEEEDKRYQREGFGKGHQS	-47.75	45.2	HHHHHHHHHHHHHHHHHHHHHHHHCCCCCCC
028	EEEKRKLLEKINNEEEDKRYQREGWGKGHNP	-47.75	45.2	HHHHHHHHHHHHHHHHHHHHHHHHCCCCCCC
944	EEEKRKLLEKINNEEEDKRYQRYGFGKGHSS	-47.75	45.2	HHHHHHHHHHHHHHHHHHHHHHHCCCCCCCC
804	EEEKRKLLEKINNEKEDKEYQKQGWGKGHNP	-47.75	45.2	HHHHHHHHHHHHHHHHHHHHHHHCCCCCCCC
214	EEEKRKLLEKINNEQEDEEYQRKGRGKGHQP	-47.75	45.2	HHHHHHHHHHHHHHHHHHHHHHHHCCCCCCC
492	EEEKRKLLEKINNEQEDKSYQRYGFGKGHQS	-47.75	45.2	HHHHHHHHHHHHHHHHCHHHHHHCCCCCCCC
315	EEEKRKLLEKLNNEKEDKKYQQEGHGKGHSR	-47.75	48.4	HHHHHHHHHHHHHHHHHHHHHHHCCCCCCCC
786	EEEKRKLLEKLNNEKEDKRYQQQGFGKGHSS	-47.75	45.2	HHHHHHHHHHHHHHHHHHHHHHHCCCCCCCC
279	EEEKRKLLEKLNNEKEDKRYQRYGMGKGHQP	-47.75	45.2	HHHHHHHHHHHHHHHHHHHHHHHHCCCCCCC
462	EEEKRKLLEKLNNEQEDKRYQRQGFGKGHQS	-47.75	45.2	HHHHHHHHHHHHHHHHHHHHHHHHCCCCCCC
380	EEERRKLLEKINNEEEDKRYQQQGFGKGHQS	-47.75	45.2	HHHHHHHHHHHHHHHHHHHHHHHHCCCCCCC
948	EQEKRKLLEKINNEQEDKSYQRYGFGKGHQS	-47.75	41.9	HHHHHHHHHHHHHHHHCHHHHHHCCCCCCCC
513	EEERRKQLDQYNNRYEDEYYQQKGQGKGHSH	-47.74	41.9	HHHHHHHHHHHHHHHHHHHHHHHHCCCCCCC
482	EEEQRKILEKINNEEEDRRKQRYGFGSGKQN	-47.73	38.7	HHHHHHHHHHHHHHHHHHHHHHCCCCCCCCC
791	EEEKRKLLDAINNREEDERYQQQGMGKGHQP	-47.71	41.9	HHHHHHHHHHHHHHHHHHHHHHHHCCCCCCC
782	EEERKKQLEQYNNEYEDEEYQRKGRGKGHQP	-47.68	45.2	HHHHHHHHHHHHHHHHHHHHHHHHCCCCCCC
013	EEERRKQLEQFNNEFEDKYYQQKGFGKGHQS	-47.68	45.2	HHHHHHHHHHHHHHHHHHHHHHHCCCCCCCC
164	EEERRKQLEQYNNEYEDEEYQRKGRGKGHQS	-47.68	41.9	HHHHHHHHHHHHHHHHHHHHHHHHCCCCCCC
363	EEERRKQLEQYNNEYEDQRYQQEGMGKGHQP	-47.68	41.9	HHHHHHHHHHHHHHHHHHHHHHHHCCCCCCC
474	EEERRKQLEKYNNEAEDRRYQREGMGKGHQP	-47.68	48.4	HHHHHHHHHHHHHHHHHHHHHHHHHCCCCCC
951	EEEERKFQEKYNQEWEDMRYQREGFGKGHQS	-47.67	45.2	CHHHHHHHHHHHHHHHHHHHHHHHHCCCCCC
845	EEEKRKFLDQQKNEEEDQRYQQYGFGKGHQS	-47.67	45.2	HHHHHHHHHHHHHHHHHHHHHHHCCCCCCCC
780	EEEQKKLYEKLNNEKEDKRYQREGKGKGHSH	-47.64	45.2	HHHHHHHHHHHHHHHHHHHHHHHCCCCCCCC

699	EEERKKQLDQLNNEKEDQRYQRYGFGKGHSP	-47.62	48.4	HHHHHHHHHHHHHHHHHHHHHHHCCCCCCCC
751	EEEQRKQQEQYNNEYEDQFYQQQGFGKGHQS	-47.61	41.9	HHHHHHHHHHHHHHHHHHHHHHHCCCCCCCC
014	EEEKRKFLDQKNNEEEDQRYQQYGFGKGHQP	-47.59	48.4	HHHHHHHHHHHHHHHHHHHHHHHCCCCCCCC
865	EEEKRKMLDKFNNEYEDKYYQQQGFGKGNQN	-47.57	51.6	HHHHHHHHHHHHHHHHHHHHHHHCCCCCCCC
406	EEERRKQLDQINQEAEDKRYQRYGMGKGHQP	-47.55	48.4	HHHHHHHHHHHHHHHHHHHHHHHHCCCCCCC
584	EEERRKQLDDYNQRWEDMRYQREGMGKGHQP	-47.54	41.9	HHHHHHHHHHHHHHHHHHHHHHHHHCCCCCC
656	EQELRKILEQYNNEEEDMRYQREGFGKGHQN	-47.53	38.7	HHHHHHHHHHHCHHHHHHHHHHHHCCCCCCC
683	EEEKRKLLEKINNEEEDERYQQQGKGKGHSH	-47.52	45.2	HHHHHHHHHHHHHHHHHHHHHHHCCCCCCCC
427	EEEEKKFQEKYNNESEDKRFQRYGFGKGKQN	-47.50	45.2	CHHHHHHHHHHHHHHHHHHHHHHCCCCCCCC
649	EEEKRKFLEKFNNEQEDKSFQRHGFGKGKQN	-47.50	48.4	HHHHHHHHHHHHHHHHHHHHHHHCCCCCCCC
914	EEEKRKFLEKFNQEEEDKEYQRVGRGKGHQF	-47.48	51.6	HHHHHHHHHHHHHHHHHHHHHHHHCCCCCCC
616	EEEKKKLLEKINNEKEDKEFQKQGQGKGKQS	-47.47	45.2	HHHHHHHHHHHHHHHHHHHHHHHHCCCCCCC
138	EEEQKKQREQYNQEYEDEEYQRKGRGKGDSR	-47.47	48.4	HHHHHHHHHHHHHHHHHHHHHHHCCCCCCCC
581	EEEKRKFLEKYNNESEDQRFQRYGFGKGKSR	-47.46	48.4	HHHHHHHHHHHHHCHHHHHHHHHCCCCCCCC
686	EEEKRKFLDQYNNEYEDEEYQRKGRGKGHQS	-47.46	48.4	HHHHHHHHHHHHHHHHHHHHHHHHCCCCCCC
099	EEEERKQRQQMNNEKEDEEYQRRGMGKGHQP	-47.46	38.7	HHHHHHHHHHHHHHHHHHHHHHHHHCCCCCC
939	EEEQKKFEDARNQREEDQRYQQQGFGKGHQS	-47.44	45.2	CHHHHHHHHHHHHHHHHHHHHHHCCCCCCCC
932	EEEKRKLLQKINQEKEDKEYQKQGFGKGHQS	-47.41	45.2	HHHHHHHHHHHHHHHHHHHHHHHHCCCCCCC
433	EEERRKQLDQFNNEKEQQRYQQQGFGQGHSS	-47.39	41.9	HHHHHHHHHHHHHHHHHHHHHHHCCCCCCCC
212	EEEKRKILEKINNEEEDRRYQQYGMGKGHQP	-47.37	45.2	HHHHHHHHHHHHHHHHHHHHHHHHCCCCCCC
135	EEEKRKILEKINNEKEDREYQKQGMGKGHQP	-47.37	45.2	HHHHHHHHHHHHHHHHHHHHHHHHCCCCCCC
768	EEEKKKLLEQINQEEEDQRYQQQGFGKGHQS	-47.28	45.2	HHHHHHHHHHHHHHHHHHHHHHHCCCCCCCC
729	EEEKKKLLEQLNQEKEDQSYQKEGFGKGHQS	-47.28	45.2	HHHHHHHHHHHHHHHHHHHHHHHHCCCCCCC
648	EEEKRKLLEQINQEQEDEEYQRKGRGKGHQS	-47.28	41.9	HHHHHHHHHHHHHHHHHHHHHHHHCCCCCCC
964	EEERRKLLEQINQEEEDKRYQREGMGKGHQP	-47.28	41.9	HHHHHHHHHHHHHHHHHHHHHHHHCCCCCCC
259	EEEKRKMLDQINQEAEDQRYQRQGFGKGHQS	-47.25	48.4	HHHHHHHHHHHHHHHHHHHHHHHHCCCCCCC
621	EEEKRKFLDQYNNEEEDMRYQREGFGKGNQN	-47.25	48.4	HHHHHHHHHHHCHHHHHHHHHHHHCCCCCCC
139	EEEKRKLLEKINNEKEDEEYQRYGFGKGHQN	-47.20	45.2	HHHHHHHHHHHHHHHHHHHHHHHCCCCCCCC
253	EEEKRKFLEQLNNDEEDERYQQQGFGKGHSS	-47.20	41.9	HHHHHHHHHHHHHHHHHHHHHHHCCCCCCCC
400	EEEQRKFEEQRNNDEEDQRYQQQGMGKGHQP	-47.20	38.7	HHHHHHHHHHHHHHHHHHHHHHHHCCCCCCC
501	EEEKKKFLEKQNNEEEDKRYQQQGMGKGHQP	-47.19	51.6	HHHHHHHHHHHHHHHHHHHHHHHHCCCCCCC
152	EEEKKKFLEKYNNESEDQRYQRYGFGKGHQN	-47.19	51.6	HHHHHHHHHHHHHCHHHHHHHHHCCCCCCCC
272	EEEKKKFLEKYNNEYEDEEYQRKGRGKGHQS	-47.19	51.6	HHHHHHHHHHHHHHHHHHHHHHHHCCCCCCC
000	EEEKKKFLEKYSNESEDKRYQQQGFGKGHQS	-47.19	48.4	HHHHHHHHHHHHHHHHHHHHHHHHCCCCCCC
017	EEEKRKFEEKRNNEEEDKRYQQYGMGKGHQP	-47.19	45.2	HHHHHHHHHHHHHHHHHHHHHHHHCCCCCCC
976	EEEKRKFLEKFNNEFEDKYYQQKGFGKGHQP	-47.19	51.6	HHHHHHHHHHHHHHHHHHHHHHHCCCCCCCC
526	EEEKRKFLEKFNNEYEDKYYQQKGFGKGHQS	-47.19	51.6	HHHHHHHHHHHHHHHHHHHHHHHCCCCCCCC
392	EEEKRKFLEKFNNEYEDKYYQQKGQGKGHSH	-47.19	51.6	HHHHHHHHHHHHHHHHHHHHHHHHCCCCCCC
079	EEEKRKFLEKKNNEEEDKRYQQYGMGKGHQP	-47.19	48.4	HHHHHHHHHHHHHHHHHHHHHHHHCCCCCCC
464	EEEKRKFLEKMKNEKEDEEYQRYGFGKGHSS	-47.19	45.2	HHHHHHHHHHHHHHHHHHHHHHHCCCCCCCC
360	EEEKRKFLEKYNNEAEDERYQRYGFGKGHQS	-47.19	51.6	HHHHHHHHHHHHHHHHHHHHHHHCCCCCCCC
945	EEEKRKFLEKYNNEAEDKRYQREGKGKGHSH	-47.19	51.6	HHHHHHHHHHHHHHHHHHHHHHHCCCCCCCC
280	EEEKRKFLEKYNNEAEDKRYQREGMGKGHQP	-47.19	51.6	HHHHHHHHHHHHHHHHHHHHHHHHHCCCCCC
387	EEEKRKFLEKYNNEAEDKRYQRQGFGKGHQS	-47.19	51.6	HHHHHHHHHHHHHHHHHHHHHHHHCCCCCCC
651	EEEKRKFLEKYNNEAEDKRYQRYGFGKGHQS	-47.19	51.6	HHHHHHHHHHHHHHHHHHHHHHHCCCCCCCC
274	EEEKRKFLEKYNNESEDKRYQQQGMGKGHQP	-47.19	48.4	HHHHHHHHHHHHHHHHHHHHHHHHCCCCCCC
991	EQEKRKFLEKYNNEYEDKYYQQNGQGKGHSY	-47.19	45.2	HHHHHHHHHHHHHHHHHHHHHHHCCCCCCCC
895	EQEWRKFIEKMKNEKEDKEYQKQGFGKGHSS	-47.19	38.7	CHHHHHHHHHHHHHHHHHHHHHHCCCCCCCC
922	EEEKRKFLDKYNNESEHQRYQQQGWGQGHNP	-47.18	45.2	HHHHHHHHHHHHHHHHHHHHHHHHCCCCCCC
920	EEELKKFLEKMNNEQEDKRYQKEGFGKGHSS	-47.17	51.6	HHHHHHHHHHHHHHHHHHHHHHHHCCCCCCC
353	EEERRKQLEQFNNEYEDEEYQRKGRGKGHSS	-47.16	45.2	HHHHHHHHHHHHHHHHHHHHHHHCCCCCCCC
107	EEERRKQLEQYNNEYEDQEYQRNGRGKGHSY	-47.16	41.9	HHHHHHHHHHHHHHHHHHHHHHCCCCCCCCC
955	EQEERKKLEQYKNEYEDKQYQKEGFGKGHQS	-47.16	35.5	CHHHHHHHHHHHHHHHHHHHHHHHCCCCCCC
093	EEEKKKLLEQIENEREDQEYQRKGMGKGHQP	-47.15	41.9	HHHHHHHHHHHHHHHHHHHHHHHHCCCCCCC
002	EEEKRKMLEQINQDEEDKRYQREGFGKGHQS	-47.10	38.7	HHHHHHHHHHHHHHHHHHHHHHHHCCCCCCC
620	EEEEKKLQEQINNEEEDRRYQREGQGKGHQS	-47.09	41.9	CHHHHHHHHHHHHHHHHHHHHHHHCCCCCCC
340	EEEERKLQEQINNEEEDRRYQQYGKGKGHSH	-47.09	38.7	HHHHHHHHHHHHHHHHHHHHHHHHCCCCCCC
562	EEEKKKLLEQINNEAEDRRYQRYGFGKGHQS	-47.09	48.4	HHHHHHHHHHHHHHHHHHHHHHHCCCCCCCC
593	EEEKKKLLEQLNNEKEDRRYQKEGQGKGHQS	-47.09	45.2	HHHHHHHHHHHHHHHHHHHHHHHCCCCCCCC
195	EEEKRKLLEQINNEEEDRNYQREGKGKGHSH	-47.09	41.9	HHHHHHHHHHHHHHHHHHHHHHHCCCCCCCC
839	EEEKKKMLEKINQEAEDKRYQRYGFGKGHQN	-47.09	51.6	HHHHHHHHHHHHHHHHHHHHHHHCCCCCCCC
542	EEEKKKMLEKINQEKEDEEYQRRGMGKGHQP	-47.09	48.4	HHHHHHHHHHHHHHHHHHHHHHHHHCCCCCC
814	EQERRKQLEKFNNEYEDKFFQKQGFGKGKQN	-47.08	45.2	HHHHHHHHHHHHHHHHHHHHHHHCCCCCCCC
952	EEERRKQLEQINQDAEDQRYQKEGFGKGHQS	-47.07	41.9	HHHHHHHHHHHHHHHHHHHHHHHHCCCCCCC
546	EEEYKKWLEKFKNEYEDKYYQQKGFGKGHQS	-47.06	48.4	HHHHHHHHHHHHHHHHHHHHHHHCCCCCCCC
972	EEEKRKMLDQYNNEWEDEEYQRKGRGKGDSR	-47.06	51.6	HHHHHHHHHHHHHHHHHHHHHHHCCCCCCCC
289	EEEKKKWLEKLNNEKEDKRYQRQGFGKGHQS	-47.06	48.4	HHHHHHHHHHHHHHHHHHHHHHHHCCCCCCC

628	EEEKRKWLEKLNNEKEDKEYQKQGFGKGHQS	-47.06	45.2	HHHHHHHHHHHHHHHHHHHHHHHCCCCCCCC
258	EEERKKQLEQYNNEYEDEEYQRKGRGKGDSR	-47.04	51.6	HHHHHHHHHHHHHHHHHHHHHHHCCCCCCCC
159	EEERRKQLQQYNNESEDQRYQQQGMGKGHQP	-47.04	41.9	HHHHHHHHHHHHHCHHHHHHHHHCCCCCCCC
196	EEELRKILDQINNEKEQREYQRYGFGQGHQS	-47.04	38.7	HHHHHHHHHHHHHHHHHHHHHHHCCCCCCCC
549	EQEKRKILEQLNNEKEDQRYQRYGFGKGDSR	-47.03	45.2	HHHHHHHHHHHHHHHHHHHHHHHCCCCCCCC
954	EEEKRKFLEQFNQEYEDQFFQQKGFGKGKQN	-47.02	48.4	HHHHHHHHHHHHHHHHHHHHHHHCCCCCCCC
815	EEEKRKLLEKINNEEEDKRYQQYGFGKGDSR	-47.02	51.6	HHHHHHHHHHHHHHHHHHHHHHHCCCCCCCC
041	EEEKRKLLEQINNDEEDRRYQRYGFGKGHQN	-47.00	38.7	HHHHHHHHHHHHHHHHHHHHHHHCCCCCCCC
193	EEEKRKLLEQINNRKEDEEYQRNGSGKGHSY	-47.00	38.7	HHHHHHHHHHHHHHHHHHHHHHCCCCCCCCC
355	EEEKRKFLDQFNNEYEHQFKQQKGFGSGKQN	-46.99	45.2	HHHHHHHHHHHHHHHHHHHHHHHCCCCCCCC
008	EEEKKKLLEQINNEAEDKRYQREGKGKGHSH	-46.99	48.4	HHHHHHHHHHHHHHHHHHHHHHHCCCCCCCC
890	EEEKKKLLEQINNEAEDKRYQRYGFGKGHSN	-46.99	48.4	HHHHHHHHHHHHHHHHHHHHHHHCCCCCCCC
831	EEEKKKLLEQINNEEEDERYQQQGFGKGHQS	-46.99	45.2	HHHHHHHHHHHHHHHHHHHHHHHCCCCCCCC
166	EEEKKKLLEQINNEEEDQRYQQYGFGKGHQS	-46.99	45.2	HHHHHHHHHHHHHHHHHHHHHHHCCCCCCCC
706	EEEKKKLLEQINNEEEDQRYQQYGWGKGHTP	-46.99	45.2	HHHHHHHHHHHHHHHHHHHHHHHCCCCCCCC
369	EEEKKKLLEQINNEEEDQRYQREGFGKGHQS	-46.99	45.2	HHHHHHHHHHHHHHHHHHHHHHHCCCCCCCC
260	EEEKKKLLEQINNEKEDEEYQRRGKGKGHSH	-46.99	45.2	HHHHHHHHHHHHHHHHHHHHHHHCCCCCCCC
170	EEEKKKLLEQINNEYEDEFYQQKGFGKGHQS	-46.99	48.4	HHHHHHHHHHHHHHHHHHHHHHHCCCCCCCC
219	EEEKKKLLEQLNNEKEDKRYQRQGFGKGHSS	-46.99	45.2	HHHHHHHHHHHHHHHHHHHHHHHCCCCCCCC
180	EEEKRKLLEQINNEAEDERYQQQGFGKGHQS	-46.99	45.2	HHHHHHHHHHHHHHHHHHHHHHHCCCCCCCC
905	EEEKRKLLEQINNEAEDKRYQRYGFGKGHQS	-46.99	45.2	HHHHHHHHHHHHHHHHHHHHHHHCCCCCCCC
288	EEEKRKLLEQINNEAEDQRYQRQGFGKGHSN	-46.99	45.2	HHHHHHHHHHHHHHHHHHHHHHHCCCCCCCC
892	EEEKRKLLEQINNEEEDERYQQQGKGKGHSS	-46.99	41.9	HHHHHHHHHHHHHHHHHHHHHHHCCCCCCCC
519	EEEKRKLLEQINNEEEDKRYQQYGFGKGHQS	-46.99	41.9	HHHHHHHHHHHHHHHHHHHHHHHCCCCCCCC
390	EEEKRKLLEQINNEEEDKRYQREGKGKGHSH	-46.99	41.9	HHHHHHHHHHHHHHHHHHHHHHHCCCCCCCC
878	EEEKRKLLEQINNEEEDKRYQREGMGKGHQP	-46.99	41.9	HHHHHHHHHHHHHHHHHHHHHHHCCCCCCCC
679	EEEKRKLLEQINNEEEDKRYQREGQGKGHQS	-46.99	41.9	HHHHHHHHHHHHHHHHHHHHHHHCCCCCCCC
869	EEEKRKLLEQINNEEEDKRYQRYGMGKGHQP	-46.99	41.9	HHHHHHHHHHHHHHHHHHHHHHHCCCCCCCC
370	EEEKRKLLEQINNEEEDQRYQQQGWGKGHNP	-46.99	41.9	HHHHHHHHHHHHHHHHHHHHHHHCCCCCCCC
769	EEEKRKLLEQINNEEEDQRYQQYGWGKGHNP	-46.99	41.9	HHHHHHHHHHHHHHHHHHHHHHHCCCCCCCC
154	EEEKRKLLEQINNEEEDQRYQREGFGKGHQS	-46.99	41.9	HHHHHHHHHHHHHHHHHHHHHHHCCCCCCCC
547	EEEKRKLLEQINNEEEDQRYQRYGFGKGHSN	-46.99	41.9	HHHHHHHHHHHHHHHHHHHHHHHCCCCCCCC
109	EEEKRKLLEQINNEKEDEEYQRRGMGKGHQP	-46.99	41.9	HHHHHHHHHHHHHHHHHHHHHHHHCCCCCCC
023	EEEKRKLLEQINNEKEDEEYQRYGMGKGHQP	-46.99	41.9	HHHHHHHHHHHHHHHHHHHHHHHHCCCCCCC
284	EEEKRKLLEQINNEKEDKEYQKQGFGKGHQS	-46.99	41.9	HHHHHHHHHHHHHHHHHHHHHHHCCCCCCCC
476	EEEKRKLLEQINNEKEDQEYQKQGMGKGHQP	-46.99	41.9	HHHHHHHHHHHHHHHHHHHHHHHCCCCCCCC
762	EEEKRKLLEQINNEKEDQEYQRAGFGKGHSN	-46.99	41.9	HHHHHHHHHHHHHHHHHHHHHHHCCCCCCCC
147	EEEKRKLLEQINNEQEDERYQRQGFGKGHQS	-46.99	41.9	HHHHHHHHHHHHHHHHHHHHHHHCCCCCCCC
744	EEEKRKLLEQINNEQEDQRYQREGMGKGHQP	-46.99	41.9	HHHHHHHHHHHHHHHHHHHHHHHCCCCCCCC
038	EEEKRKLLEQLNNEKEDKRYQREGMGKGHQP	-46.99	41.9	HHHHHHHHHHHHHHHHHHHHHHHCCCCCCCC
770	EEEKRKLLEQLNNEKEDMRYQQQGFGKGHQS	-46.99	41.9	HHHHHHHHHHHHHHHHHHHHHHHCCCCCCCC
073	EEEKRKLLEQLNNEKEDQRYQKEGFGKGHQS	-46.99	41.9	HHHHHHHHHHHHHHHHHHHHHHHCCCCCCCC
150	EEEKRKLLEQLNNEKEDQRYQRYGFGKGHQS	-46.99	41.9	HHHHHHHHHHHHHHHHHHHHHHHCCCCCCCC
595	EEEKRKLLEQLNNEKEDQRYQRYGFGKGHSS	-46.99	41.9	HHHHHHHHHHHHHHHHHHHHHHHCCCCCCCC
365	EEEWRKLMEQYNNEAEDQRYQREGMGKGHQP	-46.99	41.9	HHHHHHHHHHHHHHHHHHHHHHHHCCCCCCC
343	EEEWRKLMEQYNNEYEDQEYQRTGRGKGHSF	-46.99	38.7	HHHHHHHHHHHHHHHHHHHHHHCCCCCCCCC
225	EQEARKLQEQINNEEEDKRYQREGVGKGHNP	-46.99	38.7	HHHHHHHHHHHHHHHHHHHHHHHCCCCCCCC
779	EQEKRKLLEQINNEAEDKRYQKEGWGKGHNP	-46.99	41.9	HHHHHHHHHHHHHHHHHHHHHHHCCCCCCCC
108	EQEKRKLLEQINNEEEDQRYQRYGFGKGHQS	-46.99	38.7	HHHHHHHHHHHHHHHHHHHHHHHCCCCCCCC
974	EEEKRKMLEKINNEKEHEEYQRYGFGRGHQS	-46.96	38.7	HHHHHHHHHHHHHHHHHHHHHHHCCCCCCCC
691	EEEKRKFLEKYNNEYEDEEYQRNGRGKGHSY	-46.95	48.4	HHHHHHHHHHHHHHHHHHHHHHCCCCCCCCC
672	EEEQRKYEQKRNQEEEDKYYQQYGQGKGHSH	-46.94	41.9	CHHHHHHHHHHHHHHHHHHHHHHHCCCCCCC
301	EEEERKFQDQYNNEYEDEEYQRKGRGKGDSR	-46.94	51.6	CHHHHHHHHHHHHHHHHHHHHHHCCCCCCCC
439	EEEKKKLLEQIEQEREDSEYQQKGFGKGHQS	-46.93	41.9	HHHHHHHHHHHHHHHHHHHHHHHCCCCCCCC
373	EEERRKQLEKFNQEYEDKFYQKEGFGKGHQN	-46.92	51.6	HHHHHHHHHHHHHHHHHHHHHHHCCCCCCCC
536	EEEKRKFLQQYNQDYEDQEYQRNGRGKGHQY	-46.85	41.9	HHHHHHHHHHHHHHHHHHHHHHHCCCCCCCC
393	EEEKKKLLEKINNEAEDKRYQRQGFGKGNSN	-46.82	51.6	HHHHHHHHHHHHHHHHHHHHHHHCCCCCCCC
582	EEEKRKLLDKINNEAEQKRYQREGWGQGHNP	-46.80	45.2	HHHHHHHHHHHHHHHHHHHHHHHCCCCCCCC
587	EEEKRKLLDKINNEKEQEEYQRRGMGQGHQP	-46.80	41.9	HHHHHHHHHHHHHHHHHHHHHHHHCCCCCCC
451	DEERKKMLEKINNEEEDKRYQQQGMGKGHQP	-46.79	45.2	HHHHHHHHHHHHHHHHHHHHHHHCCCCCCCC
167	DEERKKMLEKINNEKEDKEYQKRGWGKGHNP	-46.79	45.2	HHHHHHHHHHHHHHHHHHHHHHHCCCCCCCC
785	EEEKKKMLEKFNNEYEDKYYQQKGQGKGHSH	-46.79	51.6	HHHHHHHHHHHHHHHHHHHHHHHCCCCCCCC
460	EEEKKKMLEKINNEAEDKRYQRYGFGKGHSS	-46.79	51.6	HHHHHHHHHHHHHHHHHHHHHHHCCCCCCCC
318	EEEKKKMLEKINNEAEDKRYQRYGMGKGHQP	-46.79	51.6	HHHHHHHHHHHHHHHHHHHHHHHCCCCCCCC
398	EEEKKKMLEKINNEKEDKEYQRGGFGKGHQP	-46.79	48.4	HHHHHHHHHHHHHHHHHHHHHHCCCCCCCCC
252	EEEKKKMLEKINNEKEDKEYQRSGMGKGHQP	-46.79	51.6	HHHHHHHHHHHHHHHHHHHHHHHCCCCCCCC
617	EEEKKKMLEKYNNEAEDIYYQKQGFGKGHSS	-46.79	51.6	HHHHHHHHHHHHHHHHHHHHHHHCCCCCCCC

711	EEEKKKMLEKYNNEYEDEEYQRKGRGKGHQP	-46.79	48.4	HHHHHHHHHHHHHHHHHHHHHHHHHHCCCCCCC
638	EEEKRKMLEKFNNEFEDKYYQQYGFGKGHSS	-46.79	48.4	HHHHHHHHHHHHHHHHHHHHHHHHHCCCCCCCC
958	EEEKRKMLEKFNNEYEDKFYQQQGFGKGHQS	-46.79	51.6	HHHHHHHHHHHHHHHHHHHHHHHHHCCCCCCCC
378	EEEKRKMLEKFNNEYEDKYYQQKGQGKGHSH	-46.79	48.4	HHHHHHHHHHHHHHHHHHHHHHHHHHCCCCCCC
993	EEEKRKMLEKINNEAEDKRYQRYGFGKGHQS	-46.79	48.4	HHHHHHHHHHHHHHHHHHHHHHHHHCCCCCCCC
901	EEEKRKMLEKINNEEEDKRYQQYGMGKGHQP	-46.79	45.2	HHHHHHHHHHHHHHHHHHHHHHHHHHCCCCCCC
468	EEEKRKMLEKINNEKEDKEYQKQGFGKGHQS	-46.79	45.2	HHHHHHHHHHHHHHHHHHHHHHHHHHCCCCCCC
539	EEEQRKMLEKINNEEEDKRYQRQGFGKGHQN	-46.79	45.2	HHHHHHHHHHHHHHHHHHHHHHHHHHCCCCCCC
643	EQEKRKMLEKINNEKEDKEYQKQGMGKGHQP	-46.79	41.9	HHHHHHHHHHHHHHHHHHHHHHHHHHCCCCCCC
377	EEERRKQLDQMKNEKEQEEYQRYGFGQGHSN	-46.78	35.5	HHHHHHHHHHHHHHHHHHHHHHHHHCCCCCCCC
702	EEERKKQLEKINNEAEDKRYQREGMGKGHQP	-46.77	51.6	HHHHHHHHHHHHHHHHHHHHHHHHHHHCCCCCC
058	EEERKKQLEKYNNEEEDMRYQREGFGKGHQS	-46.77	48.4	HHHHHHHHHHHCCHHHHHHHHHHHHHCCCCCCC
913	EEERRKQLEKINNEAEDKRYQQYGFGKGHQS	-46.77	48.4	HHHHHHHHHHHHHHHHHHHHHHHHHCCCCCCCC
234	EEERRKQLEKINNEAEDKRYQQYGFGKGHSS	-46.77	48.4	HHHHHHHHHHHHHHHHHHHHHHHHHCCCCCCCC
882	EEERRKQLEKINNEEEDKRYQREGQGKGHQS	-46.77	45.2	HHHHHHHHHHHHHHHHHHHHHHHHHHCCCCCCC
975	EEERRKQLEKYNNESEDKRYQRYGFGKGHSS	-46.77	45.2	HHHHHHHHHHHHHHHHHHHHHHHHCCCCCCCCC
856	EEERRKQLEKYNNEYEDEEYQRKGRGKGHQS	-46.77	45.2	HHHHHHHHHHHHHHHHHHHHHHHHHHCCCCCCC
813	EQERRKQLEKINNEAEDKRYQREGMGKGHQP	-46.77	45.2	HHHHHHHHHHHHHHHHHHHHHHHHHHHCCCCCC
739	EEQKRKFLEKKNNEEEDKRYQQQGWGKGHTP	-46.75	51.6	HHHHHHHHHHHHHHHHHHHHHHHHHCCCCCCCC
555	EEEKRKLLEQINNEAEDKRYQREGMGKGHQP	-46.75	45.2	HHHHHHHHHHHHHHHHHHHHHHHHHHHCCCCCC
320	EEEKKKWLEKLNNEKEDMRYQREGMGKGHQP	-46.73	48.4	HHHHHHHHHHHHHHHHHHHHHHHHHHHCCCCCC
296	EEEEKKFQEQYNNEYEDEEFQRKGRGKGKSN	-46.73	41.9	CHHHHHHHHHHHHHHHHHHHHHHHHCCCCCCCC
231	EEEKKKFLEQYNNEYEDQQFQKEGFGKGKSN	-46.73	45.2	HHHHHHHHHHHHHHHHHHHHHHHHHHCCCCCCC
802	EEEKRKFLEQMKNEKEDQEFQRAGFGKGKQN	-46.73	38.7	HHHHHHHHHHHHHHHHHHHHHHHHHHCCCCCCC
973	EEEERKFREQYNQEYEDEEYQRKGRGKGHQS	-46.72	41.9	CHHHHHHHHHHHHHHHHHHHHHHHHHCCCCCCC
484	EEEKKKFLEQYNQEYEDEEYQRKGRGKGHQS	-46.72	48.4	HHHHHHHHHHHHHHHHHHHHHHHHHHCCCCCCC
894	EEEKRKFLEQFNQEFEDQFYQQKGFGKGHQS	-46.72	51.6	HHHHHHHHHHHHHHHHHHHHHHHHHCCCCCCCC
512	EEEKRKFLEQYNQEAEDERYQQQGMGKGHQP	-46.72	48.4	HHHHHHHHHHHHHHHHHHHHHHHHHHCCCCCCC
401	EEEKRKFLEQYNQEEEDMRYQREGFGKGHSS	-46.72	45.2	HHHHHHHHHHHHHHHHHHHHHHHHHHHCCCCCC
129	EEEKRKFLEQYNQESEDQRYQQQGMGKGHQP	-46.72	45.2	HHHHHHHHHHHHHHHHHHHHHHHHHHCCCCCCC
893	EEEKRKFLEQYNQESEDQRYQRYGFGKGHQS	-46.72	45.2	HHHHHHHHHHHHHHHHHHHHHHHHHCCCCCCCC
755	EEEKRKFLEQYNQEYEDEEYQRVGRGKGHSY	-46.72	45.2	HHHHHHHHHHHHHHHHHHHHHHHHHHCCCCCCC
250	EEELKKLLEQIEQEREDQEYQQKGFGKGDSR	-46.71	48.4	HHHHHHHHHHHHHHHHHHHHHHHHHCCCCCCCC
329	EEEKRKLLEQINNEKEDEEYQRRGWGKGHTP	-46.66	41.9	HHHHHHHHHHHHHHHHHHHHHHHHHCCCCCCCC
990	EEEKRKLLEQLNNEKEDQRYQREGMGKGHQP	-46.66	41.9	HHHHHHHHHHHHHHHHHHHHHHHHHHHCCCCCC
144	EQEKRKFLEQMKNEKEDEEFQRRGMGKGKQP	-46.66	35.5	HHHHHHHHHHHHHHHHHHHHHHHHHHCCCCCCC
603	EEEKKKMLDQINNEKEDRRYQQQGFGKGDSR	-46.66	54.8	HHHHHHHHHHHHHHHHHHHHHHHHHCCCCCCCC
198	EEEKKKLLEKINQEAEDRRYQREGMGKGHQP	-46.65	51.6	HHHHHHHHHHHHHHHHHHHHHHHHHHHCCCCCC
644	EEEKRKMLEQINQEEEDQRYQRYGFGKGTQN	-46.65	41.9	HHHHHHHHHHHHHHHHHHHHHHHHHCCCCCCCC
800	EEERKKQLEKINNEAEDKRYQRYGFGKGHQS	-46.62	51.6	HHHHHHHHHHHHHHHHHHHHHHHHHCCCCCCCC
075	EEEKRKWLEQLNNEKEDKRKQREGRGSGKQR	-46.61	38.7	HHHHHHHHHHHHHHHHHHHHHHHHHCCCCCCCC
467	EEEKRKFLEKYNNEYEDKFYQQKGFGKGHQS	-46.59	51.6	HHHHHHHHHHHHHHHHHHHHHHHHHCCCCCCCC
989	EEEKKKLLEKINNEEEHKRKQREGFGSGKQN	-46.56	38.7	HHHHHHHHHHHHHHHHHHHHHHHHHHHCCCCCC
316	EEEKRKFLEKYNQESEDKRYQQQGFGKGNSN	-46.55	48.4	HHHHHHHHHHHHHHHHHHHHHHHHHCCCCCCCC
257	EEEKRKFLDKYNQEEEQKRYQREGKGQGHSH	-46.54	45.2	HHHHHHHHHHHHHHHHHHHHHHHHHHCCCCCCC
404	EEERKKQLDQINNEQEHEEKQRKGRGSGKSR	-46.54	41.9	HHHHHHHHHHHHHHHHHHHHHHHHHCCCCCCCC
188	EEERRKQLEKINNEEEDKRYQRYGFGKGHQS	-46.53	45.2	HHHHHHHHHHHHHHHHHHHHHHHHHCCCCCCCC
675	EEEARKFQEQYNNEAEDRRYQRYGFGKGHQN	-46.53	48.4	HHHHHHHHHHHHHHHHHHHHHHHHHCCCCCCCC
323	EEEKKKFLEQKNNEEEDRRYQQYGMGKGHQP	-46.53	48.4	HHHHHHHHHHHHHHHHHHHHHHHHHHCCCCCCC
636	EEEKRKFLEQFNNEFEDRRYQQEGFGKGHQS	-46.53	48.4	HHHHHHHHHHHHHHHHHHHHHHHHHHCCCCCCC
847	EEEKRKFLEQYNNEEEDRRYQQQGNGKGHSH	-46.53	45.2	HHHHHHHHHHHCHHHHHHHHHHHHHCCCCCCCC
345	EEEKRKLLEKINNEAEDKRYQRQGFGKGDSR	-46.51	54.8	HHHHHHHHHHHHHHHHHHHHHHHHHCCCCCCCC
310	EEEKRKLLEKINNEQEDEEYQRKGFGKGDSR	-46.51	51.6	HHHHHHHHHHHHHHHHHHHHHHHHHCCCCCCCC
576	EQEARKQLEKYKNEYEQEEYQRKGRGQGHQP	-46.50	35.5	HHHHHHHHHHHHHHHHHHHHHHHHHHCCCCCCC
194	EEEKRKMLEQIEQEREDKEYQRQGFGKGHQS	-46.49	38.7	HHHHHHHHHHHHHHHHHHHHHHHHHHCCCCCCC
577	EEEKRKMLEKFNNEYEDEEYQRKGRGKGHQS	-46.47	48.4	HHHHHHHHHHHHHHHHHHHHHHHHHHCCCCCCC
348	EEEKKKLLEQINNEKEDQEYQRSGMGKGHQP	-46.47	48.4	HHHHHHHHHHHHHHHHHHHHHHHHHHCCCCCCC
121	EEEKRKLLEQLNNEKEDKRYQQQGQGKGHQS	-46.47	41.9	HHHHHHHHHHHHHHHHHHHHHHHHHCCCCCCCC
429	EEEWKKLMEQYNNEYEDQEYQRNGRGKGHSY	-46.47	41.9	HHHHHHHHHHHHHHHHHHHHHHHHCCCCCCCCC
589	EQEYRKWLEKYKNEAEDKRYQRYGMGKGHQP	-46.47	41.9	HHHHHHHHHHHHHHHHHHHHHHHHHHCCCCCCC
747	EQERRKFLEQYNNRYEDEEYQKEGMGKGHQP	-46.46	38.7	HHHHHHHHHHHHHHHHHHHHHHHHHHCCCCCCC
290	EEEKRKWLEKLNNEKEDEEYQRNGRGKGHSY	-46.46	45.2	HHHHHHHHHHHHHHHHHHHHHHHHHCCCCCCCC
887	EEEKRKFLEKFNNEKEDKRYQQQGFGKGDSR	-46.45	58.1	HHHHHHHHHHHHHHHHHHHHHHHHHCCCCCCCC
437	EEEKRKFLEKYNNEAEDKRYQQQGFGKGDSR	-46.45	58.1	HHHHHHHHHHHHHHHHHHHHHHHHHCCCCCCCC
222	EEEKRKILDQINNRQEQERYQREGQGQGHSS	-46.43	35.5	HHHHHHHHHHHHHHHHHHHHHHHHHCCCCCCCC
877	EEEKRKILEKINQEAEDRRYQREGKGKGDSR	-46.42	54.8	HHHHHHHHHHHHHHHHHHHHHHHHHCCCCCCCC
319	EEEAKKFEEQFRNEYEDQRYQQEGFGKGHSS	-46.42	48.4	HHHHHHHHHHHHHHHHHHHHHHHHHHCCCCCCC

459	EEEARKFKEQYDNEYEDQEYQRTGRGKGHQF	-46.42	41.9	HHHHHHHHHHHHHHHHHHHHHHCCCCCCCCC
456	EEEERKFQEQYNNESEDQRYQRYGFGKGHQS	-46.42	41.9	CHHHHHHHHHHHCCHHHHHHHHHCCCCCCCC
083	EEEERKFQEQYNNEYEDEEYQRNGRGKGHSY	-46.42	41.9	CHHHHHHHHHHHHHHHHHHHHHCCCCCCCCC
495	EEEERKFREQKNNEEEDERYQRQGWGKGHTP	-46.42	41.9	CHHHHHHHHHHHHHHHHHHHHHHCCCCCCCC
399	EEEERKFREQYNNEAEDQRYQQQGMGKGHQP	-46.42	45.2	CHHHHHHHHHHHHHHHHHHHHHHHCCCCCCC
533	EEEKKKFLEQFNNEEEDKRYQREGMGKGHQP	-46.42	51.6	HHHHHHHHHHHCHHHHHHHHHHHHCCCCCCC
409	EEEKKKFLEQFNNEFEDQYYQQKGFGKGHQS	-46.42	51.6	HHHHHHHHHHHHHHHHHHHHHHHCCCCCCCC
712	EEEKKKFLEQFNNEFEDQYYQQQGFGKGHSN	-46.42	51.6	HHHHHHHHHHHHHHHHHHHHHHHCCCCCCCC
640	EEEKKKFLEQFNNEYEDKFYQQQGFGKGHSN	-46.42	54.8	HHHHHHHHHHHHHHHHHHHHHHHCCCCCCCC
919	EEEKKKFLEQFNNEYEDQFYQQKGFGKGHQN	-46.42	54.8	HHHHHHHHHHHHHHHHHHHHHHHCCCCCCCC
115	EEEKKKFLEQFNNEYEDQYYQQKGQGKGHQP	-46.42	51.6	HHHHHHHHHHHHHHHHHHHHHHHHCCCCCCC
789	EEEKKKFLEQLNNEKEDEEYQRYGFGKGHQS	-46.42	48.4	HHHHHHHHHHHHHHHHHHHHHHHCCCCCCCC
904	EEEKKKFLEQYNNEHEDERYQQQGFGKGHQS	-46.42	48.4	HHHHHHHHHHHHHHHHHHHHHHHCCCCCCCC
100	EEEKKKFLEQYNNEHEDQRYQQQGMGKGHQP	-46.42	48.4	HHHHHHHHHHHHHHHHHHHHHHHHCCCCCCC
811	EEEKKKFLEQYNNESEDERYQQQGFGKGHQN	-46.42	48.4	HHHHHHHHHHHHHCHHHHHHHHHCCCCCCCC
317	EEEKKKFLEQYNNESEDKRYQRYGFGKGHQS	-46.42	48.4	HHHHHHHHHHHHHHHHHHHHHHHCCCCCCCC
623	EEEKKKFLEQYNNESEDKRYQRYGMGKGHQP	-46.42	48.4	HHHHHHHHHHHHHHHHHHHHHHHHCCCCCCC
757	EEEKKKFLEQYNNESEDQRYQKEGFGKGHQS	-46.42	48.4	HHHHHHHHHHHHHHHHHHHHHHHHCCCCCCC
851	EEEKKKFLEQYNNESEDQRYQRYGFGKGHQS	-46.42	48.4	HHHHHHHHHHHHHCHHHHHHHHHCCCCCCCC
540	EEEKKKFLEQYNNEYEDEEYQRKGMGKGHQP	-46.42	48.4	HHHHHHHHHHHHHHHHHHHHHHHHCCCCCCC
235	EEEKKKFLEQYNNEYEDEEYQRKGRGKGHQS	-46.42	48.4	HHHHHHHHHHHHHHHHHHHHHHHHCCCCCCC
764	EEEKKKFLEQYNNEYEDEEYQRNGRGKGHSY	-46.42	48.4	HHHHHHHHHHHHHHHHHHHHHHCCCCCCCCC
551	EEEKKKFLEQYNNEYEDEQYQRKGFGKGHQS	-46.42	48.4	HHHHHHHHHHHHHHHHHHHHHHHCCCCCCCC
203	EEEKKKFLEQYNNEYEDQYYQQKGQGKGHQP	-46.42	48.4	HHHHHHHHHHHHHHHHHHHHHHHHCCCCCCC
405	EEEKRKFLEQFNNEEEDKRYQREGFGKGHQS	-46.42	48.4	HHHHHHHHHHHHHHHHHHHHHHHHCCCCCCC
016	EEEKRKFLEQFNNEEEDKRYQREGMGKGHQP	-46.42	48.4	HHHHHHHHHHHHHHHHHHHHHHHHCCCCCCC
798	EEEKRKFLEQFNNEYEDEEYQRKGRGKGHQS	-46.42	48.4	HHHHHHHHHHHHHHHHHHHHHHHHCCCCCCC
788	EEEKRKFLEQFNNEYEDEEYQRVGRGKGHSF	-46.42	48.4	HHHHHHHHHHHHHHHHHHHHHHHHCCCCCCC
923	EEEKRKFLEQFNNEYEDKRYQQEGMGKGHQP	-46.42	48.4	HHHHHHHHHHHHHHHHHHHHHHHHCCCCCCC
483	EEEKRKFLEQFNNEYEDKYYQKEGQGKGHSH	-46.42	48.4	HHHHHHHHHHHHHHHHHHHHHHHCCCCCCCC
036	EEEKRKFLEQFNNEYEDQFYQQKGFGKGHQS	-46.42	51.6	HHHHHHHHHHHHHHHHHHHHHHHCCCCCCCC
984	EEEKRKFLEQFNNEYEDQRYQQEGFGKGHQP	-46.42	48.4	HHHHHHHHHHHHHHHHHHHHHHHHCCCCCCC
412	EEEKRKFLEQFNNEYEDQRYQQEGFGKGHQS	-46.42	48.4	HHHHHHHHHHHHHHHHHHHHHHHHCCCCCCC
059	EEEKRKFLEQFNNEYEDQRYQQEGMGKGHQP	-46.42	48.4	HHHHHHHHHHHHHHHHHHHHHHHHCCCCCCC
611	EEEKRKFLEQFNNEYEDQYYQQKGFGKGHQS	-46.42	48.4	HHHHHHHHHHHHHHHHHHHHHHHCCCCCCCC
857	EEEKRKFLEQFNNEYEDQYYQQKGQGKGHSH	-46.42	48.4	HHHHHHHHHHHHHHHHHHHHHHHHCCCCCCC
500	EEEKRKFLEQLNNEEEDQRYQKEGFGKGHQS	-46.42	45.2	HHHHHHHHHHHHHHHHHHHHHHHHCCCCCCC
507	EEEKRKFLEQMKNEKEDEEYQRRGMGKGHQP	-46.42	41.9	HHHHHHHHHHHHHHHHHHHHHHHHHCCCCCC
971	EEEKRKFLEQQSNEEEDERYQQQGFGKGHQS	-46.42	41.9	HHHHHHHHHHHHHHHHHHHHHHHCCCCCCCC
084	EEEKRKFLEQYNNEAEDEQYQQRGMGKGHQP	-46.42	48.4	HHHHHHHHHHHHHHHHHHHHHHHHHCCCCCC
631	EEEKRKFLEQYNNEAEDQRYQQQGFGKGHSS	-46.42	48.4	HHHHHHHHHHHHHHHHHHHHHHHCCCCCCCC
027	EEEKRKFLEQYNNEAEDQRYQREGMGKGHQP	-46.42	48.4	HHHHHHHHHHHHHHHHHHHHHHHHHCCCCCC
423	EEEKRKFLEQYNNEAEDQRYQRQGFGKGHQS	-46.42	48.4	HHHHHHHHHHHHHHHHHHHHHHHHCCCCCCC
862	EEEKRKFLEQYNNEAEDQRYQRYGFGKGHSS	-46.42	48.4	HHHHHHHHHHHHHHHHHHHHHHHCCCCCCCC
424	EEEKRKFLEQYNNEEEDMKYQREGKGKGHSS	-46.42	45.2	HHHHHHHHHHHHHHHHHHHHHHHCCCCCCCC
322	EEEKRKFLEQYNNEHEDQRYQQQGMGKGHQP	-46.42	45.2	HHHHHHHHHHHHHHHHHHHHHHHHCCCCCCC
302	EEEKRKFLEQYNNEHEDQRYQREGFGKGHQS	-46.42	45.2	HHHHHHHHHHHHHHHHHHHHHHHHCCCCCCC
485	EEEKRKFLEQYNNESEDERYQQQGFGKGHQS	-46.42	45.2	HHHHHHHHHHHHHCHHHHHHHHHCCCCCCCC
924	EEEKRKFLEQYNNESEDERYQQQGMGKGHQP	-46.42	45.2	HHHHHHHHHHHHHCHHHHHHHHHHCCCCCCC
821	EEEKRKFLEQYNNESEDQRYQKEGFGKGHSS	-46.42	45.2	HHHHHHHHHHHHHHHHHHHHHHHHCCCCCCC
793	EEEKRKFLEQYNNESEDQRYQRYGFGKGHQS	-46.42	45.2	HHHHHHHHHHHHHCHHHHHHHHHCCCCCCCC
342	EEEKRKFLEQYNNESEDQRYQRYGMGKGHQP	-46.42	45.2	HHHHHHHHHHHHHCHHHHHHHHHHCCCCCCC
295	EEEKRKFLEQYNNESEDSRYQQQGMGKGHQP	-46.42	45.2	HHHHHHHHHHHHHHHHHHHHHHHHCCCCCCC
629	EEEKRKFLEQYNNEYEDEEYQRKGRGKGHQN	-46.42	45.2	HHHHHHHHHHHHHHHHHHHHHHHHCCCCCCC
336	EEEKRKFLEQYNNEYEDEEYQRKGRGKGHQP	-46.42	45.2	HHHHHHHHHHHHHHHHHHHHHHHHCCCCCCC
838	EEEKRKFLEQYNNEYEDEEYQRKGRGKGHQS	-46.42	45.2	HHHHHHHHHHHHHHHHHHHHHHHHCCCCCCC
963	EEEKRKFLEQYNNEYEDEEYQRNGRGKGHSY	-46.42	45.2	HHHHHHHHHHHHHHHHHHHHHHCCCCCCCCC
921	EEEKRKFLEQYNNEYEDQEYQRTGRGKGHSF	-46.42	45.2	HHHHHHHHHHHHHHHHHHHHHHCCCCCCCCC
230	EEEKRKFLEQYNNEYEDQQYQQQGMGKGHQP	-46.42	45.2	HHHHHHHHHHHHHHHHHHHHHHHHCCCCCCC
669	EEEKRKFLEQYNNEYEDQYYQQNGQGKGHSY	-46.42	45.2	HHHHHHHHHHHHHHHHHHHHHHHCCCCCCCC
183	EEEQRKFEEQRNNEEEDSRYQQQGWGKGHTP	-46.42	41.9	HHHHHHHHHHHHHHHHHHHHHHHCCCCCCCC
242	EEEQRKFLEQYNNEYEDEQYQQQGWGKGHNP	-46.42	45.2	CHHHHHHHHHHHHHHHHHHHHHHCCCCCCCC
063	EEERRKFLEQQNNEEEDERYQQQGFGKGHSN	-46.42	45.2	HHHHHHHHHHHHHHHHHHHHHHHCCCCCCCC
480	EQEKRKFLEQFNNEFEDQYYQQKGQGKGHSH	-46.42	45.2	HHHHHHHHHHHHHHHHHHHHHHHHCCCCCCC
907	EQEKRKFLEQYNNEHEDERYQQQGMGKGHQP	-46.42	41.9	HHHHHHHHHHHHHHHHHHHHHHHHCCCCCCC
740	EQEKRKFLEQYNNEYEDEEYQQTGRGKGHQF	-46.42	41.9	HHHHHHHHHHHHHHCHHHHHHHHHCCCCCCC
529	EQEKRKFLEQYNNEYEDEEYQRKGRGKGHQS	-46.42	41.9	HHHHHHHHHHHHHHHHHHHHHHHHCCCCCCC

854	EQEKRKFLEKMENEREDKEYQKQGFGKGNQN	-46.41	41.9	HHHHHHHHHHHHHHHHHHHHHHHHHCCCCCCC
331	EEEKRKILDQINNEEEQQRYQKEGFGQGHQS	-46.40	38.7	HHHHHHHHHHHCHHHHHHHHHHHHHCCCCCCC
545	EEEKRKLLDQLNQEKEQQRYQRYGFGQGHQS	-46.39	38.7	HHHHHHHHHHHHHHHHHHHHHHHHHCCCCCCC
078	EEEKRKFLQQFNQEYEDQFFQQKGFGKGKQN	-46.39	48.4	HHHHHHHHHHHHHHHHHHHHHHHHHCCCCCCC
487	EEEKRKFLEQMNNRQEDERYQREGKGKGHSH	-46.38	41.9	HHHHHHHHHHHHHHHHHHHHHHHHHCCCCCCC
330	EEQKRKMLEQKNNDEEDQRYQQQGWGKGHNP	-46.36	41.9	HHHHHHHHHHHCCHHHHHHHHHHHHCCCCCCC
703	EEEKRKMLEKIENEREDEEYQRKGRGKGHQS	-46.36	41.9	HHHHHHHHHHHHHHHHHHHHHHHHHHCCCCCC
727	EEEKKKFLEKYNNEYEDKFYQQKGFGKGHQS	-46.36	54.8	HHHHHHHHHHHHHHHHHHHHHHHHHCCCCCCC
609	EEEKKKLLEKINNEAEDRRYQQYGFGKGHQS	-46.36	51.6	HHHHHHHHHHHHHHHHHHHHHHHHHCCCCCCC
695	EEEKKKLLEKINNEAEDRRYQRQGFGKGHQN	-46.36	51.6	HHHHHHHHHHHHHHHHHHHHHHHHHHCCCCCC
553	EEEKRKLLEKINNEEEDRRYQQYGFGKGHSS	-46.36	45.2	HHHHHHHHHHHHHHHHHHHHHHHHHCCCCCCC
116	EEEKRKLLEKLNNEKEDRRYQREGQGKGHQS	-46.36	45.2	HHHHHHHHHHHHHHHHHHHHHHHHHHCCCCCC
134	EEEKRKLLQQINNEKEDEEYQRRGRGKGHQP	-46.35	41.9	HHHHHHHHHHHHHHHHHHHHHHHHHHCCCCCC
277	EEEKKKMLEQYNNESEDQRFQRYGFGKGKQN	-46.34	41.9	HHHHHHHHHHHHHHHHHHHHHHHHHCCCCCCC
596	EEEKRKMLEQINNEAEDKRFQRQGFGKGKQN	-46.34	41.9	HHHHHHHHHHHHHHHHHHHHHHHHHHCCCCCC
070	EEEKRKLLDKINNEEEQKRYQREGMGQGHQP	-46.33	41.9	HHHHHHHHHHHCHHHHHHHHHHHHHCCCCCCC
104	EEEKRKLLDKINNEEEQKRYQRYGFGQGHSN	-46.33	41.9	HHHHHHHHHHHCHHHHHHHHHHHHHCCCCCCC
543	EEEKKKMLEQFNQEYEDEEYQRKGRGKGHQS	-46.32	48.4	HHHHHHHHHHHHHHHHHHHHHHHHHHCCCCCC
949	EEEKKKMLEQINQEKEDEEYQRYGMGKGHQP	-46.32	45.2	HHHHHHHHHHHHHHHHHHHHHHHHHHHCCCCC
246	EEEKKKMLEQYNQESEDQRYQKEGFGKGHQS	-46.32	45.2	HHHHHHHHHHHHHHHHHHHHHHHHHHCCCCCC
030	EEEKRKMLEQYNQESEDQRYQQQGWGKGHNP	-46.32	41.9	HHHHHHHHHHHHHHHHHHHHHHHHHHCCCCCC
172	EQEKRKMLEQYNQEYEDEEYQRKGRGKGHQP	-46.32	38.7	HHHHHHHHHHHHHHHHHHHHHHHHHHCCCCCC
982	EEEKKKWLEQLNNRQEDERYQRYGFGKGHQS	-46.30	41.9	HHHHHHHHHHHHHHHHHHHHHHHHHCCCCCCC
777	EEERRKQLEQINQEEEDQRYQRYGFGKGHQS	-46.30	41.9	HHHHHHHHHHHHHHHHHHHHHHHHHCCCCCCC
422	EEERRKQLEQINQEKEDEEYQRYGFGKGHSS	-46.30	41.9	HHHHHHHHHHHHHHHHHHHHHHHHHCCCCCCC
206	EEEFRKWMEQLNNEAEDQRYQKEGQGKGHQS	-46.30	41.9	HHHHHHHHHHHHHHHHHHHHHHHHHHCCCCCC
264	EEEKKKWLEQLNNEKEDQRYQREGFGKGHSS	-46.29	45.2	HHHHHHHHHHHHHHHHHHHHHHHHHHCCCCCC
625	EEEKKKWLEQLNNEQEDMRYQRYGFGKGHQS	-46.29	45.2	HHHHHHHHHHHHHHHHHHHHHHHHHCCCCCCC
594	EEEKRKWLEQLSNEKEDERYQQQGFGKGHQS	-46.29	38.7	HHHHHHHHHHHHHHHHHHHHHHHHHCCCCCCC
723	EEEQKKQQEKYSNESEQKRYQQYGMGQGHQP	-46.28	35.5	HHHHHHHHHHHCHHHHHHHHHHHHHCCCCCCC
879	EEEQRKQQEKYNNEYEQKEYQRNGRGQGHSY	-46.28	35.5	HHHHHHHHHHHHHHHHHHHHHHHHHCCCCCCC
465	EEQKRKLLEQINNEQEDEEYQRKGRGKGHQS	-46.28	45.2	HHHHHHHHHHHHHHHHHHHHHHHHHHCCCCCC
801	EEERRKQLEQFNNEFEDKFFQQKGFGKGKSR	-46.28	48.4	HHHHHHHHHHHHHHHHHHHHHHHHHCCCCCCC
291	EEEKRKLLEQINNEAEDKRYQREGFGKGDSR	-46.25	51.6	HHHHHHHHHHHHHHHHHHHHHHHHHCCCCCCC
799	EEEKRKFLDKYNNESEQKRYQRYGFGQGHQS	-46.24	45.2	HHHHHHHHHHHHHHHHHHHHHHHHHCCCCCCC
217	EEEKRKMLDAINNRAEDQRYQRYGFGKGHQS	-46.23	45.2	HHHHHHHHHHHHHHHHHHHHHHHHHCCCCCCC
478	EEEKRKMLEQINNEKEHQEYQRRGQGRGHQS	-46.20	35.5	HHHHHHHHHHHHHHHHHHHHHHHHHHCCCCCC
338	EQEKRKFLEQYNNEYEDEYYQQKGQGKGHSH	-46.19	41.9	HHHHHHHHHHHHHHHHHHHHHHHHHCCCCCCC
639	EEEKRKMLQKFNNEYEDKYYQQKGFGKGHQS	-46.16	48.4	HHHHHHHHHHHHHHHHHHHHHHHHHCCCCCCC
035	EEERRKQLDKINNEAEDRRYQQYGFGKGNSN	-46.15	51.6	HHHHHHHHHHHHHHHHHHHHHHHHHCCCCCCC
701	EEEERKFQEKYNNEYEQEEYQRKGRGRGDSR	-46.14	45.2	CHHHHHHHHHHHHHHHHHHHHHHHHCCCCCCC
265	EEEKKKMLEQFNNEFEDRYYQQQGQGKGHSY	-46.14	48.4	HHHHHHHHHHHHHHHHHHHHHHHHHHCCCCCC
554	EEEKRKMLEQINNEQEDRYYQQYGQGKGHQP	-46.14	41.9	HHHHHHHHHHHHHHHHHHHHHHHHHHHCCCCC
024	EEEKRKMLEQYNNEYEDREYQRTGRGKGHQS	-46.14	41.9	HHHHHHHHHHHHHHHHHHHHHHHHCCCCCCCC
168	EEELKKILEKLNNEKEQKRYQRQGFGQGHQS	-46.11	41.9	HHHHHHHHHHHHHHHHHHHHHHHHHCCCCCCC
852	EEEQRKILEKLNNEKEQKRYQQYGMGQGHQP	-46.11	38.7	HHHHHHHHHHHHHHHHHHHHHHHHHHCCCCCC
056	EEEKRKLLEQINNEEEDQRYQRYGFGKGDSR	-46.09	48.4	HHHHHHHHHHHHHHHHHHHHHHHHHCCCCCCC
586	EEEKRKLLEQINNEKEDQEYQKQGFGKGDSR	-46.09	48.4	HHHHHHHHHHHHHHHHHHHHHHHHHCCCCCCC
690	EEEKKKLLDQINNEEEQQRYQQQGFGQGHQS	-46.09	41.9	HHHHHHHHHHHCHHHHHHHHHHHHHCCCCCCC
447	EEEKKKLLDQINNEYEQKYYQQKGQGQGHSH	-46.09	41.9	HHHHHHHHHHHHHHHHHHHHHHHHHHCCCCCC
819	EEEKRKLLDQINNEEEQQRYQKEGFGQGHQS	-46.09	38.7	HHHHHHHHHHHCHHHHHHHHHHHHHCCCCCCC
472	EEEKRKLLDQINNEKEQEEYQRYGFGQGHQS	-46.09	38.7	HHHHHHHHHHHHHHHHHHHHHHHHHCCCCCCC
386	EEEKRKLLDQINNEQEQKSYQRYGFGQGHQS	-46.09	38.7	HHHHHHHHHHHHHHHHHHHHHHHHHCCCCCCC
619	EEEKRKLLDQINNEYEQEEYQRKGRGQGHQS	-46.09	38.7	HHHHHHHHHHHHHHHHHHHHHHHHHHCCCCCC
929	EEEKRKFLEKYNQEYEDREYQRTGRGKGHQF	-46.09	48.4	HHHHHHHHHHHHHHHHHHHHHHHHCCCCCCCC
997	EEEKRKFLEKYNQEYEDREYQRTGRGKGHSF	-46.09	48.4	HHHHHHHHHHHHHHHHHHHHHHHHCCCCCCCC
556	EEERRKQLEQINQEAEDQRYQREGKGKGHSH	-46.06	45.2	HHHHHHHHHHHHHHHHHHHHHHHHHCCCCCCC
525	EEEKRKMLEKINNEKEDKRYQQQGFGKGDSR	-46.06	51.6	HHHHHHHHHHHHHHHHHHHHHHHHHCCCCCCC
996	EEEKRKLLEQINNEEEDKRYQQYGFGKGNQN	-46.05	41.9	HHHHHHHHHHHHHHHHHHHHHHHHHCCCCCCC
305	EEERRKQLDQYNNEEEHKRYQREGFGQGHSS	-46.05	38.7	HHHHHHHHHHHCCHHHHHHHHHHHHCCCCCCC
137	EEERRKQLEKLNNEKEDKRYQKEGFGKGDSR	-46.03	51.6	HHHHHHHHHHHHHHHHHHHHHHHHHCCCCCCC
883	EEELKKLLEKINNEEEHKEYQRTGRGQGHQF	-46.03	41.9	HHHHHHHHHHHCHHHHHHHHHHHHHCCCCCCC
009	EEERRKQLEKKNNEEEQKRYQRYGFGQGHQS	-46.03	38.7	HHHHHHHHHHHCHHHHHHHHHHHHHCCCCCCC
428	EEERRKQLEKYNNEAEQKRYQRYGFGQGHQS	-46.03	41.9	HHHHHHHHHHHHHHHHHHHHHHHHHCCCCCCC
089	EEEKKKMLEQFNNEEEDQRYQQEGFGKGHQS	-46.03	48.4	HHHHHHHHHHHCHHHHHHHHHHHHHCCCCCCC
421	EEEKKKMLEQFNNEFEDQYYQQQGQGKGHSY	-46.03	48.4	HHHHHHHHHHHHHHHHHHHHHHHHHHCCCCCC
748	EEEKKKMLEQFNNEKEDQRYQQQGMGKGHQP	-46.03	48.4	HHHHHHHHHHHHHHHHHHHHHHHHHHCCCCCC

532	EEEKKKMLEQFNNEYEDQYYQQKGFGKGHQN	-46.03	48.4	HHHHHHHHHHHHHHHHHHHHHHHCCCCCCCC
496	EEEKKKMLEQFNNEYEDQYYQQKGFGKGHQS	-46.03	48.4	HHHHHHHHHHHHHHHHHHHHHHHCCCCCCCC
396	EEEKKKMLEQINNEAEDKRYQREGKGKGHSA	-46.03	48.4	HHHHHHHHHHHHHHHHHHHHHHHCCCCCCCC
034	EEEKKKMLEQINNEAEDQRYQKEGMGKGHQP	-46.03	48.4	HHHHHHHHHHHHHHHHHHHHHHHHCCCCCCC
805	EEEKKKMLEQINNEEEDERYQQQGFGKGHQS	-46.03	45.2	HHHHHHHHHHHHHHHHHHHHHHHCCCCCCCC
818	EEEKKKMLEQINNEEEDERYQQQGMGKGHQP	-46.03	45.2	HHHHHHHHHHHHHHHHHHHHHHHHCCCCCCC
521	EEEKKKMLEQINNEEEDKKYQREGKGKGHSS	-46.03	45.2	HHHHHHHHHHHHHHHHHHHHHHHCCCCCCCC
092	EEEKKKMLEQINNEEEDKRYQQQGWGKGHNP	-46.03	45.2	HHHHHHHHHHHHHHHHHHHHHHHCCCCCCCC
125	EEEKKKMLEQINNEEEDKRYQQYGFGKGHQS	-46.03	45.2	HHHHHHHHHHHHHHHHHHHHHHHCCCCCCCC
367	EEEKKKMLEQINNEEEDKRYQREGFGKGHQS	-46.03	45.2	HHHHHHHHHHHHHHHHHHHHHHHHCCCCCCC
119	EEEKKKMLEQINNEEEDKRYQREGMGKGHQP	-46.03	45.2	HHHHHHHHHHHHHHHHHHHHHHHHCCCCCCC
402	EEEKKKMLEQINNEEEDKRYQRYGFGKGHQS	-46.03	45.2	HHHHHHHHHHHHHHHHHHHHHHHCCCCCCCC
662	EEEKKKMLEQINNEEEDQRYQRYGFGKGHQS	-46.03	45.2	HHHHHHHHHHHHHHHHHHHHHHHCCCCCCCC
647	EEEKKKMLEQINNEEEDQRYQRYGMGKGHQP	-46.03	45.2	HHHHHHHHHHHHHHHHHHHHHHHHCCCCCCC
714	EEEKKKMLEQINNEKEDEEYQQRGMGKGHQP	-46.03	45.2	HHHHHHHHHHHHHHHHHHHHHHHHCCCCCCC
835	EEEKKKMLEQINNEKEDEEYQRYGFGKGHQS	-46.03	45.2	HHHHHHHHHHHHHHHHHHHHHHHCCCCCCCC
742	EEEKKKMLEQYNNEAEDQRYQRYGMGKGHQP	-46.03	48.4	HHHHHHHHHHHHHHHHHHHHHHHHCCCCCCC
419	EEEKKKMLEQYNNEFEDQQYQRTGMGKGHQF	-46.03	45.2	HHHHHHHHHHHHHHHHHHHHHHHCCCCCCCC
899	EEEKKKMLEQYNNEHEDQRYQQQGFGKGHQS	-46.03	45.2	HHHHHHHHHHHHHHHHHHHHHHHCCCCCCCC
162	EEEKKKMLEQYNNESEDKRYQQQGFGKGHQS	-46.03	45.2	HHHHHHHHHHHHHHHHHHHHHHHHCCCCCCC
876	EEEKKKMLEQYNNEYEDEEYQRKGRGKGHQS	-46.03	45.2	HHHHHHHHHHHHHHHHHHHHHHHHCCCCCCC
503	EEEKKKMLEQYNNEYEDKYYQQKGFGKGHSS	-46.03	45.2	HHHHHHHHHHHHHHHHHHHHHHHCCCCCCCC
987	EEEKKKMLEQYNNEYEDQFYQQKGMGKGHQP	-46.03	48.4	HHHHHHHHHHHHHHHHHHHHHHHHCCCCCCC
684	EEEKRKMLEQINNEEEDERYQQQGFGKGHSS	-46.03	41.9	HHHHHHHHHHHHHHHHHHHHHHHCCCCCCCC
564	EEEKRKMLEQINNEEEDKRYQREGFGKGHQS	-46.03	41.9	HHHHHHHHHHHHHHHHHHHHHHHHCCCCCCC
045	EEEKRKMLEQINNEEEDKRYQREGMGKGHQP	-46.03	41.9	HHHHHHHHHHHHHHHHHHHHHHHHCCCCCCC
366	EEEKRKMLEQINNEEEDQRYQQYGFGKGHQS	-46.03	41.9	HHHHHHHHHHHHHHHHHHHHHHHCCCCCCCC
454	EEEKRKMLEQINNEEEDQRYQQYGMGKGHQP	-46.03	41.9	HHHHHHHHHHHHHHHHHHHHHHHHCCCCCCC
981	EEEKRKMLEQINNEEEDQRYQRYGFGKGHQS	-46.03	41.9	HHHHHHHHHHHHHHHHHHHHHHHCCCCCCCC
054	EEEKRKMLEQINNEEEDQRYQRYGFGKGHSS	-46.03	41.9	HHHHHHHHHHHHHHHHHHHHHHHCCCCCCCC
506	EEEKRKMLEQINNEKEDQEYQRSGQGKGHQS	-46.03	45.2	HHHHHHHHHHHHHHHHHHHHHHHCCCCCCCC
307	EEEKRKMLEQINNEQEDERYQQQGMGKGHQP	-46.03	41.9	HHHHHHHHHHHHHHHHHHHHHHHHCCCCCCC
720	EEEKRKMLEQINNEQEDERYQRQGWGKGHNP	-46.03	41.9	HHHHHHHHHHHHHHHHHHHHHHHCCCCCCCC
255	EEEKRKMLEQINNEQEDEYYQQYGQGKGHQP	-46.03	41.9	HHHHHHHHHHHHHHHHHHHHHHHHHCCCCCC
538	EEEKRKMLEQYNNEEEDMKYQREGMGKGHQP	-46.03	41.9	HHHHHHHHHHHHHHHHHHHHHHHHHCCCCCC
767	EEEKRKMLEQYNNEYEDKQYQKEGFGKGHQS	-46.03	41.9	HHHHHHHHHHHHHHHHHHHHHHHHCCCCCCC
940	EEEKRKMLEQYNNEYEDQEYQRNGRGKGHSY	-46.03	41.9	HHHHHHHHHHHHHHHHHHHHHHCCCCCCCCC
926	EEEQRKMLEQINNEAEDKRYQREGMGKGHQP	-46.03	45.2	HHHHHHHHHHHHHHHHHHHHHHHHHCCCCCC
655	EQEKRKMLEQFNNEYEDKYYQQKGFGKGHQS	-46.03	41.9	HHHHHHHHHHHHHHHHHHHHHHHCCCCCCCC
328	EQEKRKMLEQINNEEEDKRYQRQGFGKGHQS	-46.03	38.7	HHHHHHHHHHHHHHHHHHHHHHHHCCCCCCC
897	EEERRKQLEKYNNESEQERYQQQGMGQGHQP	-46.02	38.7	HHHHHHHHHHHHHHHHHHHHHHHHCCCCCCC
816	EEEKRKFLDKFNNEYEQKYYQQKGFGQGHSN	-46.01	48.4	HHHHHHHHHHHHHHHHHHHHHHHCCCCCCCC
573	EEEKKKFLEKYEDEYRDEEYQRKGRGKGHQS	-46.01	45.2	HHHHHHHHHHHHHHHHHHHHHHHHCCCCCCC
151	EEERKKQLEQINNEEEDKRYQREGFGKGHQS	-46.00	45.2	HHHHHHHHHHHHHHHHHHHHHHHHCCCCCCC
262	EEERKKQLEQINNEEEDKRYQREGVGKGHNP	-46.00	45.2	HHHHHHHHHHHHHHHHHHHHHHHHCCCCCCC
618	EEERKKQLEQINNEEEDQRYQREGVGKGHQP	-46.00	45.2	HHHHHHHHHHHHHHHHHHHHHHHHCCCCCCC
668	EEERKKQLEQYNNEHEDERYQQQGFGKGHSS	-46.00	45.2	HHHHHHHHHHHHHHHHHHHHHHHCCCCCCCC
761	EEERRKQLEQFNNEYEDEEYQRQGRGKGHQP	-46.00	45.2	HHHHHHHHHHHHHHHHHHHHHHHHCCCCCCC
773	EEERRKQLEQINNEAEDERYQQQGFGKGHQS	-46.00	45.2	HHHHHHHHHHHHHHHHHHHHHHHCCCCCCCC
490	EEERRKQLEQINNEAEDERYQQQGMGKGHQP	-46.00	45.2	HHHHHHHHHHHHHHHHHHHHHHHHCCCCCCC
197	EEERRKQLEQINNEAEDKRYQREGMGKGHQP	-46.00	45.2	HHHHHHHHHHHHHHHHHHHHHHHHHCCCCCC
772	EEERRKQLEQINNEAEDKRYQREGQGKGHQS	-46.00	45.2	HHHHHHHHHHHHHHHHHHHHHHHHCCCCCCC
018	EEERRKQLEQINNEAEDKRYQRQGFGKGHQS	-46.00	45.2	HHHHHHHHHHHHHHHHHHHHHHHHCCCCCCC
199	EEERRKQLEQINNEAEDQRYQRQGFGKGHSS	-46.00	45.2	HHHHHHHHHHHHHHHHHHHHHHHCCCCCCCC
585	EEERRKQLEQINNEAEDQRYQRYGMGKGHQP	-46.00	45.2	HHHHHHHHHHHHHHHHHHHHHHHHCCCCCCC
559	EEERRKQLEQINNEEEDERYQQQGQGKGHQS	-46.00	41.9	HHHHHHHHHHHHHHHHHHHHHHHHCCCCCCC
646	EEERRKQLEQINNEEEDKEYQRVGRGKGHQS	-46.00	41.9	HHHHHHHHHHHHHHHHHHHHHHHHCCCCCCC
112	EEERRKQLEQINNEKEDEEYQRRGMGKGHQP	-46.00	41.9	HHHHHHHHHHHHHHHHHHHHHHHHHCCCCCC
185	EEERRKQLEQINNEKEDEEYQRYGFGKGHQS	-46.00	41.9	HHHHHHHHHHHHHHHHHHHHHHHCCCCCCCC
663	EEERRKQLEQINNEQEDEKYQQEGMGKGHQP	-46.00	41.9	HHHHHHHHHHHHHHHHHHHHHHHHCCCCCCC
681	EEERRKQLEQISNEKEDQEYQKQGRGKGHQP	-46.00	38.7	HHHHHHHHHHHHHHHHHHHHHHHHCCCCCCC
133	EQERRKQLEQYNNEYEDQYYQQKGQGKGHSH	-46.00	38.7	HHHHHHHHHHHHHHHHHHHHHHHHCCCCCCC
175	EEEKRKFLEKFNNEYEHKYKQQKGFGSGKQN	-45.99	41.9	HHHHHHHHHHHHHHHHHHHHHHHCCCCCCCC
535	EEERRKQLEQYNNRSEDERYQQQGMGKGHQP	-45.98	38.7	HHHHHHHHHHHHHCHHHHHHHHHHCCCCCCC
652	EEEQRKFLEKYNNESEDKRYQQQGFGKGDSR	-45.94	54.8	CHHHHHHHHHHHHHHHHHHHHHHCCCCCCCC
708	EEEKRKFLDQYRHEYEQEFYQQKGFGQGHSS	-45.94	45.2	HHHHHHHHHHHHHHHHHHHHHHHCCCCCCCC
169	EEEKRKLLEQINNEAEDQRYQQQGFGKGDSR	-45.93	51.6	HHHHHHHHHHHHHHHHHHHHHHHCCCCCCCC

042	EEEKRKQLEQYNQEYEQEEYQRVGRGQGHQF	-45.92	35.5	HHHHHHHHHHHHHHHHHHHHHHHHHCCCCCC
970	EEEKRKLLDQINQEAEQKRYQREGMGQGHQP	-45.92	41.9	HHHHHHHHHHHHHHHHHHHHHHHHHCCCCCC
730	EQEKRHILEQYNQEYEDEEYQRKGRGKGHQP	-45.92	38.7	HHHHHHHHHHHHHHHHHHHHHHHHCCCCCCC
364	EEEERKFQEQYNNEYEDEEYQRKGRGKGHQP	-45.90	41.9	CHHHHHHHHHHHHHHHHHHHHHHHCCCCCCC
842	EEEKRKFLEQFNNEYEDQFYQKEGFGKGHQS	-45.90	51.6	HHHHHHHHHHHHHHHHHHHHHHHHCCCCCCC
420	EEEKKKLLDKLNQEKEQKRYQRYGFGQGDSR	-45.90	51.6	HHHHHHHHHHHHHHHHHHHHHHHCCCCCCCC
698	EEEKRKLLEQIQKDREDREYQREGMGKGHQP	-45.90	35.5	HHHHHHHHHHHHHHHHHHHHHHHHCCCCCCC
650	EEEKKKMLEKINNEAEDKRYQREGFGKGNQN	-45.86	51.6	HHHHHHHHHHHHHHHHHHHHHHHHCCCCCCC
806	EEEKKKMLDKINNEAEQKRYQRYGMGQGHQP	-45.85	48.4	HHHHHHHHHHHHHHHHHHHHHHHHCCCCCCC
823	EEEKKKMLEKFNQEYEDKYYQQKGFGKGDSR	-45.85	58.1	HHHHHHHHHHHHHHHHHHHHHHHCCCCCCCC
654	EEERRKQLEKINNEQEDKSYQRHGFGKGNSN	-45.83	45.2	HHHHHHHHHHHHHHHHHHHHHHCCCCCCCCC
229	EEEKKKFLEQYNNESEDQRYQRYGFGKGHQN	-45.82	48.4	HHHHHHHHHHHHHCHHHHHHHHHCCCCCCCC
978	EEEWKKLLDQIKNEKEQEEYQRRGLGQGHQP	-45.82	41.9	HHHHHHHHHHHHHHHHHHHHHHHHHCCCCCC
278	EEERRKQLEKINQEEEDKRYQREGFGKGDSR	-45.82	51.6	HHHHHHHHHHHHHHHHHHHHHHHHCCCCCCC
508	EEEKRKMLEQINNEEEDKRYQREGKGKGHSH	-45.80	41.9	HHHHHHHHHHHHHHHHHHHHHHHCCCCCCCC
012	EEEKRKMLEQYNNEYEDQQYQKEGFGKGHQS	-45.79	41.9	HHHHHHHHHHHHHHHHHHHHHHHHCCCCCCC
263	EEEKRKFLDKKNNEEEQKRYQQYGKGQGHSH	-45.77	45.2	HHHHHHHHHHCCHHHHHHHHHHHHCCCCCCC
106	EEEKRKFLDKYNNEAEQKRYQRQGFGQGHQS	-45.77	48.4	HHHHHHHHHHHHHHHHHHHHHHHCCCCCCCC
985	EEEQRKQLEQFKNEYEQEFYQQQGFGQGHQS	-45.76	38.7	HHHHHHHHHHHHHHHHHHHHHHHCCCCCCCC
215	EEEKRKLLEQINNEAEDKRYQRYGFGKGDSR	-45.74	51.6	HHHHHHHHHHHHHHHHHHHHHHHCCCCCCCC
505	EEEKRKLLEQINNEEEDKRYQREGFGKGDSR	-45.74	48.4	HHHHHHHHHHHHHHHHHHHHHHHHCCCCCCC
470	EEEKRKLLDQINNEYEQREYQRKGFGQGHQS	-45.72	38.7	HHHHHHHHHHHHHHHHHHHHHHHHCCCCCCC
693	EEEEKKRQEQYNNEYEDQEYQRTGRGKGHQF	-45.72	41.9	HHHHHHHHHHHHHHHHHHHHHHCCCCCCCCC
174	EEEKRKLLDKINQEAEQRRYQREGMGQGHQP	-45.71	45.2	HHHHHHHHHHHHHHHHHHHHHHHHCCCCCCC
021	EEEKRKMLEQINNEAEDQRYQQQGMGKGHQP	-45.70	45.2	HHHHHHHHHHHHHHHHHHHHHHHHCCCCCCC
149	EEEKRKFLEQFNNEEEDKRYQREGFGKGDSR	-45.69	54.8	HHHHHHHHHHHHHHHHHHHHHHHHCCCCCCC
775	EEEKRKFLEQYNNEYEDEFYQQKGFGKGDSR	-45.69	54.8	HHHHHHHHHHHHHHHHHHHHHHHCCCCCCCC
885	EEEKKKMLEQIENEREDEEYQRKGMGKGHQP	-45.67	41.9	HHHHHHHHHHHHHHHHHHHHHHHHCCCCCCC
418	EEEKRKILEQINQEKEQEEFQRRGMGQGKQP	-45.65	32.3	HHHHHHHHHHHHHHHHHHHHHHHHCCCCCCC
728	EEEKRKMLDQYNNEYEDEEYQRKGRGKGHQS	-45.64	45.2	HHHHHHHHHHHHHHHHHHHHHHHHCCCCCCC
837	EEERKKQLEQKNNEEEQERYQQQGFGQGHQS	-45.63	38.7	HHHHHHHHHHHCHHHHHHHHHHHCCCCCCCC
359	EEERKKQLEQYNNESEQERYQQQGWGQGHNP	-45.63	38.7	HHHHHHHHHHHHHHHHHHHHHHHCCCCCCCC
192	EEERRKQLEQYNNEAEQQRYQKEGFGQGHQS	-45.63	38.7	HHHHHHHHHHHHHHHHHHHHHHHCCCCCCCC
131	EEERRKQLEQYNNEAEQQRYQRQGFGQGHQS	-45.63	38.7	HHHHHHHHHHHHHHHHHHHHHHHCCCCCCCC
015	EEEWKKQMEQYNNEYEQEEYQRKGRGQGHQS	-45.63	35.5	HHHHHHHHHHHHHHHHHHHHHHHHCCCCCCC
026	EEEKKKLLDQINNEEEQQRYQREGKGQGHSH	-45.62	41.9	HHHHHHHHHHHCHHHHHHHHHHHHCCCCCCC
086	EEEKRKLLDQINNEKEQEEYQRRGKGQGHSH	-45.62	38.7	HHHHHHHHHHHHHHHHHHHHHHHHHCCCCCC
928	EEQRRKQLEQINQEQEDKSYQRHGFGKGDSR	-45.60	51.6	HHHHHHHHHHHHHHHHHHHHHHHCCCCCCCC
943	EEEKKKMLEKINNEEEHKRKQREGFGSGKQN	-45.60	38.7	HHHHHHHHHHHHHHHHHHHHHHHHHCCCCCC
339	EEQKKKMLEQYNNEYEDQYYQQKGQGKGHSH	-45.59	48.4	HHHHHHHHHHHHHHHHHHHHHHHHCCCCCCC
007	EEERRKQLEKYNNEAEQRRFQRYGFGQGKSN	-45.56	38.7	HHHHHHHHHHHHHHHHHHHHHHHCCCCCCCC
697	EEQKKKFLEQFNQEYEDEEYQRKGRGKGDSR	-45.54	61.3	HHHHHHHHHHHHHHHHHHHHHHHCCCCCCCC
351	EEEKKKFLDQYNNEAEQQRYQQQGFGQGHQS	-45.52	48.4	HHHHHHHHHHHHHHHHHHHHHHHCCCCCCCC
232	EEEKKKFLDQYNNEEEQMRYQREGFGQGHQS	-45.52	45.2	HHHHHHHHHHHCCHHHHHHHHHHHHCCCCCC
844	EEEKKKFLDQYNNESEQQRYQRYGMGQGHQP	-45.52	45.2	HHHHHHHHHHHHCHHHHHHHHHHHCCCCCCC
850	EEEKRKFLDQYNNEAEQQRYQRYGFGQGHQN	-45.52	45.2	HHHHHHHHHHHHHHHHHHHHHHHCCCCCCCC
118	EEEKRKFLDQYNNEAEQQRYQRYGFGQGHQS	-45.52	45.2	HHHHHHHHHHHHHHHHHHHHHHHCCCCCCCC
560	EEEKRKFLDQYNNEHEQQRYQQQGFGQGHQS	-45.52	41.9	HHHHHHHHHHHHHHHHHHHHHHHCCCCCCCC
917	EEEKRKFLDQYNNEHEQQRYQQQGMGQGHQP	-45.52	41.9	HHHHHHHHHHHHHHHHHHHHHHHHCCCCCCC
517	EEEKRKFLDQYNNEYEQQYYQQKGFGQGHQS	-45.52	41.9	HHHHHHHHHHHHHHHHHHHHHHHCCCCCCCC
389	EEEKKKMLEQINNEKEDQEYQRGGFGKGHQP	-45.51	45.2	HHHHHHHHHHHHHHHHHHHHHHCCCCCCCCC
333	EEEKRKFLDQFNNRYEQEFYQQQGKGQGHSH	-45.50	45.2	HHHHHHHHHHHHHHHHHHHHHHHHCCCCCCC
069	EEEWKKFIEQYNNEYEDEEYQRKGRGKGNSN	-45.49	45.2	HHHHHHHHHHHHHHHHHHHHHHHCCCCCCCC
898	EEEKRKFLEQYNNEYEDEEYQRKGRGKGNQN	-45.48	45.2	HHHHHHHHHHHHHHHHHHHHHHHHCCCCCCC
872	EEEKRKFLEQYNQEAEDKRYQRYGFGKGDSR	-45.47	54.8	HHHHHHHHHHHHHHHHHHHHHHHCCCCCCCC
597	EEEKRKFLEQKNNREEDERYQQYGFGKGNSN	-45.46	41.9	HHHHHHHHHHHHHHHHHHHHHHHCCCCCCCC
942	EEEKRKQLEQYNNEYEQEEYQRKGRGQGHQS	-45.43	35.5	HHHHHHHHHHHHHHHHHHHHHHHHCCCCCCC
072	EEEKRKLLEQLNNEKEDMRYQRYGFGKGDSR	-45.42	48.4	HHHHHHHHHHHHHHHHHHHHHHCCCCCCCCC
497	EEEKKKMLEKINNEAEDRRYQKEGFGKGHQS	-45.40	51.6	HHHHHHHHHHHHHHHHHHHHHHHHCCCCCCC
273	EEEKRKMLEKINNEAEDRQYQRNGSGKGHSY	-45.40	48.4	HHHHHHHHHHHHHHHHHHHHHHCCCCCCCCC
184	EEEKRKMLEKINNEAEDRRYQQQGMGKGHQP	-45.40	48.4	HHHHHHHHHHHHHHHHHHHHHHHHCCCCCCC
223	EEEWKKILEQLNQEKEQQRYQREGMGQGHQP	-45.40	38.7	HHHHHHHHHHHHHHHHHHHHHHHHHCCCCCC
840	EEEKRKMLQQINNEEEDSRYQQQGFGKGHQS	-45.39	41.9	HHHHHHHHHHHHHHHHHHHHHHHCCCCCCCC
886	EEEKRKMLQQINNEKEDEEYQRYGMGKGHQP	-45.39	41.9	HHHHHHHHHHHHHHHHHHHHHHHHHCCCCCC
676	EEEKRKFLEQYNNEYEHEEYQRKGFGRGDSR	-45.39	45.2	HHHHHHHHHHHHHHHHHHHHHHHCCCCCCCC
324	EEERRKQLEKYNNEEEQMRYQREGFGQGDSR	-45.39	45.2	HHHHHHHHHHHCCHHHHHHHHHHHCCCCCCC
349	EEEKRKMLEQINQEAEDERYQQQGFGKGNQN	-45.39	45.2	HHHHHHHHHHHHHHHHHHHHHHHCCCCCCCC

463	EEEKRKLLEKINQEAEQKQYQRSGKGQGHSS	-45.39	45.2	HHHHHHHHHHHHHHHHHHHHHHHHHCCCCCCCC
237	EEEKRKLLEKINQEEEQKRYQRYGFGQGHQS	-45.39	38.7	HHHHHHHHHHHHHHHHHHHHHHHHHCCCCCCCC
294	EEERRKQLEKINNEKEDKEYQKQGFGKGDSR	-45.38	51.6	HHHHHHHHHHHHHHHHHHHHHHHHHCCCCCCCC
202	EEERRKQLEKLNNEKEDRRYQRYGFGKGHQN	-45.38	45.2	HHHHHHHHHHHHHHHHHHHHHHHHHCCCCCCCC
888	EEEKKKMLDKYNNEEEQKRYQRQGFGQGHSS	-45.37	45.2	HHHHHHHHHHHHCCHHHHHHHHHHHCCCCCCCC
491	EEEKRKLLDQINNEAEQKRYQRQGFGQGDSR	-45.36	48.4	HHHHHHHHHHHHHHHHHHHHHHHHHCCCCCCCC
692	EEEKRKWLEKLNNEKEDRRYQRYGFGKGHQS	-45.34	45.2	HHHHHHHHHHHHHHHHHHHHHHHHHCCCCCCCC
453	EEERRKQLEQFNQEYEQKFYQQKGMGQGHQP	-45.31	41.9	HHHHHHHHHHHHHHHHHHHHHHHHHHCCCCCCC
522	EEEKRKLLEKINNEKEHKEYQKQGFGQGDSR	-45.30	45.2	HHHHHHHHHHHHHHHHHHHHHHHHHCCCCCCCC
925	EEEKKKMLEQINNEAEDKRYQREGFGKGDSR	-45.29	54.8	HHHHHHHHHHHHHHHHHHHHHHHHHHCCCCCCC
052	EEEKRKMLEQINNEEEDQRYQQQGFGKGDSR	-45.29	48.4	HHHHHHHHHHHHHHHHHHHHHHHHHCCCCCCCC
599	EEEQRKMLEQINNEKEDEEYQRYGFGKGDSR	-45.29	48.4	HHHHHHHHHHHHHHHHHHHHHHHHHCCCCCCCC
095	EQEKRKMLEQINNEKEDEEYQRYGFGKGDSR	-45.29	45.2	HHHHHHHHHHHHHHHHHHHHHHHHHCCCCCCCC
003	EEEKRKLLEKINNEEEQKRYQQYGFGQGHQS	-45.28	38.7	HHHHHHHHHHHHCHHHHHHHHHHHHCCCCCCCC
741	EEEKRKYLEQYNNEYEDEQYQQQGFGKGDSR	-45.28	48.4	HHHHHHHHHHHHHHHHHHHHHHHHHCCCCCCCC
004	EEERRKQLEQINNEEEDERYQQQGFGKGDSR	-45.27	48.4	HHHHHHHHHHHHHHHHHHHHHHHHHCCCCCCCC
632	EEEKKKFLEQYEDEYRDQEYQRNGRGKGHSY	-45.24	41.9	HHHHHHHHHHHHHHHHHHHHHHHHCCCCCCCCC
261	EEEKRKFLEQYEDEYRDEEYQRKGFGKGHQS	-45.24	38.7	HHHHHHHHHHHHHHHHHHHHHHHHHCCCCCCCC
848	EEEKRKFLEQYNNESEHQRKQRYGFGSGKQN	-45.22	35.5	HHHHHHHHHHHHHHHHHHHHHHHHHCCCCCCCC
077	EEERRKQLDQINNEEEQRRYQREGFGQGHQS	-45.22	38.7	HHHHHHHHHHHHCHHHHHHHHHHHHHHCCCCCC
624	EEEKRKFLEQYNNEEEDMRYQREGFGKGDSR	-45.18	51.6	HHHHHHHHHHHHCHHHHHHHHHHHHHCCCCCCC
479	EEEWKKFIEQYNNEYEDEEYQRKGFGKGDSR	-45.18	51.6	HHHHHHHHHHHHHHHHHHHHHHHHHCCCCCCCC
210	EQEKRKFLEQYNNRHEDERYQQQGFGKGDSR	-45.15	45.2	HHHHHHHHHHHHHHHHHHHHHHHHHCCCCCCCC
849	EEEKKKFLEAYNNRYEDEEYQRNGRGKGHSY	-45.15	45.2	HHHHHHHHHHHHHHHHHHHHHHHHCCCCCCCCC
208	EEEKRKFLEQYNNRYEDEEYQRTGRGKGDSR	-45.14	48.4	HHHHHHHHHHHHHHHHHHHHHHHHCCCCCCCCC
718	EEEKKKMLDQINNEAEQKRYQQYGFGQGHQS	-45.13	45.2	HHHHHHHHHHHHHHHHHHHHHHHHHCCCCCCCC
097	EEEKKKMLDQINNEEEQKRYQRYGFGQGHSS	-45.13	41.9	HHHHHHHHHHHHCHHHHHHHHHHHHCCCCCCCC
040	EEEKRKMLDQYNNEEEQMRYQREGFGQGHQS	-45.13	38.7	HHHHHHHHHHHHCCHHHHHHHHHHHHHCCCCCC
518	EEEKRKLLEKINNEEEDRRYQQQGFGKGDSR	-45.11	51.6	HHHHHHHHHHHHHHHHHHHHHHHHHCCCCCCCC
977	EEERRKQLDQINNEKEQEEYQRYGFGQGHSS	-45.11	38.7	HHHHHHHHHHHHHHHHHHHHHHHHHCCCCCCCC
082	EEEKKKILEQINNEAEQKRYQREGMGQGHQP	-45.11	41.9	HHHHHHHHHHHHHHHHHHHHHHHHHHHCCCCCC
827	EEEKRKILEQINNEKEQEEYQRRGMGQGHQP	-45.11	35.5	HHHHHHHHHHHHHHHHHHHHHHHHHHHCCCCCC
245	EEEKRKILEQINNEKEQQEYQRGGFGQGHQP	-45.11	35.5	HHHHHHHHHHHHHHHHHHHHHHHHCCCCCCCCC
983	EEEKRKILEQLNNEKEQQRYQRYGFGQGHQS	-45.11	35.5	HHHHHHHHHHHHHHHHHHHHHHHHHCCCCCCCC
961	EQEERKIQEQFNNEYEQEFYQQQGFGQGHQS	-45.11	35.5	HHHHHHHHHHHHHHHHHHHHHHHHHCCCCCCCC
778	EEEKRKFLDDYNNRSEDQRYQRYGFGKGHQS	-45.10	45.2	HHHHHHHHHHHHHCCCHHHHHHHHHCCCCCCCC
950	EEEKKKMLEQINNEAEDKRYQRYGFGKGNQN	-45.09	48.4	HHHHHHHHHHHHHHHHHHHHHHHHHCCCCCCCC
043	EEEKRKMLEQINNEAEDERYQRKGFGKGNQN	-45.09	45.2	HHHHHHHHHHHHHHHHHHHHHHHHHCCCCCCCC
829	EEEKRKMLEQYNNEYEDEEYQRKGRGKGNQN	-45.09	41.9	HHHHHHHHHHHHHHHHHHHHHHHHHHCCCCCCC
239	EEEERKLQEKLNNEKEQKRYQRYGFGQGHQN	-45.09	35.5	HHHHHHHHHHHHHHHHHHHHHHHHHCCCCCCCC
715	EEEERKLQEKLNNEKEQKRYQRYGQGQGHQS	-45.09	35.5	HHHHHHHHHHHHHHHHHHHHHHHHHHHCCCCCC
988	EEEKKKLLEKINNEEEQKRYQRYGFGQGHSS	-45.09	41.9	HHHHHHHHHHHHCHHHHHHHHHHHHCCCCCCCC
411	EEEKRKLLEKINNEAEQKRYQRQGFGQGHQS	-45.09	41.9	HHHHHHHHHHHHHHHHHHHHHHHHHCCCCCCCC
947	EEEKRKLLEKINNEEEQKRYQREGFGQGHQS	-45.09	38.7	HHHHHHHHHHHHCHHHHHHHHHHHHHCCCCCCC
915	EEEKRKLLEKLNNEKEQKRYQKEGWGQGHNP	-45.09	38.7	HHHHHHHHHHHHHHHHHHHHHHHHHHCCCCCCC
583	EQEKRKLLEKLNNEKEQKRYQREGMGQGHQP	-45.09	35.5	HHHHHHHHHHHHHHHHHHHHHHHHHHHCCCCCC
064	EEEKKKFLDQFNNEFEQQYYQQQGFGQGHSN	-45.05	48.4	HHHHHHHHHHHHHHHHHHHHHHHHHCCCCCCCC
713	EEEKRKFLDQYNNEYEQEEYQRTGRGQGHQF	-45.05	41.9	HHHHHHHHHHHHHHHHHHHHHHHHHHCCCCCCC
833	EEERRKQLEQYNNEAEQERYQQQGMGQGHQP	-45.02	38.7	HHHHHHHHHHHHHHHHHHHHHHHHHHCCCCCCC
067	EQEKRHFLEKYNNEAEDKQYQQRGKGKGHSA	-44.95	48.4	HHHHHHHHHHHHHHHHHHHHHHHHHHCCCCCCC
511	EEEKRKWLDQLNNEKEQEEYQRYGFGQGHSS	-44.92	38.7	HHHHHHHHHHHHHHHHHHHHHHHHHCCCCCCCC
385	EEEKRKMLDQINNEKEQEEYQRRGMGQGHQP	-44.90	38.7	HHHHHHHHHHHHHHHHHHHHHHHHHHHCCCCCC
756	EEERRKQLDKYNQEYEQEEYQRKGRGQGDSR	-44.89	48.4	HHHHHHHHHHHHHHHHHHHHHHHHHCCCCCCCC
941	EEEKRKLLEKINNEEEQKRYQREGFGQGHSS	-44.86	38.7	HHHHHHHHHHHHCHHHHHHHHHHHHHCCCCCCC
843	EEEKRKFLEKYNNEYEDREYQRTGRGKGNQN	-44.86	48.4	HHHHHHHHHHHHHHHHHHHHHHHHCCCCCCCCC
598	EEEKRKFLEKYNNEYEQKEFQKEGFGQGKQN	-44.85	38.7	HHHHHHHHHHHHHHHHHHHHHHHHHCCCCCCCC
442	EEEKKKMLEQINNEKEHEEKQRYGFGSGKQN	-44.83	35.5	HHHHHHHHHHHHHHHHHHHHHHHHHCCCCCCCC
111	EEEKRKMLEQINNEEEHKRKQREGFGSGKQN	-44.83	32.3	HHHHHHHHHHHHHHHHHHHHHHHHHHHCCCCCC
687	EEEKKKFLEKFNQEKEQQRYQQQGFGQGHQS	-44.82	48.4	HHHHHHHHHHHHHHHHHHHHHHHHHCCCCCCCC
979	EEEKRKFLEKYNQEYEQKEYQKEGRGQGHQS	-44.82	41.9	HHHHHHHHHHHHHHHHHHHHHHHHHHCCCCCCC
431	EQEKRKLLEQIENEREQQEYQQKGFGQGTQN	-44.82	29.0	HHHHHHHHHHHHHHHHHHHHHHHHHHHCCCCCC
516	EEEKKKLLEQIEQRREQEEYQQKGQGQGHQS	-44.79	32.3	HHHHHHHHHHHHHHHHHHHHHHHHHHHCCCCCC
347	EEEKKKMLEQINNEEEDQRYQQYGFGKGDSR	-44.78	51.6	HHHHHHHHHHHHHHHHHHHHHHHHHCCCCCCCC
256	EEEKRKMLEQINNEAEDQRYQRYGFGKGDSR	-44.78	51.6	HHHHHHHHHHHHHHHHHHHHHHHHHCCCCCCCC
722	EEEKRKFLDDYNNRSEDERYQRYGMGKGHQP	-44.78	45.2	HHHHHHHHHHHHHCCCHHHHHHHHHCCCCCCCC
645	EEEKKKMLQKINNEAEDRRYQREGKGKGHSH	-44.76	51.6	HHHHHHHHHHHHHHHHHHHHHHHHHCCCCCCCC
314	EEEKKKMLEQINNEAEHKRKQREGQGSGKQS	-44.76	38.7	HHHHHHHHHHHHHHHHHHHHHHHHHCCCCCCCC

910	EEERRKQLEQINNEAEDKRYQRYGFGKGDSR	-44.76	51.6	HHHHHHHHHHHHHHHHHHHHHHHHCCCCCCCC
155	EEERRKQLQKINNEAEDRRYQRYGFGKGHQN	-44.74	48.4	HHHHHHHHHHHHHHHHHHHHHHHHCCCCCCCC
531	EEQKRKFLEQFNNEYEDQFYQQKGFGKGDSR	-44.74	61.3	HHHHHHHHHHHHHHHHHHHHHHHHCCCCCCCC
610	EEERRKQLEQYNNEEEQMRYQREGFGQGHQS	-44.73	35.5	HHHHHHHHHHHCCHHHHHHHHHHHHHCCCCCC
313	EEQLKKILEQINNEAEQKRYQREGKGQGHSH	-44.67	45.2	HHHHHHHHHHHHHHHHHHHHHHHHHCCCCCCC
417	EEEKKKLLEQINNEKEQEEFQRYGFGQGKQN	-44.65	35.5	HHHHHHHHHHHHHHHHHHHHHHHHCCCCCCCC
796	EEELKKILEQLNNEKEQKRYQRYGMGQGHQP	-44.63	38.7	HHHHHHHHHHHHHHHHHHHHHHHHHCCCCCCC
033	EEEKKKLLEQINQEQEQEEYQRKGRGQGHQP	-44.62	38.7	HHHHHHHHHHHHHHHHHHHHHHHHHCCCCCCC
088	EEEKRKLLEQINQEEEQQRYQQQGMGQGHQP	-44.62	35.5	HHHHHHHHHHHHHHHHHHHHHHHHHCCCCCCC
797	EEEKKKLLEKINNEAEQKRYQREGKGQGHSS	-44.62	45.2	HHHHHHHHHHHHHHHHHHHHHHHHCCCCCCCC
383	EEEKRKLLEKINNEKEQQRYQQQGMGQGHQP	-44.62	38.7	HHHHHHHHHHHHHHHHHHHHHHHHHCCCCCCC
103	EEEWRKLMEKYNNEEEQKRYQREGMGQGHQP	-44.62	35.5	HHHHHHHHHHHCCHHHHHHHHHHHHCCCCCCC
673	EEEKRKLLEQINNEAEQKRFQREGKGQGKSH	-44.58	35.5	HHHHHHHHHHHHHHHHHHHHHHHHHCCCCCCC
309	EEEQRRLYEQLNNEKEDQRYQRYGFGKGHSS	-44.58	38.7	HHHHHHHHHHHHHHHHHHHHHHHHCCCCCCCC
754	EEEKRKMLDQINNEKEQEEYQRYGFGQGHQS	-44.54	38.7	HHHHHHHHHHHHHHHHHHHHHHHHCCCCCCCC
090	EEEARKQQEQYNNESEQKRYQQYGFGQGHSN	-44.54	35.5	HHHHHHHHHHHHHHHHHHHHHHHHCCCCCCCC
694	EEEARKFQEKYSNESEQKRYQQQGFGQGHQS	-44.53	38.7	HHHHHHHHHHHHHHHHHHHHHHHHCCCCCCCC
124	EEEEKKFQEKYNNESEQKRYQRYGFGQGHQS	-44.53	41.9	CHHHHHHHHHHHHHHHHHHHHHHHCCCCCCCC
820	EEEERKFQEKYNNEAEQKRYQRYGMGQGHQP	-44.53	41.9	CHHHHHHHHHHHHHHHHHHHHHHHHCCCCCCC
830	EEEKKKFLEKFNNEYEQEYYQQNGSGQGHQY	-44.53	48.4	HHHHHHHHHHHHHHHHHHHHHHHHCCCCCCCC
604	EEEKRKFLEKFNNEYEQKYYQQKGFGQGHQS	-44.53	45.2	HHHHHHHHHHHHHHHHHHHHHHHHCCCCCCCC
680	EEEKRKFLEKFNNEYEQKYYQQKGQGQGHSH	-44.53	45.2	HHHHHHHHHHHHHHHHHHHHHHHHHCCCCCCC
592	EEEKRKFLEKMKNEKEQEEYQRYGFGQGHSN	-44.53	38.7	HHHHHHHHHHHHHHHHHHHHHHHHCCCCCCCC
415	EEEKRKFLEKYNNEAEQKRYQRYGFGQGHQS	-44.53	45.2	HHHHHHHHHHHHHHHHHHHHHHHHCCCCCCCC
567	EEEKKKLLEQIENEREQQEYQRQGFGQGHQN	-44.49	35.5	HHHHHHHHHHHHHHHHHHHHHHHHCCCCCCCC
927	EEEKRKMLQQFNNEFEDQFYQQKGFGKGNSN	-44.46	48.4	HHHHHHHHHHHHHHHHHHHHHHHHCCCCCCCC
677	EEEKRKLLQKINNEEEQKRYQREGQGQGHQS	-44.45	38.7	HHHHHHHHHHHCHHHHHHHHHHHHHCCCCCCC
306	EEEKRKMLQQINQEEEDQRYQRYGFGKGDSR	-44.44	48.4	HHHHHHHHHHHHHHHHHHHHHHHHCCCCCCCC
435	EEEKKKMLEKINQEAEQKRYQREGMGQGHQP	-44.43	45.2	HHHHHHHHHHHHHHHHHHHHHHHHHHCCCCCC
606	EEEKKKMLEKINQEEEQKRYQREGMGQGHQP	-44.43	41.9	HHHHHHHHHHHHHHHHHHHHHHHHHCCCCCCC
287	EEERRKQLEKLNQEKEQKRYQQYGFGQGHQS	-44.40	38.7	HHHHHHHHHHHHHHHHHHHHHHHHCCCCCCCC
630	EEEKRKMLDQYNNESEQQRYQQYGFGQGDSR	-44.40	45.2	HHHHHHHHHHHHHHHHHHHHHHHHCCCCCCCC
475	EEEKKKWLEKLNNEKEQKRYQRYGMGQGHQP	-44.39	41.9	HHHHHHHHHHHHHHHHHHHHHHHHHCCCCCCC
275	EEEKKKWLEKLNNEYEQQEYQRNGRGQGHSY	-44.39	41.9	HHHHHHHHHHHHHHHHHHHHHHHHCCCCCCCC
299	EEEKRKWLEKLNNEKEQERYQQQGMGQGHQP	-44.39	38.7	HHHHHHHHHHHHHHHHHHHHHHHHHCCCCCCC
992	EEEWRKWIEKLNNEKEQEEYQRKGRGQGHQS	-44.39	35.5	HHHHHHHHHHHHHHHHHHHHHHHHHCCCCCCC
047	EEEWRKWIEKLNNEKEQKRYQREGMGQGHQP	-44.39	35.5	HHHHHHHHHHHHHHHHHHHHHHHHHHCCCCCC
563	EQERRKQLEQYNNEAEQQRYQRYGFGQGDSR	-44.39	41.9	HHHHHHHHHHHHHHHHHHHHHHHHCCCCCCCC
911	EEEKRKFLQQYNEEWEQMRYQREGFGQGDSR	-44.35	45.2	HHHHHHHHHHHHHHHHHHHHHHHHHCCCCCCC
430	EEEKKKLLEQINNEAEQKRYQKEGFGQGHQS	-44.32	41.9	HHHHHHHHHHHHHHHHHHHHHHHHCCCCCCCC
113	EEEKKKLLEQINNEAEQKRYQRYGFGQGHQS	-44.32	41.9	HHHHHHHHHHHHHHHHHHHHHHHHCCCCCCCC
889	EEEKKKLLEQINNEEEQKRYQRYGMGQGHQP	-44.32	38.7	HHHHHHHHHHHCHHHHHHHHHHHHHCCCCCCC
039	EEEKKKLLEQINNEKEQEEYQRRGMGQGHQP	-44.32	38.7	HHHHHHHHHHHHHHHHHHHHHHHHHHCCCCCC
450	EEEKKKLLEQINNEQEQERYQQQGWGQGHTP	-44.32	38.7	HHHHHHHHHHHHHHHHHHHHHHHHCCCCCCCC
999	EEEKRKLLEQINNEAEQKRYQREGWGQGHNP	-44.32	38.7	HHHHHHHHHHHHHHHHHHHHHHHHHCCCCCCC
374	EEEKRKLLEQINNEEEQKRYQREGMGQGHQP	-44.32	35.5	HHHHHHHHHHHCHHHHHHHHHHHHHCCCCCCC
140	EEEKRKLLEQINNEEEQKRYQRQGFGQGHQS	-44.32	35.5	HHHHHHHHHHHCHHHHHHHHHHHHCCCCCCCC
641	EEEKRKLLEQINNEEEQQRYQQQGFGQGHQN	-44.32	35.5	HHHHHHHHHHHCHHHHHHHHHHHHCCCCCCCC
588	EEEKRKLLEQINNEKEQEEYQRRGMGQGHQP	-44.32	35.5	HHHHHHHHHHHHHHHHHHHHHHHHHHCCCCCC
494	EEEKRKLLEQINNEKEQEEYQRYGFGQGHQS	-44.32	35.5	HHHHHHHHHHHHHHHHHHHHHHHHCCCCCCCC
269	EEEKRKLLEQINNEKEQQEYQKQGFGQGHQS	-44.32	35.5	HHHHHHHHHHHHHHHHHHHHHHHHCCCCCCCC
446	EEEKRKLLEQINNEQEQEEYQRKGRGQGHQP	-44.32	35.5	HHHHHHHHHHHHHHHHHHHHHHHHHCCCCCCC
918	EEEKRKLLEQINNEQEQKSYQQQGFGQGHQS	-44.32	35.5	HHHHHHHHHHHHHHHHHHHHHHHHCCCCCCCC
209	EEEKRKLLEQINNEQEQKSYQREGMGQGHQP	-44.32	35.5	HHHHHHHHHHHHHHHHHHHHHHHHHCCCCCCC
635	EEEKRKLLEQINNEQEQQYYQQYGFGQGHSS	-44.32	35.5	HHHHHHHHHHHHHHHHHHHHHHHHCCCCCCCC
308	EEEKRKLLEQLNNEKEQQRYQRYGFGQGHQS	-44.32	35.5	HHHHHHHHHHHHHHHHHHHHHHHHCCCCCCCC
633	EEELKKLLEQINNEEEQKRYQRYGFGQGHQS	-44.32	38.7	HHHHHHHHHHHCHHHHHHHHHHHHCCCCCCCC
216	EQEKRKLLEQLNNEKEQQRYQRQGFGQGHQS	-44.32	32.3	HHHHHHHHHHHHHHHHHHHHHHHHCCCCCCCC
541	EEERRKQLEKINNEKEQQRYQQQGFGQGHQS	-44.30	38.7	HHHHHHHHHHHHHHHHHHHHHHHHCCCCCCCC
994	EEEKRKFLEKYNNESEQKRYQQQGFGQGHSS	-44.30	41.9	HHHHHHHHHHHHHHHHHHHHHHHHCCCCCCCC
558	EEEKRKFLDQYNNEAEQQRYQRYGFGQGDSR	-44.29	51.6	HHHHHHHHHHHHHHHHHHHHHHHHCCCCCCCC
998	EEEWKKFIEQYNNDYEDEEYQRKGRGKGDSR	-44.21	48.4	HHHHHHHHHHHHHHCHHHHHHHHHCCCCCCCC
828	EEEKRKMLEKINNEAEQKRYQRYGFGQGHQS	-44.20	41.9	HHHHHHHHHHHHHHHHHHHHHHHHCCCCCCCC
784	EQEKRHLLEKINNEAEDKRYQREGFGKGDSR	-44.19	51.6	HHHHHHHHHHHHHHHHHHHHHHHHHCCCCCCC
176	EQEKRHFLEQYNNESEDQRYQRYGFGKGHQS	-44.18	41.9	HHHHHHHHHHHHCCHHHHHHHHHHCCCCCCCC
171	EEERRKQLDQINNEAEQQRYQRYGFGQGNSN	-44.17	41.9	HHHHHHHHHHHHHHHHHHHHHHHHCCCCCCCC
053	EEEWRKLMDAVNNREEQERYQQQGWGQGHTP	-44.15	32.3	HHHHHHHHHHHHHHHHHHHHHHHHCCCCCCCC

244	EEEKRKLLEQINQEEEQERYQQQGMGQGHQP	-44.15	35.5	HHHHHHHHHHHHHHHHHHHHHHHCCCCCCCC
153	EEEKKKMLEKINNEEEQKRYQREGRGQGHQP	-44.13	41.9	HHHHHHHHHHHCHHHHHHHHHHHCCCCCCCC
612	EEEKKKMLEKINNEEEQKRYQRYGFGQGHSN	-44.13	41.9	HHHHHHHHHHHCHHHHHHHHHHHCCCCCCCC
523	EEEKRKMLEKFNNEEEQKRYQREGFGQGHQS	-44.13	41.9	HHHHHHHHHHHCCHHHHHHHHHHCCCCCCCC
627	EEEKRKMLEKFNNEFEQKYYQQKGQGQGHSH	-44.13	41.9	HHHHHHHHHHHHHHHHHHHHHHHHCCCCCCC
178	EEEKRKMLEKINNEEEQKRYQRYGMGQGHQP	-44.13	38.7	HHHHHHHHHHHCHHHHHHHHHHHCCCCCCCC
335	EEEKRKMLEKYNNEYEQEEYQRKGRGQGHQP	-44.13	38.7	HHHHHHHHHHHHHHHHHHHHHHHHCCCCCCC
936	EQEKRKMLEKINNEKEQKEYQRAGFGQGHQS	-44.13	35.5	HHHHHHHHHHHHHHHHHHHHHHHCCCCCCCC
276	EEERKKQLEKINNEEEQKSYQREGMGQGHQP	-44.11	41.9	HHHHHHHHHHHHHHHHHHHHHHHHCCCCCCC
853	EEERRKQLEKINNEEEQKRYQRYGFGQGHQS	-44.11	38.7	HHHHHHHHHHHCHHHHHHHHHHHCCCCCCCC
466	EEERRKQLEKYNNEYEQKEYQRTGRGQGHSF	-44.11	38.7	HHHHHHHHHHHHHHHHHHHHHHHCCCCCCCC
022	EEEKRKFLEQYNNEYEQEEFQRKGRGQGKQN	-44.08	35.5	HHHHHHHHHHHHHHHHHHHHHHHHHCCCCCC
867	EEERRKQLDAINNRKEQEEYQRYGFGQGHQS	-44.06	35.5	HHHHHHHHHHHHHHHHHHHHHHHCCCCCCCC
605	EEEKKKFLEQFNQEYEQQYYQQQGFGQGHSS	-44.06	45.2	HHHHHHHHHHHHHHHHHHHHHHHCCCCCCCC
787	EEEKRKFLEQYNQEYEQQEYQRNGRGQGHSY	-44.06	38.7	HHHHHHHHHHHHHHHHHHHHHHHCCCCCCCC
191	EEEQRKFEEQRNQEEEQQRYQQYGFGQGHQS	-44.06	35.5	HHHHHHHHHHHHHHHHHHHHHHHCCCCCCCC
871	EEEKRKFLEKFNNEFEQKYYQQKGFGQGHSS	-44.05	45.2	HHHHHHHHHHHHHHHHHHHHHHHCCCCCCCC
057	EEEKRKFLEKYNNESEQKRYQRYGFGQGHQS	-44.05	41.9	HHHHHHHHHHHCHHHHHHHHHHHCCCCCCCC
509	EEEKRKFLEKYNNEYEQKEYQRTGRGQGHQS	-44.05	41.9	HHHHHHHHHHHHHHHHHHHHHHHCCCCCCCC
432	EEEKKKLLEDINQRKEDQEYQKQGFGKGHSS	-44.05	41.9	HHHHHHHHHHHHHHHHHHHHHHHCCCCCCCC
211	EEEKRKFLEQYNQRAEQERYQREGKGQGHSS	-44.03	38.7	HHHHHHHHHHHHHHHHHHHHHHHCCCCCCCC
550	EEEKRKILEKINNEEEQRRYQQYGFGQGHQN	-44.02	38.7	HHHHHHHHHHHCHHHHHHHHHHHCCCCCCCC
213	EEEKRKMLEKYNQEEEQKKYQREGKGQGHSH	-43.95	38.7	HHHHHHHHHHHHHHHHHHHHHHHHCCCCCCC
817	EEEKRKWLEKLNNEKEQKRYQKEGFGQGHQS	-43.92	38.7	HHHHHHHHHHHHHHHHHHHHHHHHCCCCCCC
795	EEEKRKLLEDIENRREDQEYQQKGRGKGHQP	-43.92	35.5	HHHHHHHHHHHHHHHHHHHHHHHCCCCCCCC
019	EEEQRKMLEKINNEKEQEEYQRRGMGQGHQP	-43.90	38.7	HHHHHHHHHHHHHHHHHHHHHHHHHCCCCCC
966	EEEKRKFLQKKNNEEEQKRYQQYGFGQGHQS	-43.89	41.9	HHHHHHHHHHHCHHHHHHHHHHHCCCCCCCC
224	EQEKRKMLEQFNNEYEDEEYQRQGRGKGHQS	-43.88	41.9	HHHHHHHHHHHHHHHHHHHHHHHCCCCCCCC
117	EEEKRKMLDQINNEKEQKEYQRAGFGQGDSR	-43.87	45.2	HHHHHHHHHHHHHHHHHHHHHHHCCCCCCCC
570	EEEKRKYLEQYNNEAEQKRYQREGMGQGHQP	-43.86	38.7	HHHHHHHHHHHHHHHHHHHHHHHHHCCCCCC
855	EQERRKQLEQINNEAEDKRYQREGMGKGHQP	-43.85	41.9	HHHHHHHHHHHHHHHHHHHHHHHHHCCCCCC
249	EEEKRKLLEKINNEQEQKRYQREGKGQGDSR	-43.85	45.2	HHHHHHHHHHHHHHHHHHHHHHHCCCCCCCC
270	EEEKKKLLEQINNEQEQEEYQRKGRGQGHQP	-43.85	38.7	HHHHHHHHHHHHHHHHHHHHHHHHCCCCCCC
268	EEEKRKLLEQINNEAEQKRYQREGQGQGHQS	-43.85	38.7	HHHHHHHHHHHHHHHHHHHHHHHCCCCCCCC
110	EEEKRKLLEQINNEEEQKRYQREGKGQGHSH	-43.85	35.5	HHHHHHHHHHHCHHHHHHHHHHHHCCCCCCC
051	EEEKRKLLEQLNNEKEQQRYQQYGFGQGHQS	-43.85	35.5	HHHHHHHHHHHHHHHHHHHHHHHCCCCCCCC
128	EEEKKKLLEQINNEKEQKEYQKQGWGQGHNP	-43.83	38.7	HHHHHHHHHHHHHHHHHHHHHHHCCCCCCCC
220	EEEKKKFLEKYNNEYEQKEYQRTGRGQGDSR	-43.80	51.6	HHHHHHHHHHHHHHHHHHHHHHHCCCCCCCC
410	EEEKRKFLEKYNNEYEQKEYQRTGRGQGDSR	-43.80	48.4	HHHHHHHHHHHHHHHHHHHHHHHCCCCCCCC
812	EQEKRHMLEQINNEKEDEEYQRRGMGKGHQP	-43.79	38.7	HHHHHHHHHHHHHHHHHHHHHHHHHCCCCCC
471	EEEKKKFLEQKNNEEEQQRYQRYGFGQGHSS	-43.76	41.9	HHHHHHHHHHHCCHHHHHHHHHHCCCCCCCC
449	EEEKKKFLEQYNNEYEQMFYQQKGFGQGHSS	-43.76	45.2	HHHHHHHHHHHHHHHHHHHHHHHHCCCCCCC
590	EEEKKKFLEQYNNEYEQQRYQQEGMGQGHQP	-43.76	41.9	HHHHHHHHHHHHHHHHHHHHHHHHHCCCCCC
900	EEEKKKFLEQYNNEYEQQYYQQNGSGQGHSY	-43.76	41.9	HHHHHHHHHHHHHHHHHHHHHHHCCCCCCCC
568	EEEKKKFLEQYSNEYEQEEYQRNGRGQGHSY	-43.76	38.7	HHHHHHHHHHHHHHHHHHHHHHHCCCCCCCC
127	EEEKRKFLEQFNNEYEQMSYQREGKGQGHSH	-43.76	41.9	HHHHHHHHHHHHHHHHHHHHHHHCCCCCCCC
873	EEEKRKFLEQKNNEEEQQRYQQQGMGQGHQP	-43.76	38.7	HHHHHHHHHHHCCHHHHHHHHHHCCCCCCCC
226	EEEKRKFLEQMKNEKEQEEYQRRGMGQGHQP	-43.76	35.5	HHHHHHHHHHHHHHHHHHHHHHHHHCCCCCC
228	EEEKRKFLEQYNNEAEQQQYQRNGSGQGHSY	-43.76	41.9	HHHHHHHHHHHHHHHHHHHHHHHCCCCCCCC
659	EEEKRKFLEQYNNESEQKRYQRYGFGQGHSS	-43.76	38.7	HHHHHHHHHHHHHHHHHHHHHHHCCCCCCCC
809	EEEKRKFLEQYNNESEQQRYQQQGQGQGHQS	-43.76	38.7	HHHHHHHHHHHHHHHHHHHHHHHHHCCCCCC
841	EEEKRKFLEQYNNESEQQRYQRYGFGQGHQS	-43.76	38.7	HHHHHHHHHHHHCHHHHHHHHHHCCCCCCCC
438	EEEKRKFLEQYNNEWEQEEYQRKGRGQGHQS	-43.76	38.7	HHHHHHHHHHHHHHHHHHHHHHHHCCCCCCC
717	EEEKRKFLEQYNNEYEQEEYQRNGRGQGHSY	-43.76	38.7	HHHHHHHHHHHHHHHHHHHHHHHCCCCCCCC
661	EEEKRKFLEQYNNEYEQQEYQRTGRGQGHSF	-43.76	38.7	HHHHHHHHHHHHHHHHHHHHHHHHCCCCCCC
726	EEEKRKFLEQYSNEAEQQRYQKEGWGQGHNP	-43.76	38.7	HHHHHHHHHHHHHHHHHHHHHHHHCCCCCCC
384	EEEQKKFEEQRNNEEEQERYQQQGFGQGHQS	-43.76	38.7	CHHHHHHHHHHHHHHHHHHHHHHCCCCCCCC
011	EQEERKFQEQYNNEEEQQKYQQEGKGQGHSH	-43.76	32.3	CHHHHHHHHHHCHHHHHHHHHHHCCCCCCCC
271	EQEKRKFLEQYNNEEEQMRYQREGKGQGHSH	-43.76	35.5	HHHHHHHHHHHCHHHHHHHHHHHHCCCCCCC
935	EQEKRKFLEQYNNEYEQEEYQRTGRGQGHSF	-43.76	35.5	HHHHHHHHHHHHHHHHHHHHHHHHCCCCCCC
959	EEQKRKMLDQINQEKEQEEYQRYGFGQGDSR	-43.76	48.4	HHHHHHHHHHHHHHHHHHHHHHHCCCCCCCC
613	EEEKRKLLEDINNRAEDQRYQRYGFGKGHSS	-43.76	41.9	HHHHHHHHHHHHHHHHHHHHHHHCCCCCCCC
227	EEEKRKFLEQYNNRYEQEYYQQKGFGQGHQS	-43.74	35.5	HHHHHHHHHHHHHHHHHHHHHHHCCCCCCCC
608	EEEKRKLLEKINNEAEQRRYQREGKGQGHSH	-43.70	41.9	HHHHHHHHHHHHHHHHHHHHHHHHCCCCCCC
678	EEEKRKMLEQINNEAEQKRFQQQGFGQGKQN	-43.69	35.5	HHHHHHHHHHHHHHHHHHHHHHHCCCCCCCC
575	EEEKKKMLEKINNEAEDKRYQRYGFGKGHQS	-43.68	51.6	HHHHHHHHHHHHHHHHHHHHHHHCCCCCCCC
710	EEQRKKQLEKINNEAEQKRYQREGMGQGHQP	-43.67	48.4	HHHHHHHHHHHHHHHHHHHHHHHHHCCCCCC

808	EEEKRKMLEQINQEAEQQRYQKEGMGQGHQP	-43.66	38.7	HHHHHHHHHHHHHHHHHHHHHHHHCCCCCCC
397	EEEKRKMLEQINQEEEQKRYQQQGFGQGHQN	-43.66	35.5	HHHHHHHHHHHHHHHHHHHHHHHHCCCCCCC
906	EQEKRKMLEQYNQEYEQQEYQRNGRGQGHSY	-43.66	32.3	HHHHHHHHHHHHHHHHHHHHHHHHCCCCCCC
572	EEEQRKMLEKINNEKEQKEYQKQGWGQGHNP	-43.66	38.7	HHHHHHHHHHHHHHHHHHHHHHHHCCCCCCC
163	EQERRKQLEQINQDEEQRRYQQQGMGQGHQP	-43.65	29.0	HHHHHHHHHHHHHHHHHHHHHHHHCCCCCCC
029	EEERRKQLEQINQEEEQKRYQREGFGQGHQS	-43.64	35.5	HHHHHHHHHHHHHHHHHHHHHHHHCCCCCCC
286	EEERRKQLEQINQEKEQEEYQRYGMGQGHQP	-43.64	35.5	HHHHHHHHHHHHHHHHHHHHHHHHHHCCCCC
763	EEEERKFQEKYNNEYEQKYYQQQGFGQGHQS	-43.63	38.7	CHHHHHHHHHHHHHHHHHHHHHHHCCCCCCC
667	EEEKKKWLEQLNNEKEQQRYQREGMGQGHQP	-43.63	38.7	HHHHHHHHHHHHHHHHHHHHHHHHHHCCCCC
065	EEEKRKWLEQLNNEQEQQRYQRQGFGQGHQS	-43.63	35.5	HHHHHHHHHHHHHHHHHHHHHHHHCCCCCCC
445	EEEKRKMLEQINNEAEQEQFQQRGMGQGKQP	-43.62	35.5	HHHHHHHHHHHHHHHHHHHHHHHHCCCCCCC
457	EEEERKFQEKYNNEAEQKRYQREGMGQGHQP	-43.61	41.9	CHHHHHHHHHHHHHHHHHHHHHHHHHCCCCC
736	EEERRKQLDAFNNRYEQQYYQQKGFGQGHSS	-43.59	38.7	HHHHHHHHHHHHHHHHHHHHHHHHCCCCCCC
760	EEELKKFLEKMKQEKEQEEYQRYGFGQGDSR	-43.58	48.4	HHHHHHHHHHHHHHHHHHHHHHHHCCCCCCC
600	EEEKRKFLEQYNNEEEQMRYQREGFGQGHQS	-43.53	38.7	HHHHHHHHHHCHHHHHHHHHHHHHHHCCCCC
143	EEEKRKLLEQINNEEEQKRYQRYGFGQGHQS	-43.53	35.5	HHHHHHHHHHCHHHHHHHHHHHHHCCCCCCC
382	DEERKKMLDDINNRKEDEEYQRYGFGKGDSR	-43.47	48.4	HHHHHHHHHHHHHHHHHHHHHHHHCCCCCCC
068	EEELRKLLEQINNEEEQQRYQKEGFGQGHQS	-43.43	35.5	HHHHHHHHHHCHHHHHHHHHHHHHCCCCCCC
346	EEEKRKFLEQFNNEYEQRRYQQEGRGQGHQR	-43.39	45.2	HHHHHHHHHHHHHHHHHHHHHHHHCCCCCCC
074	EEELKKLLEQINNEKEQKEYQKQGFGQGNQN	-43.39	38.7	HHHHHHHHHHHHHHHHHHHHHHHHCCCCCCC
061	EEEKKKMLEQINNEEEQKRYQREGWGQGHNP	-43.37	38.7	HHHHHHHHHHCHHHHHHHHHHHHHCCCCCCC
930	EEEKKKMLEQINNEEEQKSYQREGKGQGHSH	-43.37	38.7	HHHHHHHHHHHHHHHHHHHHHHHHCCCCCCC
281	EEEKKKMLEQINNEEEQQRYQRYGMGQGHQP	-43.37	38.7	HHHHHHHHHHCHHHHHHHHHHHHHCCCCCCC
441	EEEKKKMLEQINNEKEQEEYQRRGKGQGHSS	-43.37	38.7	HHHHHHHHHHHHHHHHHHHHHHHHCCCCCCC
101	EEEKKKMLEQINNEKEQEEYQRRGMGQGHQP	-43.37	38.7	HHHHHHHHHHHHHHHHHHHHHHHHHHCCCCC
114	EEEKKKMLEQINNEKEQQEYQKMGLGQGHQP	-43.37	41.9	HHHHHHHHHHHHHHHHHHHHHHHHCCCCCCC
376	EEEKKKMLEQINNEKEQQEYQRSGMGQGHQP	-43.37	41.9	HHHHHHHHHHHHHHHHHHHHHHHHCCCCCCC
201	EEEKRKMLEQINNEEEQERYQRYGFGQGHQS	-43.37	35.5	HHHHHHHHHHCCHHHHHHHHHHHHCCCCCCC
493	EEEKRKMLEQINNEEEQQNYQREGKGQGHSH	-43.37	35.5	HHHHHHHHHHHHHHHHHHHHHHHHCCCCCCC
826	EEEKRKMLEQINNEEEQQRYQREGMGQGHQP	-43.37	35.5	HHHHHHHHHHCHHHHHHHHHHHHHHHCCCCC
241	EQEKRKMLEQINNEAEQKRYQRYGFGQGHSS	-43.37	35.5	HHHHHHHHHHHHHHHHHHHHHHHHCCCCCCC
860	EQEKRKMLEQINNEEEQKRYQREGFGQGHQS	-43.37	32.3	HHHHHHHHHHCHHHHHHHHHHHHHCCCCCCC
182	EQEKRKMLEQINNEEEQQRYQQQGMGQGHQP	-43.37	32.3	HHHHHHHHHHCHHHHHHHHHHHHHCCCCCCC
868	EEERKKQLEQFNNEYEQQFYQKEGFGQGHQS	-43.34	45.2	HHHHHHHHHHHHHHHHHHHHHHHHCCCCCCC
334	EEERKKQLEQINNEEEQKRYQREGMGQGHQP	-43.34	38.7	HHHHHHHHHHCHHHHHHHHHHHHHCCCCCCC
903	EEERRKQLEQINNEAEQKRYQRYGMGQGHQP	-43.34	38.7	HHHHHHHHHHHHHHHHHHHHHHHHCCCCCCC
141	EEERRKQLEQINNEEEQKRYQREGMGQGHQP	-43.34	35.5	HHHHHHHHHHCHHHHHHHHHHHHHCCCCCCC
055	EEERRKQLEQINNEEEQQRYQRYGWGQGHNP	-43.34	35.5	HHHHHHHHHHCHHHHHHHHHHHHHCCCCCCC
548	EQERRKQLEQINNEAEQKRYQREGMGQGHQP	-43.34	35.5	HHHHHHHHHHHHHHHHHHHHHHHHHHCCCCC
081	EQERRKQLEQINNEEEQQRYQRYGFGQGHQS	-43.34	32.3	HHHHHHHHHHCHHHHHHHHHHHHHCCCCCCC
158	EEEKRKFLEKYNNEYEQEEYQRKGRGQGDSR	-43.32	48.4	HHHHHHHHHHHHHHHHHHHHHHHHCCCCCCC
251	EEEKKKFLEQFNNEFEQQYYQQKGFGQGHQS	-43.29	45.2	HHHHHHHHHHHHHHHHHHHHHHHHCCCCCCC
752	EEEKKKFLEQYNNEYEQMEYQRTGRGQGHQF	-43.29	41.9	HHHHHHHHHHHHHHHHHHHHHHHHCCCCCCC
980	EEEKKKFLEQYNNEYEQQEYQRTGRGQGHQF	-43.29	41.9	HHHHHHHHHHHHHHHHHHHHHHHHCCCCCCC
444	EEEKRKFLEQFNNEFEQQYYQQKGQGQGHSH	-43.29	41.9	HHHHHHHHHHHHHHHHHHHHHHHHCCCCCCC
709	EEEKRKFLEQYNNEYEQEEYQRKGRGQGHQS	-43.29	38.7	HHHHHHHHHHHHHHHHHHHHHHHHCCCCCCC
372	EEEKRKFLEQYNNEYEQQYYQQQGFGQGHSS	-43.29	38.7	HHHHHHHHHHHHHHHHHHHHHHHHCCCCCCC
126	EEELKEFLKQFNEEFEDKYYQQKGFGKGDSR	-43.25	58.1	HHHHHHHHHHHHHHHHHHHHHHHHCCCCCCC
085	EEERRKQLEQINNEEEQKRYQREGQGQGHQS	-43.23	35.5	HHHHHHHHHHCHHHHHHHHHHHHHCCCCCCC
381	EEEKKKMLEKINNEEEQKRYQREGKGQGHSH	-43.22	41.9	HHHHHHHHHHCHHHHHHHHHHHHHCCCCCCC
938	EEEKKKMLEKFNNEYEQEEYQRKGRGQGNQN	-43.20	45.2	HHHHHHHHHHHHHHHHHHHHHHHHCCCCCCC
743	EEEKKKMLEKINNEKEQEEYQRYGFGQGNQN	-43.20	41.9	HHHHHHHHHHHHHHHHHHHHHHHHCCCCCCC
846	EEEKRKFLEDYNNRYEDEFYQQKGFGKGHSS	-43.19	45.2	HHHHHHHHHHHHHHHHHHHHHHHHCCCCCCC
327	EEEKRKFLEDYNNRYEDMQYQKEGMGKGHQP	-43.19	41.9	HHHHHHHHHHHHHHHHHHHHHHHHHHCCCCC
025	EEELKKLLEKINNEEEQKRYQRYGFGQGDSR	-43.18	48.4	HHHHHHHHHHCHHHHHHHHHHHHHCCCCCCC
434	EQERRHQLEKYNNEYEQEEYQRKGRGQGDSR	-43.15	41.9	HHHHHHHHHHHHHHHHHHHHHHHHCCCCCCC
515	EQERRHQLEKINNEKEDREYQRRGMGKGHQP	-43.14	41.9	HHHHHHHHHHHHHHHHHHHHHHHHCCCCCCC
145	EEEKRKFLQQYNNESEQQRYQRYGFGQGHQS	-43.12	38.7	HHHHHHHHHHHHHHHHHHHHHHHHCCCCCCC
332	EEERRKQLEHYNNRSEDQRYQQYGFGKGHQS	-43.12	38.7	HHHHHHHHHHHHCCCHHHHHHHHHCCCCCCC
356	EEEKRKLLEQLNNEKEQMRYQRYGFGQGDSR	-43.09	41.9	HHHHHHHHHHHHHHHHHHHHHHHHCCCCCCC
388	EEERKKQLEDINQRAEDQRYQRYGMGKGHQP	-43.07	45.2	HHHHHHHHHHHHHHHHHHHHHHHHCCCCCCC
666	EEEKKKFLEQYNNDYEQREYQRKGRGQGDSR	-43.04	45.2	HHHHHHHHHHHHHHHHHHHHHHHHCCCCCCC
132	EEEKKKWLEQLNNEKEQKRYQREGMGQGHQP	-43.03	38.7	HHHHHHHHHHHHHHHHHHHHHHHHHHCCCCC
200	EQEKRKFLEQFNNEYEQQFYQQKGFGQGDSR	-43.03	48.4	HHHHHHHHHHHHHHHHHHHHHHHHCCCCCCC
716	EEEKKKFLEKYNNEYEQKYYQQKGFGQGDSR	-43.02	51.6	HHHHHHHHHHHHHHHHHHHHHHHHCCCCCCC
375	EEEKRKLLDDINNRAEQQRYQREGKGQGHSS	-43.01	38.7	HHHHHHHHHHHHHHHHHHHHHHHHCCCCCCC
557	EEEKKKMLEQINNEAEQRRYQREGRGQGHQP	-43.00	41.9	HHHHHHHHHHHHHHHHHHHHHHHHCCCCCCC

822	EEQKRKMLEQINNEEEQKRYQREGVGQGHQP	-42.93	38.7	HHHHHHHHHHHCHHHHHHHHHHHHCCCCCCC
864	EEEKRKMLEKINNEAEQKRYQRQGFGQGDSR	-42.89	48.4	HHHHHHHHHHHHHHHHHHHHHHHHCCCCCCC
240	EEEKKKMLEQINNRAEQERYQREGKGQGHSH	-42.87	38.7	HHHHHHHHHHHHHHHHHHHHHHHHCCCCCCC
341	EEERRKQLEKINNEEEQKRYQRYGFGQGDSR	-42.87	45.2	HHHHHHHHHHHCHHHHHHHHHHHHCCCCCCC
781	EEERKKQLEQINNEEEQQRYQQYGFGQGHQS	-42.87	38.7	HHHHHHHHHHHCHHHHHHHHHHHHCCCCCCC
504	EEERRKQLEQINNEAEQKRYQRYGFGQGHSS	-42.87	38.7	HHHHHHHHHHHHHHHHHHHHHHHHCCCCCCC
354	EEERRKQLEQINNEKEQQRYQQQGFGQGHSS	-42.87	35.5	HHHHHHHHHHHHHHHHHHHHHHHHCCCCCCC
766	EEEKKKMLQQINNEKEQEEYQRYGFGQGHQS	-42.73	38.7	HHHHHHHHHHHHHHHHHHHHHHHHCCCCCCC
967	EEEKRRLLEKINNEAEQKRYQQYGFGQGHSS	-42.68	41.9	HHHHHHHHHHHHHHHHHHHHHHHHCCCCCCC
544	EEEKKKMLEQINNEQEQQRYQQQGFGQGDSR	-42.64	45.2	HHHHHHHHHHHHHHHHHHHHHHHHCCCCCCC
965	EQEKRKMLEQFNNEYEQEEYQRKGRGQGDSR	-42.64	41.9	HHHHHHHHHHHHHHHHHHHHHHHHCCCCCCC
486	EEEKRKFLEKFNNEYEQKYYQQKGFGQGDSR	-42.62	51.6	HHHHHHHHHHHHHHHHHHHHHHHHCCCCCCC
186	EEEKKKFLEQYNNESEQQRYQRYGFGQGDSR	-42.52	48.4	HHHHHHHHHHHHCHHHHHHHHHHHCCCCCCC
607	EEEKRKFLEQYNNEYEQEEYQRKGRGQGDSR	-42.52	45.2	HHHHHHHHHHHHHHHHHHHHHHHHCCCCCCC
187	EEEKRKFLEQYNNEYEQQEYQRTGRGQGDSR	-42.52	45.2	HHHHHHHHHHHHHHHHHHHHHHHHCCCCCCC
165	EQEKRKFLEQKNNEEEQKRYQQYGFGQGDSR	-42.52	41.9	HHHHHHHHHHHCHHHHHHHHHHHHCCCCCCC
032	EEEKRKLLEQIEEEERQKRYQREGFGQGHQS	-42.49	29.0	HHHHHHHHHHHHHHHHHHHHHHHHHCCCCCC
050	EEERKKQLEQINNEEEQKRYQREGFGQGDSR	-42.47	45.2	HHHHHHHHHHHCHHHHHHHHHHHHCCCCCCC
452	EEEKRKMLEQFNNEYEQEEYQRKGRGQGNQN	-42.43	38.7	HHHHHHHHHHHHHHHHHHHHHHHHHCCCCCC
361	EEERRKQLEQINNEAEQKRYQREGKGQGNSN	-42.41	38.7	HHHHHHHHHHHHHHHHHHHHHHHHCCCCCCC
896	EEEYRKWLEQFKNEYEQEFYQQKGFGQGDSR	-42.40	45.2	HHHHHHHHHHHHHHHHHHHHHHHHCCCCCCC
071	EEEYRKWLEQLKNEKEQEEYQRRGKGQGDSR	-42.40	38.7	HHHHHHHHHHHHHHHHHHHHHHHHCCCCCCC
765	EEQKRKYLQQYNNEEEQRQYQRSGMGQGHQP	-42.31	41.9	HHHHHHHHHHHCCHHHHHHHHHHHCCCCCCC
707	EEEKRKFLEDYNNRSEDQRYQKEGFGKGNQN	-42.26	41.9	HHHHHHHHHHHHCCHHHHHHHHHHCCCCCCC
247	EEEKRKFLEQFNNEYEQKFYQKEGFGQGDSR	-42.20	51.6	HHHHHHHHHHHHHHHHHHHHHHHHCCCCCCC
136	EEEKRKLLEQINNEQEQQRYQREGFGQGDSR	-42.09	41.9	HHHHHHHHHHHHHHHHHHHHHHHHCCCCCCC
218	EQEKRHLLEQISNEEEQQRYQRQGFGQGHQS	-42.09	29.0	HHHHHHHHHHHCHHHHHHHHHHHHCCCCCCC
120	EEEKKKMLEQINNQKEQEEYQRYGFGQGDSR	-42.05	41.9	HHHHHHHHHHHHHHHHHHHHHHHHCCCCCCC
337	EEEKRKLLEAINNREEQMRYQREGKGQGHSS	-41.99	32.3	HHHHHHHHHHHHHHHHHHHHHHHHCCCCCCC
156	EQERRKQLEQINQEEEQQRYQQYGFGQGDSR	-41.50	38.7	HHHHHHHHHHHHHHHHHHHHHHHHCCCCCCC
658	EQEKRHFLEQFNNEKEQQYYQQYGFGQGHSS	-41.05	38.7	HHHHHHHHHHHHHHHHHHHHHHHHCCCCCCC
408	EEEKKKFLDDYNNRSEQQRYQRYGFGQGDSR	-40.82	48.4	HHHHHHHHHHHHHHHHHHHHHHHHCCCCCCC

Table S4. Summary of 877 peptide sequences designed using EvoEF2 and the evolutionary profile (weight = 0.75).

#label	#peptide	#binding(EEU)	#SeqID(%)	#Secondary structure
WT	EEQAKTFLDKFNHEAEDLFYQSSGLGKGDFR	-46.46	100.	HHHHHHHHHHHHHHHHHHHHHHHCCCCCCCC
376	EEELKKILEKLNQEKEDMRYQRQGFGKGHQS	-49.06	48.4	HHHHHHHHHHHHHHHHHHHHHHHHCCCCCCC
851	EEELRKILEKINQEKEDMEYQRAGFGKGHSS	-49.06	45.2	HHHHHHHHHHHHHHHHHHHHHHHHCCCCCCC
412	EEELKKLLDQINQEAEDMRYQRYGFGKGHSS	-49.05	51.6	HHHHHHHHHHHHHHHHHHHHHHHCCCCCCCC
403	EEELRKLLDQINQEEEDMRYQQQGFGKGHSA	-49.05	45.2	HHHHHHHHHHHHHHHHHHHHHHHHCCCCCCC
207	EEELRKLLDQINQEEEDMRYQRYGFGKGHSS	-49.05	45.2	HHHHHHHHHHHHHHHHHHHHHHHCCCCCCCC
957	EEELRKLLDQINQEKEDEEYQRYGFGKGHSA	-49.05	45.2	HHHHHHHHHHHHHHHHHHHHHHHCCCCCCCC
284	EEELRKLLDKINQEEEDMRYQQYGFGKGDSR	-49.03	54.8	HHHHHHHHHHHHHHHHHHHHHHHCCCCCCCC
434	EEELKKFLQKYNEEEEDMRYQREGFGKGHQS	-49.02	51.6	HHHHHHHHHHHHHHHHHHHHHHHHHCCCCCC
602	EQELRKILEKLNQEQEDMRYQRYGFGKGHSA	-48.87	41.9	HHHHHHHHHHHHHHHHHHHHHHHCCCCCCCC
816	EEELRKLLQQINEEAEDMRYQQQGFGKGHSA	-48.81	45.2	HHHHHHHHHHHHHHHHHHHHHHHHCCCCCCC
843	EEELKKILEKINNEAEDMRYQQQGFGKGHSS	-48.77	51.6	HHHHHHHHHHHHHHHHHHHHHHHHCCCCCCC
113	EEELKKILEKINNEAEDMRYQRYGFGKGHSS	-48.77	51.6	HHHHHHHHHHHHHHHHHHHHHHHCCCCCCCC
793	EEELKKILEKINNEEEDMRYQQYGFGKGHSA	-48.77	48.4	HHHHHHHHHHHHHHHHHHHHHHHCCCCCCCC
426	EEELKKILEKINNEEEDMRYQQYGFGKGHSS	-48.77	48.4	HHHHHHHHHHHHHHHHHHHHHHHCCCCCCCC
638	EEELRKILEKINNEEEDMRYQQQGFGKGHSS	-48.77	45.2	HHHHHHHHHHHHHHHHHHHHHHHHCCCCCCC
417	EEELRKILEKYNNEEEDMRYQREGFGKGHQA	-48.77	45.2	HHHHHHHHHHHCHHHHHHHHHHHHHCCCCCC
611	EEEWKKILEKLNNEQEDMRYQQNGFGKGHSA	-48.77	48.4	HHHHHHHHHHHHHHHHHHHHHHHHCCCCCCC
815	EEELKKLLDQINNEEEDMRYQRYGFGKGHQS	-48.77	48.4	HHHHHHHHHHHHHHHHHHHHHHHCCCCCCCC
968	EESLRKILDQINNEEEDMRFQQYGFGKGKSR	-48.76	45.2	HHHHHHHHHHHHHHHHHHHHHHHCCCCCCCC
574	EEELKKLLDQLNNEQEDEEYQRKGFGKGHSS	-48.75	48.4	HHHHHHHHHHHHHHHHHHHHHHHCCCCCCCC
139	EEELRKLLDQINNEAEDMRYQQQGFGKGHSA	-48.75	48.4	HHHHHHHHHHHHHHHHHHHHHHHHCCCCCCC
838	EEELRKLLDQINNEEEDMRYQQYGFGKGHSS	-48.75	45.2	HHHHHHHHHHHHHHHHHHHHHHHCCCCCCCC
972	EEELRKLLDQINNEEEDMRYQREGFGKGHSA	-48.75	45.2	HHHHHHHHHHHHHHHHHHHHHHHHHCCCCCC
872	EEELRKLLDQINNEEEDMRYQRYGFGKGHSA	-48.75	45.2	HHHHHHHHHHHHHHHHHHHHHHHCCCCCCCC
511	EEELRKLLDQLNNEKEDMRYQRQGFGKGHSA	-48.75	45.2	HHHHHHHHHHHHHHHHHHHHHHHHCCCCCCC
841	EEELRKILEKINNEEEDMRYQQYGFGKGHSA	-48.50	45.2	HHHHHHHHHHHHHHHHHHHHHHHCCCCCCCC
591	EEELRKFLDQFNQEYEDMFYQQKGFGKGHSS	-48.49	54.8	HHHHHHHHHHHHHHHHHHHHHHHHCCCCCCC
243	EEELRKFLDQYNQEEEDMRYQREGFGKGHSA	-48.49	48.4	HHHHHHHHHHHHHHHHHHHHHHHHHCCCCCC
026	EEELRKFLQQFNEEYEDMFYQQKGFGKGHSS	-48.25	51.6	HHHHHHHHHHHHHHHHHHHHHHHHCCCCCCC
402	EEELRKFLQQYNEEHEDMRYQQQGFGKGHSA	-48.25	45.2	HHHHHHHHHHHHHHHHHHHHHHHHCCCCCCC
143	EEELRKILEKINNEEEDMRYQQYGFGKGHQS	-48.25	45.2	HHHHHHHHHHHHHHHHHHHHHHHCCCCCCCC
317	EEELRKILEKYNNEEEDMRYQREGFGKGHSS	-48.25	45.2	HHHHHHHHHHHCHHHHHHHHHHHHCCCCCCC
125	EEELKKFLDQLNNEEEDMRYQRYGFGKGHSS	-48.19	51.6	HHHHHHHHHHHHHHHHHHHHHHHCCCCCCCC
080	EEELKKFLDQYNNEAEDMRYQQQGFGKGHQS	-48.19	54.8	HHHHHHHHHHHHHHHHHHHHHHHHCCCCCCC
319	EEELKKFLDQYNNEAEDMRYQQQGFGKGHSA	-48.19	54.8	HHHHHHHHHHHHHHHHHHHHHHHHCCCCCCC
563	EEELRKFLDQFNNEKEDMRYQQQGFGKGHSS	-48.19	51.6	HHHHHHHHHHHHHHHHHHHHHHHHCCCCCCC
848	EEELRKFLDQLNNEEEDMRYQQQGFGKGHQS	-48.19	48.4	HHHHHHHHHHHHHHHHHHHHHHHHCCCCCCC
646	EEELRKFLDQYNNEAEDMRYQRQGFGKGHSA	-48.19	51.6	HHHHHHHHHHHHHHHHHHHHHHHHCCCCCCC
998	EEELRKFLDQYNNEEEDMRYQREGFGKGHSA	-48.19	48.4	HHHHHHHHHHHHHHHHHHHHHHHHHCCCCCC
095	EEELRKFLDQYNNEEEDMRYQREGFGKGHSS	-48.19	48.4	HHHHHHHHHHHHHHHHHHHHHHHHCCCCCCC
571	EEELRKFLDQYNNEYEDMEYQRNGRGKGHSY	-48.19	48.4	HHHHHHHHHHHHHHHHHHHHHHCCCCCCCCC
781	EEELRKILEKINNEEEDMRYQQYGFGKGHSS	-48.17	45.2	HHHHHHHHHHHHHHHHHHHHHHHCCCCCCCC
610	EEELRKILEKLNNEKEDMRYQKEGFGKGHSA	-48.17	45.2	HHHHHHHHHHHHHHHHHHHHHHHHCCCCCCC
073	EEEKRKILEQFNQEYEDMFYQQQGFGKGHSS	-48.06	48.4	HHHHHHHHHHHHHHHHHHHHHHHHCCCCCCC
830	EEELRKILEQINQEAEDMRYQQQGFGKGHQS	-48.06	45.2	HHHHHHHHHHHHHHHHHHHHHHHHCCCCCCC
804	EEELKKLLEKINQEAEDMRYQQQGFGKGHSS	-48.05	51.6	HHHHHHHHHHHHHHHHHHHHHHHHCCCCCCC
444	EEELKKLLEKINQEAEDMRYQREGFGKGHSS	-48.05	51.6	HHHHHHHHHHHHHHHHHHHHHHHHHCCCCCC
428	EEELKKLLEKINQEEEDMRYQRQGFGKGHSA	-48.05	48.4	HHHHHHHHHHHHHHHHHHHHHHHHHCCCCCC
495	EEELRKLLEKINQEAEDIRYQQQGFGKGHSS	-48.05	48.4	HHHHHHHHHHHHHHHHHHHHHHHHCCCCCCC
064	EEELRKLLEKINQEEEDIRYQQQGFGKGHSS	-48.05	45.2	HHHHHHHHHHHHHHHHHHHHHHHHCCCCCCC
301	EEELRKLLEKINQEEEDMRYQREGFGKGHSS	-48.05	45.2	HHHHHHHHHHHHHHHHHHHHHHHHHCCCCCC
329	EEELRKLLEKLNQEKEDIRYQQQGFGKGHQS	-48.05	45.2	HHHHHHHHHHHHHHHHHHHHHHHHCCCCCCC
101	EEELKKLLEKINNEAEDMRFQQQGFGKGKSR	-48.03	51.6	HHHHHHHHHHHHHHHHHHHHHHHHCCCCCCC
390	EEELRKLLEKINNEAEDMRFQQQGFGKGKSR	-48.03	48.4	HHHHHHHHHHHHHHHHHHHHHHHHCCCCCCC
548	EEELRKLLEKINNEEEDMRFQREGFGKGKSR	-48.03	45.2	HHHHHHHHHHHHHHHHHHHHHHHHCCCCCCC
628	EEELKKLLDQINNEEEDMRYQQQGFGKGDSR	-48.01	54.8	HHHHHHHHHHHHHHHHHHHHHHHCCCCCCCC
723	EEELRKLLDQLNNEKEDMRYQKEGFGKGDSR	-48.01	51.6	HHHHHHHHHHHHHHHHHHHHHHHHCCCCCCC
735	EEELRKLLDQINQEEEDMRYQQQGFGKGDSR	-47.81	51.6	HHHHHHHHHHHHHHHHHHHHHHHCCCCCCCC
123	EEEKRKMLDQINNEEEDMRYQREGFGKGHSS	-47.79	45.2	HHHHHHHHHHHHHHHHHHHHHHHHHCCCCCC
869	EEELRKILEQINNEEEDMRYQQYGFGKGHSS	-47.78	41.9	HHHHHHHHHHHHHHHHHHHHHHHCCCCCCCC
619	EEELRKILEQYNNEEEDMRYQRQGFGKGHQS	-47.78	41.9	HHHHHHHHHHHCHHHHHHHHHHHHCCCCCCC
286	EEELKKILEQINNEAEDMRYQRQGFGKGHSA	-47.77	48.4	HHHHHHHHHHHHHHHHHHHHHHHHHCCCCCC
575	EEELKKILEQINNEEEDMRYQQQGFGKGHQS	-47.77	45.2	HHHHHHHHHHHHHHHHHHHHHHHHCCCCCCC

060	EEELKKILEQINNEKEDMRYQQQGFGKGHSS	-47.77	45.2	HHHHHHHHHHHHHHHHHHHHHHHHCCCCCCC
202	EEELRKILEQINNEEEDMRYQQQGFGKGHSS	-47.77	41.9	HHHHHHHHHHHHHHHHHHHHHHHHCCCCCCC
487	EEELRKILEQINNEEEDMRYQQYGFGKGHSA	-47.77	41.9	HHHHHHHHHHHHHHHHHHHHHHHCCCCCCCC
544	EEELRKILEQINNEKEDMEYQKQGFGKGHSA	-47.77	41.9	HHHHHHHHHHHHHHHHHHHHHHHHCCCCCCC
140	EEELRKILEQINNEKEDMEYQKQGFGKGHSS	-47.77	41.9	HHHHHHHHHHHHHHHHHHHHHHHCCCCCCCC
809	EEELRKILEQLNNEKEDMRYQKEGFGKGHSS	-47.77	41.9	HHHHHHHHHHHHHHHHHHHHHHHHCCCCCCC
001	EEELRKILEQLNNEKEDMRYQQQGFGKGHSS	-47.77	41.9	HHHHHHHHHHHHHHHHHHHHHHHHCCCCCCC
635	EEELRKILEQLNNEKEDMRYQRYGFGKGHSA	-47.77	41.9	HHHHHHHHHHHHHHHHHHHHHHHCCCCCCCC
607	EEELRKILEQLNNEQEDMRYQQQGFGKGHSA	-47.77	41.9	HHHHHHHHHHHHHHHHHHHHHHHHCCCCCCC
466	EEEWRKILEQINNEEEDMRYQREGFGKGHSA	-47.77	41.9	HHHHHHHHHHHHHHHHHHHHHHHHHCCCCCC
564	EEEWRKILEQLNNEKEDMRYQQNGFGKGHSS	-47.77	41.9	HHHHHHHHHHHHHHHHHHHHHHHCCCCCCCC
373	EEELRKLLEQLNNDKEDMRYQQQGFGKGHSS	-47.76	38.7	HHHHHHHHHHHHHHHHHHHHHHHHCCCCCCC
174	EEEKRKFLEKYNQESEDIRFQQQGFGKGKSR	-47.76	48.4	HHHHHHHHHHHHHHHHHHHHHHHCCCCCCCC
103	EEELKKLLEKINNEAEDIRYQQGGFGKGHES	-47.75	51.6	HHHHHHHHHHHHHHHHHHHHHHCCCCCCCCC
570	EEELKKLLEKINNEAEDIRYQQQGFGKGHSS	-47.75	51.6	HHHHHHHHHHHHHHHHHHHHHHHHCCCCCCC
685	EEELKKLLEKINNEAEDMRYQQQGFGKGHSA	-47.75	51.6	HHHHHHHHHHHHHHHHHHHHHHHHCCCCCCC
396	EEELKKLLEKINNEAEDMRYQRQGFGKGHSA	-47.75	51.6	HHHHHHHHHHHHHHHHHHHHHHHHHCCCCCC
171	EEELKKLLEKINNEAEDMRYQRQGFGKGHSS	-47.75	51.6	HHHHHHHHHHHHHHHHHHHHHHHHCCCCCCC
124	EEELKKLLEKINNEEEDIRYQQNGMGKGHSY	-47.75	48.4	HHHHHHHHHHHHHHHHHHHHHHHHCCCCCCC
799	EEELKKLLEKINNEEEDIRYQQQGFGKGHQS	-47.75	48.4	HHHHHHHHHHHHHHHHHHHHHHHHCCCCCCC
480	EEELKKLLEKINNEEEDIRYQQQGFGKGHSS	-47.75	48.4	HHHHHHHHHHHHHHHHHHHHHHHHCCCCCCC
692	EEELKKLLEKINNEEEDMRYQQQGFGKGHSA	-47.75	48.4	HHHHHHHHHHHHHHHHHHHHHHHHCCCCCCC
949	EEELKKLLEKINNEEEDMRYQQYGFGKGHSA	-47.75	48.4	HHHHHHHHHHHHHHHHHHHHHHHCCCCCCCC
092	EEELKKLLEKINNEEEDMRYQRQGFGKGHSA	-47.75	48.4	HHHHHHHHHHHHHHHHHHHHHHHHCCCCCCC
240	EEELKKLLEKINNEKEDEEYQRYGFGKGHSA	-47.75	48.4	HHHHHHHHHHHHHHHHHHHHHHHCCCCCCCC
445	EEELKKLLEKINNEQEDEEYQRVGRGKGHSY	-47.75	48.4	HHHHHHHHHHHHHHHHHHHHHHHHCCCCCCC
332	EEELKKLLEKLNNEKEDMRYQRYGFGKGHSA	-47.75	48.4	HHHHHHHHHHHHHHHHHHHHHHHCCCCCCCC
496	EEELKKLLEKLNNEKEDMYYQQKGFGKGHSS	-47.75	48.4	HHHHHHHHHHHHHHHHHHHHHHHHCCCCCCC
795	EEELKKLLEKLNNEQEDIRYQQGGFGKGHSF	-47.75	48.4	HHHHHHHHHHHHHHHHHHHHHHCCCCCCCCC
274	EEELKKLLEKLNNEQEDMQYQRAGFGKGHQS	-47.75	48.4	HHHHHHHHHHHHHHHHHHHHHHHHHCCCCCC
231	EEELRKLLEKINNEAEDIRYQQNGFGKGHSS	-47.75	48.4	HHHHHHHHHHHHHHHHHHHHHHHCCCCCCCC
637	EEELRKLLEKINNEAEDMRYQRQGFGKGHSS	-47.75	48.4	HHHHHHHHHHHHHHHHHHHHHHHHCCCCCCC
267	EEELRKLLEKINNEAEDMRYQRYGFGKGHSS	-47.75	48.4	HHHHHHHHHHHHHHHHHHHHHHHCCCCCCCC
612	EEELRKLLEKINNEEEDMRYQQQGFGKGHSA	-47.75	45.2	HHHHHHHHHHHHHHHHHHHHHHHHCCCCCCC
933	EEELRKLLEKINNEEEDMRYQQYGFGKGHSS	-47.75	45.2	HHHHHHHHHHHHHHHHHHHHHHHCCCCCCCC
900	EEELRKLLEKINNEEEDMRYQREGFGKGHSA	-47.75	45.2	HHHHHHHHHHHHHHHHHHHHHHHHHCCCCCC
709	EEELRKLLEKINNEEEDMRYQRQGFGKGHSA	-47.75	45.2	HHHHHHHHHHHHHHHHHHHHHHHHCCCCCCC
002	EEELRKLLEKINNEEEDMRYQRYGFGKGHSA	-47.75	45.2	HHHHHHHHHHHHHHHHHHHHHHHCCCCCCCC
779	EEELRKLLEKINNEQEDMRYQQQGFGKGHQS	-47.75	45.2	HHHHHHHHHHHHHHHHHHHHHHHHCCCCCCC
363	EEELRKLLEKINNEQEDMSYQQQGFGKGHSS	-47.75	45.2	HHHHHHHHHHHHHHHHHHHHHHHCCCCCCCC
285	EEELRKLLEKLNNEKEDMRYQQQGFGKGHSA	-47.75	45.2	HHHHHHHHHHHHHHHHHHHHHHHHCCCCCCC
216	EEELRKLLEKLNNEQEDMRYQRYGFGKGHSA	-47.75	45.2	HHHHHHHHHHHHHHHHHHHHHHHCCCCCCCC
912	EEEWKKLIEKINNEAEDMRYQQEGFGKGHSA	-47.75	48.4	HHHHHHHHHHHHHHHHHHHHHHHHHCCCCCC
150	EQELRKLLEKINNEEEDIRYQQQGFGKGHSS	-47.75	41.9	HHHHHHHHHHHHHHHHHHHHHHHHCCCCCCC
328	EQELRKLLEKINNEEEDMRYQQQGFGKGHQS	-47.75	41.9	HHHHHHHHHHHHHHHHHHHHHHHHCCCCCCC
520	EEELKKFLDKLNNEEEDMRYQQYGFGKGDSR	-47.66	61.3	HHHHHHHHHHHHHHHHHHHHHHHCCCCCCCC
566	EEELKKILEKFNNEYEDMFYQQKGFGKGDSR	-47.52	61.3	HHHHHHHHHHHHHHHHHHHHHHHCCCCCCCC
430	EEELKKILEKYNNEAEDIRYQQQGFGKGDSR	-47.52	58.1	HHHHHHHHHHHHHHHHHHHHHHHCCCCCCCC
263	EEELKKLLEKLNNEKEDMRYQQQGFGKGHQS	-47.51	48.4	HHHHHHHHHHHHHHHHHHHHHHHHCCCCCCC
226	EEELKKFLDKFNNEYEDRFYQQQGFGKGHSS	-47.51	61.3	HHHHHHHHHHHHHHHHHHHHHHHCCCCCCCC
069	EEELKKLLEKINNEKEDMEYQKQGFGKGHSS	-47.49	48.4	HHHHHHHHHHHHHHHHHHHHHHHCCCCCCCC
419	EEELKKFLEKFNQEEEDMRYQREGFGKGHQS	-47.48	54.8	HHHHHHHHHHHHHHHHHHHHHHHHHCCCCCC
215	EEELKKFLEKYNQEAEDMRYQQQGFGKGHSA	-47.48	54.8	HHHHHHHHHHHHHHHHHHHHHHHHCCCCCCC
159	EEELKKFLEKYNQEEEDMKYQREGKGKGHSS	-47.48	51.6	HHHHHHHHHHHHHHHHHHHHHHHCCCCCCCC
410	EEELKKFLEKYNQEEEDMRYQRQGFGKGHSS	-47.48	51.6	HHHHHHHHHHHHHHHHHHHHHHHHCCCCCCC
348	EEELKKFLEKYNQESEDMRYQQQGFGKGHSS	-47.48	51.6	HHHHHHHHHHHHHHHHHHHHHHHHCCCCCCC
895	EEELRKFLEKFNQEYEDMFYQQKGFGKGHQS	-47.48	54.8	HHHHHHHHHHHHHHHHHHHHHHHHCCCCCCC
812	EEELRKFLEKFNQEYEDMFYQQKGFGKGHSA	-47.48	54.8	HHHHHHHHHHHHHHHHHHHHHHHHCCCCCCC
126	EEELRKFLEKYNQEAEDIYYQQQGFGKGHSS	-47.48	51.6	HHHHHHHHHHHHHHHHHHHHHHHHCCCCCCC
706	EEELRKFLEKYNQESEDIRYQQQGFGKGHSA	-47.48	48.4	HHHHHHHHHHHHHHHHHHHHHHHHCCCCCCC
690	EEELRKFLEKFNNEEEDIRFQQQGFGKGKSR	-47.46	51.6	HHHHHHHHHHHCCHHHHHHHHHHHCCCCCCC
389	EEELRKILEQINNEEEDMRYQREGFGKGHSA	-47.44	41.9	HHHHHHHHHHHHHHHHHHHHHHHHHCCCCCC
915	EEELRKILEKLNNEQEDRRYQQQGFGKGHQS	-47.37	45.2	HHHHHHHHHHHHHHHHHHHHHHHHCCCCCCC
341	EEEKRKMLEKINQEAEDMRFQKEGFGKGKSR	-47.37	48.4	HHHHHHHHHHHHHHHHHHHHHHHHCCCCCCC
457	EEELKKILEQLNQEKEDMRYQREGFGKGDSR	-47.33	51.6	HHHHHHHHHHHHHHHHHHHHHHHHCCCCCCC
648	EEQLRKLLEKINNEEEDIRYQQQGFGKGHQS	-47.31	48.4	HHHHHHHHHHHHHHHHHHHHHHHCCCCCCCC
613	EEELRKLLEKINQEAEDMRYQREGFGKGDSR	-47.31	54.8	HHHHHHHHHHHHHHHHHHHHHHHHCCCCCCC

164	EEELKKLLEQINQEEEDERYQQQGFGKGHSS	-47.28	45.2	HHHHHHHHHHHHHHHHHHHHHHHHCCCCCCCC
801	EEELKKLLEQINQEEEDMRYQQQGFGKGHSA	-47.28	45.2	HHHHHHHHHHHHHHHHHHHHHHHHHCCCCCCC
919	EEELKKLLEQINQEEEDMRYQREGFGKGHSS	-47.28	45.2	HHHHHHHHHHHHHHHHHHHHHHHHHHCCCCCC
463	EEELKKLLEQLNQEKEDMRYQQQGFGKGHSA	-47.28	45.2	HHHHHHHHHHHHHHHHHHHHHHHHHCCCCCCC
631	EEELKKLLEQLNQEQEDMRYQRQGFGKGHSA	-47.28	45.2	HHHHHHHHHHHHHHHHHHHHHHHHHHCCCCCC
790	EEELRKLLEQINQEAEDMRYQKEGFGKGHSA	-47.28	45.2	HHHHHHHHHHHHHHHHHHHHHHHHHHCCCCCC
553	EEELRKLLEQINQEAEDMRYQRQGFGKGHSS	-47.28	45.2	HHHHHHHHHHHHHHHHHHHHHHHHHCCCCCCC
547	EEELRKLLEQINQEEEDMRYQKEGFGKGHSS	-47.28	41.9	HHHHHHHHHHHHHHHHHHHHHHHHHCCCCCCC
110	EEELRKLLEQINQEKEDEEYQRYGFGKGHSS	-47.28	41.9	HHHHHHHHHHHHHHHHHHHHHHHHCCCCCCCC
976	EEELRKLLEQINQEKEDMEYQRAGFGKGHSA	-47.28	41.9	HHHHHHHHHHHHHHHHHHHHHHHHHHCCCCCC
055	EEELRKLLEQLNQEKEDERYQQQGFGKGHSA	-47.28	41.9	HHHHHHHHHHHHHHHHHHHHHHHHCCCCCCCC
089	EQELRKLLEQINQEEEDMRYQREGFGKGHSS	-47.28	38.7	HHHHHHHHHHHHHHHHHHHHHHHHHHCCCCCC
228	EEELKKLLEQINNEEEDMRFQQYGFGKGKSR	-47.26	45.2	HHHHHHHHHHHHHHHHHHHHHHHHCCCCCCCC
290	EEELRKLLEQINNEQEDMFFQQKGFGKGKSR	-47.26	45.2	HHHHHHHHHHHHHHHHHHHHHHHHHCCCCCCC
157	EEELRKILEQINNEKEDMEYQRSGMGKGHQP	-47.25	45.2	HHHHHHHHHHHHHHHHHHHHHHHHHCCCCCCC
963	EEELRKLLEKINNEEEDMRYQQYGFGKGHSA	-47.23	45.2	HHHHHHHHHHHHHHHHHHHHHHHHCCCCCCCC
526	EEELRKLLEQLNNEKEDMRFQRQGFGKGKSS	-47.22	38.7	HHHHHHHHHHHHHHHHHHHHHHHHHCCCCCCC
759	EEELRKFLEQYNNDYEDMEYQRNGRGKGHSY	-47.20	41.9	HHHHHHHHHHHHCHHHHHHHHHHCCCCCCCCC
979	EEEKRKFLEKFNNEYEDMFYQQKGFGKGHSS	-47.19	54.8	HHHHHHHHHHHHHHHHHHHHHHHHHCCCCCCC
958	EEEKRKFLEKKNNEEEDMRYQRYGFGKGHSS	-47.19	48.4	HHHHHHHHHHHCHHHHHHHHHHHHCCCCCCCC
038	EEEKRKFLEKYNNESEDMRYQQQGFGKGHSS	-47.19	48.4	HHHHHHHHHHHHHHHHHHHHHHHHHCCCCCCC
021	EEEKRKFLEKYNNESEDMRYQRYGFGKGHSA	-47.19	48.4	HHHHHHHHHHHHHCHHHHHHHHHECCCCCCCC
832	EEEKRKFLEKYNNEYEDMFYQQKGFGKGHSA	-47.19	51.6	HHHHHHHHHHHHHHHHHHHHHHHHHCCCCCCC
238	EEELKKFLEKFNNEYEDMFYQKEGFGKGHQS	-47.19	58.1	HHHHHHHHHHHHHHHHHHHHHHHHHCCCCCCC
822	EEELKKFLEKFNNEYEDMFYQQKGFGKGHSA	-47.19	58.1	HHHHHHHHHHHHHHHHHHHHHHHHHCCCCCCC
922	EEELKKFLEKFNNEYEDMYYQQKGFGKGHSS	-47.19	54.8	HHHHHHHHHHHHHHHHHHHHHHHHCCCCCCCC
825	EEELKKFLEKFNNEYEDMYYQQQGFGKGHQS	-47.19	54.8	HHHHHHHHHHHHHHHHHHHHHHHHHCCCCCCC
046	EEELKKFLEKLNNEEEDMRYQQQGFGKGHSS	-47.19	51.6	HHHHHHHHHHHHHHHHHHHHHHHHHCCCCCCC
314	EEELKKFLEKYNNEAEDMRYQKEGFGKGHSA	-47.19	54.8	HHHHHHHHHHHHHHHHHHHHHHHHCCCCCCCC
302	EEELKKFLEKYNNEAEDMRYQQQGFGKGHSS	-47.19	54.8	HHHHHHHHHHHHHHHHHHHHHHHHHCCCCCCC
188	EEELKKFLEKYNNEAEDMRYQQYGFGKGHQS	-47.19	54.8	HHHHHHHHHHHHHHHHHHHHHHHHCCCCCCCC
955	EEELKKFLEKYNNEAEDMRYQREGMGKGHQP	-47.19	54.8	HHHHHHHHHHHHHHHHHHHHHHHHHHCCCCCC
045	EEELKKFLEKYNNEAEDMRYQRQGFGKGHSA	-47.19	54.8	HHHHHHHHHHHHHHHHHHHHHHHHHCCCCCCC
106	EEELKKFLEKYNNEAEDMRYQRYGFGKGHQS	-47.19	54.8	HHHHHHHHHHHHHHHHHHHHHHHHCCCCCCCC
199	EEELKKFLEKYNNEAEDMRYQRYGFGKGHSS	-47.19	54.8	HHHHHHHHHHHHHHHHHHHHHHHHCCCCCCCC
770	EEELKKFLEKYNNEEEDMRYQREGFGKGHSA	-47.19	51.6	HHHHHHHHHHHCCHHHHHHHHHHHHHCCCCCC
127	EEELKKFLEKYNNEEEDMYYQQYGFGKGHSA	-47.19	51.6	HHHHHHHHHHHCHHHHHHHHHHHHCCCCCCCC
201	EEELKKFLEKYNNESEDIRYQQQGFGKGHSS	-47.19	51.6	HHHHHHHHHHHHCHHHHHHHHHHHHCCCCCCC
599	EEELKKFLEKYNNESEDMRYQQQGFGKGHSA	-47.19	51.6	HHHHHHHHHHHHHHHHHHHHHHHHHCCCCCCC
623	EEELKKFLEKYNNESEDMRYQRYGFGKGHSS	-47.19	51.6	HHHHHHHHHHHHHHHHHHHHHHEECCCCCCCC
650	EEELKKFLEKYNNEWEDMRYQREGFGKGHSS	-47.19	51.6	HHHHHHHHHHHHHHHHHHHHHHHHHHCCCCCC
582	EEELKKFLEKYNNEYEDMEYQRTGRGKGHSF	-47.19	51.6	HHHHHHHHHHHHHHHHHHHHHHHCCCCCCCCC
679	EEELKKFLEKYNNEYEDMRYQQEGFGKGHSA	-47.19	51.6	HHHHHHHHHHHHHHHHHHHHHHHHHHCCCCCC
023	EEELRKFLEKFNNEEEDMRYQREGFGKGHQS	-47.19	51.6	HHHHHHHHHHHCHHHHHHHHHHHHHHCCCCCC
432	EEELRKFLEKFNNEEEDMRYQREGFGKGHSS	-47.19	51.6	HHHHHHHHHHHCHHHHHHHHHHHHHCCCCCCC
535	EEELRKFLEKFNNEKEDMRYQQQGFGKGHQS	-47.19	51.6	HHHHHHHHHHHHHHHHHHHHHHHHHCCCCCCC
643	EEELRKFLEKFNNEYEDKFYQQKGFGKGHQS	-47.19	54.8	HHHHHHHHHHHHHHHHHHHHHHHHCCCCCCCC
050	EEELRKFLEKFNNEYEDMFYQKEGFGKGHSS	-47.19	54.8	HHHHHHHHHHHHHHHHHHHHHHHHHCCCCCCC
367	EEELRKFLEKFNNEYEDMFYQQKGFGKGHQS	-47.19	54.8	HHHHHHHHHHHHHHHHHHHHHHHHHCCCCCCC
697	EEELRKFLEKLNNEEEDMRYQRYGFGKGHQS	-47.19	48.4	HHHHHHHHHHHHHHHHHHHHHHHHCCCCCCCC
006	EEELRKFLEKYNNEAEDIRYQQQGFGKGHSA	-47.19	51.6	HHHHHHHHHHHHHHHHHHHHHHHHHCCCCCCC
590	EEELRKFLEKYNNEAEDIRYQQQGFGKGHSS	-47.19	51.6	HHHHHHHHHHHHHHHHHHHHHHHHHCCCCCCC
083	EEELRKFLEKYNNEAEDMRYQKEGFGKGHSA	-47.19	51.6	HHHHHHHHHHHHHHHHHHHHHHHHHCCCCCCC
391	EEELRKFLEKYNNEAEDMRYQQQGFGKGHQS	-47.19	51.6	HHHHHHHHHHHHHHHHHHHHHHHHCCCCCCCC
528	EEELRKFLEKYNNEAEDMRYQQQGFGKGHSA	-47.19	51.6	HHHHHHHHHHHHHHHHHHHHHHHHCCCCCCCC
028	EEELRKFLEKYNNEAEDMRYQRQGFGKGHSA	-47.19	51.6	HHHHHHHHHHHHHHHHHHHHHHHHHCCCCCCC
615	EEELRKFLEKYNNEAEDMRYQRYGFGKGHSS	-47.19	51.6	HHHHHHHHHHHHHHHHHHHHHHHHCCCCCCCC
321	EEELRKFLEKYNNEHEDIRYQQQGFGKGHSS	-47.19	48.4	HHHHHHHHHHHHHHHHHHHHHHHHCCCCCCCC
910	EEELRKFLEKYNNESEDIRYQQQGFGKGHQS	-47.19	48.4	HHHHHHHHHHHHCHHHHHHHHHHHHCCCCCCC
220	EEELRKFLEKYNNESEDIRYQQQGFGKGHSA	-47.19	48.4	HHHHHHHHHHHHCHHHHHHHHHHHHCCCCCCC
448	EEELRKFLEKYNNESEDIRYQQQGFGKGHSS	-47.19	48.4	HHHHHHHHHHHHCHHHHHHHHHHHHCCCCCCC
589	EEELRKFLEKYNNESEDMRYQQQGFGKGHSA	-47.19	48.4	HHHHHHHHHHHHHHHHHHHHHHHHHCCCCCCC
746	EEELRKFLEKYNNEYEDMEYQRNGRGKGHSY	-47.19	48.4	HHHHHHHHHHHHHHHHHHHHHHHCCCCCCCCC
997	EEELRKFLEKYNNEYEDMFYQQKGFGKGHQS	-47.19	51.6	HHHHHHHHHHHHHHHHHHHHHHHHHCCCCCCC
120	EEELRKFLEKYNNEYEDMFYQQKGFGKGHSA	-47.19	51.6	HHHHHHHHHHHHHHHHHHHHHHHHHCCCCCCC
194	EEELRKFLEKYNNEYEDMFYQQKGFGKGHSS	-47.19	51.6	HHHHHHHHHHHHHHHHHHHHHHHHCCCCCCCC
058	EQELRKFLEKFNNEYEDKFYQQKGFGKGHSA	-47.19	51.6	HHHHHHHHHHHHHHHHHHHHHHHHCCCCCCCC

808	EQELRKFLEKFNNEYEDMFYQQKGFGKGHQS	-47.19	51.6	HHHHHHHHHHHHHHHHHHHHHHHHCCCCCCC
508	EQELRKFLEKFNNEYEDMFYQQKGFGKGHSA	-47.19	51.6	HHHHHHHHHHHHHHHHHHHHHHHHCCCCCCC
657	EQELRKFLEKFNNEYEDMFYQQKGFGKGHSS	-47.19	51.6	HHHHHHHHHHHHHHHHHHHHHHHHCCCCCCC
162	EEELKKILEKLNNEKEHMRKQRQGFGSGKQS	-47.17	38.7	HHHHHHHHHHHHHHHHHHHHHHHHHCCCCCC
725	EEELRKLLEKINNEEEDIRYQQQGFGKGHQS	-47.16	45.2	HHHHHHHHHHHHHHHHHHHHHHHHCCCCCCC
660	EEELKKILEQLNNREEDERYQRQGFGKGDSR	-47.10	48.4	HHHHHHHHHHHHHHHHHHHHHHHCCCCCCCC
775	EEELRKLLEQINNEEEDRRYQQYGFGKGHQP	-47.09	41.9	HHHHHHHHHHHHHHHHHHHHHHHCCCCCCCC
947	EEEKRKMLEKINQEAEDIRYQQQGFGKGHQA	-47.09	48.4	HHHHHHHHHHHHHHHHHHHHHHHHCCCCCCC
765	EEEKRKMLEKINQEEEDMRYQQQGFGKGHSA	-47.09	45.2	HHHHHHHHHHHHHHHHHHHHHHHHCCCCCCC
901	EEELKKFLEKFNNEYENMFKQQKGFGSGKSA	-47.07	48.4	HHHHHHHHHHHHHHHHHHHHHHHHCCCCCCC
481	EEELRKLLEQLNQEKEDMRYQQQGFGKGHQS	-47.06	41.9	HHHHHHHHHHHHHHHHHHHHHHHHCCCCCCC
015	EEELKKLLEQINQEAEDMRYQQQGFGKGHQS	-47.04	48.4	HHHHHHHHHHHHHHHHHHHHHHHHCCCCCCC
642	EEELRKILEQINNEEEDMRYQQQGFGKGDSR	-47.03	48.4	HHHHHHHHHHHHHHHHHHHHHHHCCCCCCCC
214	EEELRKILEQLNNEQEDMRYQQQGFGKGDSR	-47.03	48.4	HHHHHHHHHHHHHHHHHHHHHHHCCCCCCCC
608	EEELKKLLEKINNEAEDMRYQRQGFGKGDSR	-47.02	58.1	HHHHHHHHHHHHHHHHHHHHHHHCCCCCCCC
368	EEELRKLLEKLNNEEEDMKYQRYGFGKGDSR	-47.02	51.6	HHHHHHHHHHHHHHHHHHHHHHHCCCCCCCC
578	EEELKKLLEQINNEEEDMRYQRQGFGKGHSA	-47.00	45.2	HHHHHHHHHHHHHHHHHHHHHHHHCCCCCCC
962	EEELRKLLEQINNEAEDMRYQREGMGKGHQP	-47.00	45.2	HHHHHHHHHHHHHHHHHHHHHHHHHCCCCCC
454	EEELRKLLEQINNEEEDMRYQQQGFGKGHSA	-47.00	41.9	HHHHHHHHHHHHHHHHHHHHHHHHCCCCCCC
903	EEELRKLLEQINNEEEDMRYQQQGFGKGHSS	-47.00	41.9	HHHHHHHHHHHHHHHHHHHHHHHHCCCCCCC
640	EEELRKLLEQINNEEEDMRYQRYGFGKGHQS	-47.00	41.9	HHHHHHHHHHHHHHHHHHHHHHHCCCCCCCC
183	EEELRKLLEQINNEQEDMSYQQQGFGKGHQS	-47.00	41.9	HHHHHHHHHHHHHHHHHHHHHHHHCCCCCCC
252	EEELRKLLEQLNNEQEDMRYQREGFGKGHSA	-47.00	41.9	HHHHHHHHHHHHHHHHHHHHHHHHHCCCCCC
954	EEEKRKLLEQINNEEEDERYQREGFGKGHSS	-46.99	41.9	HHHHHHHHHHHHHHHHHHHHHHHHCCCCCCC
242	EEELKKLLEQINNEAEDERYQQQGFGKGHQS	-46.99	48.4	HHHHHHHHHHHHHHHHHHHHHHHCCCCCCCC
733	EEELKKLLEQINNEAEDMQYQRAGFGKGHSS	-46.99	48.4	HHHHHHHHHHHHHHHHHHHHHHHHHCCCCCC
203	EEELKKLLEQINNEAEDMRYQKEGFGKGHSS	-46.99	48.4	HHHHHHHHHHHHHHHHHHHHHHHHCCCCCCC
122	EEELKKLLEQINNEAEDMRYQQGGFGKGHQS	-46.99	48.4	HHHHHHHHHHHHHHHHHHHHHHCCCCCCCCC
671	EEELKKLLEQINNEAEDMRYQQQGFGKGHSA	-46.99	48.4	HHHHHHHHHHHHHHHHHHHHHHHHCCCCCCC
806	EEELKKLLEQINNEAEDMRYQREGFGKGHSS	-46.99	48.4	HHHHHHHHHHHHHHHHHHHHHHHHHCCCCCC
537	EEELKKLLEQINNEAEDMRYQRYGFGKGHSS	-46.99	48.4	HHHHHHHHHHHHHHHHHHHHHHHCCCCCCCC
737	EEELKKLLEQINNEEEDIRYQQQGFGKGHSS	-46.99	45.2	HHHHHHHHHHHHHHHHHHHHHHHHCCCCCCC
100	EEELKKLLEQINNEEEDMRYQQQGFGKGHQA	-46.99	45.2	HHHHHHHHHHHHHHHHHHHHHHHHCCCCCCC
163	EEELKKLLEQINNEEEDMRYQQQGFGKGHSA	-46.99	45.2	HHHHHHHHHHHHHHHHHHHHHHHHCCCCCCC
031	EEELKKLLEQINNEEEDMRYQQYGFGKGHSA	-46.99	45.2	HHHHHHHHHHHHHHHHHHHHHHHCCCCCCCC
700	EEELKKLLEQINNEEEDMRYQREGFGKGHSA	-46.99	45.2	HHHHHHHHHHHHHHHHHHHHHHHHHCCCCCC
493	EEELKKLLEQINNEEEDMRYQREGFGKGHSS	-46.99	45.2	HHHHHHHHHHHHHHHHHHHHHHHHHCCCCCC
593	EEELKKLLEQLNNEKEDERYQQQGFGKGHSS	-46.99	45.2	HHHHHHHHHHHHHHHHHHHHHHHCCCCCCCC
625	EEELKKLLEQLNNEKEDMRYQQQGFGKGHQS	-46.99	45.2	HHHHHHHHHHHHHHHHHHHHHHHHCCCCCCC
826	EEELKKLLEQLNNEKEDMRYQRQGFGKGHSS	-46.99	45.2	HHHHHHHHHHHHHHHHHHHHHHHHCCCCCCC
736	EEELKKLLEQLNNEQEDMRYQRYGFGKGHSS	-46.99	45.2	HHHHHHHHHHHHHHHHHHHHHHHCCCCCCCC
916	EEELRKLLEQINNEAEDIRYQQQGFGKGHSS	-46.99	45.2	HHHHHHHHHHHHHHHHHHHHHHHHCCCCCCC
653	EEELRKLLEQINNEAEDMRYQQQGFGKGHQS	-46.99	45.2	HHHHHHHHHHHHHHHHHHHHHHHHCCCCCCC
195	EEELRKLLEQINNEAEDMRYQQQGFGKGHSA	-46.99	45.2	HHHHHHHHHHHHHHHHHHHHHHHHCCCCCCC
117	EEELRKLLEQINNEAEDMRYQQQGFGKGHSS	-46.99	45.2	HHHHHHHHHHHHHHHHHHHHHHHHCCCCCCC
996	EEELRKLLEQINNEAEDMRYQQYGFGKGHSS	-46.99	45.2	HHHHHHHHHHHHHHHHHHHHHHHCCCCCCCC
620	EEELRKLLEQINNEAEDMRYQRQGFGKGHSS	-46.99	45.2	HHHHHHHHHHHHHHHHHHHHHHHHCCCCCCC
345	EEELRKLLEQINNEAEDMRYQRYGFGKGHSA	-46.99	45.2	HHHHHHHHHHHHHHHHHHHHHHHCCCCCCCC
485	EEELRKLLEQINNEAEDMRYQRYGFGKGHSS	-46.99	45.2	HHHHHHHHHHHHHHHHHHHHHHHCCCCCCCC
538	EEELRKLLEQINNEAEDMSYQKEGFGKGHSS	-46.99	45.2	HHHHHHHHHHHHHHHHHHHHHHHHCCCCCCC
427	EEELRKLLEQINNEEEDERYQQQGFGKGHSA	-46.99	41.9	HHHHHHHHHHHHHHHHHHHHHHHCCCCCCCC
890	EEELRKLLEQINNEEEDMEYQRNGFGKGHSA	-46.99	41.9	HHHHHHHHHHHHHHHHHHHHHHHHCCCCCCC
491	EEELRKLLEQINNEEEDMRYQKEGFGKGHSA	-46.99	41.9	HHHHHHHHHHHHHHHHHHHHHHHHCCCCCCC
961	EEELRKLLEQINNEEEDMRYQKEGFGKGHSS	-46.99	41.9	HHHHHHHHHHHHHHHHHHHHHHHHCCCCCCC
218	EEELRKLLEQINNEEEDMRYQQYGFGKGHSA	-46.99	41.9	HHHHHHHHHHHHHHHHHHHHHHHCCCCCCCC
834	EEELRKLLEQINNEEEDMRYQQYGFGKGHSS	-46.99	41.9	HHHHHHHHHHHHHHHHHHHHHHHCCCCCCCC
398	EEELRKLLEQINNEEEDMRYQREGFGKGHQS	-46.99	41.9	HHHHHHHHHHHHHHHHHHHHHHHHHCCCCCC
007	EEELRKLLEQINNEEEDMRYQREGFGKGHSA	-46.99	41.9	HHHHHHHHHHHHHHHHHHHHHHHHHCCCCCC
529	EEELRKLLEQINNEEEDMRYQRQGFGKGHQS	-46.99	41.9	HHHHHHHHHHHHHHHHHHHHHHHHCCCCCCC
439	EEELRKLLEQINNEEEDMRYQRQGFGKGHSA	-46.99	41.9	HHHHHHHHHHHHHHHHHHHHHHHHCCCCCCC
881	EEELRKLLEQINNEEEDQRYQQQGFGKGHSS	-46.99	41.9	HHHHHHHHHHHHHHHHHHHHHHHCCCCCCCC
757	EEELRKLLEQINNEKEDEEYQRRGMGKGHQP	-46.99	41.9	HHHHHHHHHHHHHHHHHHHHHHHHHCCCCCC
761	EEELRKLLEQINNEKEDMEYQKEGFGKGHQS	-46.99	41.9	HHHHHHHHHHHHHHHHHHHHHHHHCCCCCCC
649	EEELRKLLEQINNEKEDMRYQQQGFGKGHSA	-46.99	41.9	HHHHHHHHHHHHHHHHHHHHHHHHCCCCCCC
982	EEELRKLLEQINNEQEDMYYQQKGFGKGHQS	-46.99	41.9	HHHHHHHHHHHHHHHHHHHHHHHHCCCCCCC
699	EEELRKLLEQINNEQEDMYYQQKGFGKGHSA	-46.99	41.9	HHHHHHHHHHHHHHHHHHHHHHHHCCCCCCC
034	EEELRKLLEQLNNEEEDMKYQQQGFGKGHSA	-46.99	41.9	HHHHHHHHHHHHHHHHHHHHHHHHCCCCCCC

057	EEELRKLLEQLNNEEEDMKYQQQGFGKGHSS	-46.99	41.9	HHHHHHHHHHHHHHHHHHHHHHHHHCCCCCCCC
743	EEELRKLLEQLNNEKEDERYQQQGFGKGHSA	-46.99	41.9	HHHHHHHHHHHHHHHHHHHHHHHHHCCCCCCCC
568	EEELRKLLEQLNNEKEDERYQQQGFGKGHSS	-46.99	41.9	HHHHHHHHHHHHHHHHHHHHHHHHHCCCCCCCC
645	EEELRKLLEQLNNEKEDIRYQQQGFGKGHSS	-46.99	41.9	HHHHHHHHHHHHHHHHHHHHHHHHHCCCCCCCC
151	EEELRKLLEQLNNEKEDMRYQQQGFGKGHSS	-46.99	41.9	HHHHHHHHHHHHHHHHHHHHHHHHHHCCCCCCC
833	EEELRKLLEQLNNEKEDMRYQQYGFGKGHQS	-46.99	41.9	HHHHHHHHHHHHHHHHHHHHHHHHHCCCCCCCC
707	EEELRKLLEQLNNEKEDMRYQRQGFGKGHQS	-46.99	41.9	HHHHHHHHHHHHHHHHHHHHHHHHHHCCCCCCC
223	EEELRKLLEQLNNEKEDMRYQRQGFGKGHSA	-46.99	41.9	HHHHHHHHHHHHHHHHHHHHHHHHHHCCCCCCC
975	EEELRKLLEQLNNEKEDMRYQRYGFGKGHSA	-46.99	41.9	HHHHHHHHHHHHHHHHHHHHHHHHHCCCCCCCC
580	EEELRKLLEQLNNEQEDMRYQKEGFGKGHSA	-46.99	41.9	HHHHHHHHHHHHHHHHHHHHHHHHHHHCCCCCC
462	EEELRKLLEQLNNEQEDMRYQQQGFGKGHDP	-46.99	41.9	HHHHHHHHHHHHHHHHHHHHHHHHHHCCCCCCC
265	EEEWKKLMEQYNNESEDMRYQQQGFGKGHSS	-46.99	41.9	HHHHHHHHHHHHHHHHHHHHHHHHHHCCCCCCC
592	EEEWRKLMEQYNNEEEDMYYQQQGFGKGHSS	-46.99	38.7	HHHHHHHHHHHHHHHHHHHHHHHHHHCCCCCCC
760	EQELRKLLEQINNEKEDMEYQKQGFGKGHQS	-46.99	38.7	HHHHHHHHHHHHHHHHHHHHHHHHHHCCCCCCC
980	EEEARKMLEKINNEEEDIRYQQQGFGKGHQS	-46.98	48.4	HHHHHHHHHHHHHHHHHHHHHHHHHHCCCCCCC
239	EEELRKFLEKLNNEEEDMRYQQQGFGKGHSA	-46.92	48.4	HHHHHHHHHHHHHHHHHHHHHHHHHHCCCCCCC
415	EEELRKFLEKYNNESEDMRYQQQGFGKGHSS	-46.92	48.4	HHHHHHHHHHHHHHHHHHHHHHHHHHCCCCCCC
740	EEELRKILQKLNNEQEDMRYQQQGFGKGDSR	-46.89	51.6	HHHHHHHHHHHHHHHHHHHHHHHHHCCCCCCCC
747	EEELRKILEQLNNEKEDMRYQQQGFGKGDSR	-46.87	48.4	HHHHHHHHHHHHHHHHHHHHHHHHHCCCCCCCC
020	EEELKKLLEQINQEAEDRRYQRYGFGKGHQS	-46.87	48.4	HHHHHHHHHHHHHHHHHHHHHHHHHCCCCCCCC
715	EEELKKFLEKFNNEYEDMYYQQQGFGKGHSS	-46.86	54.8	HHHHHHHHHHHHHHHHHHHHHHHHHCCCCCCCC
236	EEELRKFLEKYNNEAEDMRYQKEGFGKGHQS	-46.86	51.6	HHHHHHHHHHHHHHHHHHHHHHHHHCCCCCCCC
490	EEELRKFLEKYNNEEEDMYYQQYGFGKGHQS	-46.86	48.4	HHHHHHHHHHHCHHHHHHHHHHHHHCCCCCCCC
750	EEELKKLLDKLNNEKEQMSYQQQGFGQGHSA	-46.80	45.2	HHHHHHHHHHHHHHHHHHHHHHHHHCCCCCCCC
326	EEELKKLLEKLNQEQEDMRYQQQGFGKGDSR	-46.80	54.8	HHHHHHHHHHHHHHHHHHHHHHHHHCCCCCCCC
777	EEELRKLLEKINQEAEDISYQQQGFGKGDSR	-46.80	54.8	HHHHHHHHHHHHHHHHHHHHHHHHHCCCCCCCC
627	EEELRKLLEKINQEEEDMRYQREGFGKGDSR	-46.80	51.6	HHHHHHHHHHHHHHHHHHHHHHHHHHCCCCCCC
536	EEELRKLLEKINQEYEDIFYQQKGFGKGDSR	-46.80	54.8	HHHHHHHHHHHHHHHHHHHHHHHHCCCCCCCCC
068	EEEKKKMLEKINNEEEDIRYQQQGFGKGHSA	-46.79	48.4	HHHHHHHHHHHHHHHHHHHHHHHHHHCCCCCCC
865	EEEKKKMLEKINNEKEDMEYQRAGFGKGHSA	-46.79	48.4	HHHHHHHHHHHHHHHHHHHHHHHHHHHCCCCCC
727	EEEKRKMLEKINNEEEDMRYQQYGFGKGHSS	-46.79	45.2	HHHHHHHHHHHHHHHHHHHHHHHHHCCCCCCCC
965	EEEKRKMLEKINNEEEDMRYQRQGFGKGHQS	-46.79	45.2	HHHHHHHHHHHHHHHHHHHHHHHHHHCCCCCCC
527	EEQLKKFLEKFNNEYEDMFYQQQGFGKGHSA	-46.75	61.3	HHHHHHHHHHHHHHHHHHHHHHHHHHCCCCCCC
991	EEELRKFLEQFNNEYEDMFKQQKGFGSGKSR	-46.74	48.4	HHHHHHHHHHHHHHHHHHHHHHHHHCCCCCCCC
989	EESLKKILEQLNNEKEDMRYQQQGFGKGHSS	-46.73	45.2	HHHHHHHHHHHHHHHHHHHHHHHHHHCCCCCCC
762	EEELKKFLEQYNQEAEDMRYQRYGFGKGHSA	-46.73	51.6	HHHHHHHHHHHHHHHHHHHHHHHHHCCCCCCCC
393	EEELKKFLEQFNQEYEDMFYQKEGFGKGHSA	-46.72	54.8	HHHHHHHHHHHHHHHHHHHHHHHHHHCCCCCCC
378	EEELKKFLEQFNQEYEDMFYQQKGFGKGHSS	-46.72	54.8	HHHHHHHHHHHHHHHHHHHHHHHHHHCCCCCCC
530	EEELKKFLEQFNQEYEDMYYQQQGFGKGHSS	-46.72	51.6	HHHHHHHHHHHHHHHHHHHHHHHHHHCCCCCCC
071	EEELKKFLEQKNQEEEDIRYQQQGFGKGHQS	-46.72	48.4	HHHHHHHHHHHHHHHHHHHHHHHHHHCCCCCCC
061	EEELKKFLEQYNQEAEDIYYQQNGFGKGHQS	-46.72	51.6	HHHHHHHHHHHHHHHHHHHHHHHHHCCCCCCCC
371	EEELKKFLEQYNQEAEDMRYQRQGFGKGHSA	-46.72	51.6	HHHHHHHHHHHHHHHHHHHHHHHHHHHCCCCCC
388	EEELKKFLEQYNQEYEDEEYQRTGRGKGHSF	-46.72	48.4	HHHHHHHHHHHHHHHHHHHHHHHHHCCCCCCCC
324	EEELRKFLEQFNQEKEDMRYQQQGFGKGHSS	-46.72	48.4	HHHHHHHHHHHHHHHHHHHHHHHHHHCCCCCCC
330	EEELRKFLEQFNQEYEDEEYQRKGFGKGHSA	-46.72	48.4	HHHHHHHHHHHHHHHHHHHHHHHHHCCCCCCCC
752	EEELRKFLEQFNQEYEDEEYQRVGRGKGHSY	-46.72	48.4	HHHHHHHHHHHHHHHHHHHHHHHHHHCCCCCCC
062	EEELRKFLEQFNQEYEDMYYQQKGFGKGHQS	-46.72	48.4	HHHHHHHHHHHHHHHHHHHHHHHHHHCCCCCCC
081	EEELRKFLEQYNQEEEDMEYQRNGRGKGHSY	-46.72	45.2	HHHHHHHHHHHHHHHHHHHHHHHHHCCCCCCCC
311	EEELRKFLEQYNQEEEDMRYQREGFGKGHSA	-46.72	45.2	HHHHHHHHHHHHHHHHHHHHHHHHHHHCCCCCC
136	EEELRKFLEQYNQESEDMRYQRYGFGKGHQS	-46.72	45.2	HHHHHHHHHHHHHHHHHHHHHHHHHCCCCCCCC
134	EEELRKFLEQYNQEYEDMEYQRTGRGKGHSF	-46.72	45.2	HHHHHHHHHHHHHHHHHHHHHHHHCCCCCCCCC
503	EEELRKFLEQYNQEYEDMQYQKEGFGKGHQA	-46.72	45.2	HHHHHHHHHHHHHHHHHHHHHHHHHHCCCCCCC
115	EEELRKFLEQYNQEYEDMRYQQEGFGKGHSA	-46.72	45.2	HHHHHHHHHHHHHHHHHHHHHHHHHHHCCCCCC
767	EEEQRKFEEQRNQEEEDMRYQQQGFGKGHSA	-46.72	41.9	HHHHHHHHHHHHHHHHHHHHHHHHHHCCCCCCC
555	EQELRKFLEQFNQEKEDMRYQQQGFGKGHQS	-46.72	45.2	HHHHHHHHHHHHHHHHHHHHHHHHHHCCCCCCC
051	EQELRKFLEQMNQEKEDMEYQRNGSGKGHSY	-46.72	41.9	HHHHHHHHHHHHHHHHHHHHHHHHHCCCCCCCC
224	EEELRKLLEQIEQEREDMEYQQKGFGKGDSR	-46.71	45.2	HHHHHHHHHHHHHHHHHHHHHHHHHCCCCCCCC
749	EEELRKFLEKYNNEAEDMRYQQQGFGKGHSS	-46.67	51.6	HHHHHHHHHHHHHHHHHHHHHHHHHHCCCCCCC
940	EEELRKFLEQYNNEEEDMSFQKEGFGKGKSA	-46.66	41.9	HHHHHHHHHHHHHHHHHHHHHHHHHHCCCCCCC
663	EEELRKLLEKINQEAEDRRYQRQGFGKGHQS	-46.65	48.4	HHHHHHHHHHHHHHHHHHHHHHHHHHCCCCCCC
256	EEELRKLLEQINNEEEQMRYQQYGFGRGHSA	-46.63	35.5	HHHHHHHHHHHCHHHHHHHHHHHHHCCCCCCCC
251	EEEKRKFLEKFNNEYEDIFYQQKGFGKGHSA	-46.59	54.8	HHHHHHHHHHHHHHHHHHHHHHHHHHCCCCCCC
886	EEEKKKWLEQLNQEQEDMQYQRAGFGKGHSS	-46.58	45.2	HHHHHHHHHHHHHHHHHHHHHHHHHHHCCCCCC
037	EEEKKKMLEQINNEAEDMRKQRYGFGSGKSR	-46.56	45.2	HHHHHHHHHHHHHHHHHHHHHHHHHCCCCCCCC
029	EEELRKFLQKYNNEAEDIRYQQQGFGKGHSA	-46.55	51.6	HHHHHHHHHHHHHHHHHHHHHHHHHHCCCCCCC
305	EEEKKKMLDQINNEAEDMRYQRQGFGKGDSR	-46.55	58.1	HHHHHHHHHHHHHHHHHHHHHHHHHCCCCCCCC
845	EEELRKLLEQINQEEEDMRYQQYGFGKGDSR	-46.54	48.4	HHHHHHHHHHHHHHHHHHHHHHHHHCCCCCCCC

017	EEELRKFLEQYNNEEEDRRYQRQGFGKGHSS	-46.53	45.2	HHHHHHHHHHHCHHHHHHHHHHHHHHCCCCCCC
569	EEELRKILEQLNNEQEDMRYQREGFGKGDSR	-46.52	48.4	HHHHHHHHHHHHHHHHHHHHHHHHHHCCCCCCC
312	EEELKKLLEKIENEREDREYQRQGFGKGHSA	-46.52	45.2	HHHHHHHHHHHHHHHHHHHHHHHHHHCCCCCCC
866	EEELKKLLEKINNEAEDMRYQQQGFGKGDSR	-46.51	58.1	HHHHHHHHHHHHHHHHHHHHHHHHHCCCCCCCC
515	EEELKKLLEKINNEQEDMSYQRYGFGKGDSR	-46.51	54.8	HHHHHHHHHHHHHHHHHHHHHHHHCCCCCCCCC
325	EEELRKLLEKINNEAEDERYQQQGFGKGDSR	-46.51	54.8	HHHHHHHHHHHHHHHHHHHHHHHHHCCCCCCCC
121	EEELRKLLEKINNEAEDMRYQKEGFGKGDSR	-46.51	54.8	HHHHHHHHHHHHHHHHHHHHHHHHHHCCCCCCC
550	EEELRKLLEKINNEAEDMRYQQQGFGKGDSR	-46.51	54.8	HHHHHHHHHHHHHHHHHHHHHHHHHCCCCCCCC
855	EQELRKLLEKLNNEKEDIRYQQQGFGKGDSR	-46.51	48.4	HHHHHHHHHHHHHHHHHHHHHHHHHCCCCCCCC
664	EEELKKFLEKYNNEAEDIYYQQNGFGKGDSR	-46.45	61.3	HHHHHHHHHHHHHHHHHHHHHHHHHCCCCCCCC
512	EEELRKFLEKFNNEYEDMFYQQKGFGKGDSR	-46.45	61.3	HHHHHHHHHHHHHHHHHHHHHHHHCCCCCCCCC
413	EEELRKFLEKYNNESEDMRYQRYGFGKGDSR	-46.45	54.8	HHHHHHHHHHHHHHHHHHHHHHHECCCCCCCCC
014	EEELKKFLEQFNNEYEDMFYQQKGFGKGHSS	-46.44	54.8	HHHHHHHHHHHHHHHHHHHHHHHHHHCCCCCCC
654	EEELKKFLEQLNNEEEDMRYQQQGFGKGHQS	-46.44	48.4	HHHHHHHHHHHHHHHHHHHHHHHHHHCCCCCCC
945	EEELRKFLEQFNNEYEDMFYQQKGFGKGHSA	-46.44	51.6	HHHHHHHHHHHHHHHHHHHHHHHHHHCCCCCCC
874	EEELRKFLEQFNNEYEDMYYQQKGFGKGHSS	-46.44	48.4	HHHHHHHHHHHHHHHHHHHHHHHHHHCCCCCCC
497	EEELRKFLEQFNNEYEDMYYQQQGFGKGHQS	-46.44	48.4	HHHHHHHHHHHHHHHHHHHHHHHHHHCCCCCCC
889	EEELRKFLEQKNNEEEDMRYQQYGFGKGHSS	-46.44	45.2	HHHHHHHHHHHHHHHHHHHHHHHHHCCCCCCCC
070	EEELKKILEQLNNEKEDMRYQQYGFGKGDSR	-46.44	51.6	HHHHHHHHHHHHHHHHHHHHHHHHHCCCCCCCC
248	EQELRKFLEQLNNDEEDRRYQQYGFGKGHSA	-46.43	38.7	HHHHHHHHHHHHHHHHHHHHHHHHHCCCCCCCC
695	EEEKRKFLEQLNNEEEDMRYQQYGFGKGHSS	-46.42	45.2	HHHHHHHHHHHHHHHHHHHHHHHHHCCCCCCCC
944	EEEKRKFLEQYNNEEEDMRYQREGFGKGHSS	-46.42	45.2	HHHHHHHHHHHCHHHHHHHHHHHHHHCCCCCCC
636	EEELKKFLEQFNNEFEDMYYQQKGFGKGHSS	-46.42	51.6	HHHHHHHHHHHHHHHHHHHHHHHHHHCCCCCCC
135	EEELKKFLEQFNNEKEDMRYQQQGFGKGHSA	-46.42	51.6	HHHHHHHHHHHHHHHHHHHHHHHHHHCCCCCCC
702	EEELKKFLEQFNNEKEDMRYQQQGFGKGHSS	-46.42	51.6	HHHHHHHHHHHHHHHHHHHHHHHHHCCCCCCCC
861	EEELKKFLEQFNNEYEDEFYQQKGFGKGHSA	-46.42	54.8	HHHHHHHHHHHHHHHHHHHHHHHHHCCCCCCCC
800	EEELKKFLEQFNNEYEDEFYQQKGFGKGHSS	-46.42	54.8	HHHHHHHHHHHHHHHHHHHHHHHHHCCCCCCCC
040	EEELKKFLEQFNNEYEDMFYQKEGFGKGHSA	-46.42	54.8	HHHHHHHHHHHHHHHHHHHHHHHHHHCCCCCCC
609	EEELKKFLEQFNNEYEDMFYQKEGFGKGHSS	-46.42	54.8	HHHHHHHHHHHHHHHHHHHHHHHHHHCCCCCCC
185	EEELKKFLEQFNNEYEDMFYQQKGFGKGHQS	-46.42	54.8	HHHHHHHHHHHHHHHHHHHHHHHHHHCCCCCCC
994	EEELKKFLEQFNNEYEDMFYQQKGFGKGHSA	-46.42	54.8	HHHHHHHHHHHHHHHHHHHHHHHHHHCCCCCCC
629	EEELKKFLEQFNNEYEDMKYQEQGFGKGHSA	-46.42	51.6	HHHHHHHHHHHHHHHHHHHHHHHHHHCCCCCCC
616	EEELKKFLEQFNNEYEDMYYQQKGFGKGHSS	-46.42	51.6	HHHHHHHHHHHHHHHHHHHHHHHHHHCCCCCCC
501	EEELKKFLEQFNNEYEDMYYQQQGFGKGHSS	-46.42	51.6	HHHHHHHHHHHHHHHHHHHHHHHHHCCCCCCCC
047	EEELKKFLEQLNNEEEDMRYQQYGFGKGHSA	-46.42	48.4	HHHHHHHHHHHHHHHHHHHHHHHHHCCCCCCCC
771	EEELKKFLEQLNNEKEDEEYQRYGFGKGHSA	-46.42	48.4	HHHHHHHHHHHHHHHHHHHHHHHHHCCCCCCCC
433	EEELKKFLEQYNNEAEDIRYQQGGFGKGHQS	-46.42	51.6	HHHHHHHHHHHHHHHHHHHHHHHHCCCCCCCCC
896	EEELKKFLEQYNNEAEDMRYQQQGFGKGHSA	-46.42	51.6	HHHHHHHHHHHHHHHHHHHHHHHHHHCCCCCCC
131	EEELKKFLEQYNNEAEDMRYQRQGFGKGHSA	-46.42	51.6	HHHHHHHHHHHHHHHHHHHHHHHHHHCCCCCCC
132	EEELKKFLEQYNNEAEDMRYQRQGFGKGHSS	-46.42	51.6	HHHHHHHHHHHHHHHHHHHHHHHHHHCCCCCCC
587	EEELKKFLEQYNNEAEDMRYQRYGFGKGHSA	-46.42	51.6	HHHHHHHHHHHHHHHHHHHHHHHHHCCCCCCCC
701	EEELKKFLEQYNNEAEDMRYQRYGFGKGHSS	-46.42	51.6	HHHHHHHHHHHHHHHHHHHHHHHHHCCCCCCCC
355	EEELKKFLEQYNNEAEDMYYQRYGFGKGHSS	-46.42	51.6	HHHHHHHHHHHHHHHHHHHHHHHHHCCCCCCCC
177	EEELKKFLEQYNNEEEDMEYQRNGFGKGHQS	-46.42	48.4	HHHHHHHHHHHCCHHHHHHHHHHHHHCCCCCCC
500	EEELKKFLEQYNNEEEDMRYQREGFGKGHSA	-46.42	48.4	HHHHHHHHHHHCHHHHHHHHHHHHHHHCCCCCC
938	EEELKKFLEQYNNESEDERYQQQGFGKGHQS	-46.42	48.4	HHHHHHHHHHHHHCCHHHHHHHHHHCCCCCCCC
956	EEELKKFLEQYNNESEDERYQQQGFGKGHSA	-46.42	48.4	HHHHHHHHHHHHHCCHHHHHHHHHHCCCCCCCC
827	EEELKKFLEQYNNESEDMRYQKEGFGKGHSA	-46.42	48.4	HHHHHHHHHHHHHHHHHHHHHHHHHHCCCCCCC
698	EEELKKFLEQYNNESEDMRYQQGGFGKGHQS	-46.42	48.4	HHHHHHHHHHHHHHHHHHHHHHHHCCCCCCCCC
386	EEELKKFLEQYNNESEDMRYQQQGFGKGHQS	-46.42	48.4	HHHHHHHHHHHHHHHHHHHHHHHHHHCCCCCCC
172	EEELKKFLEQYNNESEDMRYQQQGFGKGHSA	-46.42	48.4	HHHHHHHHHHHHHHHHHHHHHHHHHHCCCCCCC
025	EEELKKFLEQYNNESEDMRYQQQGFGKGHSS	-46.42	48.4	HHHHHHHHHHHHHHHHHHHHHHHHHHCCCCCCC
675	EEELKKFLEQYNNESEDMRYQRYGFGKGHQS	-46.42	48.4	HHHHHHHHHHHHHHHHHHHHHHHHHCCCCCCCC
016	EEELKKFLEQYNNESEDMRYQRYGFGKGHSA	-46.42	48.4	HHHHHHHHHHHHHHHHHHHHHHHHECCCCCCCC
576	EEELKKFLEQYNNEYEDEEYQRKGFGKGHSA	-46.42	48.4	HHHHHHHHHHHHHHHHHHHHHHHHHCCCCCCCC
674	EEELKKFLEQYNNEYEDEEYQRKGFGKGHSS	-46.42	48.4	HHHHHHHHHHHHHHHHHHHHHHHHHCCCCCCCC
748	EEELRKFLEQFNNEEEDERYQQQGFGKGHSS	-46.42	48.4	HHHHHHHHHHHCHHHHHHHHHHHHHCCCCCCCC
783	EEELRKFLEQFNNEYEDEFYQQKGFGKGHSA	-46.42	51.6	HHHHHHHHHHHHHHHHHHHHHHHHHCCCCCCCC
192	EEELRKFLEQFNNEYEDMFYQQKGFGKGHQS	-46.42	51.6	HHHHHHHHHHHHHHHHHHHHHHHHHHCCCCCCC
782	EEELRKFLEQFNNEYEDMYYQQKGFGKGHQS	-46.42	48.4	HHHHHHHHHHHHHHHHHHHHHHHHHHCCCCCCC
460	EEELRKFLEQFNNEYEDMYYQQKGFGKGHSA	-46.42	48.4	HHHHHHHHHHHHHHHHHHHHHHHHHHCCCCCCC
277	EEELRKFLEQFNNEYEDMYYQQNGSGKGHSY	-46.42	48.4	HHHHHHHHHHHHHHHHHHHHHHHHCCCCCCCCC
624	EEELRKFLEQFNNEYEDMYYQQQGFGKGHSA	-46.42	48.4	HHHHHHHHHHHHHHHHHHHHHHHHHHCCCCCCC
254	EEELRKFLEQLNNEEEDMRYQQQGFGKGHQA	-46.42	45.2	HHHHHHHHHHHHHHHHHHHHHHHHHHCCCCCCC
438	EEELRKFLEQLNNEEEDMRYQQQGFGKGHSA	-46.42	45.2	HHHHHHHHHHHHHHHHHHHHHHHHHHCCCCCCC
049	EEELRKFLEQLNNEEEDMRYQQYGFGKGHSA	-46.42	45.2	HHHHHHHHHHHHHHHHHHHHHHHHHCCCCCCCC
447	EEELRKFLEQLNNEEEDMRYQQYGFGKGHSS	-46.42	45.2	HHHHHHHHHHHHHHHHHHHHHHHHHCCCCCCCC

091	EEELRKFLEQYNNEAEDMRYQKEGFGKGHSA	-46.42	48.4	HHHHHHHHHHHHHHHHHHHHHHHHHCCCCCCC
175	EEELRKFLEQYNNEAEDMRYQQQGFGKGHQS	-46.42	48.4	HHHHHHHHHHHHHHHHHHHHHHHHHCCCCCCC
138	EEELRKFLEQYNNEAEDMRYQQQGFGKGHSS	-46.42	48.4	HHHHHHHHHHHHHHHHHHHHHHHHHCCCCCCC
966	EEELRKFLEQYNNEAEDMRYQRQGFGKGHSS	-46.42	48.4	HHHHHHHHHHHHHHHHHHHHHHHHHCCCCCCC
494	EEELRKFLEQYNNEEEDERYQQQGFGKGHSA	-46.42	45.2	HHHHHHHHHHHCCHHHHHHHHHHHCCCCCCCC
107	EEELRKFLEQYNNEEEDMQYQRSGMGKGHQP	-46.42	48.4	HHHHHHHHHHHCHHHHHHHHHHHHHCCCCCCC
264	EEELRKFLEQYNNEEEDMRYQREGFGKGHSA	-46.42	45.2	HHHHHHHHHHHCHHHHHHHHHHHHHHCCCCCC
349	EEELRKFLEQYNNEEEDMRYQREGFGKGHSS	-46.42	45.2	HHHHHHHHHHHCHHHHHHHHHHHHHCCCCCCC
272	EEELRKFLEQYNNEEEDMRYQREGKGKGHSH	-46.42	45.2	HHHHHHHHHHHCHHHHHHHHHHHHHCCCCCCC
974	EEELRKFLEQYNNEEEDMRYQRQGFGKGHSS	-46.42	45.2	HHHHHHHHHHHCHHHHHHHHHHHHHCCCCCCC
211	EEELRKFLEQYNNEHEDMRYQQQGFGKGHSA	-46.42	45.2	HHHHHHHHHHHHHHHHHHHHHHHHHCCCCCCC
452	EEELRKFLEQYNNESEDIRYQQQGFGKGHSS	-46.42	45.2	HHHHHHHHHHHHHHHHHHHHHHHHHCCCCCCC
858	EEELRKFLEQYNNESEDMRYQKEGFGKGHSA	-46.42	45.2	HHHHHHHHHHHHHHHHHHHHHHHHHCCCCCCC
810	EEELRKFLEQYNNESEDMRYQQGGFGKGHSF	-46.42	45.2	HHHHHHHHHHHHHHHHHHHHHHHCCCCCCCCC
067	EEELRKFLEQYNNESEDMRYQQGGFGKGHSS	-46.42	45.2	HHHHHHHHHHHHHHHHHHHHHHHCCCCCCCCC
724	EEELRKFLEQYNNESEDMRYQQQGFGKGHQS	-46.42	45.2	HHHHHHHHHHHHHHHHHHHHHHHHHCCCCCCC
431	EEELRKFLEQYNNESEDMRYQQQGFGKGHSA	-46.42	45.2	HHHHHHHHHHHHHHHHHHHHHHHHHCCCCCCC
588	EEELRKFLEQYNNESEDMRYQQQGFGKGHSS	-46.42	45.2	HHHHHHHHHHHHHHHHHHHHHHHHHCCCCCCC
381	EEELRKFLEQYNNESEDMRYQRYGFGKGHSA	-46.42	45.2	HHHHHHHHHHHHHHHHHHHHHHHECCCCCCCC
883	EEELRKFLEQYNNEYEDEEYQRKGFGKGHSA	-46.42	45.2	HHHHHHHHHHHHHHHHHHHHHHHHCCCCCCCC
842	EEELRKFLEQYNNEYEDEEYQRKGFGKGHSS	-46.42	45.2	HHHHHHHHHHHHHHHHHHHHHHHHCCCCCCCC
418	EEELRKFLEQYNNEYEDMFYQQKGFGKGHSS	-46.42	48.4	HHHHHHHHHHHHHHHHHHHHHHHHHCCCCCCC
729	EEEWKKFIEQFNNEYEDEEYQRKGFGKGHQS	-46.42	48.4	HHHHHHHHHHHHHHHHHHHHHHHHCCCCCCCC
880	EQELRKFLEQYNNEAEDMRYQQQGFGKGHSS	-46.42	45.2	HHHHHHHHHHHHHHHHHHHHHHHHHCCCCCCC
687	EQELRKFLEQYNNEEEDMRYQREGFGKGHSA	-46.42	41.9	HHHHHHHHHHHHHHHHHHHHHHHHHHCCCCCC
399	EQELRKFLEQYNNEEEDMSYQRYGFGKGHQA	-46.42	41.9	HHHHHHHHHHHHHHHHHHHHHHHHCCCCCCCC
546	EQELRKFLEQYNNESEDERYQQQGFGKGHSS	-46.42	41.9	HHHHHHHHHHHHHCHHHHHHHHHHCCCCCCCC
552	EQELRKFLEQYNNESEDMRYQRYGFGKGHQS	-46.42	41.9	HHHHHHHHHHHHHHHHHHHHHHHHCCCCCCCC
300	EEELKKILEKLNQEKEQMSYQQQGFGQGHSA	-46.40	41.9	HHHHHHHHHHHHHHHHHHHHHHHHCCCCCCCC
397	EEELKKLLEQINNEEEDMRYQQYGFGKGHSS	-46.39	45.2	HHHHHHHHHHHHHHHHHHHHHHHHCCCCCCCC
837	EEELKKLLDKINQEEEQIRYQQQGFGQGDSR	-46.38	51.6	HHHHHHHHHHHHHHHHHHHHHHHHCCCCCCCC
295	EEELKKLLEKINNEAEDRRYQQQGFGKGHSS	-46.36	51.6	HHHHHHHHHHHHHHHHHHHHHHHHCCCCCCCC
416	EEELKKLLEKINNEEEDRRYQREGFGKGHSA	-46.36	48.4	HHHHHHHHHHHHHHHHHHHHHHHHCCCCCCCC
008	EEELRKLLEKINNEQEDRSYQQQGFGKGHSA	-46.36	45.2	HHHHHHHHHHHHHHHHHHHHHHHHCCCCCCCC
056	EEEKKKMLEQINQEAEDMRYQRYGFGKGHSA	-46.32	48.4	HHHHHHHHHHHHHHHHHHHHHHHHCCCCCCCC
868	EEEKKKMLEQINQEEEDMRYQQYGFGKGHSS	-46.32	45.2	HHHHHHHHHHHHHHHHHHHHHHHHCCCCCCCC
209	EEEKKKMLEQINQEQEDMSYQQQGFGKGHSS	-46.32	45.2	HHHHHHHHHHHHHHHHHHHHHHHHCCCCCCCC
893	EEEKKKMLEQYNQESEDMRYQQQGFGKGHSA	-46.32	45.2	HHHHHHHHHHHHHHHHHHHHHHHHHCCCCCCC
936	EEEKRKMLEQINQEAEDMRYQKEGFGKGHSA	-46.32	45.2	HHHHHHHHHHHHHHHHHHHHHHHHHHCCCCCC
914	EEEKRKMLEQINQEEEDMRYQRYGFGKGHSA	-46.32	41.9	HHHHHHHHHHHHHHHHHHHHHHHHCCCCCCCC
044	EEEKRKFLDQYNNDAEQMRYQRQGFGQGHQS	-46.30	41.9	HHHHHHHHHHHHHHHHHHHHHHHHHCCCCCCC
857	EEEWKKWIEQLNNEQEDMRYQRYGFGKGHSS	-46.29	41.9	HHHHHHHHHHHHHHHHHHHHHHHHCCCCCCCC
308	EEELKKLLEQINNEAEDIRYQQQGFGKGDSR	-46.25	54.8	HHHHHHHHHHHHHHHHHHHHHHHHCCCCCCCC
197	EEELKKLLEQINNEEEDERYQQQGFGKGDSR	-46.25	51.6	HHHHHHHHHHHHHHHHHHHHHHHHCCCCCCCC
502	EEELKKLLEQINNEEEDMRYQREGFGKGDSR	-46.25	51.6	HHHHHHHHHHHHHHHHHHHHHHHHHCCCCCCC
259	EEELKKLLEQLNNEKEDMRYQQQGFGKGDSR	-46.25	51.6	HHHHHHHHHHHHHHHHHHHHHHHHCCCCCCCC
165	EEELRKLLEQINNEAEDMRYQQQGFGKGDSR	-46.25	51.6	HHHHHHHHHHHHHHHHHHHHHHHHCCCCCCCC
605	EEELKKLLEKLNNEKEDMYYQQKGFGKGDSR	-46.24	54.8	HHHHHHHHHHHHHHHHHHHHHHHHCCCCCCCC
269	EEELRKFLEKLNNEEEQIRYQQQGFGRGHSA	-46.23	41.9	HHHHHHHHHHHCHHHHHHHHHHHHHCCCCCCC
212	EEELKKFLEQFNQEYEDMRYQQEGFGKGHSA	-46.20	51.6	HHHHHHHHHHHHHHHHHHHHHHHHHHCCCCCC
105	EEELRKFLEQMNNEKEDMEYQRAGFGKGHSA	-46.18	45.2	HHHHHHHHHHHHHHHHHHHHHHHHHHCCCCCC
877	EEELRKFLEQYNNEEEDMRYQREGFGKGHQS	-46.18	45.2	HHHHHHHHHHHCHHHHHHHHHHHHHHCCCCCC
784	EEELKKILEKFNNEYEQMFYQQKGFGQGHSA	-46.11	48.4	HHHHHHHHHHHHHHHHHHHHHHHHHCCCCCCC
086	EEELKKILEKFNNEYEQMFYQQQGFGQGHSS	-46.11	48.4	HHHHHHHHHHHHHHHHHHHHHHHHHCCCCCCC
852	EEELRKILEKINNEEEQIRYQQQGFGQGHSA	-46.11	38.7	HHHHHHHHHHHCHHHHHHHHHHHHHCCCCCCC
584	EEELRKILEKINNEKEQMEYQRNGSGQGHSY	-46.11	38.7	HHHHHHHHHHHHHHHHHHHHHHHHCCCCCCCC
716	EEELRKILEKLNNEKEQMSYQQQGFGQGHSA	-46.11	38.7	HHHHHHHHHHHHHHHHHHHHHHHHCCCCCCCC
534	EQELRKILEKINNEQEQMSYQQQGFGQGHSS	-46.11	35.5	HHHHHHHHHHHHHHHHHHHHHHHHCCCCCCCC
791	EEEWRKFLEQYNNESEDMRYQQYGFGKGHSA	-46.10	45.2	HHHHHHHHHHHHHHHHHHHHHHHHCCCCCCCC
887	EEELKKFLEQYNNEAEDMRYQQQGFGKGHQA	-46.10	51.6	HHHHHHHHHHHHHHHHHHHHHHHHHCCCCCCC
315	EEEKRKFLDKYNQESEQIRYQQQGFGQGHSS	-46.07	45.2	HHHHHHHHHHHHHHHHHHHHHHHHHCCCCCCC
666	EEELRKFLEQFNNEKEQMRYQQQGFGRGHSS	-46.06	41.9	HHHHHHHHHHHHHHHHHHHHHHHHHCCCCCCC
985	EEELKKLLEQINNEAEDERYQQQGFGKGNSN	-46.05	48.4	HHHHHHHHHHHHHHHHHHHHHHHHCCCCCCCC
365	EEELRKLLEQINNEAEDERYQQQGFGKGNSN	-46.05	45.2	HHHHHHHHHHHHHHHHHHHHHHHHCCCCCCCC
995	EEELRKFLEQFNQEYEDMFYQQKGFGKGHSA	-46.05	51.6	HHHHHHHHHHHHHHHHHHHHHHHHHCCCCCCC
661	EEELKKLLEQINQEEEDMRYQQQGFGKGDSR	-46.04	51.6	HHHHHHHHHHHHHHHHHHHHHHHCCCCCCCCC
232	EEELKKLLEQLNQEKEDMRYQREGFGKGDSR	-46.04	51.6	HHHHHHHHHHHHHHHHHHHHHHHHHCCCCCCC

353	EEELRKLLEQINQEKEDEEYQRYGFGKGDSR	-46.04	48.4	HHHHHHHHHHHHHHHHHHHHHHHCCCCCCCC
506	EEEARKMLEQINNEEEDMRYQREGFGKGHSA	-46.03	45.2	HHHHHHHHHHHHHHHHHHHHHHHHHCCCCCC
347	EEEARKMLEQINNEQEDMSYQRHGFGKGHSS	-46.03	45.2	HHHHHHHHHHHHHHHHHHHHHHHCCCCCCCC
854	EEEKKKMLEQINNEAEDMRYQREGFGKGHQS	-46.03	48.4	HHHHHHHHHHHHHHHHHHHHHHHHHCCCCCC
054	EEEKKKMLEQINNEAEDMRYQRQGFGKGHSA	-46.03	48.4	HHHHHHHHHHHHHHHHHHHHHHHHHCCCCCC
310	EEEKKKMLEQINNEEEDKRYQREGVGKGHSS	-46.03	45.2	HHHHHHHHHHHHHHHHHHHHHHHHCCCCCCC
831	EEEKKKMLEQINNEEEDMRYQQQGFGKGHQS	-46.03	45.2	HHHHHHHHHHHHHHHHHHHHHHHHCCCCCCC
713	EEEKKKMLEQINNEEEDMRYQQQGFGKGHSS	-46.03	45.2	HHHHHHHHHHHHHHHHHHHHHHHHCCCCCCC
042	EEEKKKMLEQINNEEEDMRYQQYGFGKGHSA	-46.03	45.2	HHHHHHHHHHHHHHHHHHHHHHHCCCCCCCC
549	EEEKKKMLEQYNNEEEDMRYQREGFGKGHSA	-46.03	45.2	HHHHHHHHHHHCHHHHHHHHHHHHHCCCCCC
902	EEEKRKMLEQINNEEEDKRYQREGFGKGHSS	-46.03	41.9	HHHHHHHHHHHHHHHHHHHHHHHHCCCCCCC
828	EEEKKKMLEQINNREEDERYQQYGFGKGHQP	-46.01	41.9	HHHHHHHHHHHHHHHHHHHHHHHCCCCCCCC
085	EEQLRKFLEQYNNEEEDMRYQREGFGKGHQS	-45.98	48.4	HHHHHHHHHHHHHHHHHHHHHHHHHCCCCCC
449	EEQLRKFLEQYNNEEEDMRYQRQGFGKGHSA	-45.98	48.4	HHHHHHHHHHHHHHHHHHHHHHHHCCCCCCC
387	EEELRKFLEQYNQESEDMRYQRYGFGKGDSR	-45.98	51.6	HHHHHHHHHHHHHHHHHHHHHHCCCCCCCCC
179	EEELKKFLEKFNNEYEDMFYQKEGFGKGDSR	-45.94	64.5	HHHHHHHHHHHHHHHHHHHHHHHCCCCCCCC
287	EEELKKFLEKYNNEAEDIYYQQQGFGKGDSR	-45.94	61.3	HHHHHHHHHHHHHHHHHHHHHHHCCCCCCCC
802	EEELKKFLEKYNNEAEDMRYQQQGFGKGDSR	-45.94	61.3	HHHHHHHHHHHHHHHHHHHHHHHCCCCCCCC
173	EEELKKFLEKYNNESEDMRYQRYGFGKGDSR	-45.94	58.1	HHHHHHHHHHHHHHHHHHHHHECCCCCCCCC
617	EEELKKFLEKYNNEYEDMEYQRKGFGKGDSR	-45.94	58.1	HHHHHHHHHHHHHHHHHHHHHHHCCCCCCCC
181	EEELRKFLEKFNNEKEDMRYQQQGFGKGDSR	-45.94	58.1	HHHHHHHHHHHHHHHHHHHHHHHCCCCCCCC
406	EEELRKFLEKYNNEAEDIRYQQGGFGKGDSR	-45.94	58.1	HHHHHHHHHHHHHHHHHHHHHHCCCCCCCCC
283	EEELRKFLEKYNNEAEDMRYQRQGFGKGDSR	-45.94	58.1	HHHHHHHHHHHHHHHHHHHHHHHCCCCCCCC
763	EEELRKFLEKYNNESEDMRYQKEGFGKGDSR	-45.94	54.8	HHHHHHHHHHHHHHHHHHHHHHHCCCCCCCC
717	EEELRKFLEKYNNEYEDMEYQRKGFGKGDSR	-45.94	54.8	HHHHHHHHHHHHHHHHHHHHHHHCCCCCCCC
469	EEELKKFLEKYNNESEDMRYQQQGFGKGDSR	-45.93	58.1	HHHHHHHHHHHHHHHHHHHHHHHCCCCCCCC
988	EEELKKFLEKYNNEYEDMFYQQKGFGKGDSR	-45.93	61.3	HHHHHHHHHHHHHHHHHHHHHHCCCCCCCCC
614	EEEWKKFIEKYNNESEDMRYQQQGFGKGDSR	-45.93	54.8	HHHHHHHHHHHHHHHHHHHHHHHCCCCCCCC
382	EEELRKILDQINQEEEQMRYQQYGFGQGHSS	-45.93	38.7	HHHHHHHHHHHHHHHHHHHHHHHCCCCCCCC
155	EEELRKFLEKFNNEEEHKRKQREGFGSGKSA	-45.92	41.9	HHHHHHHHHHHCHHHHHHHHHHHHCCCCCCC
156	EEELRKILEKINNEQEQMSYQQQGFGQGHSA	-45.88	38.7	HHHHHHHHHHHHHHHHHHHHHHHCCCCCCCC
864	EEELRKILEKLNNEQEQMRYQRQGFGQGHSS	-45.88	38.7	HHHHHHHHHHHHHHHHHHHHHHHHCCCCCCC
543	EEEKKKMLEQIENEREDEEYQQNGMGKGHQP	-45.87	41.9	HHHHHHHHHHHHHHHHHHHHHHHCCCCCCCC
187	EEEARKMLDKINNEEEQMRYQREGFGQGHSS	-45.85	45.2	HHHHHHHHHHHHHHHHHHHHHHHHCCCCCCC
850	EEEKKKMLDKINNEAEQMRYQREGMGQGHQP	-45.85	48.4	HHHHHHHHHHHHHHHHHHHHHHHHHCCCCCC
343	EEEKRKFLEQFNNEYEDMFYQQKGFGKGHSS	-45.82	51.6	HHHHHHHHHHHHHHHHHHHHHHHHCCCCCCC
898	EEELKKFLEKYNNEAEDRRYQRYGFGKGHQS	-45.79	54.8	HHHHHHHHHHHHHHHHHHHHHHHCCCCCCCC
705	EEELKKFLEKYNNESEDRRYQQQGFGKGHSS	-45.79	51.6	HHHHHHHHHHHHHHHHHHHHHHHHCCCCCCC
429	EEELKKFLEKYNNESEDRRYQRYGFGKGHSA	-45.79	51.6	HHHHHHHHHHHHHHHHHHHHHHHCCCCCCCC
696	EEELRKFLEKFNNEYEDRFYQQKGFGKGHSS	-45.79	54.8	HHHHHHHHHHHHHHHHHHHHHHHCCCCCCCC
119	EEELRKFLEKLNNEKEDREYQRAGFGKGHSS	-45.79	48.4	HHHHHHHHHHHHHHHHHHHHHHHHCCCCCCC
153	EEELRKFLEKYNNEAEDRRYQRQGFGKGHQS	-45.79	51.6	HHHHHHHHHHHHHHHHHHHHHHHHCCCCCCC
875	EQELRKFLEKYNNEAEDRRYQRYGFGKGHSS	-45.79	48.4	HHHHHHHHHHHHHHHHHHHHHHHCCCCCCCC
776	EEEKRKMLEQFNNEKEDMRYQQQGFGKGHQA	-45.79	45.2	HHHHHHHHHHHHHHHHHHHHHHHHCCCCCCC
551	EEELRKFLQQFNNEYEDMFYQQKGFGKGHSS	-45.78	51.6	HHHHHHHHHHHHHHHHHHHHHHHHCCCCCCC
074	EEELKKLLEQINNEAEHMRKQQQGFGSGKSR	-45.76	41.9	HHHHHHHHHHHHHHHHHHHHHHHCCCCCCCC
036	EEELKKLLEQINNEAEDMRYQQQGFGKGDSR	-45.74	54.8	HHHHHHHHHHHHHHHHHHHHHHHCCCCCCCC
052	EEELKKLLEQINNEAEDMRYQRYGFGKGDSR	-45.74	54.8	HHHHHHHHHHHHHHHHHHHHHHHCCCCCCCC
200	EEELKKLLEQINNEKEDEEYQRYGFGKGDSR	-45.74	51.6	HHHHHHHHHHHHHHHHHHHHHHHCCCCCCCC
147	EEELKKLLEQLNNEKEDMRYQREGFGKGDSR	-45.74	51.6	HHHHHHHHHHHHHHHHHHHHHHHHCCCCCCC
990	EEELKKLLEQLNNEKEDMRYQRQGFGKGDSR	-45.74	51.6	HHHHHHHHHHHHHHHHHHHHHHHCCCCCCCC
769	EEELRKLLEQINNEEEDMRYQQQGFGKGDSR	-45.74	48.4	HHHHHHHHHHHHHHHHHHHHHHHCCCCCCCC
193	EEELRKLLEQINNEEEDMRYQREGFGKGDSR	-45.74	48.4	HHHHHHHHHHHHHHHHHHHHHHHHCCCCCCC
983	EEELRKLLEQINNEKEDMEYQRAGFGKGDSR	-45.74	48.4	HHHHHHHHHHHHHHHHHHHHHHHHCCCCCCC
788	EEELRKLLEQLNNEKEDMRYQQQGFGKGDSR	-45.74	48.4	HHHHHHHHHHHHHHHHHHHHHHHCCCCCCCC
774	EQELRKLLEQINNEAEDERYQQQGFGKGDSR	-45.74	48.4	HHHHHHHHHHHHHHHHHHHHHHHCCCCCCCC
208	EEEKRKFLEQYNNEAEDMRYQQYGFGKGDSR	-45.69	54.8	HHHHHHHHHHHHHHHHHHHHHHHCCCCCCCC
595	EEELKKFLEQFNNEYEDMFYQQKGFGKGDSR	-45.69	61.3	HHHHHHHHHHHHHHHHHHHHHHHCCCCCCCC
688	EEELKKFLEQFNNEYEDMYYQQQGFGKGDSR	-45.69	58.1	HHHHHHHHHHHHHHHHHHHHHHHCCCCCCCC
009	EEELKKFLEQYNNEAEDIYYQQNGFGKGDSR	-45.69	58.1	HHHHHHHHHHHHHHHHHHHHHHHCCCCCCCC
093	EEELKKFLEQYNNESEDERYQQNGFGKGDSR	-45.69	54.8	HHHHHHHHHHHHCCHHHHHHHHHCCCCCCCC
540	EEELRKFLEQFNNEYEDMFYQQKGFGKGDSR	-45.69	58.1	HHHHHHHHHHHHHHHHHHHHHHHCCCCCCCC
065	EEELRKFLEQLNNEKEDMEYQRAGFGKGDSR	-45.69	51.6	HHHHHHHHHHHHHHHHHHHHHHHHCCCCCCC
178	EEELRKFLEQYNNEEEDMRYQREGFGKGDSR	-45.69	51.6	HHHHHHHHHHHCHHHHHHHHHHHHCCCCCCC
755	EEELRKFLEQYNNEYEDMFYQQKGFGKGDSR	-45.69	54.8	HHHHHHHHHHHHHHHHHHHHHHCCCCCCCCC
805	EQELRKFLEQFNNEYEDEFYQQKGFGKGDSR	-45.69	54.8	HHHHHHHHHHHHHHHHHHHHHHHCCCCCCCC
474	EQELRKILEKLNNEKEQMSYQQQGFGQGHQS	-45.65	35.5	HHHHHHHHHHHHHHHHHHHHHHHCCCCCCCC

186	EEELKKILEQLNNEQEDMRYQRQGFGKGHSS	-45.63	45.2	HHHHHHHHHHHHHHHHHHHHHHHHHHCCCCCCC
924	EEELKKILEKLNNEKEQMRYQQQGFGQGHSA	-45.63	41.9	HHHHHHHHHHHHHHHHHHHHHHHHHHHCCCCCC
278	EEEKRKMLEQINQEEEDQRYQQQGFGKGDSR	-45.59	48.4	HHHHHHHHHHHHHHHHHHHHHHHHHHCCCCCCC
728	EEELRKFLEKFNNEYEDRFYQQKGFGKGHSA	-45.56	54.8	HHHHHHHHHHHHHHHHHHHHHHHHHHCCCCCCC
768	EEELRKFLDQYNNEEEQMRYQREGFGQGHSS	-45.54	41.9	HHHHHHHHHHHCHHHHHHHHHHHHHHCCCCCCC
823	EEELKKFLDQYNQEEEQMRFQREGFGQGKSS	-45.53	41.9	HHHHHHHHHHHHHHHHHHHHHHHHHHHCCCCCC
670	EEEARKFLDQYNNEYEQMYYQQQGFGQGHSS	-45.52	45.2	HHHHHHHHHHHHHHHHHHHHHHHHHCCCCCCCC
622	EEELRKFLDQYNNEAEQIYYQQNGFGQGHSA	-45.52	45.2	HHHHHHHHHHHHHHHHHHHHHHHHHCCCCCCCC
421	EEEARKMLQQINNEAEDRRYQREGFGKGHSA	-45.50	48.4	HHHHHHHHHHHHHHHHHHHHHHHHHHHCCCCCC
836	EEELRKFLEQYNNEEEDMRYQREGFGKGNSN	-45.48	45.2	HHHHHHHHHHHCHHHHHHHHHHHHHHCCCCCCC
921	EEELKKFLEQYNQEEEDMRYQRQGFGKGDSR	-45.47	54.8	HHHHHHHHHHHHHHHHHHHHHHHHHCCCCCCCC
532	EEELRKFLEQFNQEYEDMFYQQKGFGKGDSR	-45.47	58.1	HHHHHHHHHHHHHHHHHHHHHHHHHCCCCCCCC
558	EEELRKFLEQYNQESEDMRYQQQGFGKGDSR	-45.47	51.6	HHHHHHHHHHHHHHHHHHHHHHHHHCCCCCCCC
813	EEELRKFLEQYNQEYEDEEYQRKGFGKGDSR	-45.47	51.6	HHHHHHHHHHHHHHHHHHHHHHHHHCCCCCCCC
853	EEELKKFLDSLNNREEDMRYQRYGFGKGDSR	-45.40	54.8	HHHHHHHHHHHHHHHHHHHHHHHHHCCCCCCCC
423	EEELRKILEQINQEEEQMRYQQQGFGQGHQS	-45.40	35.5	HHHHHHHHHHHHHHHHHHHHHHHHHCCCCCCCC
539	EEELKKLLEKINQEAEQMRYQKEGFGQGHSA	-45.39	45.2	HHHHHHHHHHHHHHHHHHHHHHHHHHCCCCCCC
796	EEELKKLLEKLNQEKEQMRYQRYGFGQGHSA	-45.39	41.9	HHHHHHHHHHHHHHHHHHHHHHHHHCCCCCCCC
379	EEELRKLLEKLNQEKEQKRYQRQGFGQGHSA	-45.39	38.7	HHHHHHHHHHHHHHHHHHHHHHHHHCCCCCCCC
230	EEEWKKLMEKYNQESEQMRYQRYGFGQGHSA	-45.39	38.7	HHHHHHHHHHHHHHHHHHHHHHHHHCCCCCCCC
255	EQELRKLLEKINQEQEQKSYQQQGFGQGHQS	-45.39	35.5	HHHHHHHHHHHHHHHHHHHHHHHHHCCCCCCCC
392	EEELRKLLEKLNNEKEQMSFQQQGFGQGKSR	-45.38	38.7	HHHHHHHHHHHHHHHHHHHHHHHHHHHCCCCCC
184	EEELKKILEKINNEEEQMRYQQYGFGQGDSR	-45.38	48.4	HHHHHHHHHHHCHHHHHHHHHHHHHCCCCCCCC
694	EEELRKILEKLNNEKEQMRYQRYGFGQGDSR	-45.35	45.2	HHHHHHHHHHHHHHHHHHHHHHHHHCCCCCCCC
710	EEELRKFLEKYNQESEDRRYQRYGFGKGDSR	-45.35	54.8	HHHHHHHHHHHHHHHHHHHHHHHHCCCCCCCCC
732	EEEKRKMLEQINNEKEDMEYQRAGFGKGDSR	-45.29	48.4	HHHHHHHHHHHHHHHHHHHHHHHHHHCCCCCCC
356	EEQKRKMLEQINNEAEQMRYQRQGFGRGHSS	-45.23	41.9	HHHHHHHHHHHHHHHHHHHHHHHHHHHCCCCCC
521	EEELRKILEKINNEEEQMRYQREGFGQGHSA	-45.21	38.7	HHHHHHHHHHHCHHHHHHHHHHHHHHHCCCCCC
370	EEEKRKFLEQYNNEAEHMRKQQQGFGSGKSR	-45.19	41.9	HHHHHHHHHHHHHHHHHHHHHHHHHCCCCCCCC
712	EEEKRKFLEQYNNESEDMRYQQQGFGKGDSR	-45.18	51.6	HHHHHHHHHHHHHHHHHHHHHHHHHCCCCCCCC
453	EEELKKFLEQLNNEEEDMRYQQYGFGKGDSR	-45.18	54.8	HHHHHHHHHHHHHHHHHHHHHHHHHCCCCCCCC
484	EEELKKFLEQYNNEAEDMRYQQQGFGKGDSR	-45.18	58.1	HHHHHHHHHHHHHHHHHHHHHHHHHCCCCCCCC
486	EEELKKFLEQYNNEAEDMRYQQYGFGKGDSR	-45.18	58.1	HHHHHHHHHHHHHHHHHHHHHHHHHCCCCCCCC
087	EEELKKFLEQYNNESEDMRYQQQGFGKGDSR	-45.18	54.8	HHHHHHHHHHHHHHHHHHHHHHHHHCCCCCCCC
943	EEELKKFLEQYNNEYEDMFYQQKGFGKGDSR	-45.18	58.1	HHHHHHHHHHHHHHHHHHHHHHHHCCCCCCCCC
191	EEELRKFLEQFNNEYEDEEYQRKGFGKGDSR	-45.18	54.8	HHHHHHHHHHHHHHHHHHHHHHHHHCCCCCCCC
778	EEELRKFLEQYNNEAEDMRYQQQGFGKGDSR	-45.18	54.8	HHHHHHHHHHHHHHHHHHHHHHHHHCCCCCCCC
294	EEELRKFLEQYNNESEDERYQQQGFGKGDSR	-45.18	51.6	HHHHHHHHHHHHHCHHHHHHHHHHHCCCCCCCC
400	EEELRKFLEQYNNESEDMRYQQQGFGKGDSR	-45.18	51.6	HHHHHHHHHHHHHHHHHHHHHHHHHCCCCCCCC
084	EEELRKFLEQYNNEYEDEEYQRKGFGKGDSR	-45.18	51.6	HHHHHHHHHHHHHHHHHHHHHHHHHCCCCCCCC
280	EEELRKFLEQYNNEYEDEEYQRQGFGKGDSR	-45.18	51.6	HHHHHHHHHHHHHHHHHHHHHHHHHCCCCCCCC
253	EEELRKFLEQYNNEYEDMYYQQQGFGKGDSR	-45.18	51.6	HHHHHHHHHHHHHHHHHHHHHHHHHCCCCCCCC
794	EEELKKLLDQINQEKEQMEYQRAGFGQGDSR	-45.15	48.4	HHHHHHHHHHHHHHHHHHHHHHHHHHCCCCCCC
930	EEELKKLLDDINNRAEDMRYQKEGFGKGHSS	-45.15	48.4	HHHHHHHHHHHHHHHHHHHHHHHHHHCCCCCCC
668	EEELKKILEQFNNEYEQMFYQQKGFGQGHSA	-45.12	45.2	HHHHHHHHHHHHHHHHHHHHHHHHHHCCCCCCC
651	EEELKKILEQLNNEKEQMRYQQQGFGQGHQS	-45.12	38.7	HHHHHHHHHHHHHHHHHHHHHHHHHHCCCCCCC
137	EEELRKILEQYNNEYEQMEYQRNGRGQGHSY	-45.12	35.5	HHHHHHHHHHHHHHHHHHHHHHHHHCCCCCCCC
357	EEELKKLLEKINNEAEDRRYQRQGFGKGDSR	-45.11	58.1	HHHHHHHHHHHHHHHHHHHHHHHHHCCCCCCCC
160	EEELRKLLEKINNEAEDRRYQRYGFGKGDSR	-45.11	54.8	HHHHHHHHHHHHHHHHHHHHHHHHCCCCCCCCC
097	EEELRKLLEKINNEEEDRRYQREGFGKGDSR	-45.11	51.6	HHHHHHHHHHHHHHHHHHHHHHHHHHCCCCCCC
939	EEELKKILEQINNEEEQMRYQQYGFGQGHQS	-45.11	38.7	HHHHHHHHHHHCHHHHHHHHHHHHHCCCCCCCC
116	EEELKKILEQINNEEEQMRYQQYGFGQGHSA	-45.11	38.7	HHHHHHHHHHHCHHHHHHHHHHHHHCCCCCCCC
678	EEELRKILEQINNEKEQMEYQKQGFGQGHSA	-45.11	35.5	HHHHHHHHHHHHHHHHHHHHHHHHHHCCCCCCC
950	EEELRKILEQLNNEKEQMRYQQYGFGQGHSA	-45.11	35.5	HHHHHHHHHHHHHHHHHHHHHHHHHCCCCCCCC
144	EEEWRKILEQINNEKEQMRYQQQGFGQGHSS	-45.11	35.5	HHHHHHHHHHHHHHHHHHHHHHHHHCCCCCCCC
533	EEELRKLLEQLNNDKEQMRYQREGFGQGHQS	-45.10	32.3	HHHHHHHHHHHHHHHHHHHHHHHHHHHHCCCCC
573	EEEWKKLMEQFNNDYEQMYYQQKGFGQGHQS	-45.10	35.5	HHHHHHHHHHHHHHHHHHHHHHHHHHCCCCCCC
364	EEELKKLLEKINNEAEQIRYQQQGFGQGHSS	-45.09	45.2	HHHHHHHHHHHHHHHHHHHHHHHHHHCCCCCCC
504	EEELKKLLEKINNEAEQKRYQRQGFGQGHSA	-45.09	45.2	HHHHHHHHHHHHHHHHHHHHHHHHHCCCCCCCC
722	EEELKKLLEKINNEAEQMRYQREGMGQGHQP	-45.09	45.2	HHHHHHHHHHHHHHHHHHHHHHHHHHHCCCCCC
579	EEELKKLLEKINNEAEQMRYQRYGFGQGHSS	-45.09	45.2	HHHHHHHHHHHHHHHHHHHHHHHHHCCCCCCCC
888	EEELKKLLEKINNEAEQMSYQQQGFGQGHSA	-45.09	45.2	HHHHHHHHHHHHHHHHHHHHHHHHHCCCCCCCC
372	EEELKKLLEKINNEEEQIRYQQQGFGQGHSS	-45.09	41.9	HHHHHHHHHHHCHHHHHHHHHHHHHHCCCCCCC
519	EEELKKLLEKINNEEEQKRYQREGFGQGHSA	-45.09	41.9	HHHHHHHHHHHCHHHHHHHHHHHHHHCCCCCCC
807	EEELKKLLEKINNEEEQMRYQREGFGQGHSS	-45.09	41.9	HHHHHHHHHHHCHHHHHHHHHHHHHHCCCCCCC
876	EEELKKLLEKINNEEEQMRYQRYGFGQGHSA	-45.09	41.9	HHHHHHHHHHHCHHHHHHHHHHHHHCCCCCCCC
967	EEELKKLLEKINNEKEQMEYQRAGFGQGHQA	-45.09	41.9	HHHHHHHHHHHHHHHHHHHHHHHHHHHHCCCCC

785	EEELKKLLEKINNEKEQMEYQRRGMGQGHQP	-45.09	41.9	HHHHHHHHHHHHHHHHHHHHHHHHHCCCCCC
385	EEELKKLLEKINNEKEQMEYQRYGFGQGHSS	-45.09	41.9	HHHHHHHHHHHHHHHHHHHHHHHCCCCCCCC
260	EEELKKLLEKLNNEKEQMRYQQQGFGQGHSS	-45.09	41.9	HHHHHHHHHHHHHHHHHHHHHHHCCCCCCCC
129	EEELRKLLEKINNEAEQIRYQQQGFGQGHSS	-45.09	41.9	HHHHHHHHHHHHHHHHHHHHHHHHCCCCCCC
470	EEELRKLLEKINNEAEQMRYQQYGFGQGHSS	-45.09	41.9	HHHHHHHHHHHHHHHHHHHHHHHCCCCCCCC
322	EEELRKLLEKINNEEEQKRYQREGFGQGHSA	-45.09	38.7	HHHHHHHHHHHCHHHHHHHHHHHHCCCCCCC
464	EEELRKLLEKINNEEEQMRYQQYGFGQGHQS	-45.09	38.7	HHHHHHHHHHHCHHHHHHHHHHHCCCCCCCC
476	EEELRKLLEKINNEKEQMEYQRAGFGQGHSA	-45.09	38.7	HHHHHHHHHHHHHHHHHHHHHHHHHCCCCCC
693	EEELRKLLEKINNEQEQKSYQQQGFGQGHSA	-45.09	38.7	HHHHHHHHHHHHHHHHHHHHHHHCCCCCCCC
905	EEELRKLLEKINNEQEQKSYQQQGFGQGHSS	-45.09	38.7	HHHHHHHHHHHHHHHHHHHHHHHCCCCCCCC
934	EEELRKLLEKLNNEKEQIRYQQQGFGQGHSS	-45.09	38.7	HHHHHHHHHHHHHHHHHHHHHHHHCCCCCCC
780	EEELRKLLEKLNNEKEQMSYQQQGFGQGHQP	-45.09	38.7	HHHHHHHHHHHHHHHHHHHHHHHHCCCCCCC
222	EEELRKFLDQYNNEYEQEEYQRKGFGQGHSA	-45.05	41.9	HHHHHHHHHHHHHHHHHHHHHHHCCCCCCCC
987	EEELKKILEQLNQEKEQMRYQRQGFGQGHSA	-44.93	38.7	HHHHHHHHHHHHHHHHHHHHHHHHCCCCCCC
971	EEELRKLLEKLNNEKEQMSYQQQGFGQGHSA	-44.86	38.7	HHHHHHHHHHHHHHHHHHHHHHHCCCCCCCC
522	EEELKKFLEKYNQEAEQIYYQQQGFGQGHSS	-44.82	48.4	HHHHHHHHHHHHHHHHHHHHHHHHCCCCCCC
003	EEELKKFLEKYNQEEEQMRYQREGFGQGHSS	-44.82	45.2	HHHHHHHHHHHCHHHHHHHHHHHHCCCCCCC
309	EEELRKFLEKFNQEYEQMFYQQKGFGQGHSA	-44.82	48.4	HHHHHHHHHHHHHHHHHHHHHHHHCCCCCCC
170	EEELRKFLEKYNQEEEQMSYQRYGFGQGHSS	-44.82	41.9	HHHHHHHHHHHHHHHHHHHHHHHCCCCCCCC
369	EEELRKFLEKFNNEYEQIFFQQKGFGQGKSR	-44.82	48.4	HHHHHHHHHHHHHHHHHHHHHHHCCCCCCCC
719	EEELRKFLEKYNNEEEQMSFQKEGFGQGKSR	-44.82	41.9	HHHHHHHHHHHCHHHHHHHHHHHHCCCCCCC
471	EEELRKLLEKLNNEQEQMRYQQQGFGQGHQA	-44.80	38.7	HHHHHHHHHHHHHHHHHHHHHHHHCCCCCCC
059	EEEKKKMLEQINNEAEDMRYQQQGFGKGDSR	-44.78	54.8	HHHHHHHHHHHHHHHHHHHHHHHCCCCCCCC
772	EEEKRKMLEQINNEAEDMRYQRYGFGKGDSR	-44.78	51.6	HHHHHHHHHHHHHHHHHHHHHHHCCCCCCCC
441	EEEKRKMLEQINNEQEDEEYQRKGFGKGDSR	-44.78	48.4	HHHHHHHHHHHHHHHHHHHHHHHCCCCCCCC
141	EEELKKILEQLNNEKEQKRYQKEGFGQGHSA	-44.78	38.7	HHHHHHHHHHHHHHHHHHHHHHHHCCCCCCC
738	EEELKKILEQLNNEQEQERYQQQGFGQGHSS	-44.78	38.7	HHHHHHHHHHHHHHHHHHHHHHHCCCCCCCC
455	EEELKKLLEKINNEQEQMSYQQQGFGQGHQA	-44.77	41.9	HHHHHHHHHHHHHHHHHHHHHHHHCCCCCCC
847	EQELRKLLEKINQEEEQIRYQQQGFGQGDSR	-44.66	41.9	HHHHHHHHHHHHHHHHHHHHHHHCCCCCCCC
839	EEEARKMLEKINNEEEDMRYQREGFGKGHSA	-44.64	48.4	HHHHHHHHHHHHHHHHHHHHHHHHHCCCCCC
359	EEELKKLLEQINQEQEQMYYQQKGFGQGHQS	-44.62	38.7	HHHHHHHHHHHHHHHHHHHHHHHHCCCCCCC
219	EEELRKLLEQINQEAEQEFYQQKGFGQGHQS	-44.62	41.9	HHHHHHHHHHHHHHHHHHHHHHHCCCCCCCC
090	EEELRKLLEQINQEAEQIRYQQQGFGQGHSS	-44.62	38.7	HHHHHHHHHHHHHHHHHHHHHHHCCCCCCCC
676	EEELRKLLEQINQEEEQMRYQQQGFGQGHSS	-44.62	35.5	HHHHHHHHHHHHHHHHHHHHHHHCCCCCCCC
517	EEELRKLLEQINQEEEQMRYQQYGFGQGHSS	-44.62	35.5	HHHHHHHHHHHHHHHHHHHHHHHCCCCCCCC
358	EEELRKLLEQLNQEEEQMKYQRYGFGQGHSS	-44.62	35.5	HHHHHHHHHHHHHHHHHHHHHHHCCCCCCCC
745	EEELKKLLEKINNEAEQIRYQQQGFGQGHSA	-44.62	45.2	HHHHHHHHHHHHHHHHHHHHHHHHCCCCCCC
405	EEELKKLLEKLNNEQEQMRYQQNGFGQGHQS	-44.62	41.9	HHHHHHHHHHHHHHHHHHHHHHHHCCCCCCC
146	EEELRKLLEKINNEEEQMFYQQKGFGQGHSS	-44.62	41.9	HHHHHHHHHHHHHHHHHHHHHHHHCCCCCCC
111	EEELRKLLEKINNEKEQMEYQKQGFGQGHSS	-44.62	38.7	HHHHHHHHHHHHHHHHHHHHHHHCCCCCCCC
870	EEELRKLLEKLNNEKEQMRYQQYGFGQGHSA	-44.62	38.7	HHHHHHHHHHHHHHHHHHHHHHHCCCCCCCC
394	EEELKKLLEQINNEEEQMRFQQQGFGQGKSR	-44.62	38.7	HHHHHHHHHHHCHHHHHHHHHHHHCCCCCCC
018	EEEKRKFLDQFNQEYEQMFYQQKGFGQGDSR	-44.59	54.8	HHHHHHHHHHHHHHHHHHHHHHHCCCCCCCC
509	EEELRKILEQINNEAEQMFYQQKGFGQGHSS	-44.59	41.9	HHHHHHHHHHHHHHHHHHHHHHHHCCCCCCC
499	EEELRRLLEQINNEAEDMRYQQQGFGKGHQS	-44.58	45.2	HHHHHHHHHHHHHHHHHHHHHHHHCCCCCCC
773	EQELRKFLEKFNNEYEDRFYQQKGFGKGDSR	-44.55	58.1	HHHHHHHHHHHHHHHHHHHHHHHCCCCCCCC
565	EEELKKFLEKFNNEEEQMSYQRQGFGQGHSA	-44.53	48.4	HHHHHHHHHHHCHHHHHHHHHHHCCCCCCCC
221	EEELKKFLEKFNNEYEQMYYQQKGFGQGHSS	-44.53	48.4	HHHHHHHHHHHHHHHHHHHHHHHHCCCCCCC
189	EEELKKFLEKYNNEAEQIRYQQQGFGQGHSA	-44.53	48.4	HHHHHHHHHHHHHHHHHHHHHHHHCCCCCCC
744	EEELKKFLEKYNNEAEQKRYQQQGFGQGHSS	-44.53	48.4	HHHHHHHHHHHHHHHHHHHHHHHCCCCCCCC
918	EEELKKFLEKYNNEAEQMRYQQQGFGQGHSS	-44.53	48.4	HHHHHHHHHHHHHHHHHHHHHHHCCCCCCCC
408	EEELKKFLEKYNNEEEQMSYQKEGFGQGHSA	-44.53	45.2	HHHHHHHHHHHCHHHHHHHHHHHHCCCCCCC
296	EEELKKFLEKYNNEYEQKFYQQKGFGQGHQS	-44.53	48.4	HHHHHHHHHHHHHHHHHHHHHHHCCCCCCCC
012	EEELKKFLEKYNNEYEQMEYQRTGRGQGHSF	-44.53	45.2	HHHHHHHHHHHHHHHHHHHHHHHCCCCCCCC
984	EEELKKFLEKYNNEYEQMQYQKEGFGQGHSS	-44.53	45.2	HHHHHHHHHHHHHHHHHHHHHHHHCCCCCCC
245	EEELRKFLEKFNNEYEQMFYQQKGFGQGHSA	-44.53	48.4	HHHHHHHHHHHHHHHHHHHHHHHHCCCCCCC
510	EEELRKFLEKFNNEYEQMFYQQKGFGQGHSS	-44.53	48.4	HHHHHHHHHHHHHHHHHHHHHHHHCCCCCCC
004	EEELRKFLEKFNNEYEQMYYQQQGFGQGHSA	-44.53	45.2	HHHHHHHHHHHHHHHHHHHHHHHCCCCCCCC
033	EEELRKFLEKYNNEAEQIRYQQQGMGQGHQP	-44.53	45.2	HHHHHHHHHHHHHHHHHHHHHHHHCCCCCCC
247	EEELRKFLEKYNNEAEQMRYQKEGFGQGHQS	-44.53	45.2	HHHHHHHHHHHHHHHHHHHHHHHHCCCCCCC
456	EEELRKFLEKYNNEAEQMRYQQQGFGQGHQS	-44.53	45.2	HHHHHHHHHHHHHHHHHHHHHHHCCCCCCCC
149	EEELRKFLEKYNNEAEQMRYQRQGFGQGHSA	-44.53	45.2	HHHHHHHHHHHHHHHHHHHHHHHHCCCCCCC
473	EEELRKFLEKYNNEEEQMRYQREGFGQGHQS	-44.53	41.9	HHHHHHHHHHHCCHHHHHHHHHHHHCCCCCC
814	EEELRKFLEKYNNEEEQMRYQREGKGQGHSH	-44.53	41.9	HHHHHHHHHHHCCHHHHHHHHHHHCCCCCCC
266	EEELRKFLEKYNNEEEQMRYQRQGFGQGHSS	-44.53	41.9	HHHHHHHHHHHCCHHHHHHHHHHHCCCCCCC
626	EEELRKFLEKYNNESEQIRYQQQGFGQGHSA	-44.53	41.9	HHHHHHHHHHHHHHHHHHHHHHHHCCCCCCC
227	EEELRKFLEKYNNESEQMRYQQQGFGQGHSS	-44.53	41.9	HHHHHHHHHHHHHHHHHHHHHHHHCCCCCCC

867	EEELRKFLEKYNNEYEQMEYQRKGFGQGHQS	-44.53	41.9	HHHHHHHHHHHHHHHHHHHHHHHHHCCCCCC
380	EEELRKLLEKINQEEEQMRYQQQGFGQGHSS	-44.49	38.7	HHHHHHHHHHHHHHHHHHHHHHHHCCCCCCC
011	EEEKKKMLEKINNEEEQMRFQQYGFGQGKSR	-44.43	41.9	HHHHHHHHHHHCHHHHHHHHHHHHCCCCCCC
703	EEELRKILEQINNREEQERYQQYGFGQGDSR	-44.41	38.7	HHHHHHHHHHHHHHHHHHHHHHHHCCCCCCC
479	EEQLRKFLEKYNQEEEQMRYQREGFGQGHQS	-44.38	45.2	HHHHHHHHHHHHHHHHHHHHHHHHHHCCCCC
333	EEELKKLLEKINNEAEQMSYQRHGFGQGHSS	-44.36	45.2	HHHHHHHHHHHHHHHHHHHHHHHHCCCCCCC
518	EEELKKLLEKINNEKEQMEYQKQGFGQGDSR	-44.36	48.4	HHHHHHHHHHHHHHHHHHHHHHHHCCCCCCC
477	EEELRKLLEKINNEEEQMRYQQQGFGQGDSR	-44.36	45.2	HHHHHHHHHHHCHHHHHHHHHHHHCCCCCCC
217	EEELRKLLEKINNEEEQMRYQRYGFGQGDSR	-44.36	45.2	HHHHHHHHHHHCHHHHHHHHHHHHCCCCCCC
586	EEELRKFLEKFNQEEEQMSYQRHGFGQGHSS	-44.35	45.2	HHHHHHHHHHHHHHHHHHHHHHHHCCCCCCC
291	EEELKKLLEQINNEAEQERYQQQGFGQGHQS	-44.32	41.9	HHHHHHHHHHHHHHHHHHHHHHHHCCCCCCC
894	EEELKKLLEQINNEAEQERYQQQGFGQGHSS	-44.32	41.9	HHHHHHHHHHHHHHHHHHHHHHHHCCCCCCC
246	EEELKKLLEQINNEAEQMRYQQQGFGQGHSA	-44.32	41.9	HHHHHHHHHHHHHHHHHHHHHHHHCCCCCCC
925	EEELKKLLEQINNEAEQMRYQRYGFGQGHSA	-44.32	41.9	HHHHHHHHHHHHHHHHHHHHHHHHCCCCCCC
929	EEELKKLLEQINNEAEQMRYQRYGFGQGHSS	-44.32	41.9	HHHHHHHHHHHHHHHHHHHHHHHHCCCCCCC
354	EEELKKLLEQINNEEEQMRYQQYGFGQGHSS	-44.32	38.7	HHHHHHHHHHHCHHHHHHHHHHHHCCCCCCC
680	EEELKKLLEQINNEEEQMRYQREGFGQGHSA	-44.32	38.7	HHHHHHHHHHHCHHHHHHHHHHHHHHCCCCC
213	EEELKKLLEQINNEQEQESYQQQGFGQGHQS	-44.32	38.7	HHHHHHHHHHHHHHHHHHHHHHHHCCCCCCC
754	EEELKKLLEQINNEQEQMSYQQQGFGQGHSS	-44.32	38.7	HHHHHHHHHHHHHHHHHHHHHHHHCCCCCCC
489	EEELKKLLEQLNNEKEQMRYQQQGFGQGHQA	-44.32	38.7	HHHHHHHHHHHHHHHHHHHHHHHHHCCCCCC
981	EEELKKLLEQLNNEKEQMRYQRQGFGQGHSA	-44.32	38.7	HHHHHHHHHHHHHHHHHHHHHHHHHCCCCCC
331	EEELKKLLEQLNNEKEQMRYQRYGFGQGHQS	-44.32	38.7	HHHHHHHHHHHHHHHHHHHHHHHHCCCCCCC
030	EEELRKLLEQINNEAEQMRYQQQGFGQGHSA	-44.32	38.7	HHHHHHHHHHHHHHHHHHHHHHHHCCCCCCC
306	EEELRKLLEQINNEAEQMRYQRYGFGQGHQS	-44.32	38.7	HHHHHHHHHHHHHHHHHHHHHHHHCCCCCCC
161	EEELRKLLEQINNEEEQERYQQQGFGQGHSA	-44.32	35.5	HHHHHHHHHHHCHHHHHHHHHHHHCCCCCCC
993	EEELRKLLEQINNEEEQERYQQQGFGQGHSS	-44.32	35.5	HHHHHHHHHHHCHHHHHHHHHHHHCCCCCCC
297	EEELRKLLEQINNEEEQMRYQQQGFGQGHQS	-44.32	35.5	HHHHHHHHHHHCHHHHHHHHHHHHCCCCCCC
741	EEELRKLLEQINNEEEQMRYQQQGFGQGHSA	-44.32	35.5	HHHHHHHHHHHCHHHHHHHHHHHHCCCCCCC
891	EEELRKLLEQINNEEEQMRYQQQGFGQGHSP	-44.32	35.5	HHHHHHHHHHHCHHHHHHHHHHHHHCCCCCC
053	EEELRKLLEQINNEEEQMRYQQQGFGQGHSS	-44.32	35.5	HHHHHHHHHHHCHHHHHHHHHHHHCCCCCCC
307	EEELRKLLEQINNEEEQMRYQQYGFGQGHQS	-44.32	35.5	HHHHHHHHHHHCHHHHHHHHHHHHCCCCCCC
304	EEELRKLLEQINNEEEQMRYQQYGFGQGHSA	-44.32	35.5	HHHHHHHHHHHCHHHHHHHHHHHHCCCCCCC
821	EEELRKLLEQINNEEEQMRYQQYGFGQGHSS	-44.32	35.5	HHHHHHHHHHHCHHHHHHHHHHHHCCCCCCC
352	EEELRKLLEQINNEEEQMRYQREGFGQGHQS	-44.32	35.5	HHHHHHHHHHHCHHHHHHHHHHHHHHCCCCC
766	EEELRKLLEQINNEKEQEEYQRKGFGQGHSS	-44.32	35.5	HHHHHHHHHHHHHHHHHHHHHHHHCCCCCCC
492	EEELRKLLEQINNEQEQMSYQQQGFGQGHQS	-44.32	35.5	HHHHHHHHHHHHHHHHHHHHHHHHCCCCCCC
633	EEELRKLLEQLNNEKEQMRYQRQGFGQGHSA	-44.32	35.5	HHHHHHHHHHHHHHHHHHHHHHHHHCCCCCC
288	EEELRKLLEQLNNEKEQMRYQRYGFGQGHSS	-44.32	35.5	HHHHHHHHHHHHHHHHHHHHHHHHCCCCCCC
340	EEELKKFLDQYNNESEQMRYQQQGFGQGDSR	-44.30	51.6	HHHHHHHHHHHHHHHHHHHHHHHHCCCCCCC
268	EEELRKFLEKYNNEEEQMRYQREGFGQGHSA	-44.30	41.9	HHHHHHHHHHHCCHHHHHHHHHHHHHCCCCC
641	EEELKKLLEQINQEEEQMRYQQYGFGQGHSA	-44.30	38.7	HHHHHHHHHHHHHHHHHHHHHHHHCCCCCCC
899	EEELKKFLDQLNNEKEQMEYQRAGFGQGDSR	-44.29	51.6	HHHHHHHHHHHHHHHHHHHHHHHHHCCCCCC
856	EEELRKFLEQYNQEYEQMEYQRQGFGQGSSY	-44.27	38.7	HHHHHHHHHHHHHHHHHHHHHHHHHCCCCCC
063	EQELRKFLEKFNNEEEQMRYQREGFGQGHSS	-44.24	41.9	HHHHHHHHHHHCHHHHHHHHHHHHHCCCCCC
276	EEELRKLLEKINNEAEQMRYQREGFGQGHSA	-44.21	41.9	HHHHHHHHHHHHHHHHHHHHHHHHHHCCCCC
873	EEELRKILEKYNNEEEQMRYQQQGFGQGDSR	-44.20	45.2	HHHHHHHHHHHCCHHHHHHHHHHHCCCCCCC
862	EEELKKFLEKYNNEAEEMRKQQQGFGSGKSR	-44.14	48.4	HHHHHHHHHHHHHHHHHHHHHHHHCCCCCCC
142	EEEARKMLEKINNEAEQMRYQQQGFGQGHQS	-44.13	45.2	HHHHHHHHHHHHHHHHHHHHHHHHCCCCCCC
669	EEEKKKMLEKINNEAEQIRYQQQGFGQGHSS	-44.13	45.2	HHHHHHHHHHHHHHHHHHHHHHHHHCCCCCC
206	EEEKKKMLEKINNEAEQMRYQQQGFGQGHSA	-44.13	45.2	HHHHHHHHHHHHHHHHHHHHHHHHCCCCCCC
632	EEEKKKMLEKINNEEEQERYQQQGFGQGHSA	-44.13	41.9	HHHHHHHHHHHCHHHHHHHHHHHHCCCCCCC
414	EEEKKKMLEKINNEQEQMSYQQQGFGQGHQS	-44.13	41.9	HHHHHHHHHHHHHHHHHHHHHHHHCCCCCCC
190	EEEKRKMLEKINNEAEQIRYQQNGFGQGHSS	-44.13	41.9	HHHHHHHHHHHHHHHHHHHHHHHHCCCCCCC
554	EEEKRKMLEKINNEEEQMRYQQYGFGQGHSS	-44.13	38.7	HHHHHHHHHHHCHHHHHHHHHHHHCCCCCCC
560	EEELKKFLEKYNQEAEQMRYQRQGFGQGDSR	-44.09	54.8	HHHHHHHHHHHHHHHHHHHHHHHHCCCCCCC
112	EEELRKFLEKYNQEAEQIRYQQQGFGQGDSR	-44.09	51.6	HHHHHHHHHHHHHHHHHHHHHHHHCCCCCCC
819	EEELRKFLEKYNQEAEQMRYQKEGFGQGDSR	-44.09	51.6	HHHHHHHHHHHHHHHHHHHHHHHHHCCCCCC
878	EEQLKKFLEKMNNEKEQMEYQRAGFGQGHSS	-44.09	48.4	HHHHHHHHHHHHHHHHHHHHHHHHHHCCCCC
360	EEELRKLLEQINNEEEQMRYQRYGFGQGHSA	-44.06	35.5	HHHHHHHHHHHCHHHHHHHHHHHHCCCCCCC
718	EEELKKFLEQFNQEYEQMFYQQQGFGQGHSS	-44.06	48.4	HHHHHHHHHHHHHHHHHHHHHHHHHCCCCCC
952	EEELKKFLEQYNQEAEQMRYQQQGFGQGHSA	-44.06	45.2	HHHHHHHHHHHHHHHHHHHHHHHHCCCCCCC
964	EEELRKFLEQMNQEKEQMEYQRSGFGQGHSY	-44.06	41.9	HHHHHHHHHHHHHHHHHHHHHHHHHHCCCCC
835	EEEKRKFLEKYNNEEEQMRYQREGFGQGHSS	-44.05	41.9	HHHHHHHHHHHCCHHHHHHHHHHHHCCCCCC
742	EEELRKFLEKYNNESEQMRYQQQGFGQGHSA	-44.05	41.9	HHHHHHHHHHHHHHHHHHHHHHHHHCCCCCC
647	EEELKKLLDQLNNEKEQMRYQQQGFGQGHSS	-43.96	41.9	HHHHHHHHHHHHHHHHHHHHHHHHCCCCCCC
926	EEELKKFLQKFNNEYEDRFYQKEGFGKGDSR	-43.91	64.5	HHHHHHHHHHHHHHHHHHHHHHHHCCCCCCC
292	EEELRKFLEAFNNRYEDMYYQQQGFGKGDSR	-43.91	51.6	HHHHHHHHHHHHHHHHHHHHHHHHCCCCCCC

336	EEEKKKMLDQINNEAEQMRYQRYGFGQGDSR	-43.89	51.6	HHHHHHHHHHHHHHHHHHHHHHHHCCCCCCCC
275	EEELKKFLQKYNNEEEQMSYQQNGSGQGHSY	-43.89	45.2	HHHHHHHHHHHHHHHHHHHHHHHHCCCCCCCC
523	EEELKKILEQINNEEEQMRYQREGFGQGDSR	-43.87	45.2	HHHHHHHHHHCHHHHHHHHHHHHHCCCCCCCC
338	EEELRKILEQLNNEKEQMRYQRYGFGQGDSR	-43.87	41.9	HHHHHHHHHHHHHHHHHHHHHHHHCCCCCCCC
005	EEELKKLLEKINNEAEQIRYQQQGFGQGDSR	-43.85	51.6	HHHHHHHHHHHHHHHHHHHHHHHHCCCCCCCC
424	EEELKKLLEKINNEEEQMRYQQYGFGQGDSR	-43.85	48.4	HHHHHHHHHHCHHHHHHHHHHHHHCCCCCCCC
130	EEELRKLLEKINNEKEQMEYQRYGFGQGDSR	-43.85	45.2	HHHHHHHHHHHHHHHHHHHHHHHHCCCCCCCC
279	EEELRKLLEKLNNEKEQMRYQRQGFGQGDSR	-43.85	45.2	HHHHHHHHHHHHHHHHHHHHHHHHCCCCCCCC
764	EEELKKLLEQINNEEEQMRYQQYGFGQGHSA	-43.85	38.7	HHHHHHHHHHCHHHHHHHHHHHHHCCCCCCCC
909	EEELKKFLEKYNNEEEQMRYQREGFGQGDSR	-43.80	51.6	HHHHHHHHHHCCHHHHHHHHHHHHCCCCCCCC
099	EEEWKKILEQLNNEQEQMRYQRYGFGQGDSR	-43.78	45.2	HHHHHHHHHHHHHHHHHHHHHHHHCCCCCCCC
789	EEELKKFLEQYNNEAEQMRYQRYGFGQGHSA	-43.78	45.2	HHHHHHHHHHHHHHHHHHHHHHHHCCCCCCCC
992	EEELKKFLEQYNNEHEQMRYQQQGFGQGHQS	-43.78	41.9	HHHHHHHHHHHHHHHHHHHHHHHHHCCCCCCC
884	EEEKRKFLEQYNNEAEQMRYQREGKGQGHSH	-43.76	41.9	HHHHHHHHHHHHHHHHHHHHHHHHHCCCCCCC
844	EEEKRKFLEQYNNEEEQMRYQREGFGQGHQS	-43.76	38.7	HHHHHHHHHHCHHHHHHHHHHHHHHHCCCCCC
659	EEEKRKFLEQYNNEEEQMRYQREGFGQGHSS	-43.76	38.7	HHHHHHHHHHCHHHHHHHHHHHHHHHCCCCCC
786	EEELKKFLEQFNNEAEQMQYQRGGFGQGHQP	-43.76	48.4	HHHHHHHHHHHHHHHHHHHHHHHHHCCCCCCC
820	EEELKKFLEQFNNEYEQMFYQQKGFGQGHQS	-43.76	48.4	HHHHHHHHHHHHHHHHHHHHHHHHHCCCCCCC
273	EEELKKFLEQLNNEKEQEEYQRYGFGQGHQS	-43.76	41.9	HHHHHHHHHHHHHHHHHHHHHHHHCCCCCCCC
249	EEELKKFLEQYNNEAEQMRYQKEGFGQGHSS	-43.76	45.2	HHHHHHHHHHHHHHHHHHHHHHHHHCCCCCCC
606	EEELKKFLEQYNNEAEQMRYQQQGFGQGHSS	-43.76	45.2	HHHHHHHHHHHHHHHHHHHHHHHHHCCCCCCC
948	EEELKKFLEQYNNEEEQERYQQQGFGQGHSA	-43.76	41.9	HHHHHHHHHHCCHHHHHHHHHHHHCCCCCCCC
298	EEELKKFLEQYNNEEEQMRYQREGFGQGHQS	-43.76	41.9	HHHHHHHHHHCHHHHHHHHHHHHHHHCCCCCC
960	EEELKKFLEQYNNEEEQMRYQREGFGQGHSA	-43.76	41.9	HHHHHHHHHHCHHHHHHHHHHHHHHHCCCCCC
840	EEELKKFLEQYNNESEQIRYQQQGFGQGHSS	-43.76	41.9	HHHHHHHHHHHHHHHHHHHHHHHHHCCCCCCC
384	EEELKKFLEQYNNESEQMRYQKEGFGQGHSS	-43.76	41.9	HHHHHHHHHHHHHHHHHHHHHHHHHCCCCCCC
282	EEELKKFLEQYNNESEQMRYQQNGSGQGHSY	-43.76	41.9	HHHHHHHHHHHHHHHHHHHHHHHCCCCCCCCC
927	EEELKKFLEQYNNESEQMRYQQQGFGQGHSA	-43.76	41.9	HHHHHHHHHHHHHHHHHHHHHHHHHCCCCCCC
024	EEELKKFLEQYNNESEQMRYQRYGFGQGHSA	-43.76	41.9	HHHHHHHHHHHHHHHHHHHHHHHHCCCCCCCC
404	EEELKKFLEQYNNEYEQMQYQKEGFGQGHSA	-43.76	41.9	HHHHHHHHHHHHHHHHHHHHHHHHHCCCCCCC
731	EEELRKFLEQFNNEQEQMSYQQQGFGQGHSS	-43.76	41.9	HHHHHHHHHHHHHHHHHHHHHHHHCCCCCCCC
468	EEELRKFLEQLNNEEEQMRYQQYGFGQGHQS	-43.76	38.7	HHHHHHHHHHHHHHHHHHHHHHHHCCCCCCCC
758	EEELRKFLEQYNNEAEQMRYQQQGFGQGHQS	-43.76	41.9	HHHHHHHHHHHHHHHHHHHHHHHHHCCCCCCC
409	EEELRKFLEQYNNEAEQMRYQQQGFGQGHSA	-43.76	41.9	HHHHHHHHHHHHHHHHHHHHHHHHHCCCCCCC
420	EEELRKFLEQYNNEAEQMRYQRQGFGQGHQS	-43.76	41.9	HHHHHHHHHHHHHHHHHHHHHHHHHCCCCCCC
210	EEELRKFLEQYNNEAEQMRYQRQGFGQGHSA	-43.76	41.9	HHHHHHHHHHHHHHHHHHHHHHHHHCCCCCCC
730	EEELRKFLEQYNNEEEQMRYQREGFGQGHSS	-43.76	38.7	HHHHHHHHHHCHHHHHHHHHHHHHHHCCCCCC
906	EEELRKFLEQYNNEHEQMRYQQQGFGQGHSS	-43.76	38.7	HHHHHHHHHHHHHHHHHHHHHHHHHHCCCCCC
229	EEELRKFLEQYNNESEQERYQRKGFGQGHSS	-43.76	38.7	HHHHHHHHHHCCHHHHHHHHHHHHCCCCCCCC
829	EEELRKFLEQYNNESEQMRYQQQGFGQGHSA	-43.76	38.7	HHHHHHHHHHHHHHHHHHHHHHHHHCCCCCCC
913	EEELRKFLEQYNNESEQMRYQQQGFGQGHSS	-43.76	38.7	HHHHHHHHHHHHHHHHHHHHHHHHHCCCCCCC
885	EEELRKFLEQYNNEYEQEEYQRKGFGQGHSS	-43.76	38.7	HHHHHHHHHHHHHHHHHHHHHHHHCCCCCCCC
959	EEELRKFLEQYNNEYEQMFYQQKGFGQGHSS	-43.76	41.9	HHHHHHHHHHHHHHHHHHHHHHHHHCCCCCCC
475	EEELRKFLEQYNNEYEQMYYQQKGFGQGHSS	-43.76	38.7	HHHHHHHHHHHHHHHHHHHHHHHHHCCCCCCC
618	EQELRKFLEQYNNESEQMRYQRYGFGQGHSS	-43.76	35.5	HHHHHHHHHHHHHHHHHHHHHHHHCCCCCCCC
148	EEELRKFLEQYNNRAEQERYQQQGFGQGHSA	-43.74	38.7	HHHHHHHHHHHHHHHHHHHHHHHHCCCCCCCC
953	EEEKKKMLEQINQEEEQMRYQQQGFGQGHSA	-43.66	38.7	HHHHHHHHHHHHHHHHHHHHHHHHCCCCCCCC
818	EEEKKKMLEQYNQEEEQMRYQREGFGQGHSA	-43.66	38.7	HHHHHHHHHHHHHHHHHHHHHHHHHHCCCCCC
531	EEEKKKMLEKINNEEEQIRYQQQGFGQGHSS	-43.66	41.9	HHHHHHHHHHCHHHHHHHHHHHHHHHCCCCCC
908	EEEKKKMLEKINNEEEQMRYQRYGFGQGHSA	-43.66	41.9	HHHHHHHHHHCHHHHHHHHHHHHHCCCCCCCC
482	EEELRKFLEKYNNESEQMRYQRYGFGQGHSA	-43.63	41.9	HHHHHHHHHHHHHHHHHHHHHHHHCCCCCCCC
986	EEEKRKFLDQMNNEKEQMEYQRAGFGQGDSR	-43.62	48.4	HHHHHHHHHHHHHHHHHHHHHHHHHCCCCCCC
443	EEELRKFLEQFNQEYEQMFYQQKGFGQGHSA	-43.60	45.2	HHHHHHHHHHHHHHHHHHHHHHHHHCCCCCCC
545	EEELRKILDDLNNRKEQMRYQRYGFGQGHSA	-43.58	35.5	HHHHHHHHHHHHHHHHHHHHHHHHCCCCCCCC
721	EEEKRKFLEQFNQEYEQMFYQQKGFGQGHSA	-43.58	45.2	HHHHHHHHHHHHHHHHHHHHHHHHHCCCCCCC
440	EEELKKFLEQYNQESEQMRYQQQGFGQGHQS	-43.58	41.9	HHHHHHHHHHHHHHHHHHHHHHHHHCCCCCCC
498	EEELRKFLEQYNQEEEQMRYQREGFGQGHQS	-43.58	38.7	HHHHHHHHHHHHHHHHHHHHHHHHHHCCCCCC
225	EEELRKFLEQYNQEYEQMEYQRNGRGQGHSY	-43.58	38.7	HHHHHHHHHHHHHHHHHHHHHHHHCCCCCCCC
897	EEELRKFLEQYNNEYEQMFYQQKGFGQGHQS	-43.53	41.9	HHHHHHHHHHHHHHHHHHHHHHHHHCCCCCCC
567	EEELKKILEQINQEEEQMRYQREGFGQGDSR	-43.49	45.2	HHHHHHHHHHHHHHHHHHHHHHHHHCCCCCCC
542	EEELKKLLEQINNEEEQMRYQQQGFGQGHSA	-43.43	38.7	HHHHHHHHHHCHHHHHHHHHHHHHCCCCCCCC
262	EEEKRKMLEKINNEQEQMSYQRHGFGQGDSR	-43.40	45.2	HHHHHHHHHHHHHHHHHHHHHHHHCCCCCCCC
313	EEELKKLLEQINQEEEQMRYQREGFGQGDSR	-43.38	45.2	HHHHHHHHHHHHHHHHHHHHHHHHHCCCCCCC
293	EEELRKLLEQLNQEKEQMSYQQQGFGQGDSR	-43.38	41.9	HHHHHHHHHHHHHHHHHHHHHHHHCCCCCCCC
907	EEELRKLLEQLNQEQEQMRYQQQGFGQGDSR	-43.38	41.9	HHHHHHHHHHHHHHHHHHHHHHHHCCCCCCCC
824	EEEARKMLEQINNEEEQMRYQRYGFGQGHSA	-43.37	38.7	HHHHHHHHHHHHHHHHHHHHHHHHCCCCCCCC
597	EEEKKKMLEQINNEEEQMRYQQQGFGQGHSA	-43.37	38.7	HHHHHHHHHHCHHHHHHHHHHHHHCCCCCCCC

751	EEEKKKMLEQINNEEEQMRYQREGFGQGHSS	-43.37	38.7	HHHHHHHHHHHCHHHHHHHHHHHHCCCCCCC
361	EEEKKKMLEQINNEEEQMRYQRYGFGQGHSS	-43.37	38.7	HHHHHHHHHHHCHHHHHHHHHHHCCCCCCCC
467	EEEKRKMLEQINNEAEQMRYQKEGFGQGHSS	-43.37	38.7	HHHHHHHHHHHHHHHHHHHHHHHHCCCCCCC
516	EEEKRKMLEQINNEAEQMRYQRQGFGQGHSS	-43.37	38.7	HHHHHHHHHHHHHHHHHHHHHHHCCCCCCCC
684	EEEKRKMLEQINNEAEQMRYQRYGFGQGHSA	-43.37	38.7	HHHHHHHHHHHHHHHHHHHHHHHCCCCCCCC
082	EEEKRKMLEQINNEEEQMRYQQYGFGQGHSS	-43.37	35.5	HHHHHHHHHHHHHHHHHHHHHHHCCCCCCCC
923	EEEKRKMLEQINNEQEQMSYQQQGFGQGHSS	-43.37	35.5	HHHHHHHHHHHHHHHHHHHHHHHCCCCCCCC
505	EEEKRKFLEKYNNESEQKRYQQQGFGQGDSR	-43.29	48.4	HHHHHHHHHHHHHHHHHHHHHHHCCCCCCCC
594	EEEKRKFLEKYNNEYEQMYYQQKGFGQGDSR	-43.29	48.4	HHHHHHHHHHHHHHHHHHHHHHHCCCCCCCC
686	EEELKKFLEKLNNEEEQMRYQQYGFGQGDSR	-43.29	51.6	HHHHHHHHHHHCHHHHHHHHHHHCCCCCCCC
931	EEELKKFLEKYNNESEQIRYQQQGFGQGDSR	-43.29	51.6	HHHHHHHHHHHHHHHHHHHHHHHCCCCCCCC
342	EEELRKFLEKYNNEAEQIYYQQNGFGQGDSR	-43.29	51.6	HHHHHHHHHHHHHHHHHHHHHHHCCCCCCCC
656	EEELRKFLEKYNNEYEQMFYQQKGFGQGDSR	-43.29	51.6	HHHHHHHHHHHHHHHHHHHHHHHCCCCCCCC
303	EEELKKFLEQLNNEEEQMRYQQYGFGQGHSS	-43.29	41.9	HHHHHHHHHHHHHHHHHHHHHHHCCCCCCCC
422	EEELKKFLEQYNNESEQMRYQQQGFGQGHQS	-43.29	41.9	HHHHHHHHHHHHHHHHHHHHHHHHCCCCCCC
734	EEELKKFLEQYNNESEQMRYQQQGFGQGHSS	-43.29	41.9	HHHHHHHHHHHHHHHHHHHHHHHHCCCCCCC
334	EEELRKFLEQFNNEYEQMFYQQKGFGQGHSS	-43.29	45.2	HHHHHHHHHHHHHHHHHHHHHHHHCCCCCCC
320	EEELRKFLEQYNNESEQMRYQQQGFGQGHQA	-43.29	38.7	HHHHHHHHHHHHHHHHHHHHHHHHCCCCCCC
241	EEELRKFLEQYNNESEQMRYQQQGFGQGHQS	-43.29	38.7	HHHHHHHHHHHHHHHHHHHHHHHHCCCCCCC
072	EEELRKFLEQYNNESEQMRYQRYGFGQGHQS	-43.29	38.7	HHHHHHHHHHHHHHHHHHHHHHHCCCCCCCC
234	EEELRKFLEQYNNEYEQEFYQQKGFGQGHSA	-43.29	41.9	HHHHHHHHHHHHHHHHHHHHHHHCCCCCCCC
951	EEELRRFLEQLNNEEEDMRYQQQGFGKGDSR	-43.28	51.6	HHHHHHHHHHHHHHHHHHHHHHHCCCCCCCC
817	EEELRKLLEKINNEAEQRRYQRYGFGQGHSS	-43.22	41.9	HHHHHHHHHHHHHHHHHHHHHHHCCCCCCCC
027	EEELRKFLEDYNNRAEDMRYQRQGFGKGHQS	-43.19	45.2	HHHHHHHHHHHHHHHHHHHHHHHHCCCCCCC
077	EEEKRKMLEKINQEAEQMRYQQYGFGQGDSR	-43.19	48.4	HHHHHHHHHHHHHHHHHHHHHHHCCCCCCCC
904	EEELKKLLEKINNEAEQMRYQKEGFGQGDSR	-43.18	51.6	HHHHHHHHHHHHHHHHHHHHHHHHCCCCCCC
483	EEELKKLLEKLNNEQEQIRYQQQGFGQGDSR	-43.18	48.4	HHHHHHHHHHHHHHHHHHHHHHHCCCCCCCC
970	EEEWKKLIEKINNEEEQKRYQRYGFGQGDSR	-43.18	45.2	HHHHHHHHHHHCHHHHHHHHHHHCCCCCCCC
667	EEELKKFLQKYNNESEDMRYQKEGFGKGDSR	-43.17	58.1	HHHHHHHHHHHHHCHHHHHHHHHCCCCCCCC
088	EEEKKKMLEQINNEAEQMRYQRYGFGQGHSA	-43.14	41.9	HHHHHHHHHHHHHHHHHHHHHHHCCCCCCCC
726	EEELRKFLEKFNNEYEQRFYQQKGFGQGHSA	-43.13	48.4	HHHHHHHHHHHHHHHHHHHHHHHCCCCCCCC
811	EEELKKLLEQINNEAEQMRYQQGGFGQGDSR	-43.09	48.4	HHHHHHHHHHHHHHHHHHHHHHCCCCCCCCC
634	EQELRKLLEQINNEEEQMRYQQYGFGQGDSR	-43.09	38.7	HHHHHHHHHHHHHHHHHHHHHHHCCCCCCCC
289	EEELKKFLEQYNNEEEQMRYQREGFGQGDSR	-43.03	48.4	HHHHHHHHHHHCHHHHHHHHHHHHCCCCCCC
596	EEELKKFLEQYNNESEQMRYQRYGFGQGDSR	-43.03	48.4	HHHHHHHHHHHHHHHHHHHHHHHCCCCCCCC
078	EEELRKFLEQFNNEYEQMFYQQKGFGQGDSR	-43.03	51.6	HHHHHHHHHHHHHHHHHHHHHHHCCCCCCCC
407	EEELRKFLEQLNNEEEQMRYQQYGFGQGDSR	-43.03	45.2	HHHHHHHHHHHHHHHHHHHHHHHCCCCCCCC
681	EEELRKFLEQYNNEEEQMRYQREGFGQGDSR	-43.03	45.2	HHHHHHHHHHHCHHHHHHHHHHHHCCCCCCC
451	EEEWKKFIEQYNNEAEQMRYQRQGFGQGDSR	-43.03	48.4	HHHHHHHHHHHHHHHHHHHHHHHCCCCCCCC
109	EQELRKFLEQFNNEYEQMFYQQKGFGQGDSR	-43.03	48.4	HHHHHHHHHHHHHHHHHHHHHHHCCCCCCCC
281	EEELRKILEQFNNEEEQMRYQREGFGQGDSR	-42.97	45.2	HHHHHHHHHHHCHHHHHHHHHHHHCCCCCCC
401	EEELKKLLEKLNNEKEQRRYQQQGFGQGDSR	-42.97	48.4	HHHHHHHHHHHHHHHHHHHHHHHCCCCCCCC
299	EEELKKFLEQFNNEQEQMSYQRHGFGQGHQS	-42.96	45.2	HHHHHHHHHHHHHHHHHHHHHHHCCCCCCCC
581	EEQLRKLLEQLNQEKEQMSYQRYGFGQGDSR	-42.94	45.2	HHHHHHHHHHHHHHHHHHHHHHHCCCCCCCC
205	EEEKRKFLEKYNQEAEQMRYQRYGFGQGDSR	-42.91	51.6	HHHHHHHHHHHHHHHHHHHHHHHCCCCCCCC
098	EEELKKFLEKFNQEYEQMFYQKEGFGQGDSR	-42.91	58.1	HHHHHHHHHHHHHHHHHHHHHHHCCCCCCCC
798	EEELRKFLEKLNQEEEQMRYQQYGFGQGDSR	-42.91	48.4	HHHHHHHHHHHHHHHHHHHHHHHCCCCCCCC
166	EEQLKKFLEQYNNESEQMRYQQQGFGQGHSA	-42.85	45.2	HHHHHHHHHHHHHHHHHHHHHHHHCCCCCCC
662	EEELRKFLEQYNQRSEQERYQQQGFGQGDSR	-42.79	41.9	HHHHHHHHHHHHHHHHHHHHHHHCCCCCCCC
585	EEELKKFLEDYNNRAEDMRYQQNGFGKGHSA	-42.67	48.4	HHHHHHHHHHHHHHHHHHHHHHHHCCCCCCC
911	EEELRKFLEKYNNEAEQIRYQQQGFGQGDSR	-42.62	51.6	HHHHHHHHHHHHHHHHHHHHHHHCCCCCCCC
458	EEELRKFLEQYNQEYEQMFYQQKGFGQGDSR	-42.60	48.4	HHHHHHHHHHHHHHHHHHHHHHHCCCCCCCC
437	EEEKKKMLEKINQEEEQMRYQREGFGQGDSR	-42.59	48.4	HHHHHHHHHHHHHHHHHHHHHHHHCCCCCCC
167	EEELRKFLEQFNNEYEQMFYQQKGFGQGTSR	-42.58	48.4	HHHHHHHHHHHHHHHHHHHHHHHHCCCCCCC
344	EEEKKKMLEQFNNEYEQMYYQQNGQGQGHSY	-42.57	41.9	HHHHHHHHHHHHHHHHHHHHHHHCCCCCCCC
478	EEELKKFLEQFNNEYEQMFYQQKGFGQGDSR	-42.52	54.8	HHHHHHHHHHHHHHHHHHHHHHHCCCCCCCC
860	EEELKKFLEQYNNEAEQMRYQQQGFGQGDSR	-42.52	51.6	HHHHHHHHHHHHHHHHHHHHHHHCCCCCCCC
327	EEELKKFLEQYNNEAEQMRYQRQGFGQGDSR	-42.52	51.6	HHHHHHHHHHHHHHHHHHHHHHHCCCCCCCC
010	EEELKKFLEQYNNESEQMRYQQQGFGQGDSR	-42.52	48.4	HHHHHHHHHHHHHHHHHHHHHHHCCCCCCCC
435	EEELRKFLEQYNNESEQMRYQQQGFGQGDSR	-42.52	45.2	HHHHHHHHHHHHHHHHHHHHHHHCCCCCCCC
022	EEELRKFLEQYNNEYEQEEYQRKGFGQGDSR	-42.52	45.2	HHHHHHHHHHHHHHHHHHHHHHHCCCCCCCC
318	EEEWKKFIEQYNNEEEQMRYQREGFGQGDSR	-42.52	45.2	HHHHHHHHHHHCHHHHHHHHHHHHCCCCCCC
792	EEEWRKFLEQFNNEYEQMFYQQQGFGQGDSR	-42.44	51.6	HHHHHHHHHHHHHHHHHHHHHHHCCCCCCCC
169	EEEWRKFIEQLNNEEEQMRYQQQGFGQGDSR	-42.43	41.9	HHHHHHHHHHHHHHHHHHHHHHHCCCCCCCC
244	EEEKRKMLEQINQEEEQMRYQQQGFGQGDSR	-42.42	41.9	HHHHHHHHHHHHHHHHHHHHHHHCCCCCCCC
377	EEELKKLLEQINNEQEQEEYQRKGFGQGDSR	-42.42	45.2	HHHHHHHHHHHHHHHHHHHHHHHCCCCCCCC
846	EEELKKLLEQLNNEKEQMRYQRQGFGQGDSR	-42.42	45.2	HHHHHHHHHHHHHHHHHHHHHHHCCCCCCCC

572	EEEKRKFLEKYNNESEQRRYQRYGFGQGDSR	-42.40	48.4	HHHHHHHHHHHCHHHHHHHHHHHCCCCCCCC
383	EEEARKMLEQINNEEEQMRYQQQGFGQGDSR	-42.13	45.2	HHHHHHHHHHHHHHHHHHHHHHHCCCCCCCC
258	EEELRKFLEKYNNESEQRRYQQEGFGQGDSR	-41.89	48.4	HHHHHHHHHHHHHHHHHHHHHHHCCCCCCCC
600	EEELKKFLEQYNNEEEQMRYQRQGFGQGDSR	-41.85	48.4	HHHHHHHHHHHCHHHHHHHHHHHCCCCCCCC
019	EEELKKFLEQYNNEYEQMYYQQKGFGQGDSR	-41.85	48.4	HHHHHHHHHHHHHHHHHHHHHHHCCCCCCCC
756	EEEKKKMLEQYEEEERQMRYQREGFGQGHSS	-41.54	32.3	HHHHHHHHHHHHHHHHHHHHHHHHCCCCCCC
350	EEEAKKMLEQINNEAEQMRYQRYGFGQGDSR	-41.46	51.6	HHHHHHHHHHHHHHHHHHHHHHHCCCCCCCC
639	EEEKRKMLEQINNEEEQMRYQREGFGQGDSR	-41.46	41.9	HHHHHHHHHHHCHHHHHHHHHHHHCCCCCCC
917	EEEKRKMLEQINNEEEQERYQQQGFGQGDSR	-41.19	41.9	HHHHHHHHHHHCHHHHHHHHHHHCCCCCCCC
928	EEELKKFLEDYNNRYEQEEYQRKGFGQGHSS	-40.53	38.7	HHHHHHHHHHHHHHHHHHHHHHHCCCCCCCC

Table S5. Summary of 695 peptide sequences designed using EvoEF2 and the evolutionary profile (weight = 1.00).

#label	#peptide	#binding(EEU)	#SeqID(%)	#Secondary structure
WT	EEQAKTFLDKFNHEAEDLFYQSSGLGKGDFR	-46.46	100.	HHHHHHHHHHHHHHHHHHHHHHHCCCCCCCC
322	EEELKKILEKINQEEEDMRYQQQGFGKGHDA	-49.06	48.4	HHHHHHHHHHHHHHHHHHHHHHHHCCCCCCC
591	EEELKKILEKLNQEQEDMRYQREGFGKGHSA	-49.06	48.4	HHHHHHHHHHHHHHHHHHHHHHHHHCCCCCC
276	EEEWRKILEKLNQEEEDIKYQQNGFGKGHDA	-49.06	45.2	HHHHHHHHHHHHHHHHHHHHHHHHCCCCCCC
783	EEELKKLLDQLNQEQEDMRYQQNGFGKGHDA	-49.05	48.4	HHHHHHHHHHHHHHHHHHHHHHHHCCCCCCC
773	EEELKKILEKINNEEEDIRYQQQGFGKGHDA	-48.77	48.4	HHHHHHHHHHHHHHHHHHHHHHHHCCCCCCC
255	EEELRKILEKLNNEQEDMRYQQNGFGKGHDA	-48.77	45.2	HHHHHHHHHHHHHHHHHHHHHHHHCCCCCCC
199	EEEWRKILEKLNNEEEDMKYQKEGFGKGHDA	-48.77	45.2	HHHHHHHHHHHHHHHHHHHHHHHHCCCCCCC
604	EEELRKILEKLNNEQEDMRYQRYGFGKGHDA	-48.53	45.2	HHHHHHHHHHHHHHHHHHHHHHHCCCCCCCC
895	EEELKKLLDQINNEEEDMRYQQQGFGKGHDA	-48.23	48.4	HHHHHHHHHHHHHHHHHHHHHHHHCCCCCCC
237	EEEARKFLDQFNNEYEDMFYQQKGFGKGHDA	-48.19	58.1	HHHHHHHHHHHHHHHHHHHHHHHHCCCCCCC
615	EEELKKLLEKLNQEEEDIKYQQNGFGKGHDA	-48.05	48.4	HHHHHHHHHHHHHHHHHHHHHHHHCCCCCCC
703	EEELKKLLEKLNQEKEDMTYQRHGFGKGHDA	-48.05	48.4	HHHHHHHHHHHHHHHHHHHHHHHCCCCCCCC
900	EEELRKLLEKINQEAEDIRYQQNGFGKGHDA	-48.05	48.4	HHHHHHHHHHHHHHHHHHHHHHHHCCCCCCC
758	EEELRKLLEKLNQEQEDMRYQKEGFGKGHDA	-48.05	45.2	HHHHHHHHHHHHHHHHHHHHHHHHHCCCCCC
496	EEELKKILEQLNNEKEDMSFQQQGFGKGKDA	-48.00	41.9	HHHHHHHHHHHHHHHHHHHHHHHHCCCCCCC
651	EEELKKLLEKINNEEEDIRFQQQGFGKGKDA	-47.99	45.2	HHHHHHHHHHHHHHHHHHHHHHHHCCCCCCC
355	EEELRKLLEKLNNEQEDIRYQQQGFGKGHDA	-47.94	45.2	HHHHHHHHHHHHHHHHHHHHHHHHCCCCCCC
638	EEELKKFLEQYNNEAEDMRYQRQGFGKGHDA	-47.92	51.6	HHHHHHHHHHHHHHHHHHHHHHHHCCCCCCC
806	EEELRKLLEKIENEREDITYQQNGFGKGHDA	-47.91	41.9	HHHHHHHHHHHHHHHHHHHHHHHCCCCCCCC
936	EEELRKLLEKLNQEQEDIRYQQNGFGKGHDA	-47.81	45.2	HHHHHHHHHHHHHHHHHHHHHHHHCCCCCCC
091	EEELKKLLEKINNEAEDMRYQQQGFGKGHDA	-47.77	51.6	HHHHHHHHHHHHHHHHHHHHHHHHCCCCCCC
263	EEELKKILEQINNEAEDMRYQQGGFGKGHDF	-47.77	48.4	HHHHHHHHHHHHHHHHHHHHHHCCCCCCCCC
075	EEELKKILEQINNEEEDMRYQQQGFGKGHDA	-47.77	45.2	HHHHHHHHHHHHHHHHHHHHHHHHCCCCCCC
934	EEELKKILEQINNEEEDMRYQQYGFGKGHDA	-47.77	45.2	HHHHHHHHHHHHHHHHHHHHHHHCCCCCCCC
415	EEELKKILEQLNNEEEDMKYQQNGFGKGHDA	-47.77	45.2	HHHHHHHHHHHHHHHHHHHHHHHHCCCCCCC
512	EEELKKILEQLNNEQEDMRYQQQGFGKGHDA	-47.77	45.2	HHHHHHHHHHHHHHHHHHHHHHHHCCCCCCC
623	EEELKKILEQLNNEQEDMRYQRYGFGKGHDA	-47.77	45.2	HHHHHHHHHHHHHHHHHHHHHHHCCCCCCCC
316	EEELRKILEQINNEEEDMRYQQYGFGKGHDA	-47.77	41.9	HHHHHHHHHHHHHHHHHHHHHHHCCCCCCCC
886	EEELRKILEQLNNEQEDMRYQQQGFGKGHDA	-47.77	41.9	HHHHHHHHHHHHHHHHHHHHHHHHCCCCCCC
239	EEELKKLLEKINNEAEDIRYQQNGFGKGHDA	-47.75	51.6	HHHHHHHHHHHHHHHHHHHHHHHHCCCCCCC
910	EEELKKLLEKINNEEEDIRYQQQGFGKGHDA	-47.75	48.4	HHHHHHHHHHHHHHHHHHHHHHHHCCCCCCC
413	EEELKKLLEKINNEEEDIRYQQQGFGKGHSA	-47.75	48.4	HHHHHHHHHHHHHHHHHHHHHHHHCCCCCCC
720	EEELKKLLEKINNEEEDMRYQQYGFGKGHDA	-47.75	48.4	HHHHHHHHHHHHHHHHHHHHHHHCCCCCCCC
999	EEELKKLLEKINNEEEDMRYQREGFGKGHDA	-47.75	48.4	HHHHHHHHHHHHHHHHHHHHHHHHHCCCCCC
526	EEELKKLLEKLNNEKEDMRYQQQGFGKGHDA	-47.75	48.4	HHHHHHHHHHHHHHHHHHHHHHHHCCCCCCC
733	EEELKKLLEKLNNEQEDIRYQQNGFGKGHDA	-47.75	48.4	HHHHHHHHHHHHHHHHHHHHHHHHCCCCCCC
979	EEELKKLLEKLNNEQEDMRYQQQGFGKGHDA	-47.75	48.4	HHHHHHHHHHHHHHHHHHHHHHHHCCCCCCC
363	EEELRKLLEKINNEAEDIRYQQNGFGKGHDA	-47.75	48.4	HHHHHHHHHHHHHHHHHHHHHHHHCCCCCCC
045	EEELRKLLEKINNEAEDIRYQQNGFGKGHSA	-47.75	48.4	HHHHHHHHHHHHHHHHHHHHHHHHCCCCCCC
849	EEELRKLLEKINNEAEDMRYQQQGFGKGHDA	-47.75	48.4	HHHHHHHHHHHHHHHHHHHHHHHHCCCCCCC
819	EEELRKLLEKINNEAEDMRYQRQGFGKGHDA	-47.75	48.4	HHHHHHHHHHHHHHHHHHHHHHHHCCCCCCC
361	EEELRKLLEKINNEEEDIRYQQQGFGKGHDA	-47.75	45.2	HHHHHHHHHHHHHHHHHHHHHHHHCCCCCCC
454	EEELRKLLEKINNEEEDIRYQQQGFGKGHSA	-47.75	45.2	HHHHHHHHHHHHHHHHHHHHHHHHCCCCCCC
275	EEELRKLLEKINNEEEDMRYQQYGFGKGHDA	-47.75	45.2	HHHHHHHHHHHHHHHHHHHHHHHCCCCCCCC
326	EEELRKLLEKLNNEKEDMRYQQQGFGKGHDA	-47.75	45.2	HHHHHHHHHHHHHHHHHHHHHHHHCCCCCCC
285	EEELRKLLEKLNNEKEDMSYQQQGFGKGHDA	-47.75	45.2	HHHHHHHHHHHHHHHHHHHHHHHCCCCCCCC
107	EEELRKLLEKLNNEQEDIRYQQNGFGKGHDA	-47.75	45.2	HHHHHHHHHHHHHHHHHHHHHHHHCCCCCCC
510	EEELRKLLEKLNNEQEDMRYQQNGFGKGHDA	-47.75	45.2	HHHHHHHHHHHHHHHHHHHHHHHHCCCCCCC
567	EEELRKLLEKLNNEQEDMRYQRQGFGKGHDA	-47.75	45.2	HHHHHHHHHHHHHHHHHHHHHHHHCCCCCCC
469	EEEWKKLMEKYNNEEEDMRYQREGFGKGHDA	-47.75	45.2	HHHHHHHHHHHCCHHHHHHHHHHHHCCCCCC
582	EQELRKLLEKLNNEQEDIRYQQNGFGKGHDA	-47.75	41.9	HHHHHHHHHHHHHHHHHHHHHHHHCCCCCCC
959	EEEARKFLEKYNQEEEDMRYQQQGFGKGHSA	-47.67	51.6	HHHHHHHHHHHHHHHHHHHHHHHHCCCCCCC
833	EEELRKILEQLNNEQEDMRYQQGGFGKGHDA	-47.53	41.9	HHHHHHHHHHHHHHHHHHHHHHCCCCCCCCC
788	EEEARKFLEKYNQEEEDMRYQREGFGKGHSA	-47.49	51.6	HHHHHHHHHHHHHHHHHHHHHHHHHCCCCCC
228	EEEARKFLEKFNQEEEDMRYQRQGFGKGHDA	-47.48	54.8	HHHHHHHHHHHHHHHHHHHHHHHHCCCCCCC
042	EEEARKFLEKYNQEAEDIRYQQNGFGKGHDA	-47.48	54.8	HHHHHHHHHHHHHHHHHHHHHHHHCCCCCCC
694	EEEARKFLEKYNQEAEDIYYQQNGFGKGHDA	-47.48	54.8	HHHHHHHHHHHHHHHHHHHHHHHCCCCCCCC
301	EEEARKFLEKYNQEAEDMRYQQQGFGKGHDA	-47.48	54.8	HHHHHHHHHHHHHHHHHHHHHHHHCCCCCCC
486	EEEARKFLEKYNQEEEDMRYQQQGFGKGHDA	-47.48	51.6	HHHHHHHHHHHHHHHHHHHHHHHHCCCCCCC
006	EEEARKFLEKYNQEEEDMRYQREGFGKGHDA	-47.48	51.6	HHHHHHHHHHHHHHHHHHHHHHHHHCCCCCC
547	EEEARKFLEKYNQESEDIRYQQNGFGKGHDA	-47.48	51.6	HHHHHHHHHHHHHHHHHHHHHHHCCCCCCCC
101	EEEARKFLEKYNQESEDIRYQQQGFGKGHDA	-47.48	51.6	HHHHHHHHHHHHHHHHHHHHHHHHCCCCCCC
504	EEEARKFLEKYNQESEDMRYQQQGFGKGHDA	-47.48	51.6	HHHHHHHHHHHHHHHHHHHHHHHHCCCCCCC

712	EEEARKFLEKYNQESEDMRYQRYGFGKGHDA	-47.48	51.6	HHHHHHHHHHHHHHHHHHHHHHHCCCCCCCC
995	EEELKKFLEKFNQEYEDMFYQQKGFGKGHDA	-47.48	58.1	HHHHHHHHHHHHHHHHHHHHHHHHCCCCCCC
704	EEELKKFLEKLNQEEEDIRYQQQGFGKGHDA	-47.48	51.6	HHHHHHHHHHHHHHHHHHHHHHHHCCCCCCC
551	EEELKKFLEKYNQEAEDIYYQQNGFGKGHDA	-47.48	54.8	HHHHHHHHHHHHHHHHHHHHHHHCCCCCCCC
865	EEELKKFLEKYNQEEEDMRYQREGFGKGHDA	-47.48	51.6	HHHHHHHHHHHHHHHHHHHHHHHHHCCCCCC
648	EEELKKFLEKYNQESEDIRYQQNGFGKGHDA	-47.48	51.6	HHHHHHHHHHHHHHHHHHHHHHHCCCCCCCC
259	EEELRKLLEKINQEEEDMRYQQYGFGKGHDA	-47.45	45.2	HHHHHHHHHHHHHHHHHHHHHHHCCCCCCCC
874	EEEAKKFLEKYNQEEEDMYYQQKGFGKGHDA	-47.43	54.8	HHHHHHHHHHHHHHHHHHHHHHHHCCCCCCC
687	EEEARKFLEKYNNEAEDIRFQQNGFGKGKSA	-47.42	51.6	HHHHHHHHHHHHHHHHHHHHHHHCCCCCCCC
291	EEELKKFLEKYNNEAEDIYFQQNGFGKGKDA	-47.42	51.6	HHHHHHHHHHHHHHHHHHHHHHHCCCCCCCC
965	EEELKKFLEKYNNESEDMRFQQQGFGKGKDA	-47.42	48.4	HHHHHHHHHHHHHHHHHHHHHHHHCCCCCCC
553	EEELKKFLEKLNNEEEDMRYQQQGFGKGHDA	-47.37	51.6	HHHHHHHHHHHHHHHHHHHHHHHHCCCCCCC
216	EEELKKFLEKYNNESEDIRYQQNGFGKGHDA	-47.37	51.6	HHHHHHHHHHHHCHHHHHHHHHHCCCCCCCC
057	EEELRKLLEQINNEAEDRTFQQNGFGKGKDA	-47.33	41.9	HHHHHHHHHHHHHHHHHHHHHHHCCCCCCCC
323	EEELKKLLEQINQEEEDMRYQQQGFGKGHDA	-47.30	45.2	HHHHHHHHHHHHHHHHHHHHHHHHCCCCCCC
164	EEELKKLLEQINQEAEDMRYQQQGFGKGHDA	-47.28	48.4	HHHHHHHHHHHHHHHHHHHHHHHHCCCCCCC
892	EEELKKLLEQINQEEEDMRYQREGFGKGHDA	-47.28	45.2	HHHHHHHHHHHHHHHHHHHHHHHHHCCCCCC
552	EEELKKLLEQLNQEEEDRRYQQNGFGKGHDA	-47.28	45.2	HHHHHHHHHHHHHHHHHHHHHHHCCCCCCCC
675	EEELKKLLEQLNQEKEDMRYQQQGFGKGHDA	-47.28	45.2	HHHHHHHHHHHHHHHHHHHHHHHHCCCCCCC
668	EEELKKLLEQLNQEQEDMRYQQNGFGKGHDA	-47.28	45.2	HHHHHHHHHHHHHHHHHHHHHHHHCCCCCCC
158	EEELKKLLEQLNQEQEDMRYQRQGFGKGHDA	-47.28	45.2	HHHHHHHHHHHHHHHHHHHHHHHHCCCCCCC
197	EEELRKLLEQINQEAEDIRYQQNGFGKGHDA	-47.28	45.2	HHHHHHHHHHHHHHHHHHHHHHHHCCCCCCC
559	EEELRKLLEQINQEEEDMFYQQKGFGKGHSA	-47.28	45.2	HHHHHHHHHHHHHHHHHHHHHHHHCCCCCCC
973	EEELRKLLEQINQEEEDMRYQQQGFGKGHDA	-47.28	41.9	HHHHHHHHHHHHHHHHHHHHHHHHCCCCCCC
889	EEELRKLLEQINQEEEDMRYQREGFGKGHDA	-47.28	41.9	HHHHHHHHHHHHHHHHHHHHHHHHHCCCCCC
795	EEELRKLLEQINQEEEDMRYQRYGFGKGHDA	-47.28	41.9	HHHHHHHHHHHHHHHHHHHHHHHCCCCCCCC
459	EEELRKLLEQLNQEKEDMSYQQQGFGKGHDA	-47.28	41.9	HHHHHHHHHHHHHHHHHHHHHHHCCCCCCCC
610	EEELRKLLEQLNQEQEDMRYQQNGFGKGHDA	-47.28	41.9	HHHHHHHHHHHHHHHHHHHHHHHHCCCCCCC
398	EQELRKLLEQINQEEEDMRYQQQGFGKGHDA	-47.28	38.7	HHHHHHHHHHHHHHHHHHHHHHHHCCCCCCC
458	EEELRKILEQFNNEEEDMYYQQYGFGKGHDA	-47.25	45.2	HHHHHHHHHHHHHHHHHHHHHHHCCCCCCCC
426	EEELKKLLEQINNEAEDMRFQKEGFGKGKDA	-47.22	45.2	HHHHHHHHHHHHHHHHHHHHHHHHCCCCCCC
605	EEELRKLLEQINNEEEDMRFQQQGFGKGKDA	-47.22	38.7	HHHHHHHHHHHHHHHHHHHHHHHHCCCCCCC
757	EEELRKLLEQLNNEQEDMRFQQQGFGKGKDA	-47.22	38.7	HHHHHHHHHHHHHHHHHHHHHHHHCCCCCCC
393	EEELKKLLEKINNEEEDIRYQQQGFGKGDSR	-47.20	54.8	HHHHHHHHHHHHHHHHHHHHHHHCCCCCCCC
794	EEELKKLLEKLNNEQEDIRYQQNGFGKGDSR	-47.20	54.8	HHHHHHHHHHHHHHHHHHHHHHHCCCCCCCC
626	EEELRKILEQYNNEEEDMRYQREGFGKGHDA	-47.20	41.9	HHHHHHHHHHHCHHHHHHHHHHHHHCCCCCC
825	EEEWKKFLEKLNNEAEDMQYQRAGFGKGHDA	-47.19	54.8	HHHHHHHHHHHHHHHHHHHHHHHHHCCCCCC
520	EEEARKFLEKFNNEAEDMQYQRAGFGKGHDA	-47.19	58.1	HHHHHHHHHHHHHHHHHHHHHHHHHCCCCCC
894	EEEARKFLEKFNNEEEDMSYQQQGFGKGHDA	-47.19	54.8	HHHHHHHHHHHHHHHHHHHHHHHCCCCCCCC
441	EEEARKFLEKFNNEYEDMFYQQKGFGKGHDA	-47.19	58.1	HHHHHHHHHHHHHHHHHHHHHHHHCCCCCCC
971	EEEARKFLEKFNNEYEDMFYQQQGFGKGHDA	-47.19	58.1	HHHHHHHHHHHHHHHHHHHHHHHHCCCCCCC
240	EEEARKFLEKLNNEEEDIRYQQQGFGKGHDA	-47.19	51.6	HHHHHHHHHHHHHHHHHHHHHHHHCCCCCCC
770	EEEARKFLEKLNNEEEDMRYQQQGFGKGHDA	-47.19	51.6	HHHHHHHHHHHHHHHHHHHHHHHHCCCCCCC
400	EEEARKFLEKYNNEAEDIRYQQGGFGKGHDA	-47.19	54.8	HHHHHHHHHHHHHHHHHHHHHHCCCCCCCCC
753	EEEARKFLEKYNNEAEDIRYQQQGFGKGHDA	-47.19	54.8	HHHHHHHHHHHHHHHHHHHHHHHHCCCCCCC
406	EEEARKFLEKYNNEAEDIYYQQNGFGKGHDA	-47.19	54.8	HHHHHHHHHHHHHHHHHHHHHHHCCCCCCCC
994	EEEARKFLEKYNNEAEDMRYQQQGFGKGHDA	-47.19	54.8	HHHHHHHHHHHHHHHHHHHHHHHHCCCCCCC
569	EEEARKFLEKYNNESEDIRYQQGGFGKGHDA	-47.19	51.6	HHHHHHHHHHHCHHHHHHHHHCCCCCCCCCC
683	EEEARKFLEKYNNESEDIRYQQQGFGKGHDA	-47.19	51.6	HHHHHHHHHHHCHHHHHHHHHHHHCCCCCCC
131	EEEARKFLEKYNNESEDIRYQQQGFGKGHSA	-47.19	51.6	HHHHHHHHHHHCHHHHHHHHHHHHCCCCCCC
980	EEELKKFLEKFNNEYEDIFYQQKGFGKGHDA	-47.19	58.1	HHHHHHHHHHHHHHHHHHHHHHHHCCCCCCC
546	EEELKKFLEKFNNEYEDIFYQQQGFGKGHDA	-47.19	58.1	HHHHHHHHHHHHHHHHHHHHHHHCCCCCCCC
089	EEELKKFLEKFNNEYEDMFYQQKGFGKGHDA	-47.19	58.1	HHHHHHHHHHHHHHHHHHHHHHHHCCCCCCC
620	EEELKKFLEKFNNEYEDMFYQQKGFGKGHSA	-47.19	58.1	HHHHHHHHHHHHHHHHHHHHHHHHCCCCCCC
126	EEELKKFLEKLNNEEEDMRYQREGFGKGHDA	-47.19	51.6	HHHHHHHHHHHHHHHHHHHHHHHHHCCCCCC
207	EEELKKFLEKYNNEAEDIRYQQNGFGKGHDA	-47.19	54.8	HHHHHHHHHHHHHHHHHHHHHHHHCCCCCCC
144	EEELKKFLEKYNNEAEDIRYQQQGFGKGHDA	-47.19	54.8	HHHHHHHHHHHHHHHHHHHHHHHHCCCCCCC
961	EEELKKFLEKYNNEAEDIYYQQNGFGKGHDA	-47.19	54.8	HHHHHHHHHHHHHHHHHHHHHHHCCCCCCCC
976	EEELKKFLEKYNNEAEDIYYQQNGFGKGHSA	-47.19	54.8	HHHHHHHHHHHHHHHHHHHHHHHCCCCCCCC
409	EEELKKFLEKYNNEAEDMRYQQQGFGKGHDA	-47.19	54.8	HHHHHHHHHHHHHHHHHHHHHHHHCCCCCCC
008	EEELKKFLEKYNNEEEDMRYQREGFGKGHDA	-47.19	51.6	HHHHHHHHHHHCCHHHHHHHHHHHHCCCCCC
984	EEELKKFLEKYNNEEEDMSYQRYGFGKGHDA	-47.19	51.6	HHHHHHHHHHHHHHHHHHHHHHCCCCCCCCC
090	EEELKKFLEKYNNESEDIRYQQGGFGKGHDF	-47.19	51.6	HHHHHHHHHHHHCHHHHHHHHCCCCCCCCCC
442	EEELKKFLEKYNNESEDIRYQQQGFGKGHDA	-47.19	51.6	HHHHHHHHHHHHCHHHHHHHHHHHCCCCCCC
803	EEELKKFLEKYNNEYEDIFYQQKGFGKGHDA	-47.19	54.8	HHHHHHHHHHHHHHHHHHHHHHHHCCCCCCC
310	EEELKKFLEKYNNEYEDMEYQRNGRGKGHDY	-47.19	51.6	HHHHHHHHHHHHHHHHHHHHHHHCCCCCCCC
784	EEELKKFLEKYNNEYEDMFYQQKGFGKGHDA	-47.19	54.8	HHHHHHHHHHHHHHHHHHHHHHHHCCCCCCC

129	EEEWKKFIEKYNNEAEDIYYQQNGFGKGHDA	-47.19	51.6	HHHHHHHHHHHHHHHHHHHHHHHCCCCCCCC
117	EQEARKFLEKYNNESEDIRYQQGGFGKGHDF	-47.19	48.4	HHHHHHHHHHHHHHHHHHHHHCCCCCCCCCC
989	EEEARKFLEKFNQEYEDMFYQQKGFGKGHDA	-47.16	58.1	HHHHHHHHHHHHHHHHHHHHHHHHCCCCCCC
755	EEELKKLLEKLNQEQEDMYYQQKGFGKGDSR	-47.15	54.8	HHHHHHHHHHHHHHHHHHHHHHHCCCCCCCC
283	EEELKKLLEQINQEAEDMRYQQNGFGKGHDA	-47.04	48.4	HHHHHHHHHHHHHHHHHHHHHHHHCCCCCCC
587	EEEARKFLEKYNQEAEQIRYQQQGFGRGHDA	-47.03	48.4	HHHHHHHHHHHHHHHHHHHHHHHCCCCCCCC
289	EEELKKLLEQINNEEEDMRYQQQGFGKGHDA	-47.00	45.2	HHHHHHHHHHHHHHHHHHHHHHHHCCCCCCC
431	EEELKKLLEQINNEEEDMRYQREGFGKGHDA	-47.00	45.2	HHHHHHHHHHHHHHHHHHHHHHHHHCCCCCC
530	EEELRKLLEQINNEKEDMEYQRAGFGKGHDA	-47.00	41.9	HHHHHHHHHHHHHHHHHHHHHHHHCCCCCCC
066	EEELKKFLEKYNQEAENIYKQQNGFGSGKDA	-46.99	45.2	HHHHHHHHHHHHHHHHHHHHHHHCCCCCCCC
108	EEELKKLLEQINNEAEDIRYQQNGFGKGHDA	-46.99	48.4	HHHHHHHHHHHHHHHHHHHHHHHHCCCCCCC
109	EEELKKLLEQINNEAEDIRYQQNGFGKGHSA	-46.99	48.4	HHHHHHHHHHHHHHHHHHHHHHHHCCCCCCC
529	EEELKKLLEQINNEAEDMRYQQNGFGKGHDA	-46.99	48.4	HHHHHHHHHHHHHHHHHHHHHHHHCCCCCCC
831	EEELKKLLEQINNEAEDMRYQQQGFGKGHDA	-46.99	48.4	HHHHHHHHHHHHHHHHHHHHHHHHCCCCCCC
843	EEELKKLLEQINNEEEDMRYQQQGFGKGHSA	-46.99	45.2	HHHHHHHHHHHHHHHHHHHHHHHHCCCCCCC
058	EEELKKLLEQINNEEEDMRYQQYGFGKGHDA	-46.99	45.2	HHHHHHHHHHHHHHHHHHHHHHHCCCCCCCC
014	EEELKKLLEQINNEKEDMEYQRAGFGKGHDA	-46.99	45.2	HHHHHHHHHHHHHHHHHHHHHHHHCCCCCCC
966	EEELKKLLEQLNNEEEDKQYQRAGFGKGHDA	-46.99	45.2	HHHHHHHHHHHHHHHHHHHHHHHHCCCCCCC
223	EEELKKLLEQLNNEEEDMKYQQNGFGKGHDA	-46.99	45.2	HHHHHHHHHHHHHHHHHHHHHHHHCCCCCCC
281	EEELKKLLEQLNNEEEDRRYQQGGFGKGHDA	-46.99	45.2	HHHHHHHHHHHHHHHHHHHHHHCCCCCCCCC
438	EEELKKLLEQLNNEKEDMRYQQQGFGKGHDA	-46.99	45.2	HHHHHHHHHHHHHHHHHHHHHHHHCCCCCCC
991	EEELKKLLEQLNNEKEDMSYQQQGFGKGHDA	-46.99	45.2	HHHHHHHHHHHHHHHHHHHHHHHCCCCCCCC
226	EEELKKLLEQLNNEQEDIRYQQGGFGKGHDF	-46.99	45.2	HHHHHHHHHHHHHHHHHHHHHHCCCCCCCCC
955	EEELKKLLEQLNNEQEDMRYQQNGFGKGHDA	-46.99	45.2	HHHHHHHHHHHHHHHHHHHHHHHHCCCCCCC
192	EEELKKLLEQLNNEQEDMRYQQQGFGKGHDA	-46.99	45.2	HHHHHHHHHHHHHHHHHHHHHHHHCCCCCCC
368	EEELKKLLEQLNNEQEDMRYQRQGFGKGHDA	-46.99	45.2	HHHHHHHHHHHHHHHHHHHHHHHHCCCCCCC
815	EEELRKLLEQINNEAEDMQYQRAGFGKGHDA	-46.99	45.2	HHHHHHHHHHHHHHHHHHHHHHHHHCCCCCC
203	EEELRKLLEQINNEAEDMRYQQNGFGKGHDA	-46.99	45.2	HHHHHHHHHHHHHHHHHHHHHHHHCCCCCCC
210	EEELRKLLEQINNEAEDMSYQKEGFGKGHDA	-46.99	45.2	HHHHHHHHHHHHHHHHHHHHHHHHCCCCCCC
187	EEELRKLLEQINNEEEDMRYQQQGFGKGHDA	-46.99	41.9	HHHHHHHHHHHHHHHHHHHHHHHHCCCCCCC
399	EEELRKLLEQINNEEEDMRYQQYGFGKGHDA	-46.99	41.9	HHHHHHHHHHHHHHHHHHHHHHHCCCCCCCC
201	EEELRKLLEQINNEEEDMRYQREGFGKGHDA	-46.99	41.9	HHHHHHHHHHHHHHHHHHHHHHHHHCCCCCC
484	EEELRKLLEQINNEKEDMEYQRAGFGKGHSA	-46.99	41.9	HHHHHHHHHHHHHHHHHHHHHHHHHCCCCCC
295	EEELRKLLEQLNNEEEDMKYQKEGFGKGHDA	-46.99	41.9	HHHHHHHHHHHHHHHHHHHHHHHHHCCCCCC
649	EEELRKLLEQLNNEEEDMKYQQNGFGKGHDA	-46.99	41.9	HHHHHHHHHHHHHHHHHHHHHHHHCCCCCCC
483	EEELRKLLEQLNNEEEDMRYQQNGFGKGHDA	-46.99	41.9	HHHHHHHHHHHHHHHHHHHHHHHHCCCCCCC
159	EEELRKLLEQLNNEKEDMSYQQQGFGKGHDA	-46.99	41.9	HHHHHHHHHHHHHHHHHHHHHHHCCCCCCCC
983	EEELRKLLEQLNNEQEDMRYQQNGFGKGHDA	-46.99	41.9	HHHHHHHHHHHHHHHHHHHHHHHHCCCCCCC
196	EEELRKLLEQLNNEQEDMRYQQQGFGKGHDA	-46.99	41.9	HHHHHHHHHHHHHHHHHHHHHHHHCCCCCCC
284	EEELRKLLEQLNNEQEDMRYQRYGFGKGHDA	-46.99	41.9	HHHHHHHHHHHHHHHHHHHHHHHCCCCCCCC
402	EEELKKFLEKFNNEYEDMYYQQQGFGKGHDA	-46.95	54.8	HHHHHHHHHHHHHHHHHHHHHHHHCCCCCCC
044	EEELKKFLEQYNQEAEDMRFQQGGFGKGKDA	-46.95	48.4	HHHHHHHHHHHHHHHHHHHHHHCCCCCCCCC
902	EEELKKFLEKLNQEEEDMRYQQYGFGKGHDA	-46.89	51.6	HHHHHHHHHHHHHHHHHHHHHHHCCCCCCCC
848	EEEARKFLEKYNNEAEQIYYQQNGFGRGHDA	-46.83	48.4	HHHHHHHHHHHHHHHHHHHHHHHCCCCCCCC
723	EEELKKLLDKINNEEEQIRYQQQGFGQGHDA	-46.80	45.2	HHHHHHHHHHHHHHHHHHHHHHHCCCCCCCC
065	EEELKKLLEKINQEAEDIRYQQQGFGKGDSR	-46.80	58.1	HHHHHHHHHHHHHHHHHHHHHHHCCCCCCCC
561	EEELRKLLEKINQEEEDIRYQQQGFGKGDSR	-46.80	51.6	HHHHHHHHHHHHHHHHHHHHHHHCCCCCCCC
329	EEEARKFLEKFNQEYEDMFYQQKGFGKGDSR	-46.75	64.5	HHHHHHHHHHHHHHHHHHHHHHHCCCCCCCC
796	EEEARKFLEKYNQEAEDMRYQRQGFGKGDSR	-46.75	61.3	HHHHHHHHHHHHHHHHHHHHHHHCCCCCCCC
935	EEELKKFLEKYNQEAEDIRYQQNGFGKGDSR	-46.75	61.3	HHHHHHHHHHHHHHHHHHHHHHHCCCCCCCC
296	EEEAKKFLEQYNQEYEDMFYQQKGFGKGHDA	-46.72	54.8	HHHHHHHHHHHHHHHHHHHHHHHHCCCCCCC
088	EEEARKFLEQFNQEYEDMFYQQKGFGKGHDA	-46.72	54.8	HHHHHHHHHHHHHHHHHHHHHHHHCCCCCCC
855	EEEARKFLEQFNQEYEDMFYQQKGFGKGHSA	-46.72	54.8	HHHHHHHHHHHHHHHHHHHHHHHHCCCCCCC
920	EEEARKFLEQFNQEYEDMYYQQQGFGKGHDA	-46.72	51.6	HHHHHHHHHHHHHHHHHHHHHHHHCCCCCCC
969	EEEARKFLEQLNQEEEDMRYQQQGFGKGHSA	-46.72	48.4	HHHHHHHHHHHHHHHHHHHHHHHHCCCCCCC
506	EEEARKFLEQLNQEEEDMRYQQYGFGKGHDA	-46.72	48.4	HHHHHHHHHHHHHHHHHHHHHHHCCCCCCCC
345	EEEARKFLEQYNQEAEDIYYQQNGFGKGHDA	-46.72	51.6	HHHHHHHHHHHHHHHHHHHHHHHCCCCCCCC
437	EEEARKFLEQYNQEAEDMRYQQNGFGKGHDA	-46.72	51.6	HHHHHHHHHHHHHHHHHHHHHHHHCCCCCCC
171	EEEARKFLEQYNQEAEDMRYQREGFGKGHDA	-46.72	51.6	HHHHHHHHHHHHHHHHHHHHHHHHHCCCCCC
248	EEEARKFLEQYNQEEEDMRYQREGFGKGHDA	-46.72	48.4	HHHHHHHHHHHHHHHHHHHHHHHHHCCCCCC
858	EEEARKFLEQYNQESEDIRYQQNGFGKGHDA	-46.72	48.4	HHHHHHHHHHHHHHHHHHHHHHHCCCCCCCC
766	EEEARKFLEQYNQESEDIRYQQQGFGKGHDA	-46.72	48.4	HHHHHHHHHHHHHHHHHHHHHHHHCCCCCCC
279	EEEARKFLEQYNQESEDMRYQQQGFGKGHDA	-46.72	48.4	HHHHHHHHHHHHHHHHHHHHHHHHCCCCCCC
360	EEELKKFLEQLNQEAEDMRYQQNGFGKGHDA	-46.72	51.6	HHHHHHHHHHHHHHHHHHHHHHHHCCCCCCC
321	EEELKKFLEQYNQEAEDIRYQQQGFGKGHDA	-46.72	51.6	HHHHHHHHHHHHHHHHHHHHHHHHCCCCCCC
083	EEELKKFLEQYNQEEEDMRYQREGFGKGHSA	-46.72	48.4	HHHHHHHHHHHHHHHHHHHHHHHHHCCCCCC
948	EEELKKFLEQYNQESEDMRYQQQGFGKGHDA	-46.72	48.4	HHHHHHHHHHHHHHHHHHHHHHHHCCCCCCC

143	EEELKKFLEQYNQEYEDMFYQQQGFGKGHSA	-46.72	51.6	HHHHHHHHHHHHHHHHHHHHHHHHCCCCCCC
715	EEELRKFLEQFNQEYEDMFYQQKGFGKGHDA	-46.72	51.6	HHHHHHHHHHHHHHHHHHHHHHHHCCCCCCC
808	EEELRKLLEKINNEEEQMRYQREGFGRGDSR	-46.71	45.2	HHHHHHHHHHHCHHHHHHHHHHHCCCCCCCC
769	EEELKKLLEQLNQEEEDMKYQQNGFGKGHDA	-46.68	45.2	HHHHHHHHHHHHHHHHHHHHHHHHCCCCCCC
069	EEEARKFLEKYNNEAEDIRYQQNGFGKGHDA	-46.67	54.8	HHHHHHHHHHHHHHHHHHHHHHHHCCCCCCC
156	EEEAKKFLEQYNNEEEDMFFQQKGFGKGKDA	-46.66	51.6	HHHHHHHHHHHHHHHHHHHHHHHHCCCCCCC
667	EEEARKFLEQFNNEEEDMQFQRSGFGKGKDA	-46.66	51.6	HHHHHHHHHHHHHHHHHHHHHHHHCCCCCCC
601	EEEARKFLEQFNNEYEDMFFQQKGFGKGKDA	-46.66	51.6	HHHHHHHHHHHHHHHHHHHHHHHHCCCCCCC
468	EEEARKFLEQYNNEAEDMRFQQQGFGKGKDA	-46.66	48.4	HHHHHHHHHHHHHHHHHHHHHHHHCCCCCCC
374	EEELKKFLEKFNNEYEDMFYQQKGFGKGDSR	-46.64	64.5	HHHHHHHHHHHHHHHHHHHHHHCCCCCCCCC
634	EEELKKFLEQYNNEAEDMRYQQQGFGKGSDY	-46.63	51.6	HHHHHHHHHHHHHHHHHHHHHHHHCCCCCCC
016	EEQWKKLIEQINNEKEDMEYQRAGFGKGHDA	-46.55	45.2	HHHHHHHHHHHHHHHHHHHHHHHHCCCCCCC
749	EEELKKLLEQINQEAEDMQYQRAGFGKGDSR	-46.54	54.8	HHHHHHHHHHHHHHHHHHHHHHHHCCCCCCC
046	EEELKKLLEQLNQEQEDMRYQQNGFGKGDSR	-46.54	51.6	HHHHHHHHHHHHHHHHHHHHHHHCCCCCCCC
876	EEELRKLLEQLNQEQEDMRYQQNGFGKGDSR	-46.54	48.4	HHHHHHHHHHHHHHHHHHHHHHHCCCCCCCC
568	EESLKKFLDQYNNESEQMRYQQNGFGRGHDA	-46.54	45.2	HHHHHHHHHHHHHHHHHHHHHHHHCCCCCCC
740	EEELKKILEQINNEEEDMRYQQQGFGKGDSR	-46.52	51.6	HHHHHHHHHHHHHHHHHHHHHHHCCCCCCCC
273	EEELKKILEQLNNEQEDMRYQQNGFGKGDSR	-46.52	51.6	HHHHHHHHHHHHHHHHHHHHHHHCCCCCCCC
339	EEELKKLLEKINNEAEDIRYQQQGFGKGDSR	-46.51	58.1	HHHHHHHHHHHHHHHHHHHHHHHCCCCCCCC
152	EEELKKLLEKINNEAEDMRYQQQGFGKGDSR	-46.51	58.1	HHHHHHHHHHHHHHHHHHHHHHHCCCCCCCC
761	EEELKKLLEKLNNEQEDMRYQQQGFGKGDSR	-46.51	54.8	HHHHHHHHHHHHHHHHHHHHHHHCCCCCCCC
049	EEELRKLLEKINNEAEDIRYQQNGFGKGDSR	-46.51	54.8	HHHHHHHHHHHHHHHHHHHHHHHCCCCCCCC
186	EEELRKLLEKINNEAEDMRYQQQGFGKGDSR	-46.51	54.8	HHHHHHHHHHHHHHHHHHHHHHHCCCCCCCC
899	EEELRKLLEKINNEAEDMRYQREGFGKGDSR	-46.51	54.8	HHHHHHHHHHHHHHHHHHHHHHHHCCCCCCC
004	EEELRKLLEKLNNEEEDMKYQQGGFGKGDSR	-46.51	51.6	HHHHHHHHHHHHHHHHHHHHHHCCCCCCCCC
691	EEELRKLLEKLNNEKEDMSYQQQGFGKGDSR	-46.51	51.6	HHHHHHHHHHHHHHHHHHHHHHHCCCCCCCC
050	EEELKKFLEQFNQEYEDMFYQQKGFGKGHDA	-46.50	54.8	HHHHHHHHHHHHHHHHHHHHHHHHCCCCCCC
738	EEELKKFLEQLNQEEEDMRYQQQGFGKGHSA	-46.48	48.4	HHHHHHHHHHHHHHHHHHHHHHHHCCCCCCC
252	EEEAKKMLEKINNEEEDIRYQQQGFGKGHDA	-46.47	51.6	HHHHHHHHHHHHHHHHHHHHHHHHCCCCCCC
521	EEEARKFLEKFNNEYEDMFYQQKGFGKGDSR	-46.45	64.5	HHHHHHHHHHHHHHHHHHHHHHCCCCCCCCC
507	EEEARKFLEKYNNEEEDMRYQRQGFGKGDDR	-46.45	58.1	HHHHHHHHHHHCHHHHHHHHHHHCCCCCCCC
377	EEEARKFLEKYNNESEDIRYQQQGFGKGDSR	-46.45	58.1	HHHHHHHHHHHCHHHHHHHHHHHCCCCCCCC
175	EEELKKFLEKYNNEAEDIYYQQNGFGKGDSR	-46.45	61.3	HHHHHHHHHHHHHHHHHHHHHHHCCCCCCCC
891	EEELKKFLEKYNNESEDIRYQQNGFGKGDSR	-46.45	58.1	HHHHHHHHHHHHCHHHHHHHHHHCCCCCCCC
531	EEELKKFLEKYNNEYEDMFYQQKGFGKGDSR	-46.45	61.3	HHHHHHHHHHHHHHHHHHHHHHCCCCCCCCC
417	EEEARKFLEQFNNEYEDMFYQQKGFGKGHDA	-46.44	54.8	HHHHHHHHHHHHHHHHHHHHHHHHCCCCCCC
452	EEEARKFLEQYNNEEEDMRYQRQGFGKGHDA	-46.44	48.4	HHHHHHHHHHHCHHHHHHHHHHHHCCCCCCC
318	EEELKKFLEQYNNEAEDMRYQQQGFGKGHDA	-46.44	51.6	HHHHHHHHHHHHHHHHHHHHHHHHCCCCCCC
225	EEEWRKFLEQLNNEEEDMRYQQQGFGKGHSA	-46.43	45.2	HHHHHHHHHHHHHHHHHHHHHHHHCCCCCCC
023	EEEWRKFLEQLNNEEEDMRYQREGFGKGHDA	-46.43	45.2	HHHHHHHHHHHHHHHHHHHHHHHHHCCCCCC
370	EEEAKKFLEQFNNEYEDMFYQQKGFGKGHSA	-46.42	58.1	HHHHHHHHHHHHHHHHHHHHHHHHCCCCCCC
586	EEEAKKFLEQFNNEYEDMFYQQQGFGKGHDA	-46.42	58.1	HHHHHHHHHHHHHHHHHHHHHHHHCCCCCCC
963	EEEAKKFLEQYNNEAEDIRYQQGGFGKGHDY	-46.42	54.8	HHHHHHHHHHHHHHHHHHHHHHCCCCCCCCC
905	EEEAKKFLEQYNNEAEDIYYQQNGFGKGHDA	-46.42	54.8	HHHHHHHHHHHHHHHHHHHHHHHCCCCCCCC
832	EEEAKKFLEQYNNESEDIRYQQNGFGKGHDA	-46.42	51.6	HHHHHHHHHHHHHHHHHHHHHHHCCCCCCCC
222	EEEAKKFLEQYNNESEDIRYQQQGFGKGHDA	-46.42	51.6	HHHHHHHHHHHHHHHHHHHHHHHHCCCCCCC
937	EEEAKKFLEQYNNEYEDMFYQQQGFGKGHDA	-46.42	54.8	HHHHHHHHHHHHHHHHHHHHHHHHCCCCCCC
919	EEEARKFLEQFNNEKEDMRYQQQGFGKGHDA	-46.42	51.6	HHHHHHHHHHHHHHHHHHHHHHHHCCCCCCC
932	EEEARKFLEQLNNEEEDMQYQRSGFGKGHDA	-46.42	51.6	HHHHHHHHHHHHHHHHHHHHHHHHCCCCCCC
637	EEEARKFLEQLNNEEEDMRYQQYGFGKGHDA	-46.42	48.4	HHHHHHHHHHHHHHHHHHHHHHHCCCCCCCC
149	EEEARKFLEQYNNEAEDIYYQQNGFGKGHDA	-46.42	51.6	HHHHHHHHHHHHHHHHHHHHHHHCCCCCCCC
555	EEEARKFLEQYNNEAEDIYYQQNGFGKGHSA	-46.42	51.6	HHHHHHHHHHHHHHHHHHHHHHHCCCCCCCC
000	EEEARKFLEQYNNEAEDMRYQQNGFGKGHDA	-46.42	51.6	HHHHHHHHHHHHHHHHHHHHHHHHCCCCCCC
112	EEEARKFLEQYNNEAEDMRYQQNGFGKGHSA	-46.42	51.6	HHHHHHHHHHHHHHHHHHHHHHHHCCCCCCC
317	EEEARKFLEQYNNEAEDMRYQQQGFGKGHDA	-46.42	51.6	HHHHHHHHHHHHHHHHHHHHHHHHCCCCCCC
621	EEEARKFLEQYNNEAEDMRYQQQGFGKGHSA	-46.42	51.6	HHHHHHHHHHHHHHHHHHHHHHHHCCCCCCC
781	EEEARKFLEQYNNEAEDMRYQRQGFGKGHDA	-46.42	51.6	HHHHHHHHHHHHHHHHHHHHHHHHCCCCCCC
081	EEEARKFLEQYNNEEEDMFYQQKGFGKGHDA	-46.42	51.6	HHHHHHHHHHHHHHHHHHHHHHHHCCCCCCC
121	EEEARKFLEQYNNEEEDMKYQQEGFGKGHDA	-46.42	48.4	HHHHHHHHHHHHHHHHHHHHHHHHHCCCCCC
047	EEEARKFLEQYNNEEEDMRYQQQGFGKGHSA	-46.42	48.4	HHHHHHHHHHHHHHHHHHHHHHHHCCCCCCC
879	EEEARKFLEQYNNEEEDMRYQREGFGKGHDA	-46.42	48.4	HHHHHHHHHHHCHHHHHHHHHHHHHCCCCCC
881	EEEARKFLEQYNNESEDIRYQQGGFGKGHSA	-46.42	48.4	HHHHHHHHHHHHHHHHHHHHHCCCCCCCCCC
294	EEEARKFLEQYNNESEDIRYQQNGFGKGHDA	-46.42	48.4	HHHHHHHHHHHHHHHHHHHHHHHCCCCCCCC
463	EEEARKFLEQYNNESEDMRYQQNGFGKGHDA	-46.42	48.4	HHHHHHHHHHHHHHHHHHHHHHHHCCCCCCC
839	EEEARKFLEQYNNESEDMRYQQNGFGKGHSA	-46.42	48.4	HHHHHHHHHHHHHHHHHHHHHHHHCCCCCCC
772	EEEARKFLEQYNNESEDMRYQQQGFGKGHDA	-46.42	48.4	HHHHHHHHHHHHHHHHHHHHHHHHCCCCCCC
525	EEEARKFLEQYNNEYEDMEYQRSGFGKGHDA	-46.42	51.6	HHHHHHHHHHHHHHHHHHHHHHHHCCCCCCC

732	EEEARKFLEQYNNEYEDMFYQQKGFGKGHDA	-46.42	51.6	HHHHHHHHHHHHHHHHHHHHHHHHCCCCCCC
235	EEELKKFLEQFNNEAEDMRYQQQGFGKGHDA	-46.42	54.8	HHHHHHHHHHHHHHHHHHHHHHHHCCCCCCC
341	EEELKKFLEQFNNEEEDMRYQREGFGKGHDA	-46.42	51.6	HHHHHHHHHHHHHHHHHHHHHHHHHCCCCCC
153	EEELKKFLEQFNNEYEDMFYQKEGFGKGHDA	-46.42	54.8	HHHHHHHHHHHHHHHHHHHHHHHHCCCCCCC
166	EEELKKFLEQFNNEYEDMFYQQKGFGKGHDA	-46.42	54.8	HHHHHHHHHHHHHHHHHHHHHHHHCCCCCCC
111	EEELKKFLEQFNNEYEDMFYQQKGFGKGHSA	-46.42	54.8	HHHHHHHHHHHHHHHHHHHHHHHHCCCCCCC
650	EEELKKFLEQFNNEYEDMYYQQKGFGKGHDA	-46.42	51.6	HHHHHHHHHHHHHHHHHHHHHHHHCCCCCCC
274	EEELKKFLEQLNNEEEDMRYQQQGFGKGHDA	-46.42	48.4	HHHHHHHHHHHHHHHHHHHHHHHHCCCCCCC
600	EEELKKFLEQLNNEEEDMRYQQQGFGKGHSA	-46.42	48.4	HHHHHHHHHHHHHHHHHHHHHHHHCCCCCCC
688	EEELKKFLEQLNNEEEDMRYQQYGFGKGHDA	-46.42	48.4	HHHHHHHHHHHHHHHHHHHHHHHCCCCCCCC
103	EEELKKFLEQLNNEEEDMRYQREGFGKGHDA	-46.42	48.4	HHHHHHHHHHHHHHHHHHHHHHHHHCCCCCC
516	EEELKKFLEQYNNEAEDIRYQQNGFGKGHDA	-46.42	51.6	HHHHHHHHHHHHHHHHHHHHHHHHCCCCCCC
258	EEELKKFLEQYNNEAEDIYYQQNGFGKGHDA	-46.42	51.6	HHHHHHHHHHHHHHHHHHHHHHHCCCCCCCC
033	EEELKKFLEQYNNEAEDIYYQQQGFGKGHDA	-46.42	51.6	HHHHHHHHHHHHHHHHHHHHHHHHCCCCCCC
653	EEELKKFLEQYNNEAEDMRYQQEGFGKGHDA	-46.42	51.6	HHHHHHHHHHHHHHHHHHHHHHHHHCCCCCC
763	EEELKKFLEQYNNEAEDMRYQQGGFGKGHDA	-46.42	51.6	HHHHHHHHHHHHHHHHHHHHHHCCCCCCCCC
475	EEELKKFLEQYNNEAEDMRYQQNGFGKGHDA	-46.42	51.6	HHHHHHHHHHHHHHHHHHHHHHHHCCCCCCC
288	EEELKKFLEQYNNEAEDMRYQRYGFGKGHDA	-46.42	51.6	HHHHHHHHHHHHHHHHHHHHHHHCCCCCCCC
022	EEELKKFLEQYNNEEEDMKYQQEGFGKGHSA	-46.42	48.4	HHHHHHHHHHHHHHHHHHHHHHHHHCCCCCC
043	EEELKKFLEQYNNEEEDMRYQREGFGKGHDA	-46.42	48.4	HHHHHHHHHHHCHHHHHHHHHHHHHCCCCCC
594	EEELKKFLEQYNNESEDMRYQQNGFGKGHDA	-46.42	48.4	HHHHHHHHHHHHHHHHHHHHHHHHCCCCCCC
508	EEELKKFLEQYNNESEDMRYQQQGFGKGHDA	-46.42	48.4	HHHHHHHHHHHHHHHHHHHHHHHHCCCCCCC
800	EEELKKFLEQYNNEYEDMEYQRSGFGKGHDA	-46.42	51.6	HHHHHHHHHHHHHHHHHHHHHHHHCCCCCCC
933	EEELKKFLEQYNNEYEDMFYQQKGFGKGHDA	-46.42	51.6	HHHHHHHHHHHHHHHHHHHHHHHHCCCCCCC
071	EEEWKKFIEQYNNEAEDIYYQQNGFGKGHDA	-46.42	48.4	HHHHHHHHHHHHHHHHHHHHHHHCCCCCCCC
070	EQEARKFLEQFNNEQEDMTYQKEGFGKGHDA	-46.42	48.4	HHHHHHHHHHHHHHHHHHHHHHHHCCCCCCC
102	EQEARKFLEQYNNEAEDMRYQQQGFGKGHDA	-46.42	48.4	HHHHHHHHHHHHHHHHHHHHHHHHCCCCCCC
533	EQEARKFLEQYNNEYEDMFYQKEGFGKGHDA	-46.42	48.4	HHHHHHHHHHHHHHHHHHHHHHHHCCCCCCC
334	EEELKKFLEQFNNEYEDMYFQQKGFGKGKDA	-46.39	48.4	HHHHHHHHHHHHHHHHHHHHHHHHCCCCCCC
663	EEELRKLLDQINQEEEQMRYQREGFGQGHDA	-46.39	38.7	HHHHHHHHHHHHHHHHHHHHHHHHHCCCCCC
038	EEELKKLLEKINNEEEDRRYQQYGFGKGHDA	-46.36	48.4	HHHHHHHHHHHHHHHHHHHHHHHCCCCCCCC
303	EEELKKFLQKLNEEEEQMRYQQYGFGQGHDA	-46.35	45.2	HHHHHHHHHHHHHHHHHHHHHHHCCCCCCCC
491	EEELRKLLEQINNEAEDMRYQQQGFGKGDSR	-46.27	51.6	HHHHHHHHHHHHHHHHHHHHHHHCCCCCCCC
366	EEELRKLLEQINNEEEDMRYQQQGFGKGDSR	-46.27	48.4	HHHHHHHHHHHHHHHHHHHHHHHCCCCCCCC
188	EEELKKLLEQINNEAEDMRYQQNGFGKGDSR	-46.25	54.8	HHHHHHHHHHHHHHHHHHHHHHHCCCCCCCC
141	EEELKKLLEQLNNEEEDMKYQQQGFGKGDSR	-46.25	51.6	HHHHHHHHHHHHHHHHHHHHHHHCCCCCCCC
975	EEELKKLLEQLNNEQEDMRYQQNGFGKGDSR	-46.25	51.6	HHHHHHHHHHHHHHHHHHHHHHHCCCCCCCC
764	EEELKKLLEQLNNEQEDMRYQQQGFGKGDSR	-46.25	51.6	HHHHHHHHHHHHHHHHHHHHHHHCCCCCCCC
896	EEELKKLLEQLNNEQEDMRYQREGFGKGDSR	-46.25	51.6	HHHHHHHHHHHHHHHHHHHHHHHHCCCCCCC
233	EEELRKLLEQLNNEQEDMRYQQNGFGKGDSR	-46.25	48.4	HHHHHHHHHHHHHHHHHHHHHHHCCCCCCCC
570	EEELKKFLEKFNQEQEDMTYQRHGFGKGDSR	-46.24	61.3	HHHHHHHHHHHHHHHHHHHHHHHCCCCCCCC
774	EEELKKFLEKYNQEAEDIRYQQQGFGKGDDR	-46.24	61.3	HHHHHHHHHHHHHHHHHHHHHHHCCCCCCCC
606	EEELKKFLEKYNQEAEDMRYQKEGFGKGDSR	-46.24	61.3	HHHHHHHHHHHHHHHHHHHHHHHHCCCCCCC
351	EEELRKFLEQYNNESEDMRYQQQGFGKGHSA	-46.21	45.2	HHHHHHHHHHHHHHHHHHHHHHHHCCCCCCC
389	EEEWRKILEQLNNEQEDMRYQQNGFGKGDSR	-46.19	48.4	HHHHHHHHHHHHHHHHHHHHHHHCCCCCCCC
782	EEELKKFLEQYNNDAEDMRYQRQGFGKGHDA	-46.13	48.4	HHHHHHHHHHHHHHHHHHHHHHHHCCCCCCC
950	EEELKKFLEQYNQESEDIRYQQNGFGKGHDA	-46.12	48.4	HHHHHHHHHHHHHHHHHHHHHHHCCCCCCCC
655	EEELKKILEKINNEAEQIRYQQNGFGQGHDA	-46.11	45.2	HHHHHHHHHHHHHHHHHHHHHHHHCCCCCCC
424	EEELKKILEKLNNEQEQMQYQRAGFGQGHDA	-46.11	41.9	HHHHHHHHHHHHHHHHHHHHHHHHHCCCCCC
394	EEELKKLLDQINNEAEQIRYQQNGFGQGHDA	-46.09	45.2	HHHHHHHHHHHHHHHHHHHHHHHHCCCCCCC
513	EESLKKLLEQINQEAEDMFYQQKGFGKGHDA	-46.06	51.6	HHHHHHHHHHHHHHHHHHHHHHHHCCCCCCC
092	EEELKKLLEQINQEEEDMRYQQQGFGKGDSR	-46.04	51.6	HHHHHHHHHHHHHHHHHHHHHHHCCCCCCCC
115	EEELKKLLEQLNQEEEDMKYQQNGFGKGDSR	-46.04	51.6	HHHHHHHHHHHHHHHHHHHHHHHCCCCCCCC
260	EEELRKLLEQINQEEEDMRYQQYGFGKGDSR	-46.04	48.4	HHHHHHHHHHHHHHHHHHHHHHHCCCCCCCC
056	EEELRKLLEQLNQEEEDMKYQQNGFGKGDSR	-46.04	48.4	HHHHHHHHHHHHHHHHHHHHHHHCCCCCCCC
061	EEEARKMLEQINNEEEDMRYQREGFGKGHDA	-46.03	45.2	HHHHHHHHHHHHHHHHHHHHHHHHHCCCCCC
079	EEEARKFLEQYNQEEEDMRYQREGFGKGDSR	-45.98	54.8	HHHHHHHHHHHHHHHHHHHHHHHHCCCCCCC
372	EEELKKFLEKLNNEEEDRRYQQYGFGKGHDA	-45.98	51.6	HHHHHHHHHHHHHHHHHHHHHHHCCCCCCCC
505	EEEWRKFLEKYNNEAEDIRYQQQGFGKGDSR	-45.95	58.1	HHHHHHHHHHHHHHHHHHHHHHHCCCCCCCC
138	EEEAKKFLEKFNNEEEDMRYQREGFGKGDSR	-45.94	64.5	HHHHHHHHHHHCHHHHHHHHHHHHCCCCCCC
747	EEEARKFLEKYNNEAEDIRYQQNGFGKGDSR	-45.94	61.3	HHHHHHHHHHHHHHHHHHHHHHHCCCCCCCC
481	EEELKKFLEKLNNEKEDMEYQKQGFGKGDSR	-45.94	58.1	HHHHHHHHHHHHHHHHHHHHHHHCCCCCCCC
574	EEELKKFLEKYNNEAEDIRYQQNGFGKGDSR	-45.94	61.3	HHHHHHHHHHHHHHHHHHHHHHHCCCCCCCC
719	EEELKKFLEKYNNEAEDIRYQQQGFGKGDSR	-45.94	61.3	HHHHHHHHHHHHHHHHHHHHHHHCCCCCCCC
631	EEELKKFLEKYNNEAEDIYYQQQGFGKGDSR	-45.94	61.3	HHHHHHHHHHHHHHHHHHHHHHHCCCCCCCC
717	EEELKKFLEKYNNEAEDMRYQQQGFGKGDSR	-45.94	61.3	HHHHHHHHHHHHHHHHHHHHHHHCCCCCCCC
212	EEELKKILEKLNQEQEQIRYQQNGFGQGHDA	-45.88	41.9	HHHHHHHHHHHHHHHHHHHHHHHHCCCCCCC

018	EEELKKFLEKYNNEYERMFFQQKGFGKGKDA	-45.88	48.4	HHHHHHHHHHHHHHHHHHHHHHHHHCCCCCCCC
474	EEELRKLLDKLNQEQEQMSYQQQGFGQGDSR	-45.87	48.4	HHHHHHHHHHHHHHHHHHHHHHHHHCCCCCCCC
416	EEEARKFLEQFNNEYEDMYYQQQGFGKGHSA	-45.82	51.6	HHHHHHHHHHHHHHHHHHHHHHHHHHCCCCCCC
028	EEEARKFLEKFNNEEEDRSYQQQGFGKGHDA	-45.79	54.8	HHHHHHHHHHHHHHHHHHHHHHHHHCCCCCCCC
037	EEEARKFLEKFNNEYEDRFYQQKGFGKGHDA	-45.79	58.1	HHHHHHHHHHHHHHHHHHHHHHHHHCCCCCCCC
257	EEELKKFLEKYNNEAEDRRYQKEGFGKGHDA	-45.79	54.8	HHHHHHHHHHHHHHHHHHHHHHHHHHCCCCCCC
309	EESLKKLLEQINNEEEDMRYQQYGFGKGHDA	-45.78	45.2	HHHHHHHHHHHHHHHHHHHHHHHHHCCCCCCCC
554	EEELKKLLEQINNEAEDMRYQQQGFGKGDSR	-45.74	54.8	HHHHHHHHHHHHHHHHHHHHHHHHHCCCCCCCC
269	EEELKKLLEQINNEEEDIRYQQQGFGKGDSR	-45.74	51.6	HHHHHHHHHHHHHHHHHHHHHHHHHCCCCCCCC
589	EEELKKLLEQINNEEEDMRYQQQGFGKGDSR	-45.74	51.6	HHHHHHHHHHHHHHHHHHHHHHHHHCCCCCCCC
861	EEELKKLLEQINNEEEDMRYQREGFGKGDSR	-45.74	51.6	HHHHHHHHHHHHHHHHHHHHHHHHHHCCCCCCC
206	EEELRKLLEQINNEKEDMEYQRAGFGKGDSR	-45.74	48.4	HHHHHHHHHHHHHHHHHHHHHHHHHHCCCCCCC
177	EEELRKLLEQLNNEQEDMRYQQQGFGKGDSR	-45.74	48.4	HHHHHHHHHHHHHHHHHHHHHHHHHCCCCCCCC
644	EEELKKLLEAINNREEDMRYQQYGFGKGHDA	-45.72	41.9	HHHHHHHHHHHHHHHHHHHHHHHHHCCCCCCCC
443	EEELKKFLEQFNNEYEDMFYQQKGFGKGDSR	-45.70	61.3	HHHHHHHHHHHHHHHHHHHHHHHHHCCCCCCCC
221	EEEARKFLEQFNNEYEDMFYQQKGFGKGDSR	-45.69	61.3	HHHHHHHHHHHHHHHHHHHHHHHHHCCCCCCCC
182	EEEARKFLEQLNNEEEDMRYQQQGFGKGDSR	-45.69	54.8	HHHHHHHHHHHHHHHHHHHHHHHHHCCCCCCCC
997	EEEARKFLEQYNNEAEDIRYQQGGFGKGDSR	-45.69	58.1	HHHHHHHHHHHHHHHHHHHHHHHHCCCCCCCCC
348	EEEARKFLEQYNNEAEDMRYQQQGFGKGDSR	-45.69	58.1	HHHHHHHHHHHHHHHHHHHHHHHHHCCCCCCCC
660	EEEARKFLEQYNNEEEDMRYQRQGFGKGDSR	-45.69	54.8	HHHHHHHHHHHCHHHHHHHHHHHHHCCCCCCCC
305	EEELKKFLEQLNNEEEDIRYQQQGFGKGDSR	-45.69	54.8	HHHHHHHHHHHHHHHHHHHHHHHHHCCCCCCCC
450	EEELKKFLEQYNNEAEDMRYQRYGFGKGDSR	-45.69	58.1	HHHHHHHHHHHHHHHHHHHHHHHHHCCCCCCCC
939	EEEWKKFIEQYNNEEEDMYYQQQGFGKGDSR	-45.69	51.6	HHHHHHHHHHHHHHHHHHHHHHHHHCCCCCCCC
867	EEELKKLLQQLNEEEEQMQYQRAGFGQGHDA	-45.68	38.7	HHHHHHHHHHHHHHHHHHHHHHHHHHCCCCCCC
246	EEELKKFLEQFNNVYEDMFYQQKGFGKGDSR	-45.63	58.1	HHHHHHHHHHHHHHHHHHHHHHHHCCCCCCCCC
497	EEELKKLLDQINNEEEQMRYQQQGFGQGHDA	-45.62	41.9	HHHHHHHHHHHHHHHHHHHHHHHHHCCCCCCCC
378	EEELKKFLEKLNNEEEDRRYQQQGFGKGHDA	-45.59	51.6	HHHHHHHHHHHHHHHHHHHHHHHHHHCCCCCCC
238	EEELRKLLEKINQEAEQIRYQQNGFGQGHSA	-45.57	41.9	HHHHHHHHHHHHHHHHHHHHHHHHHHCCCCCCC
696	EEEARKMLEKINNEEEDIRYQQQGFGKGDSR	-45.55	54.8	HHHHHHHHHHHHHHHHHHHHHHHHHCCCCCCCC
189	EEEARKFLQQYNNEYEDMFYQQKGFGKGHDA	-45.55	51.6	HHHHHHHHHHHHHHHHHHHHHHHHHHCCCCCCC
790	EEELRKFLEQFNQEYEDMFYQQQGFGKGDSR	-45.49	58.1	HHHHHHHHHHHHHHHHHHHHHHHHHCCCCCCCC
888	EEEARKFLEQYNQEAEDMRYQQQGFGKGDSR	-45.47	58.1	HHHHHHHHHHHHHHHHHHHHHHHHHCCCCCCCC
412	EEEARKFLEQYNQEEEDMFYQQKGFGKGDSR	-45.47	58.1	HHHHHHHHHHHHHHHHHHHHHHHHHCCCCCCCC
266	EEEARKFLEQYNQEEEDMRYQQQGFGKGDSR	-45.47	54.8	HHHHHHHHHHHHHHHHHHHHHHHHHCCCCCCCC
654	EEELKKFLEQLNQEEEDMRYQQQGFGKGDSR	-45.47	54.8	HHHHHHHHHHHHHHHHHHHHHHHHHCCCCCCCC
232	EEELKKFLEQYNQEAEDMRYQQNGFGKGDSR	-45.47	58.1	HHHHHHHHHHHHHHHHHHHHHHHHHCCCCCCCC
870	EEELKKFLEQYNQEEEDMRYQREGFGKGDSR	-45.47	54.8	HHHHHHHHHHHHHHHHHHHHHHHHHHCCCCCCC
528	EQEARKFLEQYNQEEEDMRYQREGFGKGDSR	-45.47	51.6	HHHHHHHHHHHHHHHHHHHHHHHHHHCCCCCCC
661	EEELRKLLEQINQEEEDMRYQQQGFGKGDSR	-45.44	48.4	HHHHHHHHHHHHHHHHHHHHHHHHHCCCCCCCC
195	EEELKKLLEKINQEAEQIRYQQNGFGQGHDA	-45.39	45.2	HHHHHHHHHHHHHHHHHHHHHHHHHCCCCCCCC
032	EEELKKLLEKINQEEEQIRYQQQGFGQGHDA	-45.39	41.9	HHHHHHHHHHHHHHHHHHHHHHHHHCCCCCCCC
713	EEELKKLLEKINQEEEQMRYQKEGFGQGHDA	-45.39	41.9	HHHHHHHHHHHHHHHHHHHHHHHHHHCCCCCCC
168	EEELKKLLEKLNQEKEQIRYQQNGFGQGHDA	-45.39	41.9	HHHHHHHHHHHHHHHHHHHHHHHHHCCCCCCCC
541	EEELKKLLEKLNQEKEQMSYQQQGFGQGHSA	-45.39	41.9	HHHHHHHHHHHHHHHHHHHHHHHHHCCCCCCCC
119	EEELRKLLEKINQEAEQMRYQRYGFGQGHDA	-45.39	41.9	HHHHHHHHHHHHHHHHHHHHHHHHHCCCCCCCC
160	EEELRKLLEKINQEAEQMSYQQQGFGQGHSA	-45.39	41.9	HHHHHHHHHHHHHHHHHHHHHHHHHCCCCCCCC
864	EEELRKLLEKLNQEKEQMRYQQQGFGQGHDA	-45.39	38.7	HHHHHHHHHHHHHHHHHHHHHHHHHHCCCCCCC
346	EEELRKLLEKLNQEQEQMSYQQQGFGQGHDA	-45.39	38.7	HHHHHHHHHHHHHHHHHHHHHHHHHCCCCCCCC
060	EEEARKFLEKYNNEAEDMRYQRQGFGKGDSR	-45.35	61.3	HHHHHHHHHHHHHHHHHHHHHHHHHCCCCCCCC
679	EEELKKLLEKINNEAEQIRFQQNGFGQGKDA	-45.34	41.9	HHHHHHHHHHHHHHHHHHHHHHHHHHCCCCCCC
515	EEELRKLLEKIEQEREQITYQQNGFGQGHDA	-45.32	35.5	HHHHHHHHHHHHHHHHHHHHHHHHHCCCCCCCC
746	EEELRKLLEKLNNEQEQIRYQQNGFGQGHSA	-45.28	38.7	HHHHHHHHHHHHHHHHHHHHHHHHHHCCCCCCC
540	EEEARKFLEQLNNEEEQMRYQQYGFGQGHDA	-45.26	41.9	HHHHHHHHHHHHHHHHHHHHHHHHHCCCCCCCC
518	EEEARKFLEQYNQEAEDMRYQQQGFGKGDDR	-45.25	58.1	HHHHHHHHHHHHHHHHHHHHHHHHHCCCCCCCC
234	EEQARKFLEQYNNESEDMRYQQQGFGKGDSR	-45.25	58.1	HHHHHHHHHHHHHHHHHHHHHHHHHCCCCCCCC
673	EESLKKFLEQYNNESEDIRYQQNGFGKGHDA	-45.20	48.4	HHHHHHHHHHHHHHHHHHHHHHHHHCCCCCCCC
184	EEEAKKFLEQLNNEEEDMRYQQQGFGKGDSR	-45.18	58.1	HHHHHHHHHHHHHHHHHHHHHHHHHCCCCCCCC
853	EEEAKKFLEQLNNEEEDMRYQQYGFGKGDSR	-45.18	58.1	HHHHHHHHHHHHHHHHHHHHHHHHHCCCCCCCC
502	EEEARKFLEQLNNEEEDMRYQQNGFGKGDSR	-45.18	54.8	HHHHHHHHHHHHHHHHHHHHHHHHHCCCCCCCC
272	EEEARKFLEQLNNEEEDMRYQQYGFGKGDSR	-45.18	54.8	HHHHHHHHHHHHHHHHHHHHHHHHHCCCCCCCC
593	EEEARKFLEQYNNEAEDIRYQQNGFGKGDSR	-45.18	58.1	HHHHHHHHHHHHHHHHHHHHHHHHHCCCCCCCC
154	EEEARKFLEQYNNEAEDMRYQQNGFGKGDDR	-45.18	58.1	HHHHHHHHHHHHHHHHHHHHHHHHHCCCCCCCC
324	EEEARKFLEQYNNEEEDMFYQQKGFGKGDSR	-45.18	58.1	HHHHHHHHHHHHHHHHHHHHHHHHHCCCCCCCC
801	EEEARKFLEQYNNEEEDMRYQQEGFGKGDSR	-45.18	54.8	HHHHHHHHHHHHHHHHHHHHHHHHHHCCCCCCC
822	EEELKKFLEQFNNEYEDMFYQQKGFGKGDDR	-45.18	61.3	HHHHHHHHHHHHHHHHHHHHHHHHHCCCCCCCC
074	EEELKKFLEQLNNEEEDMRYQQYGFGKGDSR	-45.18	54.8	HHHHHHHHHHHHHHHHHHHHHHHHHCCCCCCCC
151	EEELKKFLEQYNNEAEDIYYQQNGFGKGDSR	-45.18	58.1	HHHHHHHHHHHHHHHHHHHHHHHHHCCCCCCCC

472	EEELKKFLEQYNNEAEDMRYQQGGFGKGDSR	-45.18	58.1	HHHHHHHHHHHHHHHHHHHHHHCCCCCCCCC
382	EEELKKFLEQYNNEAEDMRYQQNGFGKGDSR	-45.18	58.1	HHHHHHHHHHHHHHHHHHHHHHHCCCCCCCC
786	EEELKKFLEQYNNEAEDMRYQQQGFGKGDSR	-45.18	58.1	HHHHHHHHHHHHHHHHHHHHHHHCCCCCCCC
780	EEELKKFLEQYNNEAEDMRYQRQGFGKGDSR	-45.18	58.1	HHHHHHHHHHHHHHHHHHHHHHHCCCCCCCC
519	EEELKKFLEQYNNESEDIRYQQNGFGKGDSR	-45.18	54.8	HHHHHHHHHHHHHHHHHHHHHHHCCCCCCCC
838	EEELKKFLEQYNNEYEDMFYQQKGFGKGDSR	-45.18	58.1	HHHHHHHHHHHHHHHHHHHHHHCCCCCCCCC
304	EEELKKFLEQYNNESEDMRYQQQGFGKGDSR	-45.17	54.8	HHHHHHHHHHHHHHHHHHHHHHHCCCCCCCC
328	EEEARKFLEQYNQEAEDMRYQQNGFGKGDSR	-45.15	58.1	HHHHHHHHHHHHHHHHHHHHHHHCCCCCCCC
422	EEELRKLLEKINNEEEDRRYQQQGFGKGDSR	-45.11	51.6	HHHHHHHHHHHHHHHHHHHHHHHCCCCCCCC
179	EEELKKLLEKINNEAEQIRYQQNGFGQGHDA	-45.09	45.2	HHHHHHHHHHHHHHHHHHHHHHHHCCCCCCC
215	EEELKKLLEKINNEAEQITYQQNGFGQGHDA	-45.09	45.2	HHHHHHHHHHHHHHHHHHHHHHHCCCCCCCC
522	EEELKKLLEKINNEAEQITYQQNGFGQGHSA	-45.09	45.2	HHHHHHHHHHHHHHHHHHHHHHHCCCCCCCC
635	EEELKKLLEKINNEEEQMRYQQQGFGQGHDA	-45.09	41.9	HHHHHHHHHHHCHHHHHHHHHHHCCCCCCCC
172	EEELKKLLEKINNEQEQMSYQQQGFGQGHDA	-45.09	41.9	HHHHHHHHHHHHHHHHHHHHHHHCCCCCCCC
353	EEELKKLLEKLNNEEEQMKYQQNGFGQGHDA	-45.09	41.9	HHHHHHHHHHHCHHHHHHHHHHHHCCCCCCC
388	EEELKKLLEKLNNEEEQMKYQQQGFGQGHDA	-45.09	41.9	HHHHHHHHHHHCHHHHHHHHHHHCCCCCCCC
928	EEELKKLLEKLNNEEEQMKYQRYGFGQGHDA	-45.09	41.9	HHHHHHHHHHHCHHHHHHHHHHHCCCCCCCC
901	EEELKKLLEKLNNEKEQITYQQNGFGQGHDA	-45.09	41.9	HHHHHHHHHHHHHHHHHHHHHHHCCCCCCCC
549	EEELKKLLEKLNNEQEQIRYQQGGFGQGHDF	-45.09	41.9	HHHHHHHHHHHHHHHHHHHHHHCCCCCCCCC
162	EEELKKLLEKLNNEQEQIRYQQNGFGQGHDA	-45.09	41.9	HHHHHHHHHHHHHHHHHHHHHHHHCCCCCCC
941	EEELKKLLEKLNNEQEQMRYQQNGFGQGHDA	-45.09	41.9	HHHHHHHHHHHHHHHHHHHHHHHHCCCCCCC
509	EEELKKLLEKLNNEQEQMRYQRQGFGQGHDA	-45.09	41.9	HHHHHHHHHHHHHHHHHHHHHHHHCCCCCCC
331	EEELRKLLEKINNEAEQIRYQQGGFGQGHDF	-45.09	41.9	HHHHHHHHHHHHHHHHHHHHHHCCCCCCCCC
478	EEELRKLLEKINNEAEQIRYQQNGFGQGHDA	-45.09	41.9	HHHHHHHHHHHHHHHHHHHHHHHHCCCCCCC
306	EEELRKLLEKINNEEEQIRYQQQGFGQGHDA	-45.09	38.7	HHHHHHHHHHHCHHHHHHHHHHHCCCCCCCC
700	EEELRKLLEKINNEKEQMEYQRAGFGQGHDA	-45.09	38.7	HHHHHHHHHHHHHHHHHHHHHHHHCCCCCCC
191	EEELRKLLEKLNNEEEQIKYQQNGFGQGHDA	-45.09	38.7	HHHHHHHHHHHCHHHHHHHHHHHHCCCCCCC
242	EEELRKLLEKLNNEEEQMKYQQNGFGQGHDA	-45.09	38.7	HHHHHHHHHHHCHHHHHHHHHHHHCCCCCCC
227	EEELRKLLEKLNNEEEQMKYQQQGFGQGHDA	-45.09	38.7	HHHHHHHHHHHCHHHHHHHHHHHCCCCCCCC
856	EEELRKLLEKLNNEKEQMSYQQQGFGQGHDA	-45.09	38.7	HHHHHHHHHHHHHHHHHHHHHHHCCCCCCCC
173	EEELRKLLEKLNNEQEQMRYQQGGFGQGHDF	-45.09	38.7	HHHHHHHHHHHHHHHHHHHHHHCCCCCCCCC
862	EEELRKLLEKLNNEQEQMRYQQQGFGQGHDA	-45.09	38.7	HHHHHHHHHHHHHHHHHHHHHHHCCCCCCCC
447	EEELRKLLEKLNNEQEQMRYQRQGFGQGHDA	-45.09	38.7	HHHHHHHHHHHHHHHHHHHHHHHHCCCCCCC
007	EEELKKFLEKYNQEAEQMRFQQQGFGQGKDA	-45.07	45.2	HHHHHHHHHHHHHHHHHHHHHHHCCCCCCCC
931	EEEARKFLEKYNNESEDIRYQQNGFGKGHDA	-45.04	51.6	HHHHHHHHHHHCCHHHHHHHHHHCCCCCCCC
051	EESLKKLLEQINNEEEDMRYQQQGFGKGDSR	-45.03	51.6	HHHHHHHHHHHHHHHHHHHHHHHCCCCCCCC
407	EESARKFLDKFNNEYEQMFYQKEGFGQGHDA	-45.02	54.8	HHHHHHHHHHHHHHHHHHHHHHHHCCCCCCC
595	EEELRKLLEKINQEAEEMRKQQQGFGSGKDA	-44.96	38.7	HHHHHHHHHHHHHHHHHHHHHHHHCCCCCCC
883	EEELKKLLEKLNQEQEQMRYQRQGFGQGHDA	-44.91	41.9	HHHHHHHHHHHHHHHHHHHHHHHHCCCCCCC
629	EEEARKFLEQYNNEEEDMYYQQKGFGKGDSR	-44.86	54.8	HHHHHHHHHHHHHHHHHHHHHHHCCCCCCCC
990	EEEARKFLEKFNQEAEQITYQQNGFGQGHDA	-44.82	51.6	HHHHHHHHHHHHHHHHHHHHHHHCCCCCCCC
658	EEEARKFLEKYNQESEQIRYQQNGFGQGHDA	-44.82	45.2	HHHHHHHHHHHHHHHHHHHHHHHCCCCCCCC
599	EEEARKFLEKYNQESEQIRYQQQGFGQGHDA	-44.82	45.2	HHHHHHHHHHHHHHHHHHHHHHHHCCCCCCC
369	EEELKKFLEKFNQEYEQMFYQQKGFGQGHDA	-44.82	51.6	HHHHHHHHHHHHHHHHHHHHHHHHCCCCCCC
802	EEELKKFLEKLNQEEEQIRYQQQGFGQGHSA	-44.82	45.2	HHHHHHHHHHHHHHHHHHHHHHHHCCCCCCC
503	EEELKKFLEKLNQEEEQMRYQQQGFGQGHDA	-44.82	45.2	HHHHHHHHHHHHHHHHHHHHHHHCCCCCCCC
851	EEELKKFLEKYNQEAEQIYYQQNGFGQGHDA	-44.82	48.4	HHHHHHHHHHHHHHHHHHHHHHHCCCCCCCC
205	EEELKKFLEKYNQEAEQLRYQQKGFGQGHDA	-44.82	51.6	HHHHHHHHHHHHHHHHHHHHHHHHCCCCCCC
456	EEELKKFLEKYNQESEQIRYQQNGFGQGHDA	-44.82	45.2	HHHHHHHHHHHHHHHHHHHHHHHCCCCCCCC
217	EEEARKMLEQINNEEEDMRYQQYGFGKGDSR	-44.80	51.6	HHHHHHHHHHHHHHHHHHHHHHHCCCCCCCC
110	EEELKKLLEKINQEEEQMRYQQYGFGQGHDA	-44.79	41.9	HHHHHHHHHHHHHHHHHHHHHHHCCCCCCCC
985	EEEARKMLEQINNEAEDMRYQQNGFGKGDSR	-44.78	54.8	HHHHHHHHHHHHHHHHHHHHHHHCCCCCCCC
873	EEEARKFLEKFNNEYEQMFYQQKGFGQGHSA	-44.71	51.6	HHHHHHHHHHHHHHHHHHHHHHHHCCCCCCC
500	EEELRKILEQINQEEEQMRYQQYGFGQGDSR	-44.67	41.9	HHHHHHHHHHHHHHHHHHHHHHHCCCCCCCC
672	EQEAREFLKQFNEEYEDMFYQQKGFGKGHDA	-44.65	51.6	HHHHHHHHHHHHHHHHHHHHHHHHCCCCCCC
596	EEELRKILEQINNEEEQMRYQQQGFGQGHSA	-44.63	35.5	HHHHHHHHHHHCHHHHHHHHHHHCCCCCCCC
477	EEELKKLLEQINQEAEQMRYQKEGFGQGHDA	-44.62	41.9	HHHHHHHHHHHHHHHHHHHHHHHHCCCCCCC
542	EEELKKLLEQINQEAEQMRYQQNGFGQGHDA	-44.62	41.9	HHHHHHHHHHHHHHHHHHHHHHHCCCCCCCC
445	EEELKKLLEQINQEAEQMRYQQQGFGQGHDA	-44.62	41.9	HHHHHHHHHHHHHHHHHHHHHHHCCCCCCCC
760	EEELKKLLEQLNQEKEQMSYQRYGFGQGHDA	-44.62	38.7	HHHHHHHHHHHHHHHHHHHHHHHCCCCCCCC
249	EEELRKLLEQINQEAEQMRYQQNGFGQGHDA	-44.62	38.7	HHHHHHHHHHHHHHHHHHHHHHHCCCCCCCC
434	EEELRKLLEQINQEEEQMRYQREGFGQGHDA	-44.62	35.5	HHHHHHHHHHHHHHHHHHHHHHHHHCCCCCC
677	EEELRKLLEQLNQEEEQMKYQQQGFGQGHDA	-44.62	35.5	HHHHHHHHHHHHHHHHHHHHHHHCCCCCCCC
293	EEELKKLLEQINQEEEQMRYQREGFGQGHDA	-44.60	38.7	HHHHHHHHHHHHHHHHHHHHHHHHHCCCCCC
639	EEEARKFLEQFNNEEEDMRYQQQGFGKGDSR	-44.58	58.1	HHHHHHHHHHHHHHHHHHHHHHHCCCCCCCC
752	EEELRKFLEKYNNEAEDRRYQQQGFGKGDSR	-44.55	58.1	HHHHHHHHHHHHHHHHHHHHHHHCCCCCCCC
583	EEELKKFLEQLNNDKEQMEYQRAGFGQGHDA	-44.54	38.7	HHHHHHHHHHHHHHHHHHHHHHHHCCCCCCC

868	EEEAKKFLEKYNNEYEQMEYQRTGRGQGHDF	-44.53	48.4	HHHHHHHHHHHHHHHHHHHHHHHHHCCCCCCCC
840	EEEARKFLEKFNNEAEQMQYQRAGFGQGHDA	-44.53	51.6	HHHHHHHHHHHHHHHHHHHHHHHHHHCCCCCCC
470	EEEARKFLEKFNNEEEQMRYQQEGFGQGHSA	-44.53	48.4	HHHHHHHHHHHCHHHHHHHHHHHHHHHCCCCCC
118	EEEARKFLEKFNNEYEQMFYQQKGFGQGHDA	-44.53	51.6	HHHHHHHHHHHHHHHHHHHHHHHHHHCCCCCCC
607	EEEARKFLEKLNNEEEQMRYQQQGFGQGHDA	-44.53	45.2	HHHHHHHHHHHHHHHHHHHHHHHHHCCCCCCCC
165	EEEARKFLEKLNNEEEQMRYQQQGFGQGHSA	-44.53	45.2	HHHHHHHHHHHHHHHHHHHHHHHHHCCCCCCCC
039	EEEARKFLEKLNNEEEQMRYQQYGFGQGHDA	-44.53	45.2	HHHHHHHHHHHHHHHHHHHHHHHHHCCCCCCCC
414	EEEARKFLEKYNNEAEQIRYQQNGFGQGHDA	-44.53	48.4	HHHHHHHHHHHHHHHHHHHHHHHHHHCCCCCCC
087	EEEARKFLEKYNNEAEQIYYQQNGFGQGHDA	-44.53	48.4	HHHHHHHHHHHHHHHHHHHHHHHHHCCCCCCCC
548	EEEARKFLEKYNNEEEQMFYQQKGFGQGHDA	-44.53	48.4	HHHHHHHHHHHCHHHHHHHHHHHHHHCCCCCCC
903	EEEARKFLEKYNNESEQIRYQQNGFGQGHDA	-44.53	45.2	HHHHHHHHHHHCHHHHHHHHHHHHHCCCCCCCC
633	EEEARKFLEKYNNEYEQIFYQQKGFGQGHDA	-44.53	48.4	HHHHHHHHHHHHHHHHHHHHHHHHHHCCCCCCC
678	EEELKKFLEKFNNEEEQMSYQQQGFGQGHDA	-44.53	48.4	HHHHHHHHHHHCHHHHHHHHHHHHHCCCCCCCC
829	EEELKKFLEKFNNEYEQIFYQQKGFGQGHDA	-44.53	51.6	HHHHHHHHHHHHHHHHHHHHHHHHHHCCCCCCC
956	EEELKKFLEKFNNEYEQMFYQQKGFGQGHDA	-44.53	51.6	HHHHHHHHHHHHHHHHHHHHHHHHHHCCCCCCC
493	EEELKKFLEKFNNEYEQMFYQQKGFGQGHSA	-44.53	51.6	HHHHHHHHHHHHHHHHHHHHHHHHHHCCCCCCC
768	EEELKKFLEKLNNEEEQMRYQQYGFGQGHDA	-44.53	45.2	HHHHHHHHHHHCHHHHHHHHHHHHHCCCCCCCC
737	EEELKKFLEKYNNEAEQIRYQQNGFGQGHDA	-44.53	48.4	HHHHHHHHHHHHHHHHHHHHHHHHHHCCCCCCC
671	EEELKKFLEKYNNEAEQIRYQQQGFGQGHDA	-44.53	48.4	HHHHHHHHHHHHHHHHHHHHHHHHHHCCCCCCC
395	EEELKKFLEKYNNEAEQIYYQQNGFGQGHDA	-44.53	48.4	HHHHHHHHHHHHHHHHHHHHHHHHHCCCCCCCC
392	EEELKKFLEKYNNEAEQIYYQQNGFGQGHSA	-44.53	48.4	HHHHHHHHHHHHHHHHHHHHHHHHHCCCCCCCC
077	EEELKKFLEKYNNEAEQMRYQQQGFGQGHDA	-44.53	48.4	HHHHHHHHHHHHHHHHHHHHHHHHHHCCCCCCC
198	EEELKKFLEKYNNEEEQMRYQREGFGQGHDA	-44.53	45.2	HHHHHHHHHHHCCHHHHHHHHHHHHHHCCCCCC
992	EEELKKFLEKYNNEEEQMSYQQQGFGQGHDA	-44.53	45.2	HHHHHHHHHHHCHHHHHHHHHHHHHCCCCCCCC
202	EEELKKFLEKYNNESEQIRYQQNGFGQGHDA	-44.53	45.2	HHHHHHHHHHHHHHHHHHHHHHHHHCCCCCCCC
946	EEELKKFLEKYNNESEQIRYQQQGFGQGHDA	-44.53	45.2	HHHHHHHHHHHHHHHHHHHHHHHHHHCCCCCCC
571	EEELKKFLEKYNNESEQMRYQQQGFGQGHDA	-44.53	45.2	HHHHHHHHHHHHHHHHHHHHHHHHHHCCCCCCC
243	EEELKKFLEKYNNEYEQMFYQQKGFGQGHDA	-44.53	48.4	HHHHHHHHHHHHHHHHHHHHHHHHHHCCCCCCC
435	EEELRKFLEKYNNEYEQIFYQQKGFGQGHDA	-44.53	45.2	HHHHHHHHHHHHHHHHHHHHHHHHHHCCCCCCC
480	EQEARKFLEKYNNEAEQIYYQQNGFGQGHDA	-44.53	45.2	HHHHHHHHHHHHHHHHHHHHHHHHHCCCCCCCC
183	EEELKKFLEQYNQESEEMRKQQNGFGSGKDA	-44.52	38.7	HHHHHHHHHHHHHHHHHHHHHHHHHHCCCCCCC
034	EEELKKILEQLNQEEEQMKYQRYGFGQGHDA	-44.48	38.7	HHHHHHHHHHHHHHHHHHHHHHHHHCCCCCCCC
730	EEELKKLLEKLNQEQEQMSKQQQGFGSGKDA	-44.46	38.7	HHHHHHHHHHHHHHHHHHHHHHHHHHCCCCCCC
914	EEEARKFLEKYNQEAEEIYKQQNGFGSGKDA	-44.40	45.2	HHHHHHHHHHHHHHHHHHHHHHHHHCCCCCCCC
573	EEELKKILEQLNNEKEQMSYQQQGFGQGDSR	-44.38	45.2	HHHHHHHHHHHHHHHHHHHHHHHHHCCCCCCCC
375	EEELKKLLEKINNEAEQIRYQQNGFGQGDSR	-44.36	51.6	HHHHHHHHHHHHHHHHHHHHHHHHHCCCCCCCC
964	EEELKKLLEKLNNEEEQMKYQQQGFGQGDSR	-44.36	48.4	HHHHHHHHHHHCHHHHHHHHHHHHHCCCCCCCC
136	EEELRKLLEKINNEQEQISYQQNGFGQGDSR	-44.36	45.2	HHHHHHHHHHHHHHHHHHHHHHHHHCCCCCCCC
418	EEELRKLLEKLNNEQEQMRYQQNGFGQGDSR	-44.36	45.2	HHHHHHHHHHHHHHHHHHHHHHHHHCCCCCCCC
645	EEEARKFLEKFNQEYEQIFYQQKGFGQGHDA	-44.35	51.6	HHHHHHHHHHHHHHHHHHHHHHHHHHCCCCCCC
734	EEEARKFLEKYNQEEEQMRYQRQGFGQGHDA	-44.35	45.2	HHHHHHHHHHHHHHHHHHHHHHHHHHCCCCCCC
127	EEELKKLLEQINNEEEQMRYQQQGFGQGHDA	-44.34	38.7	HHHHHHHHHHHCHHHHHHHHHHHHHCCCCCCCC
878	EEELRKLLEQINNEEEQMRYQQYGFGQGHDA	-44.34	35.5	HHHHHHHHHHHCHHHHHHHHHHHHHCCCCCCCC
564	EEELKKLLEQINNEAEQIRYQQNGFGQGHDA	-44.32	41.9	HHHHHHHHHHHHHHHHHHHHHHHHHHCCCCCCC
665	EEELKKLLEQINNEAEQIRYQQNGFGQGHSA	-44.32	41.9	HHHHHHHHHHHHHHHHHHHHHHHHHHCCCCCCC
190	EEELKKLLEQINNEAEQMRYQQNGFGQGHDA	-44.32	41.9	HHHHHHHHHHHHHHHHHHHHHHHHHHCCCCCCC
550	EEELKKLLEQINNEEEQIRYQQQGFGQGHDA	-44.32	38.7	HHHHHHHHHHHCHHHHHHHHHHHHHCCCCCCCC
335	EEELKKLLEQINNEEEQMRYQQYGFGQGHDA	-44.32	38.7	HHHHHHHHHHHCHHHHHHHHHHHHHCCCCCCCC
214	EEELKKLLEQINNEKEQMEYQRAGFGQGHDA	-44.32	38.7	HHHHHHHHHHHHHHHHHHHHHHHHHHCCCCCCC
837	EEELKKLLEQLNNEEEQMKYQQGGFGQGHDF	-44.32	38.7	HHHHHHHHHHHCHHHHHHHHHHHHCCCCCCCCC
024	EEELKKLLEQLNNEEEQMKYQQNGFGQGHDA	-44.32	38.7	HHHHHHHHHHHCHHHHHHHHHHHHHHCCCCCCC
875	EEELKKLLEQLNNEKEQMSYQQQGFGQGHDA	-44.32	38.7	HHHHHHHHHHHHHHHHHHHHHHHHHCCCCCCCC
557	EEELKKLLEQLNNEKEQMSYQQQGFGQGHSA	-44.32	38.7	HHHHHHHHHHHHHHHHHHHHHHHHHCCCCCCCC
457	EEELKKLLEQLNNEQEQMRYQQNGFGQGHDA	-44.32	38.7	HHHHHHHHHHHHHHHHHHHHHHHHHHCCCCCCC
597	EEELKKLLEQLNNEQEQMRYQQQGFGQGHDA	-44.32	38.7	HHHHHHHHHHHHHHHHHHHHHHHHHCCCCCCCC
880	EEELKKLLEQLNNEQEQMRYQRQGFGQGHDA	-44.32	38.7	HHHHHHHHHHHHHHHHHHHHHHHHHHCCCCCCC
949	EEELKKLLEQLNNEQEQMRYQRYGFGQGHDA	-44.32	38.7	HHHHHHHHHHHHHHHHHHHHHHHHHCCCCCCCC
735	EEELRKLLEQINNEAEQMRYQQNGFGQGHDA	-44.32	38.7	HHHHHHHHHHHHHHHHHHHHHHHHHHCCCCCCC
690	EEELRKLLEQINNEAEQMSYQQQGFGQGHDA	-44.32	38.7	HHHHHHHHHHHHHHHHHHHHHHHHHCCCCCCCC
710	EEELRKLLEQINNEEEQMRYQQQGFGQGHDA	-44.32	35.5	HHHHHHHHHHHCHHHHHHHHHHHHHCCCCCCCC
791	EEELRKLLEQINNEEEQMRYQREGFGQGHDA	-44.32	35.5	HHHHHHHHHHHCHHHHHHHHHHHHHHHCCCCCC
410	EEELRKLLEQLNNEEEQMKYQQNGFGQGHDA	-44.32	35.5	HHHHHHHHHHHCHHHHHHHHHHHHHHCCCCCCC
517	EEELRKLLEQLNNEKEQMRYQRQGFGQGHDA	-44.32	35.5	HHHHHHHHHHHHHHHHHHHHHHHHHHCCCCCCC
947	EEELRKLLEQLNNEKEQMSYQQQGFGQGHDA	-44.32	35.5	HHHHHHHHHHHHHHHHHHHHHHHHHCCCCCCCC
927	EEELRKLLEQLNNEQEQMRYQREGFGQGHDA	-44.32	35.5	HHHHHHHHHHHHHHHHHHHHHHHHHHHCCCCCC
996	EQELRKLLEQINNEEEQMRYQQQGFGQGHDA	-44.32	32.3	HHHHHHHHHHHHHHHHHHHHHHHHHCCCCCCCC
330	EEEWKKLIEQINNDEEQMRYQQQGFGQGHDA	-44.32	32.3	HHHHHHHHHHHCCHHHHHHHHHHHHCCCCCCCC

167	EEEWKKFLEKYNNEAEQIYYQQNGFGQGHSA	-44.21	48.4	HHHHHHHHHHHHHHHHHHHHHHHCCCCCCCC
471	EEEARKFLEKFNNEEEQMSYQQQGFGQGHDA	-44.20	48.4	HHHHHHHHHHHHHHHHHHHHHHHCCCCCCCC
277	EEELKKILEQLNQEQEQMSYQKEGFGQGDSR	-44.16	45.2	HHHHHHHHHHHHHHHHHHHHHHHCCCCCCCC
793	EEELKKLLEKLNQEEEQMKYQQNGFGQGDSR	-44.15	48.4	HHHHHHHHHHHHHHHHHHHHHHHCCCCCCCC
124	EEELRKLLEQINQEEEQMRYQQQGFGQGHDA	-44.15	35.5	HHHHHHHHHHHHHHHHHHHHHHHCCCCCCCC
492	EEELKKFLEKFNNEYEEMFKQQKGFGSGKDA	-44.10	48.4	HHHHHHHHHHHHHHHHHHHHHHHCCCCCCCC
383	EEELKKFLEKYNNEAEEIRKQQGGFGSGKDA	-44.10	45.2	HHHHHHHHHHHHHHHHHHHHHHCCCCCCCCC
813	EEELKKFLEKYNNEAEEIRKQQNGFGSGKDA	-44.10	45.2	HHHHHHHHHHHHHHHHHHHHHHHCCCCCCCC
181	EEELKKFLEKFNQEYEQMFYQQKGFGQGDSR	-44.09	58.1	HHHHHHHHHHHHHHHHHHHHHHHCCCCCCCC
643	EEEARKFLEQYNQEAEQMRYQQQGFGQGHSA	-44.07	45.2	HHHHHHHHHHHHHHHHHHHHHHHCCCCCCCC
930	EEEARKFLEQFNQEYEQMFYQQKGFGQGHDA	-44.06	48.4	HHHHHHHHHHHHHHHHHHHHHHHHCCCCCCC
106	EEEARKFLEQFNQEYEQMYYQQQGFGQGHDA	-44.06	45.2	HHHHHHHHHHHHHHHHHHHHHHHCCCCCCCC
408	EEEARKFLEQLNQEEEQMRYQQQGFGQGHDA	-44.06	41.9	HHHHHHHHHHHHHHHHHHHHHHHCCCCCCCC
290	EEEARKFLEQYNQESEQMRYQQQGFGQGHDA	-44.06	41.9	HHHHHHHHHHHHHHHHHHHHHHHHCCCCCCC
439	EEELKKFLEQFNQEYEQMFYQQKGFGQGHDA	-44.06	48.4	HHHHHHHHHHHHHHHHHHHHHHHHCCCCCCC
913	EEELKKFLEQYNQEAEQIYYQQNGFGQGHDA	-44.06	45.2	HHHHHHHHHHHHHHHHHHHHHHHCCCCCCCC
967	EEELKKFLEQYNQEAEQMRYQQNGFGQGHSA	-44.06	45.2	HHHHHHHHHHHHHHHHHHHHHHHHCCCCCCC
137	EEELKKFLEQYNQEEEQMRYQREGFGQGHDA	-44.06	41.9	HHHHHHHHHHHHHHHHHHHHHHHHHCCCCCC
847	EEELKKFLEQYNQESEQMRYQQQGFGQGHDA	-44.06	41.9	HHHHHHHHHHHHHHHHHHHHHHHHCCCCCCC
527	EEELKKFLEKFNNEEEQMRYQRQGFGQGHDA	-44.05	48.4	HHHHHHHHHHHCHHHHHHHHHHHHCCCCCCC
142	EEELKKFLEKYNNEAEQMRYQQQGFGQGHSA	-44.05	48.4	HHHHHHHHHHHHHHHHHHHHHHHHCCCCCCC
728	EEEWKKFIEKLNNEEEQMRYQQQGFGQGHDA	-44.05	41.9	HHHHHHHHHHHCHHHHHHHHHHHCCCCCCCC
804	EEELKKLLEKLNNEQEQIRYQQGGFGQGDSR	-44.04	48.4	HHHHHHHHHHHHHHHHHHHHHHCCCCCCCCC
693	EEELRKLLEKINNEEEQIRYQQQGFGQGDSR	-44.04	45.2	HHHHHHHHHHHCHHHHHHHHHHHCCCCCCCC
729	EEEARKFLEKYNNESEQMRYQQQGFGQGDSR	-43.98	51.6	HHHHHHHHHHHHHHHHHHHHHHHCCCCCCCC
657	EEEARKFLEKYNNEAEQMRYQQQGFGQGHDA	-43.93	48.4	HHHHHHHHHHHHHHHHHHHHHHHHCCCCCCC
627	EEEAKKFLEKYNQEEEQMSYQQQGFGQGHDA	-43.93	48.4	HHHHHHHHHHHHHHHHHHHHHHHCCCCCCCC
180	EEELKKLLEQINNEKEEMEKQKQGFGSGKDA	-43.90	35.5	HHHHHHHHHHHHHHHHHHHHHHHHCCCCCCC
872	EEELKKLLEQLNNEEEEMKKQQNGFGSGKDA	-43.90	35.5	HHHHHHHHHHHHHHHHHHHHHHHHCCCCCCC
725	EEQLKKLLEQINNEEEQIRYQQQGFGQGHDA	-43.89	41.9	HHHHHHHHHHHCHHHHHHHHHHHCCCCCCCC
224	EESLKKLLEKINNEAEQIRYQQNGFGQGHDA	-43.87	45.2	HHHHHHHHHHHHHHHHHHHHHHHHCCCCCCC
585	EEELKKLLEKLNNEKEQMSYQQQGFGQGDSR	-43.85	48.4	HHHHHHHHHHHHHHHHHHHHHHHCCCCCCCC
325	EEELRKLLEKINNEEEQMRYQQQGFGQGDSR	-43.85	45.2	HHHHHHHHHHHCHHHHHHHHHHHCCCCCCCC
436	EEELRKLLEKINNEQEQMSYQQQGFGQGDSR	-43.85	45.2	HHHHHHHHHHHHHHHHHHHHHHHCCCCCCCC
908	EEELRKLLEKLNNEEEQMKYQKEGFGQGDSR	-43.85	45.2	HHHHHHHHHHHCHHHHHHHHHHHCCCCCCCC
386	EEELRKLLEKLNNEQEQMRYQRYGFGQGDSR	-43.85	45.2	HHHHHHHHHHHHHHHHHHHHHHHCCCCCCCC
662	EEELRKLLEQLNNEQEQMRYQQNGFGQGHDA	-43.85	35.5	HHHHHHHHHHHHHHHHHHHHHHHHCCCCCCC
026	EEELRKLLEQLNNEQEQMRYQRQGFGQGHDA	-43.85	35.5	HHHHHHHHHHHHHHHHHHHHHHHHCCCCCCC
314	EEELKKFLEKLNNEQEQMSYQQQGFGQGDSR	-43.80	51.6	HHHHHHHHHHHHHHHHHHHHHHHCCCCCCCC
256	EEELKKFLEKYNNEEEQMRYQRQGFGQGDDR	-43.80	51.6	HHHHHHHHHHHCCHHHHHHHHHHCCCCCCCC
499	EEELKKFLEKYNNEEEQMRYQREGFGQGHSA	-43.79	45.2	HHHHHHHHHHHCCHHHHHHHHHHHHCCCCCC
646	EEEARKFLENYNNRYEDMEYQRTGRGKGHDF	-43.78	45.2	HHHHHHHHHHHHHHHHHHHHHHCCCCCCCCC
957	EEEAKKFLEQFNNEEEQMSYQQQGFGQGHDA	-43.76	48.4	HHHHHHHHHHHHHHHHHHHHHHHCCCCCCCC
863	EEEAKKFLEQYNNEYEQMFYQQKGFGQGHDA	-43.76	48.4	HHHHHHHHHHHHHHHHHHHHHHHHCCCCCCC
376	EEEARKFLEQFNNEEEQMSYQQNGFGQGHDA	-43.76	45.2	HHHHHHHHHHHHHHHHHHHHHHHHCCCCCCC
280	EEEARKFLEQFNNEEEQMSYQQQGFGQGHDA	-43.76	45.2	HHHHHHHHHHHHHHHHHHHHHHHCCCCCCCC
204	EEEARKFLEQFNNEYEQMFYQQKGFGQGHDA	-43.76	48.4	HHHHHHHHHHHHHHHHHHHHHHHHCCCCCCC
307	EEEARKFLEQLNNEEEQMRYQQQGFGQGHDA	-43.76	41.9	HHHHHHHHHHHHHHHHHHHHHHHCCCCCCCC
411	EEEARKFLEQLNNEKEQMEYQRAGFGQGHDA	-43.76	41.9	HHHHHHHHHHHHHHHHHHHHHHHHCCCCCCC
857	EEEARKFLEQYNNEAEQIYYQQNGFGQGHDA	-43.76	45.2	HHHHHHHHHHHHHHHHHHHHHHHCCCCCCCC
584	EEEARKFLEQYNNEAEQMRYQQQGFGQGHDA	-43.76	45.2	HHHHHHHHHHHHHHHHHHHHHHHHCCCCCCC
271	EEEARKFLEQYNNEAEQMYYQQQGFGQGHDA	-43.76	45.2	HHHHHHHHHHHHHHHHHHHHHHHCCCCCCCC
745	EEEARKFLEQYNNEEEQMFYQQKGFGQGHDA	-43.76	45.2	HHHHHHHHHHHCHHHHHHHHHHHHCCCCCCC
860	EEEARKFLEQYNNEEEQMRYQQQGFGQGHDA	-43.76	41.9	HHHHHHHHHHHCHHHHHHHHHHHCCCCCCCC
013	EEEARKFLEQYNNEEEQMRYQREGFGQGHDA	-43.76	41.9	HHHHHHHHHHHCHHHHHHHHHHHHHCCCCCC
739	EEEARKFLEQYNNEEEQMRYQREGFGQGHSA	-43.76	41.9	HHHHHHHHHHHCHHHHHHHHHHHHHCCCCCC
576	EEEARKFLEQYNNEEEQMSYQQQGFGQGHDA	-43.76	41.9	HHHHHHHHHHHHHHHHHHHHHHHCCCCCCCC
617	EEEARKFLEQYNNESEQIRYQQNGFGQGHDA	-43.76	41.9	HHHHHHHHHHHHHHHHHHHHHHHCCCCCCCC
797	EEEARKFLEQYNNESEQIRYQQQGFGQGHDA	-43.76	41.9	HHHHHHHHHHHHHHHHHHHHHHHHCCCCCCC
721	EEEARKFLEQYNNESEQMRYQQQGFGQGHDA	-43.76	41.9	HHHHHHHHHHHHHHHHHHHHHHHHCCCCCCC
904	EEEARKFLEQYNNEYEQMFYQQKGFGQGHDA	-43.76	45.2	HHHHHHHHHHHHHHHHHHHHHHHHCCCCCCC
628	EEELKKFLEQFNNEQEQMTYQRYGFGQGHDA	-43.76	45.2	HHHHHHHHHHHHHHHHHHHHHHHCCCCCCCC
017	EEELKKFLEQFNNEYEQMFYQQKGFGQGHDA	-43.76	48.4	HHHHHHHHHHHHHHHHHHHHHHHHCCCCCCC
479	EEELKKFLEQFNNEYEQMFYQQQGFGQGHDA	-43.76	48.4	HHHHHHHHHHHHHHHHHHHHHHHHCCCCCCC
286	EEELKKFLEQLNNEEEQMRYQQQGFGQGHDA	-43.76	41.9	HHHHHHHHHHHHHHHHHHHHHHHCCCCCCCC
332	EEELKKFLEQLNNEEEQMRYQQYGFGQGHDA	-43.76	41.9	HHHHHHHHHHHHHHHHHHHHHHHCCCCCCCC
776	EEELKKFLEQYNNEAEQIYYQQNGFGQGHDA	-43.76	45.2	HHHHHHHHHHHHHHHHHHHHHHHCCCCCCCC

489	EEELKKFLEQYNNEAEQMRYQKEGFGQGHDA	-43.76	45.2	HHHHHHHHHHHHHHHHHHHHHHHHHHCCCCCCC
253	EEELKKFLEQYNNEAEQMRYQQNGFGQGHDA	-43.76	45.2	HHHHHHHHHHHHHHHHHHHHHHHHHHCCCCCCC
379	EEELKKFLEQYNNEAEQMRYQQQGFGQGHDA	-43.76	45.2	HHHHHHHHHHHHHHHHHHHHHHHHHHCCCCCCC
419	EEELKKFLEQYNNEAEQMRYQRQGFGQGHDA	-43.76	45.2	HHHHHHHHHHHHHHHHHHHHHHHHHHCCCCCCC
692	EEELKKFLEQYNNEEEQMFYQQKGFGQGHDA	-43.76	45.2	HHHHHHHHHHHCHHHHHHHHHHHHHHCCCCCCC
598	EEELKKFLEQYNNEEEQMRYQQQGFGQGHDA	-43.76	41.9	HHHHHHHHHHHCHHHHHHHHHHHHHCCCCCCCC
921	EEELKKFLEQYNNEEEQMRYQREGFGQGHEA	-43.76	41.9	HHHHHHHHHHHCHHHHHHHHHHHHHHHCCCCCC
556	EEELKKFLEQYNNEEEQMSYQQQGFGQGHDA	-43.76	41.9	HHHHHHHHHHHCHHHHHHHHHHHHHCCCCCCCC
890	EEELKKFLEQYNNESEQIRYQQNGFGQGHDA	-43.76	41.9	HHHHHHHHHHHHHHHHHHHHHHHHHCCCCCCCC
104	EEELKKFLEQYNNESEQMRYQQGGFGQGHDF	-43.76	41.9	HHHHHHHHHHHHHHHHHHHHHHHHCCCCCCCCC
292	EEELKKFLEQYNNESEQMRYQQQGFGQGHDA	-43.76	41.9	HHHHHHHHHHHHHHHHHHHHHHHHHHCCCCCCC
019	EQEARKFLEQYNNEEEQMRYQREGFGQGHDA	-43.76	38.7	HHHHHHHHHHHCHHHHHHHHHHHHHHHCCCCCC
938	EEELKKLLEQINQEAEQMRYQRYGFGQGHDA	-43.70	41.9	HHHHHHHHHHHHHHHHHHHHHHHHHCCCCCCCC
707	EEEARKFLEQFNQEYEEMFKQQKGFGSGKDA	-43.63	45.2	HHHHHHHHHHHHHHHHHHHHHHHHHCCCCCCCC
231	EEELKKLLEQINNEAEQIRYQQNGFGQGDSR	-43.59	48.4	HHHHHHHHHHHHHHHHHHHHHHHHHCCCCCCCC
401	EEELKKLLEQINNEEEQMRYQQQGFGQGDSR	-43.59	45.2	HHHHHHHHHHHCHHHHHHHHHHHHHCCCCCCCC
974	EEELKKLLEQINNEQEQMSYQQQGFGQGDSR	-43.59	45.2	HHHHHHHHHHHHHHHHHHHHHHHHHCCCCCCCC
680	EEELRKLLEQLNNEQEQMRYQQQGFGQGDSR	-43.59	41.9	HHHHHHHHHHHHHHHHHHHHHHHHHCCCCCCCC
724	EEEARKFLEKYNQEEEQMRYQREGFGQGDSR	-43.58	51.6	HHHHHHHHHHHHHHHHHHHHHHHHHHCCCCCCC
311	EEELKKFLEKLNQEEEQMRYQQQGFGQGDSR	-43.58	51.6	HHHHHHHHHHHHHHHHHHHHHHHHHCCCCCCCC
912	EEEARKFLEQLNQEEEQMRYQQYGFGQGHDA	-43.58	41.9	HHHHHHHHHHHHHHHHHHHHHHHHHCCCCCCCC
298	EEEARKFLEQYNQEYEQMEYQRSGFGQGHDA	-43.58	45.2	HHHHHHHHHHHHHHHHHHHHHHHHHHCCCCCCC
343	EEELKKFLEQYNQEEEQMRYQREGFGQGHSA	-43.58	41.9	HHHHHHHHHHHHHHHHHHHHHHHHHHHCCCCCC
473	EEELKKLLEKLNQEQEQIRYQQNGFGQGDSR	-43.48	48.4	HHHHHHHHHHHHHHHHHHHHHHHHHCCCCCCCC
811	EEELRKLLEKLNQEQEQIRYQQNGFGQGDSR	-43.48	45.2	HHHHHHHHHHHHHHHHHHHHHHHHHCCCCCCCC
697	EEEARKFLEKYNQEAEQRRYQQNGFGQGHDA	-43.43	48.4	HHHHHHHHHHHHHHHHHHHHHHHHHHCCCCCCC
208	EEELKKLLEQINQEAEQITYQQNGFGQGDSR	-43.38	48.4	HHHHHHHHHHHHHHHHHHHHHHHHHCCCCCCCC
923	EEELRKLLEQLNQEKEQMSYQQQGFGQGDSR	-43.38	41.9	HHHHHHHHHHHHHHHHHHHHHHHHHCCCCCCCC
063	EEEARKMLEQINNEEEQIRYQQQGFGQGHDA	-43.37	38.7	HHHHHHHHHHHHHHHHHHHHHHHHHCCCCCCCC
320	EEEARKMLEQINNEEEQMRYQQQGFGQGHDA	-43.37	38.7	HHHHHHHHHHHHHHHHHHHHHHHHHCCCCCCCC
592	EEEARKFLEQYNNEAEEIYKQQNGFGSGKDA	-43.34	41.9	HHHHHHHHHHHHHHHHHHHHHHHHHCCCCCCCC
350	EEEARKFLEKFNNEYEQMYYQQKGFGQGDSR	-43.29	54.8	HHHHHHHHHHHHHHHHHHHHHHHHHCCCCCCCC
871	EEEARKFLEKLNNEEEQMRYQREGFGQGDSR	-43.29	51.6	HHHHHHHHHHHHHHHHHHHHHHHHHCCCCCCCC
025	EEEARKFLEKYNNEAEQIRYQQNGFGQGDSR	-43.29	54.8	HHHHHHHHHHHHHHHHHHHHHHHHHCCCCCCCC
005	EEEARKFLEKYNNEAEQIYYQQNGFGQGDDR	-43.29	54.8	HHHHHHHHHHHHHHHHHHHHHHHHHCCCCCCCC
178	EEELKKFLEKLNNEEEQMQYQRSGFGQGDSR	-43.29	54.8	HHHHHHHHHHHCHHHHHHHHHHHHHHCCCCCCC
924	EEELKKFLEKLNNEEEQMRYQQQGFGQGDSR	-43.29	51.6	HHHHHHHHHHHCHHHHHHHHHHHHHCCCCCCCC
779	EEELKKFLEKLNNEKEQIEYQRKGFGQGDSR	-43.29	51.6	HHHHHHHHHHHHHHHHHHHHHHHHHCCCCCCCC
603	EEELKKFLEKYNNEAEQIYYQQNGFGQGDSR	-43.29	54.8	HHHHHHHHHHHHHHHHHHHHHHHHHCCCCCCCC
100	EEELKKFLEKYNNESEQIRYQQQGFGQGDDR	-43.29	51.6	HHHHHHHHHHHHHHHHHHHHHHHHHCCCCCCCC
267	EEELKKFLEQYNNEAEQIYYQQNGCGQGHDY	-43.29	45.2	HHHHHHHHHHHHHHHHHHHHHHHHHCCCCCCCC
951	EEELKKFLEQYNNEAEQMRYQQGGFGQGHDF	-43.29	45.2	HHHHHHHHHHHHHHHHHHHHHHHHCCCCCCCCC
229	EEELKKFLEQLNQEEEQMRYQQQGFGQGDSR	-43.25	48.4	HHHHHHHHHHHHHHHHHHHHHHHHHCCCCCCCC
236	EEELKEFLKQYNEEAEDIYYQQNGFGKGDSR	-43.25	58.1	HHHHHHHHHHHHHHHHHHHHHHHHHCCCCCCCC
669	EEEARKFLEKYNNESEQIRYQQQGFGQGDSR	-43.20	51.6	HHHHHHHHHHHCHHHHHHHHHHHHHCCCCCCCC
887	EEELKKLLEKLNNEEEQIKYQQNGFGQGDSR	-43.18	48.4	HHHHHHHHHHHCHHHHHHHHHHHHHCCCCCCCC
614	EEELKKLLEKLNNEQEQMRYQQNGFGQGDDR	-43.18	48.4	HHHHHHHHHHHHHHHHHHHHHHHHHCCCCCCCC
333	EEEARKFLQQYNNEAEQMRYQQQGFGQGHDA	-43.12	45.2	HHHHHHHHHHHHHHHHHHHHHHHHHHCCCCCCC
942	EEELKKFLQQYNNEEEQMSYQKEGFGQGHDA	-43.12	41.9	HHHHHHHHHHHHHHHHHHHHHHHHHHCCCCCCC
613	EQELRKLLEQINNEEEQMRYQQQGFGQGDSR	-43.10	38.7	HHHHHHHHHHHHHHHHHHHHHHHHHCCCCCCCC
030	EESLRKLLEQINNEAEQMSYQQQGFGQGHDA	-43.10	38.7	HHHHHHHHHHHHHHHHHHHHHHHHHCCCCCCCC
268	EEELKKLLEQINNEAEQMRYQQNGFGQGDSR	-43.09	48.4	HHHHHHHHHHHHHHHHHHHHHHHHHCCCCCCCC
609	EEELKKLLEQINNEEEQMRYQQYGFGQGDSR	-43.09	45.2	HHHHHHHHHHHCHHHHHHHHHHHHHCCCCCCCC
099	EEELKKLLEQLNNEKEQMSYQQQGFGQGDSR	-43.09	45.2	HHHHHHHHHHHHHHHHHHHHHHHHHCCCCCCCC
359	EEELRKLLEQINNEAEQMRYQRQGFGQGDSR	-43.09	45.2	HHHHHHHHHHHHHHHHHHHHHHHHHCCCCCCCC
105	EEELRKLLEQINNEEEQMRYQQQGFGQGDSR	-43.09	41.9	HHHHHHHHHHHCHHHHHHHHHHHHHCCCCCCCC
759	EEELRKLLEQINNEEEQMRYQQYGFGQGDSR	-43.09	41.9	HHHHHHHHHHHCHHHHHHHHHHHHHCCCCCCCC
396	EEELRKLLEQLNNEKEQMSYQQQGFGQGDSR	-43.09	41.9	HHHHHHHHHHHHHHHHHHHHHHHHHCCCCCCCC
929	EEELKKFLEKLNNEAEQMQYQRAGFGQGDSR	-43.08	54.8	HHHHHHHHHHHHHHHHHHHHHHHHHHCCCCCCC
176	EEEARKFLEQFNNEEEQMSYQQQGFGQGDSR	-43.03	51.6	HHHHHHHHHHHHHHHHHHHHHHHHHCCCCCCCC
140	EEEARKFLEQYNNEAEQMRYQQQGFGQGDSR	-43.03	51.6	HHHHHHHHHHHHHHHHHHHHHHHHHCCCCCCCC
812	EEELKKFLEQYNNEAEQMRYQQNGFGQGDSR	-43.03	51.6	HHHHHHHHHHHHHHHHHHHHHHHHHCCCCCCCC
344	EEELKKFLEQYNNEEEQMSYQQNGFGQGDSR	-43.03	48.4	HHHHHHHHHHHCHHHHHHHHHHHHHCCCCCCCC
347	EEELKKFLEQYNNEYEQMEYQRSGFGQGDSR	-43.03	51.6	HHHHHHHHHHHHHHHHHHHHHHHHHCCCCCCCC
695	EEELKKFLEKYNQEEEQRRYQQQGFGQGHDA	-42.95	45.2	HHHHHHHHHHHCHHHHHHHHHHHHHCCCCCCCC
666	EEEARKFLEQYNNEYEQMEYQRTGRGQGHDF	-42.84	41.9	HHHHHHHHHHHHHHHHHHHHHHHHHCCCCCCCC
841	EEEARKFLEQFNQEEEQMSYQQNGFGQGDSR	-42.82	51.6	HHHHHHHHHHHHHHHHHHHHHHHHHCCCCCCCC

420	EEEARKFLEQYNQEEEQMSYQQQGFGQGDSR	-42.82	48.4	HHHHHHHHHHHHHHHHHHHHHHHCCCCCCCC
220	EEELKKFLEQFNQEYEQMFYQQKGFGQGDSR	-42.82	54.8	HHHHHHHHHHHHHHHHHHHHHHHCCCCCCCC
041	EEELKKFLEQYNQEEEQMSYQKEGFGQGDSR	-42.82	48.4	HHHHHHHHHHHHHHHHHHHHHHHCCCCCCCC
792	EEELKKFLEQYNQEEEQMSYQQQGFGQGDSR	-42.82	48.4	HHHHHHHHHHHHHHHHHHHHHHHCCCCCCCC
270	EEELKKLLEQLNQEQEQMRYQQQGFGQGDSR	-42.71	45.2	HHHHHHHHHHHHHHHHHHHHHHHCCCCCCCC
476	EEELKKLLEQINQEEEQMRYQQQGFGQGDSR	-42.69	45.2	HHHHHHHHHHHHHHHHHHHHHHHCCCCCCCC
132	EEEARKFLQQYNNEAEQMRYQQNGFGQGHDA	-42.65	45.2	HHHHHHHHHHHHHHHHHHHHHHHHCCCCCCC
560	EEEARKFLEKFNNEEEQMRYQREGFGQGDSR	-42.62	54.8	HHHHHHHHHHHCHHHHHHHHHHHHCCCCCCC
319	EEELKKFLEKFNNEAEQMSYQQQGFGQGDSR	-42.62	58.1	HHHHHHHHHHHHHHHHHHHHHHHCCCCCCCC
095	EEEARKFLEQLNNEEEQMRYQQYGFGQGDSR	-42.55	48.4	HHHHHHHHHHHHHHHHHHHHHHHCCCCCCCC
072	EEELKKFLEQYNNEYEQMFYQQKGFGQGDSR	-42.55	51.6	HHHHHHHHHHHHHHHHHHHHHHHCCCCCCCC
581	EEEARKFLEQFNNEYEQMFYQQKGFGQGDSR	-42.52	54.8	HHHHHHHHHHHHHHHHHHHHHHHCCCCCCCC
059	EEEARKFLEQYNNEEEQMRYQRQGFGQGDSR	-42.52	48.4	HHHHHHHHHHHCHHHHHHHHHHHCCCCCCCC
155	EEELKKFLEQFNNEYEQMFYQQKGFGQGDSR	-42.52	54.8	HHHHHHHHHHHHHHHHHHHHHHHCCCCCCCC
495	EEELKKFLEQYNNEAEQIYYQQNGFGQGDSR	-42.52	51.6	HHHHHHHHHHHHHHHHHHHHHHHCCCCCCCC
076	EEELKKFLEQYNNEAEQMRYQQQGFGQGDSR	-42.52	51.6	HHHHHHHHHHHHHHHHHHHHHHHCCCCCCCC
170	EEELKKFLEQYNNEYEQMEYQRKGFGQGDSR	-42.52	48.4	HHHHHHHHHHHHHHHHHHHHHHHCCCCCCCC
536	EQEARKFLEQFNNEEEQMSYQQQGFGQGDSR	-42.52	48.4	HHHHHHHHHHHHHHHHHHHHHHHCCCCCCCC
064	EEEARKFLEKYNNEEEQMRYQREGFGQGDSR	-42.30	51.6	HHHHHHHHHHHCCHHHHHHHHHHHCCCCCCC
367	EEEARKFLEQFNQEYEQMFYQQKGFGQGDSR	-42.15	54.8	HHHHHHHHHHHHHHHHHHHHHHHCCCCCCCC
150	EEELKKFLEQYNQEEEQMRYQREGFGQGDSR	-42.15	48.4	HHHHHHHHHHHHHHHHHHHHHHHHCCCCCCC
998	EEELKKFLEQYNQESEQIRYQQQGFGQGDSR	-42.15	48.4	HHHHHHHHHHHHHHHHHHHHHHHCCCCCCCC
125	EEELKKFLEQYNQESEQMRYQQGGFGQGDSR	-42.15	48.4	HHHHHHHHHHHHHHHHHHHHHHCCCCCCCCC
139	EEELRRLLEKINNEEEQMRYQQYGFGQGDSR	-41.95	45.2	HHHHHHHHHHHCHHHHHHHHHHHCCCCCCCC
357	EEEARKFLEQLNNEEEQMRYQQQGFGQGDSR	-41.85	48.4	HHHHHHHHHHHHHHHHHHHHHHHCCCCCCCC
145	EEEARKFLEQYNNESEQMRYQKEGFGQGDSR	-41.85	48.4	HHHHHHHHHHHHHHHHHHHHHHHHCCCCCCC
944	EEELKKFLEQLNNEEEQMRYQQQGFGQGDSR	-41.85	48.4	HHHHHHHHHHHHHHHHHHHHHHHCCCCCCCC
926	EEEARKFLEQYNNEAEQMRYQQNGFGQGDSR	-41.62	51.6	HHHHHHHHHHHHHHHHHHHHHHHCCCCCCCC
364	EESLKKFLEQYNNEEEQMSYQQQGFGQGDSR	-41.30	48.4	HHHHHHHHHHHHHHHHHHHHHHHCCCCCCCC
